# Linking periodontitis with 20 cancers, emphasis on oropharyngeal cancer: a Mendelian randomization analysis

**DOI:** 10.1038/s41598-024-63447-4

**Published:** 2024-05-31

**Authors:** Jun Xiong, Hao Liu, Conghua Li, Yong Li, Jiali Feng

**Affiliations:** 1 Department of Stomatology, The Second Affiliated Hospital of Chongqing Medical University, Chongqing, China; 2 Stomatological Hospital of Chongqing Medical University, Chongqing, China; 3 Chongqing Key Laboratory of Oral Diseases, Chongqing, China; 4 Chongqing Municipal Key Laboratory of Oral Biomedical Engineering of Higher Education, Chongqing, China

**Keywords:** Periodontitis, Cancer, Mendelian randomization, Causal relationship, Genomics, Cancer, Computational biology and bioinformatics, Genetics, Oncology

## Abstract

While associations between periodontitis and an elevated risk of cancer have been suggested, the results of existing observational studies have been inconsistent, also leaving room for further investigation into the underlying mechanisms. This study was designed to delve into the possible causal link between periodontitis and 20 standard cancers while concurrently identifying potential mediators. We initiated a Mendelian randomization analysis that drew from either publicly accessible or personally obtained genome-wide association study (GWAS) datasets. The inverse variance weighting (IVW) method served as our primary tool for analysis. To ensure the strength and consistency of our results, we implemented additional strategies, including weighted median, weighted mode, MR-Egger regression, and MR pleiotropy residual sum and outlier (MR-PRESSO), bolstered by funnel plots. Our analysis unveiled an elevated risk of head and neck cancer concomitant with periodontitis (p = 0.041, OR 0.999, 95% CI 0.999–1.000), specifically a heightened risk of oropharyngeal cancer (p = 0.022, OR 0.999, 95% CI 0.999–1.000). As a result of probing into potential mediators, Fusobacterium nucleatum emerged as a likely intermediary in the promoting effect of periodontitis on oropharyngeal cancer (p = 0.021, OR 0.999, 95% CI 0.998–1.000). Inversely, basal cell carcinoma and endometrial cancer demonstrated an association with an increased incidence of periodontitis (basal cell carcinoma: p = 0.020, OR 0.987, 95% CI 0.976–0.998; endometrial cancer: p = 0.027, OR 0.984, 95% CI 0.970–0.998). However, periodontitis exerted no significant causal impact on the 19 other common cancers or the three subtypes of head and neck cancer. To conclude, our results support the theory that periodontitis contributes to an enhanced risk of head and neck cancer, particularly oropharyngeal cancer, with Fusobacterium nucleatum functioning as a potential intermediary.

## Introduction

Periodontitis, a pervasively consequential public health concern worldwide, operates as a microbiota-mediated affliction characterized by inflammation and immune response^1^. Over the past decade (2011–2020), the global incidence of periodontitis has grown alarmingly, affecting 62% of the population^2^, a marked increase from the estimated prevalence in 1990–2010^3^. Of this figure, severe periodontitis constitutes 23.6%, surpassing earlier approximations^4^.The major pathogenic progression of periodontitis initiates subtly with barely noticeable bleeding, which can lead to impaired chewing functionality and, ultimately, tooth loss^5^. This progression has a significant detrimental impact on an individual’s wellbeing^6^ while also placing a substantial economic strain on societal resources^7^.

Moreover, periodontitis and systemic health are intrinsically linked. A wealth of evidence underscores periodontitis’ role in exacerbating the initiation and progress of several systemic diseases, such as atherosclerosis, chronic gastritis, peptic ulcers, aspiration pneumonia, systemic lupus erythematosus, arthritis, and obesity^8^. Certain observational studies have even proposed periodontitis as a potential catalyst for cancer, inciting further focused research to corroborate this hypothesis^9^. Since the collaborative 2012 workshop between the European Federation of Periodontology and the American Academy of Periodontology, countless epidemiological studies have been carried out, albeit limited intervention studies and a lack of randomized controlled trials. These studies have scrutinized associations with various types of cancer, such as head and neck cancer^10^, digestive system cancers^11^, lung cancer^12^, breast cancer^13^, and urogenital system cancers^14^, yielding somewhat contentious results. While some perspectives uphold the theory of periodontitis as a cancer promoter, others derived from similar studies question this correlation. Thus, the actual relationship between periodontitis and cancer necessitates further clarification. Additionally, the intervening mechanisms at play in this association are relatively uncharted territory and warrant further in-depth investigation. However, the traditional gold-standard randomized controlled trials (RCTs), involving randomization and blinding, are typically precluded due to inherent limitations pertaining to the study subjects. Therefore, relying exclusively on current observational studies may not provide thorough and decisive findings.

The advent of advanced genetics research, particularly large-scale genome-wide association studies (GWAS), has spotlighted Mendelian randomization analysis as a highly promising alternative solution^15^. This approach leverages specific genetic variants as substitutes for exposure, facilitating the simulation of randomization in line with Mendel’s law of independent assortment to offspring. This thereby enables the exploration of the causal relationships between exposure and expected outcomes. The logical robustness of the causal inferences generated by Mendelian randomization has been successfully demonstrated in a multitude of studies^16^. However, the implementation of Mendelian randomization is not without its challenges. Primarily, there must be sufficiently strong instrumental variables, i.e. genetic variations that are substantially associated with the exposure, to ensure effective randomization. Secondly, the genetic variants utilised ought not to affect any other paths to the outcome variables, aside from through the exposure. This would limit confounding, but such an ideal scenario may be hard to achieve in practice, as there may be genetic pleiotropy or linkage disequilibrium. Despite these challenges, Mendelian randomization analysis is still considered a powerful complementary tool for unveiling potential causal relationships. In this area, we seek to uncover potential causal relationships between periodontitis and 20 common cancers and identify potential mediators, seeking to offer insights pertinent to disease prevention, epidemiological public policy, and comprehensive disease management.

## Materials and methods

### Research design

**Figure 1:**
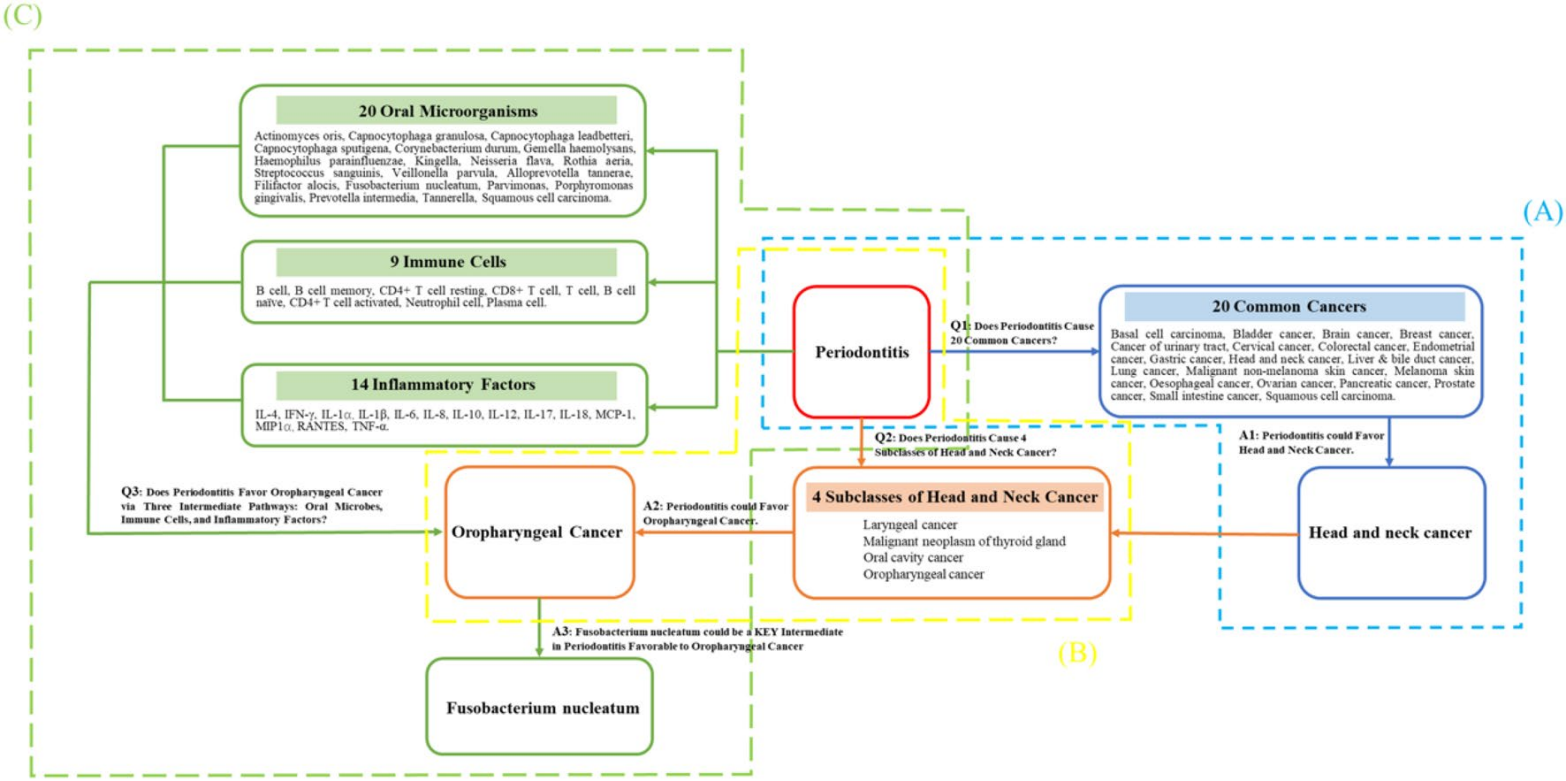
Flow chart: questions and answers.

Our research methodology was rigorously aligned with the three fundamental considerations of Mendelian randomization^17^. All data that was utilized had either pre-existing public availability or was endorsed for use. Our investigation systematically addressed the following inquiries: (A) is there a bidirectional causal relationship between periodontitis and 20 common cancers, specifically, does periodontitis escalate the risk of cancer, and conversely, does cancer enhance the incidence of periodontitis? (B) Does periodontitis act as a catalyst for the manifestation of four subgroups of head and neck cancer? (C) What might be the intermediary variables propelling the elevated risk of oropharyngeal cancer due to periodontitis? Could this be owed to shifts in periodontal microbial composition, inflammatory factors, or immune cell alterations? Figure 1 provides a detailed overview of these questions (Q) along with the respective responses (A), and it additionally outlines the conjectures and segments, reflecting the logical framework of this study.

### Data source

Our data on periodontitis was sourced from the most comprehensive combined dataset available to date: the latest meta-analysis by the gene-lifestyle interactions in dental endpoints (GLIDE) consortium, encompassing 17,353 periodontitis patients and 28,210 controls^18^. The diagnosis of periodontitis was confirmed based on the criteria set by the Centre for Disease Control/American Academy of Periodontology (CDC/AAP) or the community periodontal index (CPI). Access to the data can be obtained by downloading from the link provided in the original article (https://data.bris.ac.uk/data/dataset/2j2rqgzedxlq02oqbb4vmycnc2).

Data concerning 20 common cancers, four subtypes of head and neck cancer, and nine notable changes in immune cell factors triggered by periodontitis were derived from the public GWAS database—OpenGWAS (https://gwas.mrcieu.ac.uk/), one of the largest freely accessible GWAS databases. Details regarding study ID, phenotype, sample size, and number of genetic variant sites have been provided in Supplement S1.

Details on 20 periodontitis-specific oral microbial genetic changes were gathered from publicly available articles by Xiaomin Liu et al.^19^, encompassing 2017 dorsal tongue sample studies and 1915 saliva sample studies. Access to this restricted data was obtained in accordance with the guidelines for protecting individual privacy established by the Chinese human genetic resources administration and gene organ review committee (https://db.cngb.org/search/project/CNP0001664). However, we are not permitted to share these raw data.

Data featuring 14 periodontitis-specific changes in inflammatory factors was collected from openly accessible data from Ahola-Olli et al., which pertains to a participant pool of 8293 individuals^20^. We downloaded the data from the provided public site (https://data.bris.ac.uk/data/dataset/3g3i5smgghp0s2uvm1doflkx9x).

### Selection of genetic instruments

The genetic tools chosen for our study underwent a two-stage rigorous pre-selection process. Initially, entry prerequisites for pre-selection at the significance level p were set less than 1 × 10^−6^, which provided us with preliminary instruments^21^. In the subsequent refinement stage, these criteria were further refined, setting linkage disequilibrium screening with R2 < 0.001 and a window size of 10,000 kb^22^. The strength and reliability of our genetic instruments were also ensured by excluding the target SNP from the outcome GWAS dataset to prevent potential Type II error and manually filtering out probable SNPs with intermediate impacts^23^. The rigour of this selection process ensured that we drew robust conclusions based on genetic instruments that were strongly associated with our exposure of interest.

### Statistical analysis

We employed a range of complementary statistical analysis methods to ensure the roustness and reliability of our results. Initially, we used the inverse variance weighted (IVW) method as our primary analysis tool. However, understanding the limitation of relying solely on one method, we also incorporated additional strategies such as the weighted median, weighted mode, MR-Egger regression, and MR pleiotropy residual sum and outlier (MR-PRESSO) for cross-verification. These methods helped us to procure fairly reliable effect estimates. Among these, the IVW method was considered as the primary evaluation criteria, while the rest acted as supplementary.

Simultaneously, to ensure the robustness and consistency of our results, we performed tests for sensitivity and heterogeneity. We conducted the MR-Egger regression analysis to gauge the potential for horizontal pleiotropy and carried out the MR-PRESSO test to assess the existence of gene pleiotropy. In situations where horizontal pleiotropy was detected, we corrected it by removing outliers and compared the changes in causal effects before and after outlier removal. We also calculated Cochran’s Q statistic to ascertain if heterogeneity existed between the IVW and MR-Egger regression methods, using the difference in significance (p) as a determinant. Last but not the least, we also constructed a funnel plot to verify the overall robustness and consistency of our results. This comprehensive statistical approach and sensitivity analysis strategy robustly ensured the reliability and validity of the results of our investigation.

All analyses were conducted using the R 3.5.3 software, and the R packages used in the study were procured from formal resources and utilized in accordance with the technical manual.

### Ethics approval

This research utilized publicly accessible identified data from participant studies that had received approval from an ethics committee regarding human experimentation. No additional ethical clearance was necessary for this study.

## Results

### Instrument selection

In our study involving 64 exposure factors related to periodontal disease, we meticulously selected an array of high-quality SNP clusters as instrumental variables. The specific number of SNPs varies across different groups, with a maximum of 127 and a minimum of 7. A detailed list of SNPs and associated data can be found in Supplement S2. During the selection of these instrumental variables, we adhered to strict criteria, ensuring that each chosen genetic variable had a profound association with the exposure factors of the study, maintained their independence, and we made every effort to eradicate SNPs that could trigger intermediary effects. We meticulously handled each step to prevent potential weak instrument bias, ensuring the accuracy and reliability of our study results. In certain phases of the analysis, we also adopted suitable strategies, such as reducing the number of SNPs when necessary, to mitigate potential overlaps. The implementation of these stepping stones and strategies was designed to satisfy necessary assumptions and align with our primary screening benchmarks. In Supplement S2, we have provided a detailed display of all the screening procedures and outcomes, offering a comprehensive and profound understanding.

### Causal influence of periodontitis on 20 common cancers

**Figure 2:**
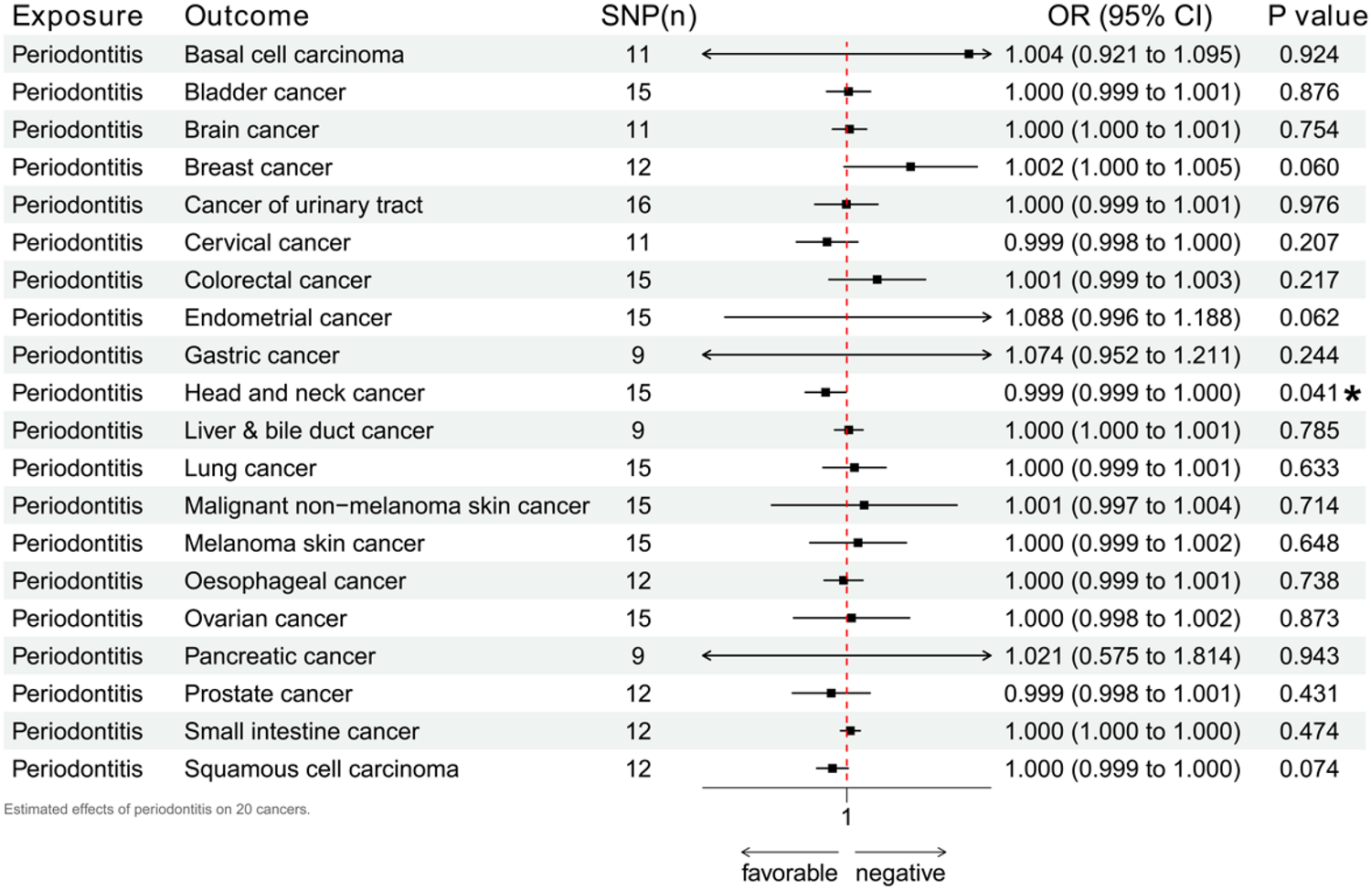
Estimated effects of periodontitis on 20 common cancers.

MR estimates for different methods, sensitivity and heterogeneity estimates, and visual charts are summarized in Supplement S3. We specially discuss the IVW method here. Figure 2 is an essential demonstration of the IVW results. An increased risk of periodontitis may promote an increased risk of head and neck cancer (p = 0.041, OR 0.999, 95% CI 0.999–1.000). MR-Egger, weighted median, and weighted mode methods showed consistent impact tendencies. Heterogeneity and multi-directionality checks did not show decisive opposition. The remaining 19 cancers (basal cell carcinoma, bladder cancer, brain cancer, breast cancer, cancer of urinary tract, cervical cancer, colorectal cancer, endometrial cancer, gastric cancer, liver & bile duct cancer, lung cancer, malignant non-melanoma skin cancer, melanoma skin cancer, oesophageal cancer, ovarian cancer, gastric cancer, liver & bile duct cancer, lung cancer, malignant non-melanoma skin cancer, melanoma skin cancer, oesophageal cancer, ovarian cancer, pancreatic cancer, prostate cancer, small intestine cancer, squamous cell carcinoma) did not show statistically significant impacts.

### Causal influence of periodontitis on four subtypes of head and neck cancer

**Figure 3:**
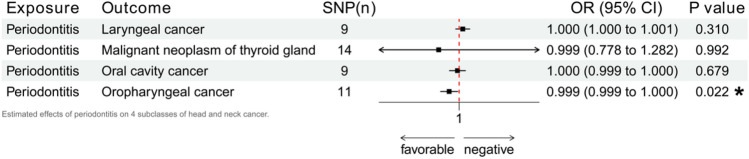
Estimated effects of periodontitis on 4 subclasses of head and neck cancer.

Furthermore, head and neck cancer refers to a series of cancers that occur in the anatomical regions of the head and neck. We painstakingly teased apart its four main subtypes, namely laryngeal cancer, malignant neoplasm of thyroid gland, oral cavity cancer and oropharyngeal cancer. Supplement S4 shows detailed results, while Fig. 3 is our major focus for IVW results. The risk of periodontitis may potentially drive an increased risk of oropharyngeal cancer (p = 0.022, OR 0.999, 95% CI 0.999–1.000), with other results in agreement. Heterogeneity and sensitivity tests found no decisive anomalies. The other three subtypes of head and neck cancer did not show positive responses.

### Impact of three possible mediators

The occurrence, progression, and manifestation of periodontitis mainly operate through changes in the features of oral microorganisms, inflammation factors, and immune cells. Therefore, the function of periodontitis in promoting the risk of oropharyngeal cancer may be mediated by these three factors.

**Figure 4:**
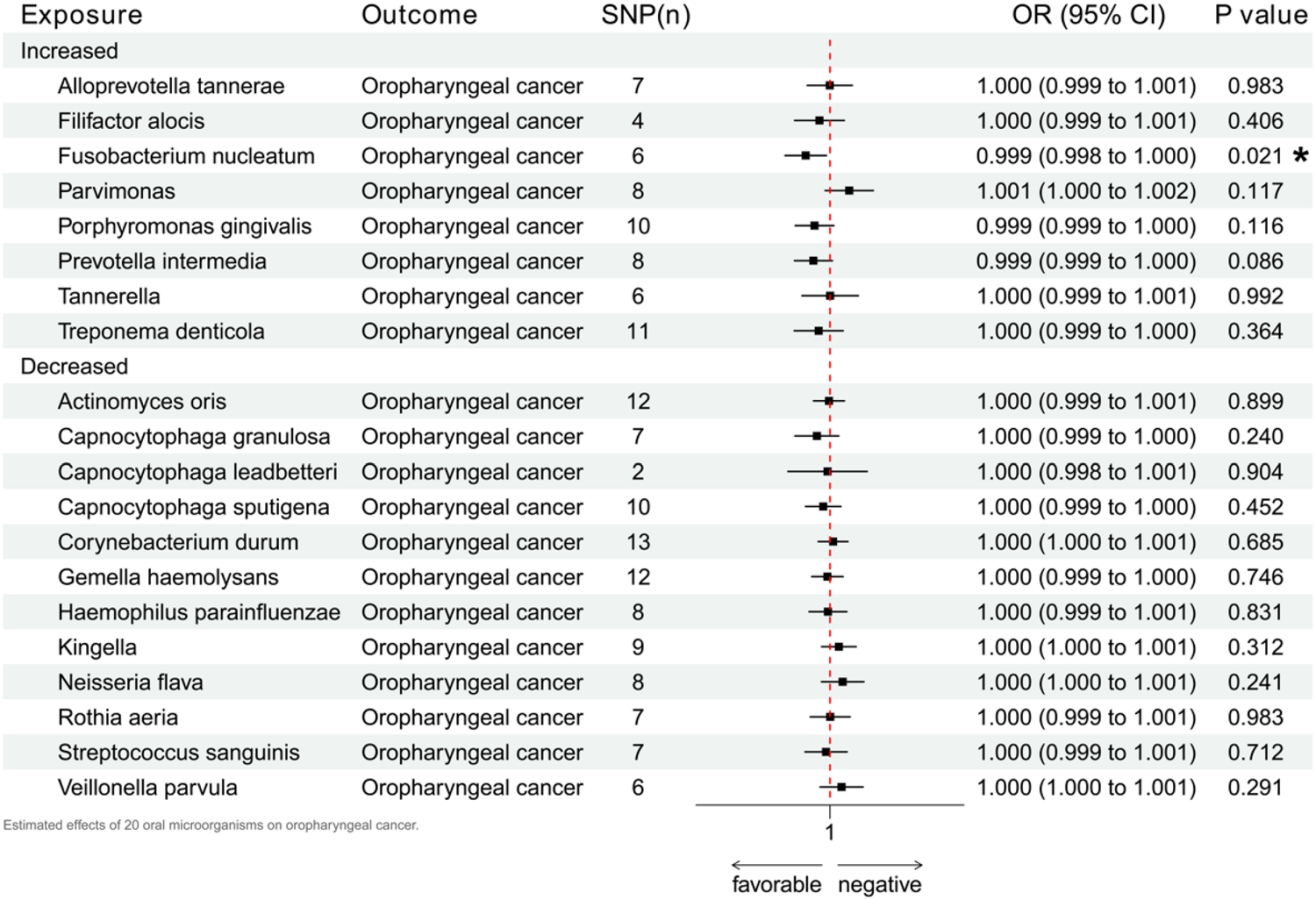
Estimated effects of 20 oral microorganisms on oropharyngeal cancer.

The occurrence of periodontitis can lead to changes in the community of oral microbes, most notably an increase in 19 types of oral microbes and a decrease in 21 types of oral microbes^24^. Restricted by the current oral microbial GWAS data, we only analyzed the possible mediating roles of 20 oral microbes, including eight relative increases (Alloprevotella tannerae, Filifactor alocis, Fusobacterium nucleatum, Parvimonas, Porphyromonas gingivalis, Prevotella intermedia, Prevotella intermedia Tannerella and Treponema denticola) and 12 relative decreases (Actinomyces oris, Capnocytophaga granulosa, Capnocytophaga leadbetteri, Capnocytophaga sputigena. Corynebacterium durum, Gemella haemolysans, Haemophilus parainfluenzae, Kingella, Neisseria flava, Rothia aeria, Streptococcus sanguinis and Veillonella parvula). The evidence for changes in 20 oral microbes due to the presence of periodontitis is positive, so our mediator analysis only discusses whether these 20 microbes would increase the risk of oropharyngeal cancer. The MR assessment and chat for all the methods are in Supplement S5, while Fig. 4 exhibits the main results. The presence of Fusobacterium nucleatum could possibly lead to an increased risk of oropharyngeal cancer (p = 0.021, OR 0.999, 95% CI 0.998–1.000), with other results and quality assessments in agreement. The occurrence of periodontitis can lead to an increased dominance of F. nucleatum, matching the change in direction of this bacterium’s dominant promotion of oropharyngeal cancer. Thus, F. nucleatum could be an intermediate factor mediating the functionality of periodontitis in promoting oropharyngeal cancer. None of the other 19 microbes had any significant results.

**Figure 5:**
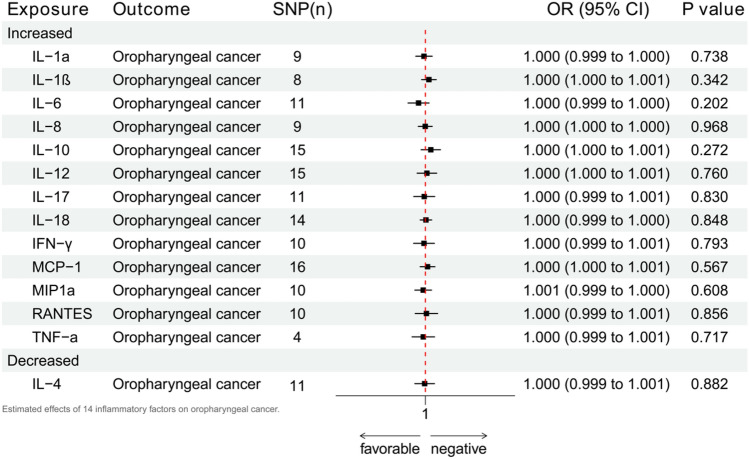
Estimated effects of 14 inflammatory factors on oropharyngeal cancer.

The occurrence of periodontitis can lead to concurrent changes in 15 inflammation factors^25^. Combinining this with the currently available GWAS research data, we explored the possible impacts of 14 of these, including 13 increases (IL-1a, IL-1ß, IL-6, IL-8, IL-10, IL-12, IL-17, IL-18, IFN-γ, MCP-1, MIP1a, RANTES, and TNF-a) and one decrease (IL-4). The complete results are in Supplement S6, while Fig. 5 exhibits the IVW results. For all the 14 inflammation factors, there was no significant impact seen on oropharyngeal cancer.

**Figure 6:**
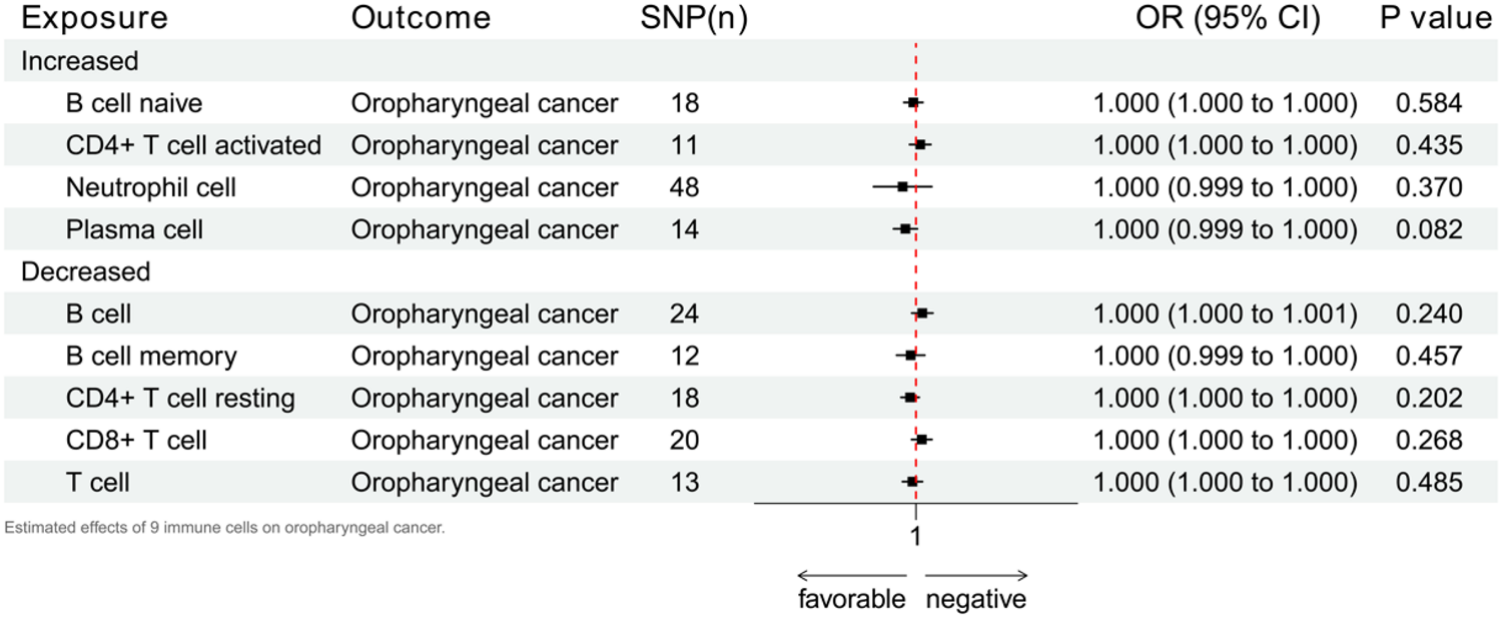
Estimated effects of 9 immune cells on oropharyngeal cancer.

The presence of periodontitis can trigger the response of 22 different types and states of immune cells^26^. In our research, we discussed the effects of nine immune cells, including four increases (B cell naïve, CD4+ T cell activated, neutrophil cell, and plasma cell) and five decreases (B cell, B cell memory, CD4+ T cell resting, CD8+ T cell, and T cell). The complete results are in Supplement S7, while Fig. 6 exhibits the IVW results. For the nine immune cells, none had any meaningful effects on oropharyngeal cancer.

### Causal influence of 20 common cancers on periodontitis

**Figure 7:**
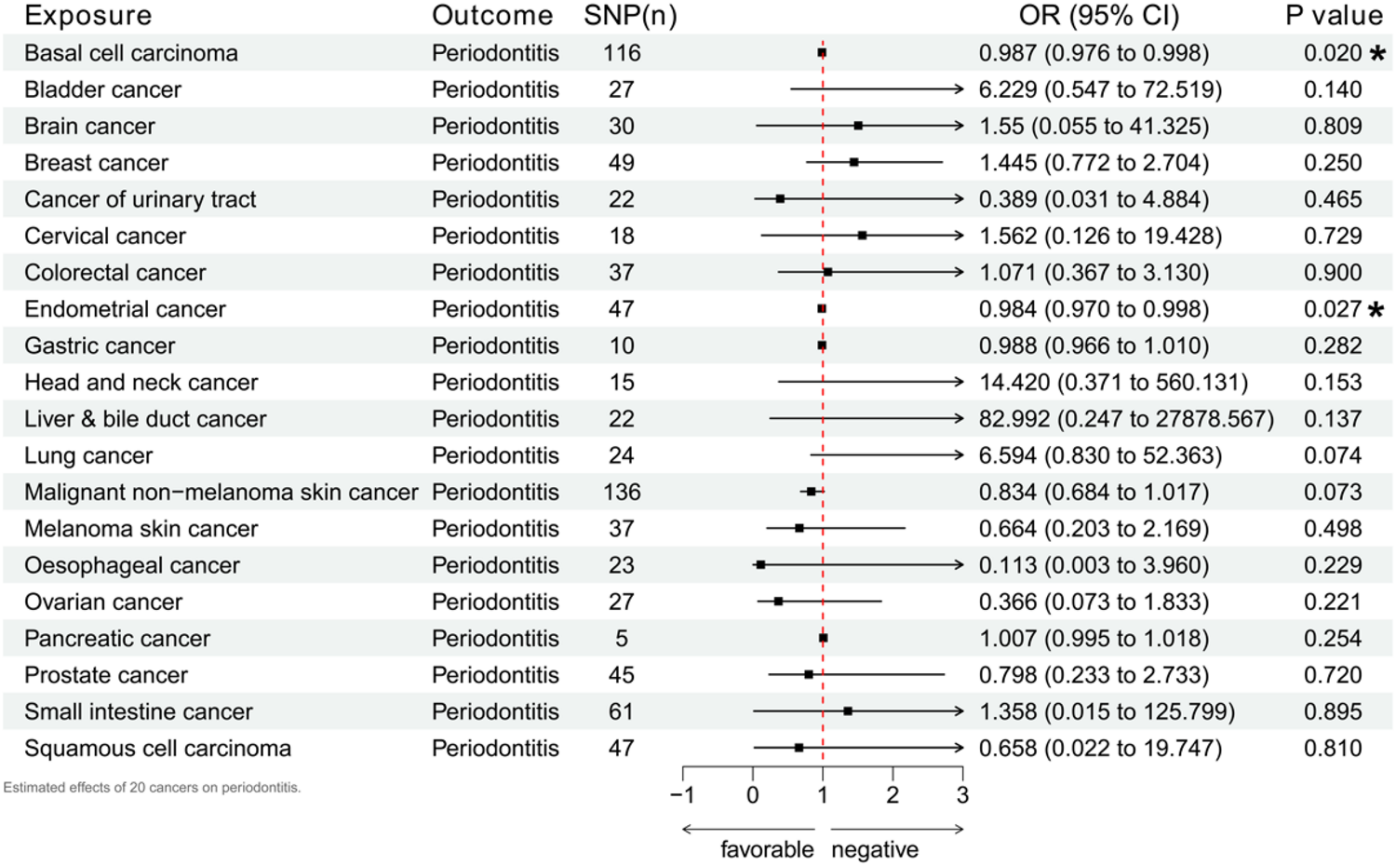
Estimated effects of 20 Common cancers on periodontitis.

The reverse MR analysis is also an integral part of the study. We used 20 common cancers as exposures and matched them with periodontitis for analysis. The detailed results are shown in Supplement S8, while Fig. 7 presents the IVW results. It was found that the occurrence of basal cell carcinoma (p = 0.020, OR 0.987, 95% CI 0.976–0.998) and endometrial cancer (p = 0.027, OR 0.984, 95% CI 0.970–0.998) might increase the risk of periodontitis. There were no objections from other methods' verification and heterogeneity judgment. The other 18 common cancers did not affect the risk of periodontitis.

## Discussion

Cancer continues to be a primary cause of death globally, with the annual increase in new cases suggesting an unfaltering progression. This poses a substantial threat to human lifespan^27^. The significance of early cancer prevention and the potential for effective early-stage interventions cannot be overstated^28^. Our study encompasses 20 commonly diagnosed types of cancer, which constitute over 90% of overall cancer cases globally^29^. Parallelly, periodontitis also holds significant importance in the global public health scenario. The effects of periodontitis on cancer risk have not been unequivocally determined. Our study will enhance our understanding of the periodontitis-cancer relationship, distinguished as the inaugural Mendelian randomization study in this domain.

Our unidirectional Mendelian randomization study suggests periodontitis could be a definitive exposure escalating the risk of head and neck cancer. A refined analysis indicates that exposure to periodontitis exclusively contributes to the incidence of oropharyngeal cancer and confers no particular advantage or risk toward oral cancer, malignant thyroid tumors, and nasopharyngeal cancer. Several published epidemiological studies substantiate the first half of our conclusion—periodontitis possibly increases the risk of head and neck cancer, and there's a positive correlation with the severity of periodontitis. Primarily, three independent meta-analyses serve as the main evidence supporting this view^30–32^. Collectively, they integrate between 8 and 11 non-interventional observational studies, with an overall research population exceeding 10,000 person-times. However, a 2017 cohort study, primarily involving 65,869 American women, contested this view, reporting no substantial association between periodontitis and head and neck cancer comorbidity^33^. The differences possibly stem from the variation in studied groups, most notably in overlooking inflammation and immune factors. Contemporary studies have diversified opinions on our latter conclusion, mainly focusing on oral cancer. While most observational studies support the theory of periodontitis positively impacting oral cancer^14,34,35^, a significant number of control studies share our perspective^36^. Therefore, based on non-interventional observation, it’s challenging to reach a consensus. Our perspective can provide substantial support to the current minority viewpoint.

Few currently published studies have proposed a practical explanation for the relationship between periodontitis and oropharyngeal cancer or periodontitis and head-and-neck cancer. Our research is conducted based on the understanding that periodontitis is a disease that results from a combination of bacteria, inflammation, and immunity. Significant changes in oral microbiome characteristics, inflammatory factors, and immune cell transformations caused by periodontitis have been well-acknowledged and play a vital role as intermediate participants in the onset and progression of some related systemic diseases, such as infective endocarditis^37^, Alzheimer’s disease^38^, arthritis^39^, etc. Our mediation analysis suggests that Fusobacterium nucleatum may be the key intermediary factor in the increase of oropharyngeal cancer risk induced by periodontitis. F. nucleatum, one of the most abundant Gram-negative bacteria in the oral cavity^40^, is an actively engaged species during the period of periodontitis^41^. Referring to F. nucleatum and cancer, most attention is primarily focused on colorectal cancer. Initial studies discovered anomalously excessive traces of F. nucleatum in colon cancer tissue^42^, tracing back to the oral cavity^43^, forming a pathogenic mechanism of colorectal cancer comprised of oral-gut F. nucleatum transfer. This transfer could be through the digestive swallowing^44^ or via a hematogenous transfer^45^. Additionally, F. nucleatum might also be associated with certain glandular cancers, such as pancreatic cancer^46^, esophageal cancer^47^, breast cancer^48^, etc. However, research associating F. nucleatum and oropharyngeal cancer is lacking. Existing research indicates that the main carcinogenic mechanism of F. nucleatum is its predominant proliferation, breaking the original microbial homeostasis and establishing a microenvironment conducive to tumor growth^44^. It can promote cancer cell proliferation and metastasis through two pathways. One is the intracellular-extracellular signaling pathway mediated by its surface adhesin FadA^49^, while the other is by upregulating microRNA21 (miR21)^50^. Additionally, it can inhibit the recruitment and activity of tumor-infiltrating T cells, reducing immune damage to cancer tissues^51^. The exploration of more molecular mechanisms is still in progress.

Our study utilizes reverse Mendelian randomization analysis to propose a potential acceleration of periodontitis due to the presence of basal cell carcinoma and endometrial cancer. These conditions might possibly represent an unusual form of periodontitis, which could serve as a forewarning or manifestation of basal cell carcinoma and endometrial cancer. To date, no direct reports have clarified the impact of basal cell carcinoma and endometrial cancer on predisposition to periodontitis, thus our discussion mainly presents conjectures of potential future developments. Intercommunication along the skin-oral microbiome axis may serve as a basal mechanism that could result in periodontitis induced by basal cell carcinoma^52^. Irregularities in skin microbial signals could possibly disrupt the oral microbiome equilibrium, eventually leading to periodontitis, but this notion requires further study. The link connecting endometrial cancer and periodontitis might revolve around hormonal factors. A specific hormonal environment could provide conditions conducive to the growth and progression of periodontitis^53^. Without question, endometrial cancer is a potent disruptor of hormonal balance in women of reproductive age^54^. These theories underpin our conjectures. Despite the speculative nature of these associations, we hope that future researchers will explore and consider these theories with a keen interest.

Our Mendelian study is pioneering within its field as a quasi-randomized controlled trial delivering our perspective towards the persisting periodontitis-cancer dilemma. Our study proposes that periodontitis accelerates the risk of oropharyngeal cancer but doesn’t significantly influence the other 19 common cancers and three subclasses of head and neck cancers. However, our study is not without limitations. Our GWAS datasets largely consist of European participants, and different races and populations may have different genetic risks, thus lacking global representativeness. Meanwhile, factors such as lifestyle habits, dietary habits, environmental exposures, and other unconsidered biological mechanisms might interact with genetic factors, affecting phenotypes and potentially offering alternative explanations to our findings. This complex interaction may affect our results and must be considered in further research. Furthermore, our intermediary factor analysis only focused on a subset of oral microbes, inflammatory factors, and immune cells, making the study less comprehensive. Moreover, our proposal based on the study's findings anticipates more research in the future.

### Supplementary Information

Supplementary Information.

## Data Availability

Data supporting the findings of this study are available within the article and its supplementary information files. Publicly available datasets were analyzed during this study and can be found at the gene-lifestyle interactions in dental endpoints (GLIDE) consortium database (https://data.bris.ac.uk/data/dataset/2j2rqgzedxlq02oqbb4vmycnc2), the OpenGWAS database (https://gwas.mrcieu.ac.uk/), the Chinese human genetic resources administration and gene organ review committee database (https://db.cngb.org/search/project/CNP0001664), and at the data repository hosted by Bristol University (https://data.bris.ac.uk/data/dataset/3g3i5smgghp0s2uvm1doflkx9x). The data that support the findings of this study are available on request from the corresponding author, Jiali Feng. The data are not publicly available due to privacy or ethical restrictions.
