# Supplementary material for: Linking periodontitis with 20 cancers, emphasis on oropharyngeal cancer: a Mendelian randomization analysis
Source: Sci Rep. 2024 May 31;14:12511. doi: 10.1038/s41598-024-63447-4 (PMC11143368; doi:10.1038/s41598-024-63447-4)
Supplement: Supplementary file 1 — Supplementary Information. [file 41598_2024_63447_MOESM1_ESM.docx]

**Linking Periodontitis with 20 Cancers, Emphasis on oropharyngeal cancer: A Mendelian Randomization Analysis**

**Jun Xiong^1^, Hao Liu^2, 3, 4^, Conghua Li^2, 3, 4^, Yong Li^2, 3, 4^, Jiali Feng^2, 3, 4^**

**S1.** Overview of the genetic data

**S2.** Detailed information on instrumental variables used in Mendelian randomization analysis

**S3.** Mendelian randomization analysis of periodontitis and 20 cancers.

**S4.** Mendelian randomization analysis of periodontitis and 4 subclasses of head and neck cancer

**S5.**  Mendelian randomization analysis of 20 oral microorganisms and oropharyngeal cancer

**S6.** Mendelian randomization analysis of 9 immune cells and oropharyngeal cancer

**S7.** Mendelian randomization analysis of 14 inflammatory factors and oropharyngeal cancer

**S8.** Mendelian randomization analysis of 20 cancers and periodontitis.

**S1.** Overview of the genetic data

- 1. Sources of genetic data for periodontitis

Genetic data on periodontitis were obtained from the latest GWAS meta-analysis from the Gene-Lifestyle Interactions in Dental Endpoints (GLIDE) Consortium, a pooled data set containing the largest sample to date, involving 17,353 clinically diagnosed cases and 28,210 controls[1]. Periodontitis cases are classified according to the Centers for Disease Control and Prevention/American Academy of Periodontology Case Definition (CDC/AAP) or the Community Periodontal Index (CPI). Access to the data can be obtained by downloading from the link provided in the original article (https://data.bris.ac.uk/data/dataset/2j2rqgzedxlq02oqbb4vmycnc2).

- 1. Sources of genetic data for 20 cancers

GWAS data for 20 cancers were obtained from the public GWAS database OpenGWAS (https://gwas.mrcieu.ac.uk/). **Table 1** shows the data source numbers and characteristics.

**Table 1 Overview of the genetic data for 20 cancers**

| **GWAS ID** | **Year** | **Trait** | **Consortium** | **Sample size** | **Number of SNPs** |
| --- | --- | --- | --- | --- | --- |
| ieu-b-4875 | 2021 | Brain cancer | NA | 372,622 | 8,629,116 |
| ieu-b-4912 | 2021 | Head and neck cancer | UK Biobank | 373,122 | 9,655,080 |
| ieu-b-4960 | 2021 | Oesophageal cancer | UK Biobank | 372,756 | 8,970,465 |
| bbj-a-119 | 2019 | Gastric cancer | NA | 202,308 | 8,885,324 |
| ukb-a-56 | 2017 | Small intestine cancer | Neale Lab | 337,159 | 10,894,596 |
| ieu-b-4965 | 2021 | Colorectal cancer | UK Biobank | 377,673 | 11,738,639 |
| ieu-b-4954 | 2021 | Lung cancer | UK Biobank | 374,687 | 11,078,115 |
| ieu-b-4915 | 2021 | Liver & bile duct cancer | UK Biobank | 372,366 | 7,687,713 |
| bbj-a-140 | 2019 | Pancreatic cancer | NA | 196,187 | 8,885,075 |
| ukb-d-C_URINARY_TRACT | 2018 | Cancer of urinary tract | NA | 361,194 | 10,309,627 |
| ieu-b-4874 | 2021 | Bladder cancer | NA | 373,295 | 9,904,926 |
| ukb-a-57 | 2017 | Prostate cancer | Neale Lab | 337,159 | 10,894,596 |
| ukb-a-55 | 2017 | Breast cancer | Neale Lab | 337,159 | 10,894,596 |
| ieu-b-4963 | 2021 | Ovarian cancer | UK Biobank | 199,741 | 9,822,229 |
| ieu-b-4876 | 2021 | Cervical cancer | NA | 199,086 | 8,506,261 |
| ebi-a-GCST006464 | 2018 | Endometrial cancer | NA | 121,885 | 9,470,555 |
| ieu-b-4959 | 2021 | Malignant non-melanoma skin cancer | UK Biobank | 395,710 | 12,321,875 |
| ieu-b-4969 | 2021 | Melanoma skin cancer | UK Biobank | 375,767 | 11,396,019 |

1.3 Sources of genetic data for 4 head and neck cancers

GWAS data for the 4 subclasses of head and neck cancer were also obtained from OpenGWAS (<https://gwas.mrcieu.ac.uk/>). **Table 2** shows the data source numbers and characteristics.

**Table 2 Overview of the genetic data for 4 head and neck cancers**

| **GWAS ID** | **Year** | **Trait** | **Consortium** | **Sample size** | **Number of SNPs** |
| --- | --- | --- | --- | --- | --- |
| ieu-b-4961 | 2021 | Oral cavity cancer | UK Biobank | 372,373 | 7,723,107 |
| ieu-b-4968 | 2021 | Oropharyngeal cancer | UK Biobank | 372,510 | 8,283,869 |
| ieu-b-4913 | 2021 | Laryngeal cancer | UK Biobank | 372,289 | 7,239,512 |
| finn-b-C3_THYROID_GLAND | 2021 | Malignant neoplasm of thyroid gland | NA | — | 16,380,466 |

1.4 Sources of genetic data for 20 oral microorganisms

Genetic data for 20 oral microorganisms were obtained from the same source as the original study by Xiaomin Liu et al[2]. involving 2017 dorsal tongue samples and 1915 saliva samples. According to the Human Genetic Resources Administration of China regulation and the institutional review board of BGI-Shenzhen related to protecting individual privacy, we obtained an access license for this restricted data from the data repository (https://db.cngb.org/search/project/CNP0001664). We are not allowed to provide this raw data.

1.5 Sources of genetic data for 9 immune cells

GWAS data for 9 immune cells were obtained from OpenGWAS (<https://gwas.mrcieu.ac.uk/>). **Table 3** shows the data source numbers and characteristics.

**Table 3 Overview of the genetic data for 9 immune cells**

| **GWAS ID** | **Year** | **Trait** | **Consortium** | **Sample size** | **Number of SNPs** |
| --- | --- | --- | --- | --- | --- |
| ebi-a-GCST90001642 | 2020 | B cell Absolute Count | NA | 3,653 | 15,195,758 |
| ebi-a-GCST90001407 | 2020 | Memory B cell Absolute Count | NA | 3,656 | 15,048,937 |
| ebi-a-GCST90001603 | 2020 | T cell Absolute Count | NA | 3,653 | 15,195,758 |
| ebi-a-GCST90001480 | 2020 | Resting CD4 regulatory T cell Absolute Count | NA | 3,405 | 15,131,843 |
| ebi-a-GCST90001592 | 2020 | CD8+ T cell Absolute Count | NA | 3,652 | 15,195,743 |
| ebi-a-GCST90001405 | 2020 | Plasma Blast-Plasma Cell Absolute Count | NA | 3,657 | 15,049,159 |
| ebi-a-GCST90001409 | 2020 | Naive-mature B cell Absolute Count | NA | 3,656 | 15,048,937 |
| ebi-a-GCST90001486 | 2020 | Activated CD4 regulatory T cell Absolute Count | NA | 3,405 | 15,131,843 |
| ieu-b-34 | 2020 | neutrophil cell count | Blood Cell Consortium | 563,946 | — |

1.6 Sources of genetic data for 14 inflammatory factors

GWAS data for 14 inflammatory factors were obtained from publicly published and available data from Ahola-Olli. et al[3]. The study included 8,293 participants. We downloaded the data from the provided public site (https://data.bris.ac.uk/data/dataset/3g3i5smgghp0s2uvm1doflkx9x).

**References:**

1. Shungin D, Haworth S, Divaris K, Agler CS, Kamatani Y, Keun LM, Grinde K, Hindy G, Alaraudanjoki V, Pesonen P *et al*: **Genome-wide analysis of dental caries and periodontitis combining clinical and self-reported data**. *NAT COMMUN* 2019, **10**(1):2773.

2. Liu X, Tong X, Zhu J, Tian L, Jie Z, Zou Y, Lin X, Liang H, Li W, Ju Y *et al*: **Metagenome-genome-wide association studies reveal human genetic impact on the oral microbiome**. *CELL DISCOV* 2021, **7**(1):117.

3. Ahola-Olli AV, Würtz P, Havulinna AS, Aalto K, Pitkänen N, Lehtimäki T, Kähönen M, Lyytikäinen LP, Raitoharju E, Seppälä I *et al*: **Genome-wide Association Study Identifies 27 Loci Influencing Concentrations of Circulating Cytokines and Growth Factors**. *AM J HUM GENET* 2017, **100**(1):40-50.

**S2.** Detailed information on instrumental variables used in Mendelian randomization analysis

(SNP: single nucleotide polymorphism; Chr: chromosome; Pos: position according to GRCh37/hg19 genome assembly; EA: effect allele; OA: other allele; EAF: effect allele frequency; SE: standard error of beta.)

Table 1 Instrumental variables of periodontitis

| SNP | Chr | Pos | EA | OA | EAF | Beta | SE | P |
| --- | --- | --- | --- | --- | --- | --- | --- | --- |
| rs10757466 | 9 | 24000258 | T | C | 0.14 | -0.117 | 0.026 | 9.26E-06 |
| rs118009719 | 8 | 86928036 | A | C | 0.04 | -0.275 | 0.062 | 9.24E-06 |
| rs13005050 | 2 | 52705571 | T | C | 0.88 | -0.143 | 0.031 | 3.76E-06 |
| rs148287804 | 2 | 43560590 | T | G | 0.98 | -0.383 | 0.085 | 6.22E-06 |
| rs151226594 | 11 | 64256137 | T | G | 0.97 | -0.367 | 0.077 | 1.75E-06 |
| rs184267209 | 12 | 94396361 | A | G | 0.02 | 0.246 | 0.055 | 8.91E-06 |
| rs186040223 | 7 | 64014696 | A | C | 0.85 | -0.158 | 0.035 | 5.66E-06 |
| rs1901299 | 18 | 22442179 | A | C | 0.07 | -0.09 | 0.02 | 8.63E-06 |
| rs190792824 | 20 | 42706632 | T | G | 0.04 | -0.301 | 0.066 | 5.75E-06 |
| rs28546695 | 18 | 49760987 | A | G | 0.38 | 0.083 | 0.019 | 8.21E-06 |
| rs2976950 | 8 | 8249082 | A | G | 0.6 | 0.096 | 0.02 | 7.99E-07 |
| rs4640758 | 5 | 7613236 | A | G | 0.36 | -0.085 | 0.019 | 7.28E-06 |
| rs4956201 | 4 | 109527782 | A | C | 0.06 | -0.241 | 0.047 | 3.89E-07 |
| rs4969455* | 17 | 78785746 | T | C | 0.06 | -0.183 | 0.041 | 7.83E-06 |
| rs6816769 | 4 | 122216017 | T | C | 0.08 | -0.135 | 0.029 | 4.57E-06 |
| rs73155039# | 7 | 136207654 | A | G | 0.99 | 0.832 | 0.176 | 2.22E-06 |
| rs78422482 | 4 | 19970150 | A | G | 0.02 | 0.243 | 0.051 | 2.02E-06 |
| rs78982133 | 6 | 11688132 | A | G | 0.97 | -0.356 | 0.08 | 8.71E-06 |

Table 2 Instrumental variables of Actinomyces oris

| SNP | EA | OA | Beat | SE | P |
| --- | --- | --- | --- | --- | --- |
| rs10127711 | A | G | -0.1054 | 0.0225 | 2.99E-06 |
| rs7627316 | A | C | -0.1018 | 0.0225 | 6.50E-06 |
| rs2282171 | C | T | 0.1043 | 0.02253 | 3.96E-06 |
| rs61794897 | T | C | 0.1204 | 0.02243 | 8.89E-08 |
| rs7731715 | A | G | -0.1014 | 0.02244 | 6.63E-06 |
| rs72790034 | A | G | -0.1077 | 0.0225 | 1.83E-06 |
| rs11749001 | C | T | -0.1 | 0.02246 | 8.87E-06 |
| rs4298358 | G | C | -0.1039 | 0.02248 | 4.09E-06 |
| rs17847551 | T | C | -0.1021 | 0.0225 | 6.03E-06 |
| rs9523056 | T | C | -0.1115 | 0.02246 | 7.50E-07 |
| rs10135427 | G | T | -0.1051 | 0.0225 | 3.20E-06 |
| rs896668 | G | A | -0.1006 | 0.02249 | 8.15E-06 |
| rs4993369 | T | G | 0.1002 | 0.02255 | 9.29E-06 |
| rs691797 | C | G | 0.1035 | 0.0225 | 4.53E-06 |
| rs8096890 | G | T | 0.1012 | 0.0225 | 7.32E-06 |

Table 3 Instrumental variables of Capnocytophaga granulosa

| SNP | EA | OA | Beat | SE | P |
| --- | --- | --- | --- | --- | --- |
| rs141517526 | T | G | 0.1024 | 0.02263 | 6.46E-06 |
| rs12632659 | T | A | -0.1006 | 0.02266 | 9.45E-06 |
| rs2400263 | G | A | -0.1166 | 0.02265 | 2.96E-07 |
| rs1935864 | G | A | 0.1075 | 0.02268 | 2.29E-06 |
| rs11830426 | C | T | -0.106 | 0.02264 | 3.06E-06 |
| rs9523379 | C | T | 0.1031 | 0.02271 | 5.95E-06 |
| rs6492905 | C | A | 0.1072 | 0.02269 | 2.45E-06 |
| rs2270412 | G | A | -0.1024 | 0.02266 | 6.58E-06 |
| rs2190494 | A | G | 0.1014 | 0.02265 | 8.04E-06 |

Table 4 Instrumental variables of Capnocytophaga leadbetteri

| SNP | EA | OA | Beat | SE | P |
| --- | --- | --- | --- | --- | --- |
| rs12752324 | A | G | -0.102 | 0.0226 | 6.81E-06 |
| rs6555118 | C | G | 0.1035 | 0.02258 | 4.89E-06 |
| rs7019960 | G | A | 0.1029 | 0.02265 | 5.90E-06 |
| rs72847864 | T | A | -0.1049 | 0.02274 | 4.22E-06 |
| rs61877162 | C | T | 0.1006 | 0.02255 | 8.60E-06 |
| rs77451023 | T | A | 0.1101 | 0.02263 | 1.25E-06 |
| rs12910901 | C | A | -0.1046 | 0.02252 | 3.62E-06 |
| rs240761 | C | G | -0.1046 | 0.02263 | 4.05E-06 |

Table 5 Instrumental variables of Capnocytophaga sputigena

| SNP | EA | OA | Beat | SE | P |
| --- | --- | --- | --- | --- | --- |
| rs2274531 | C | T | 0.1045 | 0.02258 | 3.92E-06 |
| rs62251091 | C | T | -0.1031 | 0.02267 | 5.71E-06 |
| rs4687982 | G | T | 0.122 | 0.02254 | 7.07E-08 |
| rs9833256 | T | C | -0.1105 | 0.02263 | 1.13E-06 |
| rs2310137 | T | C | 0.1055 | 0.02255 | 3.14E-06 |
| rs6881079 | A | G | 0.1039 | 0.02261 | 4.62E-06 |
| rs6933801 | C | T | 0.1023 | 0.02267 | 6.86E-06 |
| rs11139009 | C | A | -0.1022 | 0.02261 | 6.60E-06 |
| rs498603 | C | T | 0.1019 | 0.02258 | 6.84E-06 |
| rs4940989 | G | A | 0.1035 | 0.02263 | 5.12E-06 |
| rs2080831 | T | G | -0.1044 | 0.02257 | 3.97E-06 |
| rs73230320 | G | C | -0.1013 | 0.0227 | 8.64E-06 |

Table 6 Instrumental variables of Corynebacterium durum

| SNP | EA | OA | Beat | SE | P |
| --- | --- | --- | --- | --- | --- |
| rs17098431 | T | C | -0.1077 | 0.02246 | 1.76E-06 |
| rs11571091 | A | G | 0.1073 | 0.02259 | 2.19E-06 |
| rs9973657 | C | A | -0.1033 | 0.02256 | 4.98E-06 |
| rs2103355 | G | T | -0.1061 | 0.02255 | 2.73E-06 |
| rs34720860 | A | G | 0.1016 | 0.02266 | 7.75E-06 |
| rs79596360 | A | T | 0.1021 | 0.02266 | 7.05E-06 |
| rs7727095 | A | G | 0.1009 | 0.02254 | 8.07E-06 |
| rs12678495 | C | G | 0.1039 | 0.02254 | 4.33E-06 |
| rs13258367 | C | A | 0.1192 | 0.02246 | 1.25E-07 |
| rs60831859 | C | T | -0.1089 | 0.02251 | 1.43E-06 |
| rs1322471 | A | C | -0.1205 | 0.02245 | 8.98E-08 |
| rs12000829 | T | C | -0.1006 | 0.02252 | 8.45E-06 |
| rs11219510 | C | G | 0.1002 | 0.02245 | 8.53E-06 |
| rs1241511 | C | A | 0.1013 | 0.02249 | 6.98E-06 |
| rs2012112 | C | T | 0.1055 | 0.02247 | 2.87E-06 |
| rs2092046 | C | T | 0.1091 | 0.02251 | 1.35E-06 |

Table 7 Instrumental variables of Gemella haemolysans

| SNP | EA | OA | Beat | SE | P |
| --- | --- | --- | --- | --- | --- |
| rs3011969 | A | G | -0.09701 | 0.02161 | 7.60E-06 |
| rs445213 | G | C | 0.1014 | 0.02164 | 2.97E-06 |
| rs4522770 | A | G | 0.09937 | 0.02164 | 4.66E-06 |
| rs1355760 | G | A | -0.1016 | 0.02157 | 2.65E-06 |
| rs324676 | A | G | 0.09795 | 0.02163 | 6.30E-06 |
| rs2274647 | T | G | -0.1048 | 0.0217 | 1.47E-06 |
| rs2942349 | C | T | 0.09777 | 0.02159 | 6.29E-06 |
| rs4551677 | G | T | 0.1046 | 0.0217 | 1.54E-06 |
| rs11638639 | C | T | 0.09851 | 0.02169 | 5.92E-06 |
| rs12442073 | G | A | -0.1263 | 0.0216 | 5.89E-09 |
| rs7196133 | G | A | -0.0993 | 0.02157 | 4.41E-06 |
| rs62033254 | T | C | 0.09873 | 0.02163 | 5.33E-06 |
| rs2003811 | T | C | 0.09578 | 0.02159 | 9.64E-06 |

Table 8 Instrumental variables of Haemophilus parainfluenzae

| SNP | EA | OA | Beat | SE | P |
| --- | --- | --- | --- | --- | --- |
| rs11802596 | C | T | 0.1012 | 0.02253 | 7.51E-06 |
| rs9874483 | C | G | -0.1121 | 0.02264 | 7.98E-07 |
| rs7624703 | A | C | 0.111 | 0.02254 | 9.15E-07 |
| rs9869515 | C | G | -0.1079 | 0.02287 | 2.56E-06 |
| rs4613624 | G | A | 0.1121 | 0.02255 | 7.19E-07 |
| rs1486756 | A | G | -0.1088 | 0.02256 | 1.53E-06 |
| rs11598219 | G | A | 0.1026 | 0.02258 | 5.91E-06 |
| rs12314926 | G | T | 0.1067 | 0.02257 | 2.42E-06 |
| rs34748 | G | A | -0.1135 | 0.0226 | 5.52E-07 |
| rs116159234 | C | T | -0.102 | 0.02256 | 6.50E-06 |
| rs10139701 | C | G | -0.1114 | 0.02254 | 8.42E-07 |
| rs1376075 | T | C | -0.1028 | 0.02261 | 5.79E-06 |
| rs139794 | C | T | 0.1079 | 0.02259 | 1.93E-06 |

Table 9 Instrumental variables of Kingella

| SNP | EA | OA | Beat | SE | P |
| --- | --- | --- | --- | --- | --- |
| rs11122108 | G | A | 0.1164 | 0.02279 | 3.59E-07 |
| rs997029 | C | T | -0.1131 | 0.02274 | 7.19E-07 |
| rs72694491 | A | G | 0.1033 | 0.02282 | 6.41E-06 |
| rs11765142 | A | G | 0.1107 | 0.02284 | 1.37E-06 |
| rs6464386 | C | T | 0.1029 | 0.0228 | 6.73E-06 |
| rs1960416 | C | T | -0.1033 | 0.02276 | 6.04E-06 |
| rs3843622 | A | T | -0.1057 | 0.02281 | 3.80E-06 |
| rs9551920 | T | A | 0.1018 | 0.02277 | 8.21E-06 |
| rs8043987 | C | A | 0.101 | 0.02279 | 9.90E-06 |
| rs78126055 | T | A | -0.1013 | 0.02276 | 9.07E-06 |
| rs116978045 | T | C | 0.1112 | 0.02277 | 1.12E-06 |
| rs743802 | G | A | -0.1049 | 0.02289 | 4.84E-06 |

Table 10 Instrumental variables of Neisseria flava

| SNP | EA | OA | Beat | SE | P |
| --- | --- | --- | --- | --- | --- |
| rs116345289 | G | A | 0.1075 | 0.02262 | 2.16E-06 |
| rs76877294 | T | A | 0.1115 | 0.0227 | 9.87E-07 |
| rs13237679 | G | A | 0.1044 | 0.02269 | 4.46E-06 |
| rs9986975 | G | A | -0.1076 | 0.02266 | 2.21E-06 |
| rs12700692 | C | T | 0.1054 | 0.02271 | 3.68E-06 |
| rs17617603 | T | C | 0.1037 | 0.0227 | 5.27E-06 |
| rs11765949 | C | T | 0.1023 | 0.02267 | 6.87E-06 |
| rs10509630 | G | A | -0.1054 | 0.02265 | 3.51E-06 |
| rs2104641 | A | G | 0.12 | 0.02265 | 1.30E-07 |
| rs73243848 | T | C | 0.1189 | 0.0227 | 1.78E-07 |
| rs2328222 | C | T | -0.1021 | 0.02269 | 7.22E-06 |

Table 11 Instrumental variables of Rothia aeria

| SNP | EA | OA | Beat | SE | P |
| --- | --- | --- | --- | --- | --- |
| rs775792 | C | G | -0.1012 | 0.02248 | 7.21E-06 |
| rs56029204 | A | G | -0.1012 | 0.02253 | 7.51E-06 |
| rs9866285 | C | G | -0.1012 | 0.02249 | 7.30E-06 |
| rs11723070 | G | A | 0.1014 | 0.02256 | 7.41E-06 |
| rs138667209 | A | C | -0.1032 | 0.02258 | 5.19E-06 |
| rs6909879 | A | G | -0.1052 | 0.02249 | 3.13E-06 |
| rs2193184 | C | T | -0.1036 | 0.02265 | 5.09E-06 |
| rs4769296 | C | A | -0.1025 | 0.02256 | 5.85E-06 |
| rs2172724 | A | G | -0.1015 | 0.02253 | 7.02E-06 |
| rs59057498 | T | A | -0.1127 | 0.02268 | 7.42E-07 |
| rs2092046 | C | T | 0.1185 | 0.02251 | 1.55E-07 |

Table 12 Instrumental variables of Streptococcus sanguinis

| SNP | EA | OA | Beat | SE | P |
| --- | --- | --- | --- | --- | --- |
| rs6683394 | G | A | 0.1109 | 0.02261 | 1.01E-06 |
| rs2773155 | G | T | -0.1043 | 0.0226 | 4.20E-06 |
| rs16832579 | G | C | -0.1093 | 0.02261 | 1.46E-06 |
| rs7707327 | A | G | -0.1006 | 0.02261 | 9.11E-06 |
| rs7731715 | A | G | -0.1027 | 0.02256 | 5.67E-06 |
| rs11968648 | T | C | -0.1012 | 0.02268 | 8.59E-06 |
| rs58654082 | G | A | -0.1135 | 0.02266 | 5.99E-07 |
| rs1558737 | G | A | -0.105 | 0.02263 | 3.70E-06 |
| rs6023331 | T | C | -0.1034 | 0.02261 | 5.10E-06 |

Table 13 Instrumental variables of Veillonella parvula

| SNP | EA | OA | Beat | SE | P |
| --- | --- | --- | --- | --- | --- |
| rs171870 | A | G | -0.1021 | 0.02276 | 7.65E-06 |
| rs72799650 | T | G | 0.101 | 0.02269 | 8.91E-06 |
| rs13189100 | C | G | 0.1043 | 0.02275 | 4.86E-06 |
| rs79601514 | C | T | -0.1162 | 0.02261 | 3.01E-07 |
| rs17643535 | G | C | 0.1013 | 0.02262 | 7.90E-06 |
| rs13261208 | G | A | -0.1025 | 0.02265 | 6.47E-06 |
| rs7857179 | C | T | -0.1014 | 0.02268 | 8.29E-06 |
| rs73537311 | C | T | -0.1012 | 0.02272 | 8.98E-06 |
| rs1325341 | T | A | 0.108 | 0.02267 | 2.07E-06 |
| rs62004643 | G | A | -0.1037 | 0.0229 | 6.30E-06 |
| rs7184601 | C | T | -0.1013 | 0.02269 | 8.55E-06 |

Table 14 Instrumental variables of Alloprevotella tannerae

| SNP | EA | OA | Beat | SE | P |
| --- | --- | --- | --- | --- | --- |
| rs13099664 | A | C | -0.1071 | 0.02332 | 4.73E-06 |
| rs17068219 | T | C | 0.1046 | 0.02269 | 4.32E-06 |
| rs71543361 | C | T | 0.1004 | 0.02263 | 9.64E-06 |
| rs17065552 | C | T | -0.1087 | 0.02256 | 1.58E-06 |
| rs11137735 | G | A | 0.101 | 0.02256 | 8.00E-06 |
| rs190840231 | C | A | 0.1041 | 0.02264 | 4.58E-06 |
| rs79735079 | C | A | 0.1095 | 0.02259 | 1.35E-06 |
| rs7998390 | G | A | 0.1102 | 0.02262 | 1.19E-06 |
| rs73979193 | G | C | 0.106 | 0.02257 | 2.83E-06 |

Table 15 Instrumental variables of Filifactor alocis

| SNP | EA | OA | Beat | SE | P |
| --- | --- | --- | --- | --- | --- |
| rs1321613 | G | A | 0.1024 | 0.02278 | 7.44E-06 |
| rs10950914 | C | A | -0.1005 | 0.02255 | 8.78E-06 |
| rs7851633 | T | G | -0.1025 | 0.02263 | 6.29E-06 |
| rs11197377 | C | T | -0.1088 | 0.02254 | 1.49E-06 |
| rs1506237 | A | T | 0.101 | 0.02262 | 8.51E-06 |

Table 16 Instrumental variables of Fusobacterium nucleatum

| SNP | EA | OA | Beat | SE | P |
| --- | --- | --- | --- | --- | --- |
| rs16839469 | G | A | -0.104 | 0.02278 | 5.28E-06 |
| rs17038192 | A | C | -0.1062 | 0.02278 | 3.33E-06 |
| rs73814865 | C | T | -0.1133 | 0.02276 | 6.99E-07 |
| rs6596855 | G | A | -0.1078 | 0.0227 | 2.19E-06 |
| rs9386276 | G | A | -0.1021 | 0.02267 | 7.05E-06 |
| rs10740590 | T | C | 0.1027 | 0.02268 | 6.33E-06 |
| rs68120139 | T | C | 0.1038 | 0.02273 | 5.25E-06 |
| rs139605585 | G | A | 0.1021 | 0.02272 | 7.51E-06 |
| rs62232234 | C | T | -0.1015 | 0.02278 | 8.82E-06 |

Table 17 Instrumental variables of Parvimonas

| SNP | EA | OA | Beat | SE | P |
| --- | --- | --- | --- | --- | --- |
| rs4590 | C | G | -0.1122 | 0.02241 | 5.99E-07 |
| rs17731631 | C | T | -0.1014 | 0.02238 | 6.27E-06 |
| rs78792578 | A | G | 0.1083 | 0.02234 | 1.36E-06 |
| rs17029269 | C | T | -0.1038 | 0.02236 | 3.72E-06 |
| rs34739023 | G | T | 0.1053 | 0.02241 | 2.81E-06 |
| rs1148114 | G | T | 0.1051 | 0.02239 | 2.89E-06 |
| rs1624953 | A | G | 0.09934 | 0.0224 | 9.78E-06 |
| rs79374875 | A | G | 0.1002 | 0.02237 | 7.85E-06 |
| rs6069771 | A | G | -0.1005 | 0.02243 | 7.87E-06 |

Table 18 Instrumental variables of Porphyromonas gingivalis

| SNP | EA | OA | Beat | SE | P |
| --- | --- | --- | --- | --- | --- |
| rs34513054 | C | G | 0.0986 | 0.02223 | 9.73E-06 |
| rs11685666 | C | T | -0.09987 | 0.02202 | 6.13E-06 |
| rs59225308 | A | T | 0.102 | 0.02214 | 4.36E-06 |
| rs144628581 | T | C | 0.1003 | 0.02202 | 5.51E-06 |
| rs3828581 | C | T | -0.1028 | 0.02217 | 3.76E-06 |
| rs78918882 | T | C | 0.109 | 0.02206 | 8.41E-07 |
| rs7458816 | A | C | 0.1029 | 0.02201 | 3.11E-06 |
| rs944069 | T | C | 0.1005 | 0.0221 | 5.72E-06 |
| rs7087496 | T | G | 0.09983 | 0.02214 | 6.89E-06 |
| rs9668910 | T | G | -0.1062 | 0.022 | 1.49E-06 |
| rs72747726 | A | G | -0.1017 | 0.02203 | 4.12E-06 |
| rs7224533 | T | C | 0.1016 | 0.02207 | 4.46E-06 |
| rs6113922 | C | A | 0.09982 | 0.02206 | 6.43E-06 |

Table 19 Instrumental variables of Prevotella intermedia

| SNP | EA | OA | Beat | SE | P |
| --- | --- | --- | --- | --- | --- |
| rs2043867 | G | A | 0.1072 | 0.02255 | 2.17E-06 |
| rs1860700 | G | A | -0.1033 | 0.02252 | 4.75E-06 |
| rs9320504 | G | C | -0.09984 | 0.02251 | 9.70E-06 |
| rs10275995 | A | G | -0.1035 | 0.02273 | 5.67E-06 |
| rs62514773 | A | G | -0.1075 | 0.02255 | 2.01E-06 |
| rs10969086 | G | A | 0.1128 | 0.02248 | 5.73E-07 |
| rs11142034 | C | T | -0.1039 | 0.02256 | 4.35E-06 |
| rs11260001 | A | G | 0.1008 | 0.02251 | 7.97E-06 |
| rs522194 | T | C | -0.1083 | 0.02262 | 1.83E-06 |
| rs136204 | A | G | 0.1035 | 0.02255 | 4.75E-06 |

Table 20 Instrumental variables of Tannerella

| SNP | EA | OA | Beat | SE | P |
| --- | --- | --- | --- | --- | --- |
| rs6744211 | A | G | 0.1035 | 0.02246 | 4.30E-06 |
| rs6761850 | C | G | -0.112 | 0.02242 | 6.42E-07 |
| rs6822701 | A | G | -0.1154 | 0.0225 | 3.23E-07 |
| rs10828263 | G | A | 0.1013 | 0.02252 | 7.31E-06 |
| rs66495310 | G | A | 0.09963 | 0.02247 | 9.82E-06 |
| rs17410829 | C | T | -0.1077 | 0.0225 | 1.85E-06 |
| rs10891326 | G | A | -0.1215 | 0.02248 | 7.37E-08 |
| rs12230193 | C | T | 0.09985 | 0.0225 | 9.58E-06 |
| rs3952319 | A | T | -0.1028 | 0.02254 | 5.43E-06 |
| rs2716556 | T | G | -0.1011 | 0.02247 | 7.30E-06 |

Table 21 Instrumental variables of Treponema denticola

| SNP | EA | OA | Beat | SE | P |
| --- | --- | --- | --- | --- | --- |
| rs1411017 | T | C | -0.1132 | 0.02286 | 8.06E-07 |
| rs7534415 | G | A | 0.1076 | 0.02275 | 2.40E-06 |
| rs2349918 | G | A | -0.1067 | 0.02281 | 3.10E-06 |
| rs77748708 | A | G | 0.1028 | 0.0229 | 7.55E-06 |
| rs58141521 | A | G | 0.1061 | 0.02289 | 3.84E-06 |
| rs963232 | T | C | -0.1098 | 0.02279 | 1.56E-06 |
| rs28520260 | A | G | -0.118 | 0.02271 | 2.25E-07 |
| rs9298138 | G | C | 0.1096 | 0.02278 | 1.63E-06 |
| rs11142034 | C | T | -0.109 | 0.02282 | 1.95E-06 |
| rs141203896 | G | A | 0.1015 | 0.02279 | 9.03E-06 |
| rs9668910 | T | G | -0.1326 | 0.02267 | 5.79E-09 |
| rs77082358 | T | C | 0.1021 | 0.02278 | 7.83E-06 |
| rs7209470 | G | A | -0.1016 | 0.02278 | 8.63E-06 |
| rs112597027 | T | C | 0.1026 | 0.02281 | 7.26E-06 |

Table 22 Instrumental variables of B cell

| SNP | Chr | Pos | EA | OA | EAF | Beta | SE | P |
| --- | --- | --- | --- | --- | --- | --- | --- | --- |
| rs490416 | 1 | 20838219 | T | A | 0.5371 | 0.1093 | 0.02432 | 7.16E-06 |
| rs17113368 | 1 | 95786229 | T | C | 0.0965 | 0.1875 | 0.04172 | 7.19E-06 |
| rs72941136 | 1 | 82245693 | C | G | 0.1043 | 0.1802 | 0.03974 | 5.99E-06 |
| rs6750281 | 2 | 99274418 | G | A | 0.4294 | 0.1112 | 0.02488 | 8.12E-06 |
| rs9883798 | 3 | 16473863 | A | C | 0.3603 | 0.1124 | 0.02525 | 8.78E-06 |
| rs2679580 | 3 | 8845344 | G | A | 0.5182 | -0.1187 | 0.02392 | 7.30E-07 |
| rs182527768 | 4 | 109048591 | A | G | 0.0342 | -0.3328 | 0.06652 | 5.91E-07 |
| rs1897558 | 5 | 167552605 | C | A | 0.2447 | -0.1305 | 0.02801 | 3.30E-06 |
| rs13173982 | 5 | 149268532 | A | G | 0.2498 | 0.1243 | 0.02807 | 9.78E-06 |
| rs10052651 | 5 | 18686084 | T | A | 0.6789 | -0.1161 | 0.02565 | 6.20E-06 |
| rs9460146 | 6 | 170723710 | C | T | 0.8277 | 0.144 | 0.03204 | 7.22E-06 |
| rs117693242 | 6 | 104445996 | G | A | 0.0159 | -0.4542 | 0.09774 | 3.48E-06 |
| rs75270114 | 6 | 147667242 | G | T | 0.0371 | 0.2916 | 0.06479 | 6.97E-06 |
| rs7834992 | 8 | 119556485 | G | A | 0.6731 | 0.1209 | 0.02608 | 3.69E-06 |
| rs2395904 | 8 | 130617819 | G | T | 0.2658 | -0.1269 | 0.02725 | 3.31E-06 |
| rs73520180 | 9 | 118756021 | C | T | 0.0216 | 0.4493 | 0.08473 | 1.21E-07 |
| rs4933299 | 10 | 85456128 | T | G | 0.2278 | -0.1282 | 0.02869 | 8.17E-06 |
| rs142089808 | 11 | 116171120 | G | A | 0.0037 | -1.048 | 0.1941 | 7.04E-08 |
| rs58114937 | 12 | 67638132 | G | A | 0.1313 | 0.1852 | 0.03578 | 2.38E-07 |
| rs1057686 | 12 | 120533696 | G | A | 0.1291 | -0.1735 | 0.0364 | 1.96E-06 |
| rs12865434 | 13 | 108970631 | G | A | 0.4777 | 0.1537 | 0.02404 | 1.83E-10 |
| rs11627066 | 14 | 66304049 | T | C | 0.4989 | -0.1147 | 0.02444 | 2.81E-06 |
| rs7500855 | 16 | 87692225 | T | C | 0.5064 | -0.1258 | 0.02435 | 2.51E-07 |
| rs7210666 | 17 | 74980293 | C | T | 0.2253 | -0.129 | 0.02915 | 9.87E-06 |
| rs1966302 | 17 | 2718946 | C | T | 0.5591 | -0.1213 | 0.02419 | 5.52E-07 |
| rs3894193 | 17 | 38125346 | G | C | 0.467 | 0.1344 | 0.02545 | 1.35E-07 |
| rs35606919 | 17 | 4353359 | G | T | 0.1033 | 0.1779 | 0.03926 | 6.02E-06 |
| rs62061834 | 17 | 49836652 | C | T | 0.0511 | 0.2608 | 0.05507 | 2.26E-06 |
| rs74390224 | 18 | 65360311 | T | A | 0.0086 | -0.5791 | 0.1301 | 8.84E-06 |
| rs11666499 | 19 | 10372310 | G | A | 0.0884 | 0.1913 | 0.0422 | 5.96E-06 |
| rs2033489 | 19 | 56768215 | T | C | 0.5761 | 0.1073 | 0.02416 | 9.17E-06 |
| rs5009526 | 20 | 38895000 | G | A | 0.1495 | -0.1516 | 0.03391 | 8.01E-06 |
| rs144825213 | 21 | 15364014 | A | G | 0.0015 | -1.553 | 0.3457 | 7.28E-06 |
| rs5756641 | 22 | 37746783 | A | G | 0.1993 | -0.1362 | 0.02972 | 4.74E-06 |

Table 23 Instrumental variables of B cell memory

| SNP | Chr | Pos | EA | OA | EAF | Beta | SE | P |
| --- | --- | --- | --- | --- | --- | --- | --- | --- |
| rs16867231 | 2 | 181050023 | A | G | 0.0108 | 0.538 | 0.1157 | 3.45E-06 |
| rs10018308 | 4 | 120657899 | G | A | 0.3441 | 0.1198 | 0.0268 | 8.09E-06 |
| rs17708396 | 5 | 148410250 | A | C | 5.00E-04 | 2.504 | 0.522 | 1.68E-06 |
| rs12216018 | 6 | 130514590 | T | C | 0.1415 | -0.1641 | 0.03601 | 5.39E-06 |
| rs74565175 | 7 | 81370578 | A | G | 0.2009 | 0.1371 | 0.03052 | 7.27E-06 |
| rs77643931 | 9 | 129171041 | G | T | 0.2798 | 0.1235 | 0.02777 | 8.97E-06 |
| rs12763172 | 10 | 85447519 | C | T | 0.7213 | 0.1259 | 0.02783 | 6.27E-06 |
| rs2879633 | 10 | 53757050 | A | G | 0.2884 | -0.1301 | 0.02775 | 2.84E-06 |
| rs36072728 | 11 | 29448646 | T | C | 0.0356 | 0.305 | 0.06718 | 5.81E-06 |
| rs557577736 | 13 | 46224092 | G | A | 0.001 | 1.829 | 0.3891 | 2.69E-06 |
| rs12874404 | 13 | 108993494 | G | A | 0.3256 | 0.173 | 0.02661 | 9.14E-11 |
| rs61997631 | 14 | 104600920 | G | C | 0.0432 | -0.2972 | 0.06141 | 1.36E-06 |
| rs77762131 | 14 | 96205838 | C | G | 0.045 | 0.2713 | 0.05795 | 2.95E-06 |
| rs139196765 | 15 | 24733764 | G | A | 0.0025 | 1.204 | 0.2523 | 1.90E-06 |
| rs11653761 | 17 | 2716535 | T | G | 0.592 | -0.1161 | 0.02478 | 2.94E-06 |
| rs144295598 | 22 | 45164171 | A | G | 0.0103 | 0.6102 | 0.1207 | 4.46E-07 |

Table 24 Instrumental variables of CD4+ T cell resting

| SNP | Chr | Pos | EA | OA | EAF | Beta | SE | P |
| --- | --- | --- | --- | --- | --- | --- | --- | --- |
| rs12044365 | 1 | 210431761 | G | A | 0.0248 | -0.3482 | 0.07468 | 3.24E-06 |
| rs116794175 | 1 | 194010208 | C | T | 0.0043 | 0.8994 | 0.1806 | 6.68E-07 |
| rs17583875 | 1 | 197924770 | A | G | 0.0029 | 1.651 | 0.2111 | 6.94E-15 |
| rs6751481 | 2 | 38897810 | C | T | 0.4699 | 0.2177 | 0.02348 | 3.10E-20 |
| rs79244630 | 2 | 130435634 | T | C | 0.0352 | 0.2841 | 0.0642 | 9.93E-06 |
| rs17607399 | 2 | 41884212 | T | C | 0.1084 | 0.1892 | 0.03839 | 8.66E-07 |
| rs408686 | 2 | 39177320 | A | C | 0.814 | -0.2089 | 0.03078 | 1.33E-11 |
| rs111714509 | 3 | 124800996 | A | G | 0.0056 | 0.6777 | 0.149 | 5.62E-06 |
| rs979640 | 4 | 132525794 | G | A | 0.7852 | -0.1339 | 0.02833 | 2.40E-06 |
| rs247636 | 5 | 142990496 | A | G | 0.0363 | -0.3186 | 0.06187 | 2.77E-07 |
| rs36058358 | 6 | 32636282 | A | G | 0.2229 | -0.1784 | 0.03687 | 1.37E-06 |
| rs9272050 | 6 | 32599071 | A | G | 0.7012 | 0.1633 | 0.03024 | 7.10E-08 |
| rs1618199 | 6 | 31275850 | A | G | 0.3771 | 0.1509 | 0.0328 | 4.38E-06 |
| rs182630194 | 7 | 71331239 | A | G | 0.0013 | 1.635 | 0.3213 | 3.81E-07 |
| rs147199078 | 7 | 80542211 | C | T | 0.0295 | 0.3441 | 0.06816 | 4.69E-07 |
| rs7787732 | 7 | 151861663 | C | T | 0.0793 | 0.1935 | 0.04143 | 3.14E-06 |
| rs7899451 | 10 | 118980344 | G | C | 0.2803 | -0.1238 | 0.02622 | 2.45E-06 |
| rs16922479 | 10 | 22863004 | C | T | 0.1094 | -0.1773 | 0.03726 | 2.03E-06 |
| rs3016770 | 11 | 128694040 | G | T | 0.89 | -0.1819 | 0.03707 | 9.74E-07 |
| rs75958070 | 11 | 79203049 | C | G | 0.1217 | -0.2006 | 0.03612 | 3.02E-08 |
| rs73060045 | 12 | 12168131 | G | A | 0.1026 | -0.1712 | 0.03829 | 8.01E-06 |
| rs72702642 | 14 | 99672909 | G | A | 0.0341 | 0.2877 | 0.06401 | 7.22E-06 |
| rs117432559 | 15 | 40508902 | G | A | 0.0142 | -0.429 | 0.09447 | 5.80E-06 |
| rs8088253 | 18 | 64904859 | G | A | 0.0467 | 0.2852 | 0.05469 | 1.94E-07 |
| rs11703793 | 22 | 27946507 | C | T | 0.0103 | -0.5089 | 0.1131 | 7.09E-06 |
| rs111941642 | 22 | 28079761 | T | C | 0.0144 | -0.476 | 0.09652 | 8.56E-07 |

Table 25 Instrumental variables of CD8+ T cell

| SNP | Chr | Pos | EA | OA | EAF | Beta | SE | P |
| --- | --- | --- | --- | --- | --- | --- | --- | --- |
| rs12130486 | 1 | 81455274 | C | T | 0.0245 | 0.36 | 0.07639 | 2.54E-06 |
| rs12119984 | 1 | 246874501 | G | A | 0.0084 | -0.6212 | 0.1311 | 2.24E-06 |
| rs78032118 | 2 | 66964383 | T | C | 0.0063 | 0.6899 | 0.1458 | 2.32E-06 |
| rs113985770 | 2 | 210377930 | A | G | 0.0101 | -0.5533 | 0.1242 | 8.68E-06 |
| rs6858602 | 4 | 179936294 | C | T | 0.4618 | -0.1209 | 0.02381 | 3.96E-07 |
| rs3130685 | 6 | 31206206 | T | C | 0.3428 | 0.1887 | 0.0284 | 3.52E-11 |
| rs75424572 | 6 | 127364239 | G | T | 0.0403 | 0.2774 | 0.06193 | 7.73E-06 |
| rs12195711 | 6 | 50386986 | T | C | 0.1314 | 0.1703 | 0.0353 | 1.46E-06 |
| rs28895267 | 6 | 32445916 | T | C | 0.3161 | 0.1842 | 0.03036 | 1.43E-09 |
| rs6915999 | 6 | 31377851 | A | G | 0.5394 | -0.1436 | 0.02774 | 2.36E-07 |
| rs79647440 | 7 | 142438302 | T | C | 4.00E-04 | 3.671 | 0.8061 | 5.43E-06 |
| rs11772710 | 7 | 132433567 | T | C | 0.4633 | -0.1099 | 0.02405 | 5.01E-06 |
| rs1550856 | 8 | 117805397 | G | A | 0.7751 | -0.1417 | 0.02903 | 1.10E-06 |
| rs11197647 | 10 | 118142066 | T | G | 0.0754 | 0.2221 | 0.04476 | 7.32E-07 |
| rs1980812 | 10 | 90274583 | G | A | 0.5531 | -0.1135 | 0.02467 | 4.34E-06 |
| rs11060493 | 12 | 130160924 | C | A | 0.102 | 0.1796 | 0.03884 | 3.91E-06 |
| rs28415769 | 14 | 94358762 | C | T | 0.3586 | -0.11 | 0.02484 | 9.76E-06 |
| rs117391590 | 17 | 42759948 | A | G | 0.0413 | -0.2742 | 0.05955 | 4.29E-06 |
| rs35238206 | 17 | 75698346 | G | A | 0.2868 | 0.1229 | 0.02638 | 3.33E-06 |
| rs11080350 | 17 | 33774635 | T | C | 0.4018 | -0.1209 | 0.02469 | 1.02E-06 |
| rs41276984 | 20 | 37580670 | G | C | 0.0011 | 1.76 | 0.3677 | 1.77E-06 |
| rs2837838 | 21 | 42191862 | G | A | 0.9259 | 0.2056 | 0.04518 | 5.54E-06 |
| rs2267983 | 22 | 50698157 | A | C | 0.3924 | -0.1266 | 0.02472 | 3.18E-07 |

Table 26 Instrumental variables of T cell

| SNP | Chr | Pos | EA | OA | EAF | Beta | SE | P |
| --- | --- | --- | --- | --- | --- | --- | --- | --- |
| rs12124698 | 1 | 246883687 | T | A | 0.007 | -0.6963 | 0.1564 | 8.78E-06 |
| rs75308045 | 1 | 111150195 | G | A | 0.3063 | -0.1332 | 0.02697 | 8.25E-07 |
| rs17339549 | 2 | 80078045 | A | G | 0.3597 | 0.1161 | 0.02516 | 4.12E-06 |
| rs17283318 | 3 | 151749774 | T | C | 0.0452 | -0.274 | 0.0597 | 4.59E-06 |
| rs76560336 | 4 | 33455497 | T | C | 0.006 | -0.6975 | 0.1553 | 7.33E-06 |
| rs10051472 | 5 | 79563530 | C | G | 0.3492 | 0.116 | 0.02567 | 6.42E-06 |
| rs9271622 | 6 | 32591845 | A | G | 0.7473 | 0.2154 | 0.03382 | 2.14E-10 |
| rs72960548 | 6 | 127873865 | C | T | 0.073 | 0.2563 | 0.04796 | 9.62E-08 |
| rs13207893 | 6 | 32586922 | C | T | 0.297 | -0.1488 | 0.03084 | 1.45E-06 |
| rs62447160 | 7 | 36527098 | C | T | 0.6388 | -0.1152 | 0.02524 | 5.12E-06 |
| rs1550856 | 8 | 117805397 | G | A | 0.7751 | -0.1325 | 0.0295 | 7.32E-06 |
| rs139994967 | 11 | 79921187 | G | A | 0.1024 | 0.1828 | 0.03991 | 4.80E-06 |
| rs3184504 | 12 | 111884608 | C | T | 0.5304 | -0.1468 | 0.02633 | 2.65E-08 |
| rs11850806 | 14 | 78669137 | G | T | 0.0083 | -0.6286 | 0.1291 | 1.17E-06 |
| rs12599179 | 16 | 86449253 | T | A | 0.0278 | -0.3225 | 0.07182 | 7.32E-06 |
| rs117391590 | 17 | 42759948 | A | G | 0.0413 | -0.2678 | 0.06035 | 9.40E-06 |
| rs35669214 | 17 | 71372662 | C | T | 0.254 | -0.1231 | 0.02758 | 8.31E-06 |
| rs75995320 | 20 | 14839626 | A | G | 0.0171 | -0.4299 | 0.08871 | 1.31E-06 |
| rs414743 | 21 | 46512075 | A | G | 0.2368 | 0.1311 | 0.02887 | 5.74E-06 |
| rs11702153 | 21 | 14911701 | T | C | 0.4347 | 0.1568 | 0.03447 | 5.55E-06 |

Table 27 Instrumental variables of B cell naive

| SNP | Chr | Pos | EA | OA | EAF | Beta | SE | P |
| --- | --- | --- | --- | --- | --- | --- | --- | --- |
| rs139077655 | 2 | 132741527 | T | C | 0.382 | -0.1159 | 0.02519 | 4.37E-06 |
| rs76981518 | 4 | 152777207 | T | C | 0.0744 | -0.2134 | 0.04708 | 6.01E-06 |
| rs115150472 | 5 | 146138788 | T | C | 4.00E-04 | 2.627 | 0.579 | 5.91E-06 |
| rs10042327 | 5 | 58278611 | A | G | 0.0613 | 0.233 | 0.05192 | 7.40E-06 |
| rs17056318 | 5 | 158285953 | C | T | 0.081 | -0.2366 | 0.0457 | 2.36E-07 |
| rs9354413 | 6 | 67039330 | C | T | 0.2964 | 0.1232 | 0.02743 | 7.30E-06 |
| rs35607968 | 7 | 67653879 | T | C | 0.1536 | 0.1584 | 0.03419 | 3.71E-06 |
| rs2390504 | 7 | 21321670 | A | G | 0.0486 | -0.2668 | 0.05743 | 3.51E-06 |
| rs143397127 | 7 | 12847391 | C | T | 0.0157 | -0.4624 | 0.09926 | 3.30E-06 |
| rs2395904 | 8 | 130617819 | G | T | 0.2657 | -0.1445 | 0.02761 | 1.74E-07 |
| rs283340 | 8 | 70193536 | C | T | 0.5621 | 0.1251 | 0.02477 | 4.60E-07 |
| rs189761842 | 10 | 125485345 | T | C | 0.0055 | 0.7902 | 0.1709 | 3.92E-06 |
| rs117344365 | 10 | 33779474 | A | G | 0.0178 | -0.4129 | 0.09241 | 8.13E-06 |
| rs12799241 | 11 | 119980729 | A | G | 0.2619 | 0.138 | 0.02805 | 9.07E-07 |
| rs2286599 | 12 | 6499533 | A | G | 0.32 | 0.1183 | 0.0266 | 8.96E-06 |
| rs58114937 | 12 | 67638132 | G | A | 0.1302 | 0.1702 | 0.0363 | 2.84E-06 |
| rs9564767 | 13 | 71534535 | G | A | 0.6075 | -0.1163 | 0.02507 | 3.63E-06 |
| rs3811215 | 14 | 22954777 | T | G | 0.3032 | -0.1229 | 0.02697 | 5.33E-06 |
| rs76263497 | 15 | 60878804 | A | G | 0.0989 | -0.1886 | 0.04138 | 5.34E-06 |
| rs143176410 | 16 | 84305239 | C | G | 0.001 | 1.819 | 0.4058 | 7.65E-06 |
| rs77983244 | 17 | 7316027 | A | G | 0.0325 | -0.3088 | 0.06711 | 4.34E-06 |
| rs62067029 | 17 | 38038391 | T | A | 0.4148 | -0.1605 | 0.02868 | 2.35E-08 |
| rs62115322 | 19 | 53848484 | G | C | 0.2083 | 0.1382 | 0.02968 | 3.31E-06 |
| rs34764986 | 20 | 59926554 | C | T | 0.0699 | -0.2347 | 0.04805 | 1.08E-06 |

Table 28 Instrumental variables of CD4+ T cell activated

| SNP | Chr | Pos | EA | OA | EAF | Beta | SE | P |
| --- | --- | --- | --- | --- | --- | --- | --- | --- |
| rs113435341 | 1 | 198151688 | G | A | 0.0032 | -1.414 | 0.2277 | 5.88E-10 |
| rs139138505 | 1 | 225667295 | G | C | 0.0085 | -0.6234 | 0.1355 | 4.38E-06 |
| rs138915779 | 1 | 190079170 | A | C | 0.0034 | -1.288 | 0.2345 | 4.23E-08 |
| rs34596162 | 2 | 195102952 | C | T | 0.0533 | 0.2633 | 0.0593 | 9.30E-06 |
| rs6901124 | 6 | 109035704 | T | C | 0.0044 | 0.9001 | 0.1901 | 2.27E-06 |
| rs146746694 | 6 | 155566192 | A | G | 0.0101 | 0.5526 | 0.1249 | 9.99E-06 |
| rs17509082 | 7 | 120927706 | A | C | 0.1816 | 0.1467 | 0.03297 | 8.95E-06 |
| rs62489142 | 7 | 135616195 | C | G | 0.0138 | -0.5075 | 0.1132 | 7.64E-06 |
| rs11593335 | 10 | 6160702 | T | A | 0.4164 | -0.1265 | 0.02623 | 1.48E-06 |
| rs148991751 | 12 | 53114409 | C | A | 4.00E-04 | -2.9 | 0.633 | 4.78E-06 |
| rs145600407 | 13 | 19280601 | T | C | 0.0206 | -0.4684 | 0.1035 | 6.25E-06 |
| rs142987815 | 15 | 98954485 | C | T | 0.0166 | -0.4526 | 0.09903 | 5.05E-06 |
| rs8064831 | 17 | 10150766 | T | C | 0.3626 | -0.1164 | 0.02611 | 8.58E-06 |
| rs10515986 | 18 | 60125602 | G | A | 0.1517 | 0.1569 | 0.035 | 7.65E-06 |
| rs66939386 | 19 | 45564915 | G | A | 0.647 | -0.1186 | 0.02662 | 8.75E-06 |
| rs12626270 | 21 | 34223997 | T | C | 0.1232 | 0.1817 | 0.03912 | 3.55E-06 |

Table 29 Instrumental variables of Neutrophil cell

| SNP | Chr | Pos | EA | OA | EAF | Beta | SE | P |
| --- | --- | --- | --- | --- | --- | --- | --- | --- |
| rs3917932 | 1 | 36943916 | G | C | 0.577138 | -0.05153 | 0.001947 | 3.00E-154 |
| rs6678033 | 1 | 66077624 | A | G | 0.367919 | -0.042708 | 0.001986 | 1.46E-102 |
| rs11121225 | 1 | 8816438 | A | G | 0.179035 | 0.024073 | 0.002501 | 6.59E-22 |
| rs192280464 | 2 | 219214529 | T | A | 0.009075 | -0.151109 | 0.011504 | 2.26E-39 |
| rs6734238 | 2 | 113841030 | G | A | 0.40298 | 0.03936 | 0.00195 | 1.58E-90 |
| rs6743819 | 2 | 27567407 | T | G | 0.395906 | -0.020804 | 0.001962 | 3.04E-26 |
| rs13068402 | 3 | 42875756 | C | T | 0.321166 | -0.021737 | 0.002055 | 3.98E-26 |
| rs2734031 | 3 | 128301390 | C | T | 0.907968 | -0.067521 | 0.003335 | 4.20E-91 |
| rs10031141 | 4 | 74594043 | G | A | 0.40419 | 0.040683 | 0.00201 | 5.03E-91 |
| rs1313766 | 4 | 3052825 | C | T | 0.507094 | 0.016582 | 0.001928 | 8.23E-18 |
| rs1371794 | 4 | 74948054 | C | A | 0.622286 | -0.07253 | 0.00198 | 1.00E-200 |
| rs2561758 | 5 | 173205282 | G | A | 0.72298 | -0.034011 | 0.002173 | 3.49E-55 |
| rs10078004 | 5 | 148199242 | A | G | 0.554333 | -0.029577 | 0.001958 | 1.60E-51 |
| rs4705938 | 5 | 131694077 | C | T | 0.474331 | -0.022666 | 0.001924 | 5.18E-32 |
| rs9378212 | 6 | 32445691 | T | C | 0.469078 | 0.059685 | 0.001926 | 1.00E-200 |
| rs9494308 | 6 | 135948804 | C | G | 0.343286 | -0.01754 | 0.00202 | 4.00E-18 |
| rs2280774 | 6 | 31928691 | A | G | 0.303509 | 0.037766 | 0.002092 | 8.79E-73 |
| rs1137160 | 6 | 29912386 | C | G | 0.096929 | 0.029275 | 0.003264 | 3.21E-19 |
| rs572021 | 7 | 28287940 | A | T | 0.34242 | -0.040469 | 0.002023 | 5.51E-89 |
| rs56388170 | 7 | 28724374 | T | G | 0.293956 | 0.070039 | 0.002132 | 1.00E-200 |
| rs342290 | 7 | 106367604 | C | A | 0.453583 | -0.015629 | 0.001925 | 4.86E-16 |
| rs601088 | 8 | 103914604 | C | A | 0.183324 | -0.020145 | 0.002476 | 4.24E-16 |
| rs7846314 | 8 | 61650831 | T | A | 0.187341 | 0.063921 | 0.002458 | 4.70E-149 |
| rs4841407 | 8 | 10516185 | A | G | 0.566656 | 0.021723 | 0.001938 | 4.07E-29 |
| rs10817484 | 9 | 116108146 | A | G | 0.354819 | -0.016982 | 0.002011 | 3.18E-17 |
| rs2519093 | 9 | 136141870 | T | C | 0.184749 | -0.040099 | 0.00248 | 9.83E-59 |
| rs4413892 | 9 | 139330158 | A | G | 0.277885 | 0.033654 | 0.002228 | 1.65E-51 |
| rs9971228 | 10 | 25193318 | T | A | 0.421613 | -0.03695 | 0.001961 | 3.86E-79 |
| rs17156193 | 10 | 44859439 | G | A | 0.207848 | -0.017671 | 0.002402 | 1.94E-13 |
| rs10786325 | 10 | 99068738 | G | C | 0.595938 | 0.042348 | 0.001955 | 6.05E-104 |
| rs7115703 | 11 | 306920 | A | T | 0.513619 | 0.044398 | 0.00202 | 5.38E-107 |
| rs4936290 | 11 | 114009255 | C | A | 0.347229 | 0.024848 | 0.002027 | 1.67E-34 |
| rs3184504 | 12 | 111884608 | C | T | 0.517462 | -0.029388 | 0.001927 | 1.90E-52 |
| rs10858730 | 12 | 88827890 | C | G | 0.840386 | -0.027248 | 0.002628 | 3.64E-25 |
| rs76428106 | 13 | 28604007 | C | T | 0.013438 | 0.104475 | 0.008878 | 6.25E-32 |
| rs2038700 | 14 | 25461989 | C | T | 0.394281 | 0.033462 | 0.00197 | 1.25E-64 |
| rs34112413 | 14 | 69840902 | G | A | 0.065616 | 0.04138 | 0.003888 | 2.04E-26 |
| rs28576226 | 15 | 101713668 | A | G | 0.122779 | 0.040318 | 0.002974 | 8.14E-42 |
| rs72726027 | 15 | 42248826 | C | T | 0.11173 | -0.043218 | 0.003051 | 1.71E-45 |
| rs305082 | 16 | 85936978 | C | T | 0.172255 | 0.036633 | 0.002594 | 3.04E-45 |
| rs4984803 | 16 | 1349929 | A | G | 0.595619 | -0.017523 | 0.00205 | 1.34E-17 |
| rs4795281 | 17 | 36616059 | C | T | 0.349503 | 0.018139 | 0.002046 | 7.92E-19 |
| rs4794820 | 17 | 38089344 | G | A | 0.551375 | 0.076088 | 0.001942 | 1.00E-200 |
| rs28678167 | 17 | 41172481 | T | G | 0.319886 | -0.021191 | 0.002084 | 3.00E-24 |
| rs75957619 | 18 | 41995504 | G | A | 0.131722 | 0.030844 | 0.002862 | 4.83E-27 |
| rs303753 | 18 | 21074922 | A | G | 0.345097 | -0.022761 | 0.002045 | 9.57E-29 |
| rs344812 | 19 | 45791838 | C | A | 0.306791 | -0.035385 | 0.0021 | 1.22E-63 |
| rs4760 | 19 | 44153100 | G | A | 0.153372 | -0.075213 | 0.002693 | 1.42E-171 |
| rs1800961 | 20 | 43042364 | T | C | 0.031018 | -0.051942 | 0.005553 | 8.99E-21 |
| rs4809327 | 20 | 62348190 | T | C | 0.672724 | 0.016278 | 0.002061 | 2.95E-15 |
| rs9977672 | 21 | 40463283 | A | G | 0.2588 | -0.022323 | 0.002215 | 7.34E-24 |
| rs5747308 | 22 | 18133500 | C | A | 0.504471 | 0.022058 | 0.001925 | 2.30E-30 |
| rs9606835 | 22 | 31766960 | C | A | 0.480486 | -0.017816 | 0.001931 | 2.94E-20 |

Table 30 Instrumental variables of Plasma cell

| SNP | Chr | Pos | EA | OA | EAF | Beta | SE | P |
| --- | --- | --- | --- | --- | --- | --- | --- | --- |
| rs80297308 | 1 | 102294391 | T | C | 0.0015 | 1.467 | 0.3075 | 1.91E-06 |
| rs10800645 | 1 | 199586940 | A | G | 0.5844 | 0.1124 | 0.02479 | 5.95E-06 |
| rs74458303 | 2 | 121699864 | A | G | 4.00E-04 | 2.692 | 0.5745 | 2.90E-06 |
| rs148362575 | 2 | 187165335 | T | A | 8.00E-04 | 2.566 | 0.5486 | 3.03E-06 |
| rs148389014 | 3 | 188082431 | T | C | 0.0051 | 0.7633 | 0.1696 | 7.02E-06 |
| rs116540222 | 3 | 117331256 | T | C | 0.0011 | 1.671 | 0.3677 | 5.68E-06 |
| rs149048155 | 3 | 957228 | C | A | 0.036 | -0.2902 | 0.06467 | 7.45E-06 |
| rs62353585 | 4 | 168102710 | C | T | 0.0227 | 0.3653 | 0.07865 | 3.53E-06 |
| rs76709581 | 5 | 179574693 | C | T | 0.1061 | -0.1773 | 0.03843 | 4.06E-06 |
| rs7449190 | 5 | 1297918 | C | T | 0.7401 | 0.1236 | 0.02749 | 7.10E-06 |
| rs61294310 | 6 | 5266557 | G | A | 0.0124 | 0.5137 | 0.1072 | 1.74E-06 |
| rs4506076 | 6 | 8860293 | C | A | 0.8043 | -0.1344 | 0.03016 | 8.54E-06 |
| rs149559870 | 8 | 39638049 | T | C | 0.0157 | 0.4466 | 0.09807 | 5.43E-06 |
| rs74565954 | 8 | 24520468 | T | C | 0.0135 | 0.4919 | 0.1035 | 2.10E-06 |
| rs62519719 | 8 | 118376967 | A | G | 0.2815 | -0.1239 | 0.02639 | 2.79E-06 |
| rs12412378 | 10 | 130483163 | A | C | 0.1608 | -0.16 | 0.03222 | 7.19E-07 |
| rs1681588 | 14 | 23098304 | C | T | 0.5559 | 0.1086 | 0.02407 | 6.62E-06 |
| rs17765677 | 16 | 65999760 | C | T | 0.0304 | -0.3422 | 0.07069 | 1.34E-06 |
| rs7185719 | 16 | 59859266 | G | C | 0.732 | -0.1228 | 0.02721 | 6.56E-06 |
| rs112471525 | 17 | 52797903 | G | A | 0.0189 | 0.3865 | 0.0862 | 7.58E-06 |
| rs72883992 | 18 | 24475181 | C | G | 0.0807 | 0.1932 | 0.04342 | 8.82E-06 |
| rs4806937 | 19 | 3443405 | A | C | 0.3229 | -0.1145 | 0.02557 | 7.79E-06 |
| rs6014246 | 20 | 53613034 | A | G | 0.3112 | -0.1186 | 0.02577 | 4.30E-06 |
| rs4820558 | 22 | 21022887 | G | C | 0.466 | 0.1172 | 0.02408 | 1.19E-06 |
| rs28652751 | 22 | 35861208 | A | T | 0.3179 | -0.1141 | 0.02564 | 8.86E-06 |

Table 31 Instrumental variables of IL-4

| SNP | EA | OA | Beat | SE | P |
| --- | --- | --- | --- | --- | --- |
| rs11709228 | C | G | 0.3003 | 0.0677 | 9.16E-06 |
| rs9863516 | A | G | 0.079 | 0.0178 | 8.70E-06 |
| rs17713451 | A | G | 0.1255 | 0.0252 | 6.41E-07 |
| rs79597994 | T | C | -0.5855 | 0.1271 | 4.06E-06 |
| rs73023729 | A | G | -0.1796 | 0.0365 | 8.56E-07 |
| rs10512267 | T | C | -0.0824 | 0.016 | 2.73E-07 |
| rs2073438 | A | G | 0.0847 | 0.0183 | 3.73E-06 |
| rs12091966 | A | G | 0.0766 | 0.0171 | 7.25E-06 |
| rs6969391 | T | C | 0.0767 | 0.0166 | 3.59E-06 |
| rs2849346 | C | G | 0.0765 | 0.0164 | 3.22E-06 |
| rs9941733 | A | G | 0.1156 | 0.0229 | 4.33E-07 |
| rs9508291 | T | C | -0.168 | 0.0358 | 2.67E-06 |
| rs6765768 | A | G | 0.0796 | 0.0167 | 1.85E-06 |
| rs117146485 | T | C | -0.2856 | 0.0625 | 4.95E-06 |
| rs10952260 | T | G | 0.0765 | 0.0173 | 9.78E-06 |
| rs12238729 | T | C | 0.5271 | 0.1096 | 1.51E-06 |
| rs116705532 | T | G | -0.4675 | 0.0978 | 1.73E-06 |
| rs75924875 | A | G | -0.1742 | 0.0385 | 5.98E-06 |
| rs6979813 | C | G | -0.0731 | 0.0163 | 7.67E-06 |
| rs13106889 | A | T | -0.1186 | 0.0224 | 1.22E-07 |
| rs7613691 | A | G | 0.1787 | 0.0382 | 2.96E-06 |
| rs78876292 | T | C | -0.2901 | 0.0646 | 7.00E-06 |

Table 32 Instrumental variables of IFN-γ

| SNP | EA | OA | Beat | SE | P |
| --- | --- | --- | --- | --- | --- |
| rs11227289 | T | G | -0.0744 | 0.0164 | 5.87E-06 |
| rs10481651 | A | G | -0.0793 | 0.0168 | 2.18E-06 |
| rs149300774 | A | C | -0.4158 | 0.0941 | 9.87E-06 |
| rs2073438 | A | G | 0.092 | 0.0188 | 9.55E-07 |
| rs60059008 | A | G | 0.0852 | 0.0176 | 1.30E-06 |
| rs11843756 | T | G | 0.1812 | 0.0391 | 3.62E-06 |
| rs73479333 | C | G | -0.1123 | 0.024 | 2.82E-06 |
| rs78296352 | T | G | 0.3419 | 0.065 | 1.42E-07 |
| rs74148555 | T | C | -0.3771 | 0.077 | 9.86E-07 |
| rs192914199 | T | G | 0.4705 | 0.106 | 9.10E-06 |
| rs27385 | T | C | 0.1278 | 0.0288 | 8.81E-06 |
| rs12420286 | T | C | 0.2357 | 0.05 | 2.45E-06 |
| rs112783231 | A | G | -0.2323 | 0.0509 | 5.11E-06 |
| rs72910442 | T | C | 0.2754 | 0.062 | 8.95E-06 |
| rs2188420 | C | G | 0.1005 | 0.0201 | 5.90E-07 |
| rs115729819 | A | G | 0.2511 | 0.0514 | 1.05E-06 |
| rs10761731 | A | T | -0.0813 | 0.0167 | 1.07E-06 |
| rs113600793 | A | C | 0.1871 | 0.0371 | 4.43E-07 |

Table 33 Instrumental variables of IL-1**α**

| SNP | EA | OA | Beat | SE | P |
| --- | --- | --- | --- | --- | --- |
| rs2809154 | T | C | -0.1751 | 0.0386 | 5.74E-06 |
| rs4783191 | T | C | 0.1527 | 0.0338 | 6.29E-06 |
| rs187166731 | T | C | -0.2424 | 0.0504 | 1.55E-06 |
| rs61335305 | A | C | 0.4315 | 0.0904 | 1.81E-06 |
| rs6699436 | A | G | -0.1858 | 0.0404 | 4.37E-06 |
| rs4566030 | A | G | -0.138 | 0.0308 | 7.66E-06 |
| rs11869294 | C | G | -0.2286 | 0.047 | 1.13E-06 |
| rs74435620 | A | G | -0.2144 | 0.048 | 7.97E-06 |
| rs3876037 | A | G | 0.1234 | 0.027 | 4.73E-06 |
| rs4441609 | T | C | 0.1056 | 0.0231 | 4.75E-06 |
| rs56134659 | A | G | -0.1109 | 0.0236 | 2.56E-06 |
| rs1054402 | T | C | 0.1325 | 0.0269 | 8.20E-07 |
| rs1522921 | C | G | -0.1084 | 0.0244 | 9.10E-06 |
| rs11627423 | A | C | 0.1178 | 0.0246 | 1.65E-06 |
| rs147747784 | C | G | 0.3582 | 0.0754 | 2.04E-06 |
| rs12121840 | T | C | 0.2584 | 0.0568 | 5.40E-06 |

Table 34 Instrumental variables of IL-1β

| SNP | EA | OA | Beat | SE | P |
| --- | --- | --- | --- | --- | --- |
| rs9898641 | T | C | -0.2787 | 0.0614 | 5.60E-06 |
| rs61335305 | A | C | 0.4333 | 0.0928 | 3.02E-06 |
| rs9891997 | A | G | 0.1196 | 0.0269 | 8.90E-06 |
| rs62015704 | A | G | 0.1786 | 0.0372 | 1.62E-06 |
| rs143319329 | T | C | 0.4357 | 0.093 | 2.84E-06 |
| rs1942793 | T | G | 0.1109 | 0.0247 | 7.05E-06 |
| rs115242021 | A | C | 0.2795 | 0.0553 | 4.25E-07 |
| rs35045206 | T | C | 0.4373 | 0.0961 | 5.33E-06 |
| rs12913251 | T | C | 0.1141 | 0.0256 | 8.57E-06 |
| rs12772183 | A | C | -0.1164 | 0.0262 | 8.97E-06 |

Table 35 Instrumental variables of IL-6

| SNP | EA | OA | Beat | SE | P |
| --- | --- | --- | --- | --- | --- |
| rs13412535 | A | G | -0.1186 | 0.0214 | 3.14E-08 |
| rs72831623 | A | G | 0.197 | 0.0369 | 9.29E-08 |
| rs4684700 | T | C | -0.0747 | 0.0162 | 3.91E-06 |
| rs76856708 | T | C | 0.336 | 0.0697 | 1.43E-06 |
| rs9288968 | T | C | -0.0724 | 0.0163 | 8.89E-06 |
| rs1744422 | A | T | -0.0714 | 0.0161 | 8.95E-06 |
| rs1884910 | C | G | 0.0763 | 0.0169 | 6.41E-06 |
| rs73273528 | T | C | 0.268 | 0.0553 | 1.25E-06 |
| rs141772556 | T | G | 0.268 | 0.0602 | 8.40E-06 |
| rs8089344 | C | G | -0.1166 | 0.0261 | 7.76E-06 |
| rs113098456 | A | G | -0.1553 | 0.0339 | 4.64E-06 |
| rs10982213 | A | G | -0.0849 | 0.0176 | 1.35E-06 |
| rs1333040 | T | C | 0.0747 | 0.0157 | 1.99E-06 |
| rs2404476 | A | G | 0.0734 | 0.0156 | 2.68E-06 |
| rs4639449 | A | G | -0.0737 | 0.0163 | 6.42E-06 |
| rs7955530 | T | C | -0.0757 | 0.0169 | 7.92E-06 |
| rs75101555 | C | G | -0.3625 | 0.0781 | 3.44E-06 |
| rs10752777 | A | T | 0.1083 | 0.0235 | 4.17E-06 |

Table 36 Instrumental variables of IL-8

| SNP | EA | OA | Beat | SE | P |
| --- | --- | --- | --- | --- | --- |
| rs141926526 | A | C | -0.6221 | 0.1308 | 1.96E-06 |
| rs12075 | A | G | 0.1148 | 0.0235 | 9.97E-07 |
| rs17866606 | T | C | -0.1482 | 0.0325 | 5.12E-06 |
| rs116726256 | T | C | -0.2247 | 0.0489 | 4.26E-06 |
| rs183628733 | T | C | 0.6547 | 0.1417 | 3.82E-06 |
| rs12438669 | A | C | -0.1182 | 0.0252 | 2.60E-06 |
| rs79274420 | A | G | 0.581 | 0.1291 | 6.75E-06 |
| rs2673604 | A | C | -0.118 | 0.0254 | 3.29E-06 |
| rs1508652 | T | C | -0.1919 | 0.0434 | 9.62E-06 |
| rs3786107 | A | G | 0.2463 | 0.0517 | 1.94E-06 |
| rs75840288 | A | C | 0.5125 | 0.1121 | 4.85E-06 |
| rs2552220 | A | G | -0.1103 | 0.0243 | 5.46E-06 |

Table 37 Instrumental variables of IL-10

| SNP | EA | OA | Beat | SE | P |
| --- | --- | --- | --- | --- | --- |
| rs2294689 | C | G | -0.0836 | 0.0187 | 7.81E-06 |
| rs41282660 | A | G | -0.1169 | 0.0254 | 4.23E-06 |
| rs6085948 | A | G | 0.0977 | 0.0202 | 1.28E-06 |
| rs3002131 | C | G | 0.1191 | 0.026 | 4.59E-06 |
| rs3965704 | T | C | 0.0758 | 0.0171 | 8.97E-06 |
| rs383684 | A | G | 0.092 | 0.0197 | 3.17E-06 |
| rs339203 | T | C | 0.0954 | 0.0203 | 2.75E-06 |
| rs282258 | T | C | 0.0993 | 0.0162 | 8.63E-10 |
| rs9951418 | A | C | -0.0898 | 0.0198 | 5.99E-06 |
| rs6921438 | A | G | -0.2876 | 0.0166 | 1.38E-67 |
| rs16863495 | A | T | -0.0962 | 0.0217 | 9.36E-06 |
| rs2375980 | C | G | 0.0844 | 0.0165 | 2.97E-07 |
| rs10493718 | A | C | -0.1081 | 0.0222 | 1.07E-06 |
| rs3025021 | T | C | 0.0913 | 0.0194 | 2.61E-06 |
| rs2086656 | T | C | -0.08 | 0.017 | 2.59E-06 |
| rs10457128 | A | G | -0.0854 | 0.0172 | 6.96E-07 |
| rs6799107 | T | C | -0.095 | 0.0206 | 3.99E-06 |
| rs4345303 | T | C | -0.083 | 0.0183 | 6.13E-06 |
| rs7088799 | T | G | -0.0815 | 0.0166 | 9.35E-07 |
| rs10888839 | C | G | 0.1203 | 0.025 | 1.56E-06 |
| rs1530455 | T | C | 0.082 | 0.0174 | 2.53E-06 |
| rs111913416 | A | T | -0.0846 | 0.0172 | 8.16E-07 |

Table 38 Instrumental variables of IL-12

| SNP | EA | OA | Beat | SE | P |
| --- | --- | --- | --- | --- | --- |
| rs2123852 | T | C | 0.0942 | 0.0204 | 3.73E-06 |
| rs782107 | A | G | 0.0765 | 0.0156 | 9.13E-07 |
| rs72831623 | A | G | 0.1929 | 0.0367 | 1.51E-07 |
| rs144160960 | A | C | -0.2477 | 0.0545 | 5.51E-06 |
| rs6993770 | A | T | 0.0918 | 0.0188 | 1.06E-06 |
| rs282258 | T | C | 0.0726 | 0.0156 | 3.28E-06 |
| rs6921438 | A | G | -0.3784 | 0.016 | 5.78E-124 |
| rs34291323 | T | C | 0.0954 | 0.0198 | 1.49E-06 |
| rs137921 | T | C | -0.1436 | 0.0325 | 9.66E-06 |
| rs71361173 | T | G | 0.1105 | 0.0238 | 3.57E-06 |
| rs9472183 | A | G | -0.1006 | 0.0157 | 1.38E-10 |
| rs10958542 | A | C | -0.0717 | 0.0159 | 6.86E-06 |
| rs79121401 | T | C | 0.5477 | 0.1206 | 5.60E-06 |
| rs2375980 | C | G | 0.0952 | 0.0159 | 1.94E-09 |
| rs273702 | A | G | -0.127 | 0.027 | 2.52E-06 |
| rs13209117 | A | G | 0.0981 | 0.0186 | 1.27E-07 |
| rs41282644 | A | G | 0.1401 | 0.0303 | 3.74E-06 |
| rs6532374 | T | C | -0.1033 | 0.0226 | 4.61E-06 |
| rs71462292 | A | G | 0.0874 | 0.0196 | 7.80E-06 |
| rs1606534 | T | C | -0.0739 | 0.0167 | 9.51E-06 |
| rs10761731 | A | T | -0.0965 | 0.0161 | 2.12E-09 |
| rs117607213 | T | C | 0.259 | 0.0577 | 7.06E-06 |
| rs200751 | C | G | -0.0962 | 0.0213 | 6.41E-06 |

Table 39 Instrumental variables of IL-17

| SNP | EA | OA | Beat | SE | P |
| --- | --- | --- | --- | --- | --- |
| rs17282552 | T | C | -0.2026 | 0.0403 | 4.88E-07 |
| rs12735700 | T | G | -0.0943 | 0.0206 | 4.50E-06 |
| rs62191444 | T | G | -0.1105 | 0.0246 | 7.31E-06 |
| rs78296352 | T | G | 0.2949 | 0.0645 | 4.81E-06 |
| rs141312283 | T | G | -0.2748 | 0.0607 | 5.86E-06 |
| rs149738638 | T | C | -0.1553 | 0.0337 | 4.11E-06 |
| rs117556572 | T | C | -0.5256 | 0.1097 | 1.66E-06 |
| rs117029961 | A | G | 0.4566 | 0.1015 | 6.86E-06 |
| rs148562661 | C | G | 0.2161 | 0.0434 | 6.37E-07 |
| rs273702 | A | G | -0.1231 | 0.0279 | 9.97E-06 |
| rs184080173 | T | C | 0.236 | 0.0471 | 5.39E-07 |
| rs141398317 | A | C | 0.2294 | 0.0517 | 9.05E-06 |
| rs9568764 | C | G | 0.0825 | 0.018 | 4.68E-06 |
| rs187475560 | T | C | -0.2345 | 0.0517 | 5.84E-06 |
| rs17106604 | T | C | 0.1119 | 0.0225 | 6.23E-07 |
| rs11640734 | C | G | -0.115 | 0.024 | 1.61E-06 |
| rs145577605 | A | G | 0.1887 | 0.0425 | 9.05E-06 |
| rs1530455 | T | C | 0.1088 | 0.0173 | 3.29E-10 |
| rs78612928 | T | C | 0.0985 | 0.0221 | 8.25E-06 |
| rs57920188 | T | G | -0.0967 | 0.0217 | 8.25E-06 |

Table 40 Instrumental variables of IL-18

| SNP | EA | OA | Beat | SE | P |
| --- | --- | --- | --- | --- | --- |
| rs12420140 | A | G | -0.2479 | 0.0261 | 1.95E-21 |
| rs1656939 | A | T | 0.1043 | 0.0235 | 8.95E-06 |
| rs117266781 | T | C | 0.7051 | 0.1436 | 9.18E-07 |
| rs2729385 | A | G | 0.1163 | 0.026 | 8.01E-06 |
| rs4885797 | C | G | -0.1093 | 0.0238 | 4.39E-06 |
| rs78716465 | A | G | 0.3173 | 0.0679 | 2.98E-06 |
| rs17229943 | A | C | -0.3076 | 0.0463 | 3.06E-11 |
| rs13065560 | A | G | -0.1069 | 0.0239 | 7.54E-06 |
| rs7444013 | A | G | -0.5318 | 0.0955 | 2.59E-08 |
| rs143370787 | C | G | -0.3447 | 0.066 | 1.75E-07 |
| rs385076 | T | C | -0.2472 | 0.0247 | 1.56E-23 |
| rs1979967 | T | C | 0.14 | 0.0285 | 8.72E-07 |
| rs111611717 | C | G | -0.2172 | 0.0481 | 6.31E-06 |
| rs10414578 | T | C | -0.1817 | 0.0347 | 1.64E-07 |
| rs78623212 | T | C | 0.8322 | 0.1676 | 6.82E-07 |
| rs116383510 | A | C | -0.5412 | 0.1052 | 2.70E-07 |
| rs4482818 | A | G | 0.1233 | 0.0243 | 4.11E-07 |
| rs610473 | A | G | 0.1274 | 0.0242 | 1.43E-07 |
| rs62571473 | T | C | -0.1679 | 0.0376 | 8.18E-06 |
| rs1921283 | T | C | 0.1113 | 0.025 | 8.20E-06 |
| rs1852105 | T | C | 0.3 | 0.0659 | 5.27E-06 |
| rs76426122 | T | C | 0.1948 | 0.0432 | 6.42E-06 |

Table 41 Instrumental variables of MCP-1

| SNP | EA | OA | Beat | SE | P |
| --- | --- | --- | --- | --- | --- |
| rs147958317 | T | C | -0.2195 | 0.0488 | 6.87E-06 |
| rs10744620 | T | C | 0.0783 | 0.0161 | 1.12E-06 |
| rs186543342 | T | C | -0.1939 | 0.0428 | 5.78E-06 |
| rs2712431 | A | C | -0.0765 | 0.0171 | 8.23E-06 |
| rs12075 | A | G | 0.2186 | 0.0154 | 1.36E-45 |
| rs7632755 | A | G | 0.2984 | 0.0315 | 2.79E-21 |
| rs7517040 | A | G | -0.097 | 0.019 | 3.41E-07 |
| rs4645850 | A | C | -0.3771 | 0.0832 | 5.84E-06 |
| rs113089229 | A | C | 0.1873 | 0.0414 | 6.04E-06 |
| rs7033586 | A | G | -0.22 | 0.0467 | 2.43E-06 |
| rs111995966 | T | G | 0.1428 | 0.0309 | 3.79E-06 |
| rs10414279 | T | C | 0.0805 | 0.018 | 7.55E-06 |
| rs149470547 | T | C | 0.4241 | 0.0931 | 5.24E-06 |
| rs6694978 | T | C | -0.1643 | 0.0367 | 7.59E-06 |
| rs7197349 | A | G | 0.0971 | 0.0206 | 2.40E-06 |
| rs2288370 | T | C | -0.1036 | 0.0162 | 1.56E-10 |
| rs8020709 | A | G | -0.073 | 0.0164 | 8.15E-06 |
| rs12073356 | A | G | -0.1436 | 0.031 | 3.49E-06 |
| rs56212190 | T | C | 0.1799 | 0.0372 | 1.32E-06 |
| rs146522229 | T | C | -0.5942 | 0.1161 | 3.09E-07 |
| rs143815843 | A | G | -0.2049 | 0.0447 | 4.61E-06 |
| rs2036297 | A | G | 0.1182 | 0.016 | 1.30E-13 |
| rs9317045 | A | C | 0.1157 | 0.0235 | 8.43E-07 |

Table 42 Instrumental variables of MIP1**α**

| SNP | EA | OA | Beat | SE | P |
| --- | --- | --- | --- | --- | --- |
| rs7232268 | A | G | 0.2629 | 0.0595 | 9.90E-06 |
| rs113889446 | A | G | -0.1642 | 0.0362 | 5.72E-06 |
| rs184154340 | A | G | 0.3251 | 0.0689 | 2.40E-06 |
| rs55723389 | T | G | 0.3542 | 0.0776 | 5.02E-06 |
| rs138407013 | A | G | 0.3638 | 0.0806 | 6.39E-06 |
| rs139507938 | A | G | -0.873 | 0.1972 | 9.53E-06 |
| rs10835056 | T | G | 0.1154 | 0.0253 | 5.04E-06 |
| rs34771762 | A | G | 0.2336 | 0.0518 | 6.57E-06 |
| rs6900267 | A | C | -0.2472 | 0.0515 | 1.60E-06 |
| rs2682471 | T | G | -0.1134 | 0.0252 | 6.75E-06 |
| rs2341217 | C | G | 0.2482 | 0.0555 | 7.84E-06 |
| rs12690897 | A | G | 0.1215 | 0.026 | 3.07E-06 |
| rs2516841 | A | G | 0.1237 | 0.0278 | 8.34E-06 |
| rs57786342 | A | G | 0.139 | 0.0283 | 8.91E-07 |
| rs60198979 | A | G | -0.2154 | 0.0455 | 2.22E-06 |
| rs116615337 | A | G | 0.1286 | 0.0278 | 3.66E-06 |

Table 43 Instrumental variables of RANTES

| SNP | EA | OA | Beat | SE | P |
| --- | --- | --- | --- | --- | --- |
| rs147509526 | T | C | -0.3558 | 0.0715 | 6.57E-07 |
| rs62438851 | A | G | -0.1904 | 0.0413 | 4.01E-06 |
| rs9675798 | T | G | -0.2583 | 0.0552 | 2.89E-06 |
| rs79958656 | T | C | 0.1469 | 0.0325 | 5.98E-06 |
| rs10899562 | A | G | -0.1651 | 0.0365 | 6.13E-06 |
| rs72793342 | A | G | -0.1505 | 0.0307 | 9.08E-07 |
| rs112072646 | A | G | 0.4209 | 0.0859 | 9.62E-07 |
| rs2251660 | A | C | 0.1831 | 0.0356 | 2.69E-07 |
| rs2731672 | T | C | -0.1242 | 0.0272 | 4.83E-06 |
| rs144099278 | T | C | -0.4817 | 0.1081 | 8.38E-06 |
| rs7170339 | C | G | -0.4283 | 0.0904 | 2.19E-06 |
| rs7000423 | T | C | -0.1314 | 0.0252 | 1.85E-07 |
| rs4363007 | T | C | 0.1157 | 0.0259 | 7.71E-06 |
| rs7455952 | A | G | 0.1554 | 0.0342 | 5.62E-06 |
| rs74472919 | T | C | 0.3547 | 0.06 | 3.35E-09 |

Table 44 Instrumental variables of TNF-**α**

| SNP | EA | OA | Beat | SE | P |
| --- | --- | --- | --- | --- | --- |
| rs10834997 | A | G | -0.123 | 0.0256 | 1.53E-06 |
| rs79105320 | A | G | 0.5573 | 0.1177 | 2.21E-06 |
| rs111332265 | A | G | -0.3678 | 0.0745 | 7.91E-07 |
| rs8121916 | A | C | 0.1262 | 0.0277 | 5.19E-06 |
| rs7256693 | T | C | -0.1841 | 0.04 | 4.11E-06 |
| rs115669577 | A | G | 0.981 | 0.1994 | 8.63E-07 |

Table 45 Instrumental variables of Basal cell carcinoma

| SNP | Chr | Pos | EA | OA | EAF | Beta | SE | P |
| --- | --- | --- | --- | --- | --- | --- | --- | --- |
| rs71628384 | 1 | 165364477 | A | G | 0.240335 | 0.059686 | 0.012521 | 1.90E-06 |
| rs4363474 | 1 | 170382898 | C | T | 0.602246 | -0.04972 | 0.01111 | 7.60E-06 |
| rs905938 | 1 | 154991389 | C | T | 0.26559 | 0.057981 | 0.012129 | 1.70E-06 |
| rs2476601 | 1 | 114377568 | G | A | 0.898518 | 0.118074 | 0.018723 | 2.90E-10 |
| rs2062595 | 1 | 16075128 | T | C | 0.229382 | 0.058241 | 0.012978 | 7.20E-06 |
| rs730153 | 1 | 17749504 | A | G | 0.362745 | 0.182102 | 0.011074 | 9.20E-61 |
| rs801108 | 1 | 228989074 | G | C | 0.246169 | 0.214705 | 0.012 | 1.40E-71 |
| rs41271951 | 1 | 150737220 | G | A | 0.084459 | 0.110875 | 0.018686 | 3.00E-09 |
| rs79522206 | 2 | 7704860 | A | G | 0.026434 | 0.249044 | 0.030308 | 2.10E-16 |
| rs3087243 | 2 | 204738919 | A | G | 0.450314 | 0.095218 | 0.01087 | 2.00E-18 |
| rs6707137 | 2 | 88554351 | A | G | 0.061865 | -0.23274 | 0.024747 | 5.20E-21 |
| rs11686655 | 2 | 189764498 | G | A | 0.024218 | -0.18776 | 0.038297 | 9.50E-07 |
| rs76637130 | 2 | 201788479 | C | A | 0.016381 | 0.178902 | 0.039451 | 5.80E-06 |
| rs340797 | 2 | 16490831 | T | G | 0.104908 | -0.19858 | 0.019021 | 1.60E-25 |
| rs1800440 | 2 | 38298139 | C | T | 0.186205 | -0.0994 | 0.014324 | 3.90E-12 |
| rs2882274 | 2 | 5688981 | C | T | 0.515507 | 0.062349 | 0.011005 | 1.50E-08 |
| rs1371048 | 2 | 145753166 | T | G | 0.365178 | -0.05946 | 0.011426 | 2.00E-07 |
| rs78456138 | 2 | 163132346 | T | C | 0.023264 | 0.170942 | 0.033448 | 3.20E-07 |
| rs3769818 | 2 | 202151163 | G | A | 0.732167 | -0.17051 | 0.01186 | 7.20E-47 |
| rs3845780 | 2 | 37189296 | C | T | 0.475253 | 0.048612 | 0.010924 | 8.60E-06 |
| rs3755569 | 3 | 98449742 | T | C | 0.255345 | -0.06458 | 0.012625 | 3.10E-07 |
| rs8154 | 3 | 11596302 | C | T | 0.319462 | -0.05831 | 0.011729 | 6.60E-07 |
| rs2049218 | 3 | 188122978 | T | C | 0.451398 | 0.074451 | 0.010927 | 9.50E-12 |
| rs11708920 | 3 | 71517527 | T | G | 0.478948 | 0.122259 | 0.010892 | 3.10E-29 |
| rs191250697 | 3 | 17038914 | C | T | 0.052489 | -0.1195 | 0.025496 | 2.80E-06 |
| rs792829 | 3 | 99462647 | A | G | 0.629683 | 0.051141 | 0.011348 | 6.60E-06 |
| rs4682103 | 3 | 112055793 | G | A | 0.528636 | 0.062297 | 0.010907 | 1.10E-08 |
| rs9809389 | 3 | 28132490 | A | G | 0.408614 | -0.0523 | 0.011165 | 2.80E-06 |
| rs139871043 | 4 | 67630810 | C | T | 0.010478 | 0.216197 | 0.048818 | 9.50E-06 |
| rs10070702 | 5 | 141057221 | T | G | 0.630019 | 0.060248 | 0.011473 | 1.50E-07 |
| rs16867717 | 5 | 38773615 | C | T | 0.424534 | 0.050917 | 0.01098 | 3.50E-06 |
| rs11738948 | 5 | 44999799 | C | G | 0.209406 | 0.069227 | 0.013134 | 1.40E-07 |
| rs7705526 | 5 | 1285974 | A | C | 0.321483 | 0.082587 | 0.011755 | 2.10E-12 |
| rs251459 | 5 | 149199148 | A | G | 0.201175 | -0.06395 | 0.013825 | 3.70E-06 |
| rs16891982 | 5 | 33951693 | G | C | 0.97544 | 0.414833 | 0.041916 | 4.30E-23 |
| rs380286 | 5 | 1320247 | A | G | 0.436629 | -0.16071 | 0.011059 | 7.50E-48 |
| rs42905 | 5 | 67751221 | C | A | 0.544955 | -0.06164 | 0.010963 | 1.90E-08 |
| rs4958013 | 5 | 111535761 | G | A | 0.698124 | 0.058597 | 0.011965 | 9.70E-07 |
| rs112319612 | 5 | 157678735 | G | A | 0.051193 | -0.1163 | 0.025802 | 6.60E-06 |
| rs162298 | 6 | 167455629 | A | G | 0.248515 | 0.090165 | 0.012263 | 1.90E-13 |
| rs4945728 | 6 | 106175535 | C | T | 0.663451 | 0.05539 | 0.01164 | 1.90E-06 |
| rs950039 | 6 | 493976 | A | G | 0.718755 | 0.081685 | 0.01256 | 7.80E-11 |
| rs3778607 | 6 | 403799 | G | A | 0.547718 | 0.099021 | 0.010987 | 2.00E-19 |
| rs9392026 | 6 | 453415 | A | G | 0.591543 | -0.11022 | 0.010994 | 1.20E-23 |
| rs72928038 | 6 | 90976768 | A | G | 0.179729 | -0.1235 | 0.014643 | 3.30E-17 |
| rs9272451 | 6 | 32605501 | G | C | 0.386296 | -0.10671 | 0.011308 | 3.90E-21 |
| rs2294214 | 6 | 22056923 | C | A | 0.315761 | 0.098039 | 0.01148 | 1.30E-17 |
| rs28732208 | 6 | 32385119 | T | C | 0.16419 | 0.098889 | 0.014186 | 3.10E-12 |
| rs4708081 | 6 | 74485858 | A | G | 0.31529 | -0.06055 | 0.011821 | 3.00E-07 |
| rs12660291 | 6 | 45600454 | G | A | 0.14468 | 0.069173 | 0.015405 | 7.10E-06 |
| rs550193 | 6 | 150393890 | C | T | 0.630662 | 0.059026 | 0.01141 | 2.30E-07 |
| rs157934 | 7 | 130585492 | C | T | 0.304587 | -0.11276 | 0.012036 | 7.30E-21 |
| rs76128631 | 7 | 17628490 | T | G | 0.209867 | 0.073857 | 0.013221 | 2.30E-08 |
| rs2551761 | 7 | 135167947 | T | C | 0.604684 | -0.05018 | 0.011052 | 5.60E-06 |
| rs11770899 | 7 | 1979425 | T | C | 0.625937 | 0.060028 | 0.011419 | 1.50E-07 |
| rs12718245 | 7 | 50177706 | C | T | 0.421538 | -0.06472 | 0.011039 | 4.50E-09 |
| rs78227582 | 7 | 101393723 | T | G | 0.247444 | -0.10922 | 0.013016 | 4.80E-17 |
| rs117744081 | 7 | 29132279 | G | A | 0.032237 | -0.24634 | 0.034092 | 5.00E-13 |
| rs834603 | 7 | 47447921 | G | A | 0.493218 | -0.06182 | 0.010875 | 1.30E-08 |
| rs28884287 | 8 | 101047448 | T | C | 0.154482 | -0.19328 | 0.015971 | 1.00E-33 |
| rs2241260 | 8 | 22875985 | G | A | 0.312488 | -0.06731 | 0.012015 | 2.10E-08 |
| rs10216564 | 8 | 77475130 | C | T | 0.06347 | -0.32074 | 0.025289 | 7.30E-37 |
| rs55942834 | 8 | 81411215 | G | A | 0.193106 | -0.11812 | 0.014248 | 1.10E-16 |
| rs4733812 | 8 | 128999640 | A | G | 0.343154 | 0.051164 | 0.011373 | 6.80E-06 |
| rs140826556 | 8 | 17942332 | G | A | 0.037461 | 0.123528 | 0.027145 | 5.30E-06 |
| rs3818625 | 9 | 106856452 | A | G | 0.550882 | 0.065426 | 0.010939 | 2.20E-09 |
| rs2026804 | 9 | 16800341 | C | A | 0.762818 | 0.06852 | 0.013003 | 1.40E-07 |
| rs7027950 | 9 | 22048391 | T | C | 0.474682 | -0.12078 | 0.010926 | 2.10E-28 |
| rs10962474 | 9 | 16529554 | T | C | 0.053092 | -0.25755 | 0.026939 | 1.20E-21 |
| rs9286380 | 9 | 92512212 | T | C | 0.460116 | 0.050683 | 0.010862 | 3.10E-06 |
| rs7867194 | 9 | 681862 | A | C | 0.441163 | 0.058214 | 0.011033 | 1.30E-07 |
| rs2153271 | 9 | 16864521 | T | C | 0.612466 | 0.07875 | 0.011221 | 2.30E-12 |
| rs11258481 | 10 | 13668787 | T | A | 0.434616 | -0.05103 | 0.010994 | 3.50E-06 |
| rs1782648 | 10 | 81060829 | A | G | 0.368223 | -0.05686 | 0.011407 | 6.20E-07 |
| rs7086072 | 10 | 64417289 | T | C | 0.73875 | 0.065146 | 0.01251 | 1.90E-07 |
| rs74623270 | 10 | 8995292 | A | G | 0.115686 | -0.25386 | 0.018593 | 1.90E-42 |
| rs61874089 | 10 | 103506911 | A | G | 0.0519 | -0.11856 | 0.02564 | 3.80E-06 |
| rs2387397 | 10 | 6390192 | C | G | 0.783097 | -0.06181 | 0.013066 | 2.20E-06 |
| rs10742797 | 11 | 47240490 | T | A | 0.241145 | -0.06117 | 0.012933 | 2.20E-06 |
| rs12576996 | 11 | 65580638 | G | T | 0.244402 | -0.08332 | 0.012995 | 1.40E-10 |
| rs10890839 | 11 | 108306236 | A | C | 0.409757 | 0.049535 | 0.010994 | 6.60E-06 |
| rs11020920 | 11 | 94447905 | C | T | 0.302445 | -0.06422 | 0.011965 | 8.00E-08 |
| rs663743 | 11 | 64107735 | A | G | 0.340422 | 0.068596 | 0.011374 | 1.60E-09 |
| rs1126809 | 11 | 89017961 | A | G | 0.303865 | 0.13852 | 0.01157 | 5.00E-33 |
| rs73114127 | 12 | 50205502 | G | A | 0.311272 | 0.056727 | 0.01167 | 1.20E-06 |
| rs35511327 | 12 | 24747872 | C | T | 0.215745 | 0.060893 | 0.012991 | 2.80E-06 |
| rs3213737 | 12 | 96379806 | A | G | 0.576638 | -0.05053 | 0.011 | 4.40E-06 |
| rs10870469 | 12 | 133142686 | C | T | 0.441456 | 0.048693 | 0.010949 | 8.70E-06 |
| rs9668178 | 12 | 26421434 | A | T | 0.240446 | 0.065228 | 0.012553 | 2.00E-07 |
| rs206951 | 12 | 120823354 | C | T | 0.148064 | -0.07079 | 0.015683 | 6.40E-06 |
| rs11170164 | 12 | 52913668 | T | C | 0.078136 | 0.195154 | 0.018772 | 2.60E-25 |
| rs2094502 | 13 | 99803836 | T | G | 0.28012 | 0.058781 | 0.011999 | 9.60E-07 |
| rs1460816 | 13 | 32928408 | A | G | 0.527744 | -0.05151 | 0.010853 | 2.10E-06 |
| rs2208598 | 14 | 21237378 | A | G | 0.48223 | 0.049708 | 0.011055 | 6.90E-06 |
| rs7152892 | 14 | 51941998 | A | G | 0.18455 | 0.068716 | 0.013822 | 6.60E-07 |
| rs56199318 | 14 | 25376150 | T | A | 0.098224 | 0.080507 | 0.017961 | 7.40E-06 |
| rs909223 | 14 | 75909897 | G | A | 0.444782 | -0.06471 | 0.010966 | 3.60E-09 |
| rs79133042 | 14 | 57229959 | G | A | 0.080685 | -0.10096 | 0.020736 | 1.10E-06 |
| rs12436874 | 14 | 58435873 | C | A | 0.238928 | 0.05706 | 0.012621 | 6.20E-06 |
| rs117886461 | 15 | 28230378 | A | G | 0.012134 | 0.243603 | 0.044862 | 5.60E-08 |
| rs758129 | 15 | 89900887 | A | G | 0.356635 | 0.052638 | 0.011288 | 3.10E-06 |
| rs11853333 | 15 | 40742338 | A | G | 0.355414 | -0.05797 | 0.011624 | 6.10E-07 |
| rs11633714 | 15 | 50142029 | A | G | 0.269854 | -0.05801 | 0.012365 | 2.70E-06 |
| rs35749174 | 16 | 89716493 | A | G | 0.099785 | 0.28426 | 0.016368 | 1.50E-67 |
| rs74415461 | 16 | 89990843 | T | C | 0.084979 | 0.134057 | 0.018559 | 5.10E-13 |
| rs144242147 | 16 | 68937606 | T | C | 0.154524 | -0.07333 | 0.015477 | 2.20E-06 |
| rs3931016 | 16 | 11415741 | C | T | 0.549193 | -0.05793 | 0.010901 | 1.10E-07 |
| rs117133873 | 16 | 50280364 | A | C | 0.057658 | -0.11021 | 0.024469 | 6.70E-06 |
| rs142222325 | 16 | 89932167 | C | G | 0.014774 | -0.2308 | 0.049879 | 3.70E-06 |
| rs854807 | 17 | 18062443 | A | G | 0.426369 | 0.061858 | 0.010947 | 1.60E-08 |
| rs2279396 | 17 | 80536908 | C | A | 0.635416 | 0.059038 | 0.011521 | 3.00E-07 |
| rs78378222 | 17 | 7571752 | G | T | 0.011228 | 0.450949 | 0.042069 | 8.30E-27 |
| rs7207781 | 17 | 59331307 | C | A | 0.068074 | 0.093396 | 0.02079 | 7.00E-06 |
| rs12943034 | 17 | 74049418 | T | C | 0.011457 | -0.27014 | 0.057387 | 2.50E-06 |
| rs4808076 | 19 | 17395401 | T | C | 0.294471 | 0.061023 | 0.011767 | 2.10E-07 |
| rs10415576 | 19 | 50164390 | C | T | 0.378127 | 0.05469 | 0.011199 | 1.00E-06 |
| rs74178437 | 19 | 4056860 | A | G | 0.702785 | -0.05841 | 0.011854 | 8.30E-07 |
| rs11669443 | 19 | 1109215 | A | G | 0.23506 | 0.075747 | 0.012578 | 1.70E-09 |
| rs6513599 | 20 | 37937661 | T | C | 0.432732 | 0.06035 | 0.011063 | 4.90E-08 |
| rs13043023 | 20 | 2182626 | C | T | 0.096673 | -0.16324 | 0.019605 | 8.30E-17 |
| rs6028848 | 20 | 38706873 | T | G | 0.134494 | 0.075379 | 0.015545 | 1.20E-06 |
| rs35640778 | 20 | 62321128 | A | G | 0.021814 | -0.19509 | 0.040533 | 1.50E-06 |
| rs214803 | 20 | 2290333 | A | C | 0.820407 | -0.21617 | 0.013276 | 1.30E-59 |
| rs6059655 | 20 | 32665748 | G | A | 0.899312 | -0.23676 | 0.016756 | 2.50E-45 |
| rs11908416 | 20 | 49392745 | A | G | 0.240924 | -0.10758 | 0.013108 | 2.30E-16 |
| rs2849699 | 21 | 43089086 | G | C | 0.317945 | -0.10549 | 0.011938 | 9.90E-19 |
| rs42936 | 22 | 30962878 | A | G | 0.660584 | 0.061458 | 0.01185 | 2.10E-07 |

Table 46 Instrumental variables of Bladder cancer

| SNP | Chr | Pos | EA | OA | EAF | Beta | SE | P |
| --- | --- | --- | --- | --- | --- | --- | --- | --- |
| rs771137 | 1 | 34614520 | A | C | 0.868627 | -0.00092 | 0.0002 | 3.80E-06 |
| rs12752338 | 1 | 2.41E+08 | C | T | 0.028468 | 0.001836 | 0.000406 | 6.20E-06 |
| rs7584610 | 2 | 1.96E+08 | A | G | 0.657618 | -0.00064 | 0.000143 | 7.60E-06 |
| rs71439172 | 2 | 25584162 | A | G | 0.146744 | -0.0009 | 0.000194 | 3.70E-06 |
| rs150222295 | 2 | 77391468 | A | C | 0.012147 | 0.002808 | 0.00062 | 5.90E-06 |
| rs6705922 | 2 | 2.36E+08 | A | G | 0.321377 | -0.0007 | 0.000147 | 2.30E-06 |
| rs79455462 | 4 | 62868612 | T | A | 0.013071 | 0.002757 | 0.000615 | 7.30E-06 |
| rs150797373 | 4 | 8266421 | A | C | 0.013936 | 0.003081 | 0.000611 | 4.70E-07 |
| rs11727393 | 4 | 57480099 | C | G | 0.014489 | 0.002695 | 0.000599 | 6.90E-06 |
| rs1132787 | 4 | 1.45E+08 | T | C | 0.305271 | -0.00065 | 0.000146 | 8.60E-06 |
| rs146190477 | 4 | 90032521 | T | C | 0.043295 | 0.002032 | 0.000344 | 3.30E-09 |
| rs2736103 | 5 | 1300401 | C | T | 0.418317 | 0.000657 | 0.000138 | 2.00E-06 |
| rs11744794 | 5 | 1.24E+08 | A | T | 0.317666 | 0.000731 | 0.000147 | 6.60E-07 |
| rs7722450 | 5 | 31077122 | A | G | 0.036075 | 0.001699 | 0.000368 | 3.90E-06 |
| rs146820664 | 5 | 91417975 | G | C | 0.015253 | 0.002733 | 0.000554 | 8.20E-07 |
| rs62408223 | 6 | 90949196 | G | A | 0.169148 | 0.000859 | 0.00018 | 1.90E-06 |
| rs9402548 | 6 | 1.34E+08 | T | G | 0.323864 | 0.000647 | 0.000146 | 9.00E-06 |
| rs392450 | 8 | 60024449 | A | G | 0.082948 | 0.001234 | 0.000247 | 5.60E-07 |
| rs10094872 | 8 | 1.29E+08 | T | A | 0.364032 | 0.000706 | 0.000141 | 5.90E-07 |
| rs34635647 | 8 | 1.44E+08 | G | A | 0.436775 | 0.000618 | 0.000137 | 6.20E-06 |
| rs146462590 | 8 | 1.26E+08 | G | T | 0.01062 | 0.003215 | 0.000675 | 1.90E-06 |
| rs144980373 | 8 | 16367131 | C | G | 0.023523 | 0.002054 | 0.000462 | 8.70E-06 |
| rs143739252 | 9 | 1.07E+08 | C | G | 0.016852 | 0.002433 | 0.000545 | 8.00E-06 |
| rs7038689 | 9 | 1709649 | C | A | 0.458891 | -0.00063 | 0.000136 | 3.20E-06 |
| rs11793757 | 9 | 1.33E+08 | A | G | 0.117217 | 0.000981 | 0.00021 | 3.10E-06 |
| rs184321190 | 10 | 17019503 | C | T | 0.014168 | 0.002795 | 0.000599 | 3.10E-06 |
| rs74131143 | 10 | 51058643 | A | G | 0.038728 | 0.001644 | 0.000351 | 2.80E-06 |
| rs75021825 | 11 | 39864108 | T | C | 0.102052 | -0.00103 | 0.00023 | 7.40E-06 |
| rs71227149 | 12 | 90852 | A | G | 0.030982 | 0.002864 | 0.000597 | 1.60E-06 |
| rs192242308 | 13 | 88165920 | T | C | 0.012816 | 0.002802 | 0.000625 | 7.40E-06 |
| rs11158808 | 14 | 69933892 | T | C | 0.134427 | -0.00102 | 0.000198 | 3.00E-07 |
| rs147813917 | 15 | 59246079 | A | G | 0.016265 | 0.002756 | 0.000553 | 6.20E-07 |
| rs3744753 | 17 | 907907 | A | G | 0.043617 | 0.001666 | 0.000334 | 6.30E-07 |
| rs35515294 | 20 | 10358090 | T | C | 0.026221 | 0.002107 | 0.000426 | 7.40E-07 |
| rs6078008 | 20 | 10995830 | A | T | 0.295149 | 0.000687 | 0.000149 | 3.70E-06 |
| rs56297045 | 22 | 39350684 | A | G | 0.257617 | 0.000732 | 0.000156 | 2.50E-06 |
| rs74903734 | 22 | 25799984 | G | A | 0.013371 | 0.003273 | 0.000589 | 2.70E-08 |

Table 47 Instrumental variables of Brain cancer

| SNP | Chr | Pos | EA | OA | EAF | Beta | SE | P |
| --- | --- | --- | --- | --- | --- | --- | --- | --- |
| rs76352481 | 1 | 47171859 | C | T | 0.031605 | 0.001263 | 0.000277 | 5.30E-06 |
| rs79310779 | 1 | 2.42E+08 | T | C | 0.050457 | 0.001059 | 0.000228 | 3.30E-06 |
| rs3003385 | 1 | 17057002 | C | T | 0.93957 | -0.00109 | 0.000239 | 5.60E-06 |
| rs145646449 | 1 | 98877107 | C | T | 0.025223 | 0.001398 | 0.000301 | 3.40E-06 |
| rs12742104 | 1 | 1.65E+08 | T | C | 0.046863 | -0.00099 | 0.000223 | 8.30E-06 |
| rs10174285 | 2 | 50965496 | G | T | 0.023115 | 0.001474 | 0.00032 | 4.20E-06 |
| rs76737990 | 2 | 1.61E+08 | C | G | 0.021216 | 0.001443 | 0.000324 | 8.30E-06 |
| rs529474930 | 3 | 46347485 | G | T | 0.030808 | 0.001285 | 0.000278 | 3.80E-06 |
| rs2309396 | 4 | 1.82E+08 | A | G | 0.546308 | 0.000442 | 9.49E-05 | 3.20E-06 |
| rs4535339 | 4 | 38689228 | G | A | 0.212118 | -0.00062 | 0.000115 | 7.80E-08 |
| rs7705526 | 5 | 1285974 | A | C | 0.325452 | 0.000769 | 0.000101 | 2.70E-14 |
| rs6895243 | 5 | 1.21E+08 | G | A | 0.632756 | -0.00047 | 9.70E-05 | 1.60E-06 |
| rs80318206 | 5 | 1.05E+08 | T | C | 0.036535 | 0.00117 | 0.000258 | 5.80E-06 |
| rs74626051 | 5 | 27810069 | G | A | 0.025164 | 0.001447 | 0.000298 | 1.20E-06 |
| rs117901418 | 6 | 1.1E+08 | C | A | 0.022108 | 0.001463 | 0.000322 | 5.50E-06 |
| rs112373346 | 7 | 18112325 | T | C | 0.062781 | 0.000913 | 0.000194 | 2.60E-06 |
| rs77126132 | 7 | 54966738 | A | G | 0.093327 | 0.000784 | 0.000162 | 1.20E-06 |
| rs79044914 | 7 | 39739457 | T | C | 0.025818 | 0.001374 | 0.000294 | 3.00E-06 |
| rs116985138 | 9 | 30774934 | G | C | 0.022072 | 0.001508 | 0.000327 | 4.00E-06 |
| rs615552 | 9 | 22026077 | C | T | 0.436756 | 0.000677 | 9.42E-05 | 6.70E-13 |
| rs4579610 | 9 | 90041160 | T | C | 0.767502 | 0.000532 | 0.000112 | 2.00E-06 |
| rs147266134 | 10 | 89094377 | T | C | 0.037112 | 0.001348 | 0.000274 | 8.70E-07 |
| rs4311994 | 10 | 1.13E+08 | T | C | 0.157323 | 0.000635 | 0.000129 | 7.70E-07 |
| rs4944742 | 11 | 87330267 | A | G | 0.661933 | -0.00046 | 9.93E-05 | 3.30E-06 |
| rs11605800 | 11 | 76519839 | T | C | 0.033766 | 0.001167 | 0.000263 | 8.80E-06 |
| rs111377985 | 12 | 50652543 | A | G | 0.043493 | 0.001053 | 0.00023 | 4.80E-06 |
| rs12590369 | 14 | 85244080 | G | C | 0.269306 | 0.000507 | 0.000105 | 1.50E-06 |
| rs11850404 | 14 | 29022089 | T | C | 0.43892 | -0.00046 | 9.45E-05 | 1.20E-06 |
| rs2201346 | 15 | 57765761 | C | T | 0.306113 | 0.000463 | 0.000102 | 6.30E-06 |
| rs4932441 | 15 | 89433907 | C | G | 0.054121 | 0.000932 | 0.000206 | 6.40E-06 |
| rs67427010 | 16 | 8169608 | T | G | 0.055993 | 0.000923 | 0.000204 | 6.10E-06 |
| rs143094271 | 17 | 7463102 | A | G | 0.020873 | 0.001569 | 0.000328 | 1.80E-06 |
| rs9962338 | 18 | 61175079 | C | A | 0.197023 | 0.000545 | 0.000117 | 3.40E-06 |
| rs140586437 | 20 | 13881901 | G | A | 0.021628 | 0.001618 | 0.000328 | 7.80E-07 |
| rs6010986 | 20 | 62275844 | C | T | 0.767366 | 0.000539 | 0.000111 | 1.20E-06 |

Table 48 Instrumental variables of Breast cancer

| SNP | Chr | Pos | EA | OA | EAF | Beta | SE | P |
| --- | --- | --- | --- | --- | --- | --- | --- | --- |
| rs6671962 | 1 | 51259645 | G | A | 0.423796 | 0.001867 | 0.000361 | 2.27E-07 |
| rs190844357 | 1 | 2.39E+08 | T | G | 0.006616 | -0.01207 | 0.002336 | 2.40E-07 |
| rs11208611 | 1 | 65684425 | T | C | 0.330028 | 0.001889 | 0.000381 | 7.02E-07 |
| rs11249433 | 1 | 1.21E+08 | G | A | 0.416014 | 0.002131 | 0.000361 | 3.53E-09 |
| rs10495109 | 1 | 2.19E+08 | C | T | 0.012783 | 0.007073 | 0.001585 | 8.12E-06 |
| rs76659818 | 1 | 1.89E+08 | T | C | 0.011458 | 0.008273 | 0.001683 | 8.80E-07 |
| rs7605422 | 2 | 7461307 | G | A | 0.968329 | -0.00501 | 0.001013 | 7.47E-07 |
| rs9967727 | 2 | 2.14E+08 | G | C | 0.366084 | 0.001813 | 0.00037 | 9.50E-07 |
| rs74423580 | 2 | 2.3E+08 | T | C | 0.056582 | 0.003476 | 0.000781 | 8.61E-06 |
| rs35380972 | 2 | 1.72E+08 | C | A | 0.367864 | -0.00178 | 0.000369 | 1.36E-06 |
| rs114033225 | 2 | 1.3E+08 | A | G | 0.015653 | 0.006692 | 0.001432 | 2.99E-06 |
| rs4442975 | 2 | 2.18E+08 | T | G | 0.511994 | -0.00256 | 0.000357 | 7.86E-13 |
| rs11715126 | 3 | 27401247 | A | G | 0.418671 | -0.00269 | 0.000361 | 8.90E-14 |
| rs2410884 | 3 | 1.51E+08 | A | C | 0.72303 | 0.001784 | 0.000397 | 6.94E-06 |
| rs11730826 | 4 | 1.88E+08 | T | A | 0.45391 | 0.001861 | 0.00036 | 2.45E-07 |
| rs13141889 | 4 | 1.06E+08 | C | A | 0.42289 | 0.00163 | 0.000361 | 6.44E-06 |
| rs2728141 | 4 | 1.78E+08 | T | A | 0.566553 | 0.001656 | 0.000362 | 4.66E-06 |
| rs148356273 | 4 | 88739101 | A | G | 0.037005 | -0.00425 | 0.000951 | 7.77E-06 |
| rs7714232 | 5 | 56011357 | T | A | 0.162047 | 0.004062 | 0.000484 | 4.57E-17 |
| rs115459895 | 5 | 1.41E+08 | T | C | 0.028637 | 0.004819 | 0.001076 | 7.53E-06 |
| rs2042226 | 5 | 1.7E+08 | G | C | 0.185687 | -0.00204 | 0.000459 | 9.13E-06 |
| rs2736109 | 5 | 1296759 | T | C | 0.409049 | -0.00162 | 0.000363 | 7.91E-06 |
| rs10941679 | 5 | 44706498 | G | A | 0.254817 | 0.002665 | 0.000412 | 1.04E-10 |
| rs6913578 | 6 | 1.52E+08 | C | A | 0.322714 | 0.002197 | 0.00038 | 7.65E-09 |
| rs1887315 | 6 | 1.12E+08 | G | C | 0.128976 | -0.00239 | 0.000533 | 7.57E-06 |
| rs34415847 | 6 | 77273792 | A | G | 0.126187 | 0.002605 | 0.000553 | 2.51E-06 |
| rs76196285 | 6 | 1.36E+08 | C | T | 0.024297 | 0.005748 | 0.001154 | 6.34E-07 |
| rs73233093 | 7 | 1.27E+08 | T | C | 0.042036 | -0.00394 | 0.000885 | 8.66E-06 |
| rs10953517 | 7 | 1.06E+08 | A | G | 0.560949 | 0.00166 | 0.000359 | 3.82E-06 |
| rs17437810 | 7 | 27281792 | A | G | 0.019603 | 0.00639 | 0.001339 | 1.81E-06 |
| rs80275972 | 8 | 1.41E+08 | C | G | 0.080695 | -0.0033 | 0.000685 | 1.50E-06 |
| rs61044379 | 8 | 1.28E+08 | T | C | 0.312129 | 0.002766 | 0.000384 | 5.63E-13 |
| rs548980 | 9 | 1.11E+08 | T | C | 0.618224 | 0.002003 | 0.000366 | 4.58E-08 |
| rs10978911 | 9 | 1.1E+08 | C | G | 0.122744 | 0.003309 | 0.000544 | 1.14E-09 |
| rs72698025 | 9 | 4475352 | T | G | 0.035549 | -0.00443 | 0.000973 | 5.28E-06 |
| rs2936870 | 10 | 1.23E+08 | C | T | 0.604938 | -0.00524 | 0.000364 | 5.18E-47 |
| rs7903793 | 10 | 80887212 | T | C | 0.157349 | 0.002766 | 0.000489 | 1.56E-08 |
| rs12098603 | 10 | 21709408 | T | C | 0.200091 | 0.002 | 0.000445 | 6.94E-06 |
| rs17434960 | 10 | 9108558 | T | C | 0.194201 | -0.00199 | 0.000449 | 9.43E-06 |
| rs61886305 | 10 | 95902053 | A | C | 0.173162 | -0.00216 | 0.000472 | 4.74E-06 |
| rs2393886 | 10 | 64258343 | T | C | 0.465319 | -0.00208 | 0.000358 | 6.52E-09 |
| rs7913694 | 10 | 1.23E+08 | G | A | 0.297949 | -0.00205 | 0.000402 | 3.28E-07 |
| rs111718303 | 11 | 1.35E+08 | A | G | 0.09748 | -0.00299 | 0.000602 | 7.10E-07 |
| rs657686 | 11 | 69332670 | G | A | 0.120303 | 0.004812 | 0.000546 | 1.20E-18 |
| rs61938093 | 12 | 96026737 | T | C | 0.294909 | -0.00219 | 0.000391 | 2.03E-08 |
| rs2133317 | 12 | 1.16E+08 | G | C | 0.384799 | -0.0017 | 0.000366 | 3.55E-06 |
| rs812020 | 12 | 28151609 | C | A | 0.258617 | -0.00184 | 0.00041 | 6.97E-06 |
| rs73127134 | 12 | 67012308 | T | A | 0.178203 | -0.0021 | 0.000466 | 6.46E-06 |
| rs2588830 | 14 | 68609319 | G | A | 0.839002 | -0.00242 | 0.000486 | 6.22E-07 |
| rs17212275 | 14 | 26882106 | A | G | 0.215994 | -0.00197 | 0.000432 | 5.18E-06 |
| rs1904316 | 15 | 49708710 | C | A | 0.472553 | -0.00167 | 0.000357 | 2.78E-06 |
| rs873994 | 16 | 80651660 | C | T | 0.218528 | 0.002073 | 0.00043 | 1.43E-06 |
| rs76393613 | 16 | 25389811 | C | T | 0.048818 | 0.004144 | 0.000905 | 4.65E-06 |
| rs8063057 | 16 | 53812433 | C | T | 0.395047 | -0.00217 | 0.000364 | 2.43E-09 |
| rs4784227 | 16 | 52599188 | T | C | 0.239161 | 0.005188 | 0.000416 | 1.29E-35 |
| rs144922787 | 17 | 8959616 | C | T | 0.175782 | 0.002252 | 0.000475 | 2.11E-06 |
| rs4940668 | 18 | 56032231 | G | A | 0.298471 | -0.00183 | 0.000389 | 2.38E-06 |
| rs2105063 | 20 | 62690683 | T | C | 0.562306 | 0.001739 | 0.000358 | 1.22E-06 |
| rs111719757 | 21 | 16650916 | A | T | 0.060817 | -0.00372 | 0.000756 | 8.73E-07 |
| rs76451560 | 22 | 32729100 | T | C | 0.196301 | 0.002028 | 0.000453 | 7.71E-06 |
| rs17002034 | 22 | 40996367 | T | G | 0.097813 | 0.003443 | 0.000598 | 8.65E-09 |

Table 49 Instrumental variables of Cancer of urinary tract

| SNP | Chr | Pos | EA | OA | EAF | Beta | SE | P |
| --- | --- | --- | --- | --- | --- | --- | --- | --- |
| rs114267184 | 1 | 219928379 | G | A | 0.01256 | 0.004049 | 0.000829 | 1.04E-06 |
| rs10800681 | 1 | 200197763 | T | C | 0.678993 | -0.00081 | 0.000179 | 6.48E-06 |
| rs192874879 | 1 | 19594561 | C | G | 0.015963 | 0.003401 | 0.000705 | 1.40E-06 |
| rs12752338 | 1 | 241004429 | C | T | 0.027885 | 0.002319 | 0.000508 | 5.02E-06 |
| rs16864797 | 3 | 152733689 | T | C | 0.081279 | 0.001429 | 0.000308 | 3.48E-06 |
| rs116441733 | 4 | 178842279 | C | T | 0.030296 | -0.00225 | 0.000497 | 6.01E-06 |
| rs78270488 | 4 | 112438016 | T | G | 0.06068 | 0.001649 | 0.000352 | 2.75E-06 |
| rs190365284 | 4 | 160963917 | G | A | 0.008217 | 0.004356 | 0.000975 | 7.92E-06 |
| rs6837394 | 4 | 83651419 | C | T | 0.083449 | -0.00145 | 0.00031 | 2.65E-06 |
| rs554982201 | 4 | 145054065 | T | C | 0.019078 | 0.003126 | 0.000649 | 1.46E-06 |
| rs115825154 | 5 | 130502694 | A | G | 0.009527 | 0.004024 | 0.000883 | 5.18E-06 |
| rs139433573 | 5 | 11273582 | A | C | 0.024146 | 0.002488 | 0.000557 | 7.80E-06 |
| rs140722937 | 5 | 19120049 | C | A | 0.014188 | 0.003387 | 0.000756 | 7.45E-06 |
| rs377413060 | 5 | 1061371 | G | A | 0.850084 | -0.00114 | 0.000253 | 7.00E-06 |
| rs144408414 | 6 | 80099549 | G | T | 0.014834 | 0.003574 | 0.000723 | 7.66E-07 |
| rs10950880 | 7 | 21900188 | T | G | 0.837903 | -0.00105 | 0.000231 | 5.28E-06 |
| rs10952479 | 7 | 154126501 | G | T | 0.810319 | -0.00099 | 0.000217 | 5.03E-06 |
| rs139898132 | 8 | 128182520 | A | G | 0.027952 | 0.002343 | 0.000524 | 7.74E-06 |
| rs10097591 | 8 | 95067894 | A | G | 0.145951 | -0.00106 | 0.000237 | 7.04E-06 |
| rs12544299 | 8 | 130238601 | T | C | 0.055623 | 0.001678 | 0.000365 | 4.24E-06 |
| rs992139 | 10 | 91932058 | A | G | 0.416567 | 0.000756 | 0.00017 | 8.63E-06 |
| rs77548406 | 11 | 45556490 | T | C | 0.014953 | 0.003374 | 0.000689 | 9.81E-07 |
| rs4910009 | 11 | 11487061 | A | G | 0.069461 | 0.001614 | 0.000331 | 1.10E-06 |
| rs188856241 | 12 | 51654157 | T | G | 0.01314 | 0.003703 | 0.000748 | 7.38E-07 |
| rs74918612 | 12 | 96149571 | A | C | 0.076915 | 0.001438 | 0.000322 | 7.93E-06 |
| rs116159570 | 13 | 109869974 | T | C | 0.156268 | 0.001069 | 0.000238 | 7.16E-06 |
| rs113308389 | 14 | 91583026 | C | A | 0.029253 | 0.002248 | 0.000497 | 6.17E-06 |
| rs147862342 | 18 | 38308866 | A | T | 0.082561 | -0.00141 | 0.000306 | 4.00E-06 |

Table 50 Instrumental variables of Cervical cancer

| SNP | Chr | Pos | EA | OA | EAF | Beta | SE | P |
| --- | --- | --- | --- | --- | --- | --- | --- | --- |
| rs56382923 | 1 | 118702036 | C | T | 0.045865 | 0.001986 | 0.000404 | 9.00E-07 |
| rs116594665 | 2 | 36249108 | C | T | 0.062457 | -0.00157 | 0.000351 | 7.90E-06 |
| rs77542369 | 2 | 218466845 | C | T | 0.028009 | 0.002461 | 0.000528 | 3.10E-06 |
| rs7560360 | 2 | 172036474 | G | A | 0.386412 | -0.00077 | 0.000174 | 9.50E-06 |
| rs2727602 | 5 | 11539622 | G | A | 0.69062 | -0.00085 | 0.000184 | 3.50E-06 |
| rs113899620 | 5 | 107027036 | C | T | 0.122543 | 0.001193 | 0.000261 | 5.10E-06 |
| rs13185925 | 5 | 43888838 | C | G | 0.035763 | 0.002048 | 0.000458 | 7.60E-06 |
| rs2067017 | 6 | 36343500 | T | A | 0.298696 | 0.000926 | 0.000199 | 3.20E-06 |
| rs9402901 | 6 | 137841657 | A | G | 0.103494 | 0.001403 | 0.000281 | 5.90E-07 |
| rs2610938 | 8 | 21155404 | G | A | 0.120795 | 0.001165 | 0.000261 | 7.80E-06 |
| rs117860885 | 9 | 110146423 | C | T | 0.033262 | -0.00215 | 0.000469 | 4.70E-06 |
| rs375516111 | 9 | 39803976 | C | A | 0.079351 | 0.002613 | 0.000509 | 2.80E-07 |
| rs12808121 | 11 | 129055150 | T | C | 0.035526 | 0.002057 | 0.000465 | 9.80E-06 |
| rs6483107 | 11 | 91002951 | A | G | 0.977149 | -0.00304 | 0.000564 | 6.60E-08 |
| rs680484 | 13 | 110881085 | T | G | 0.400706 | -0.00079 | 0.000175 | 6.90E-06 |
| rs12184592 | 13 | 63405679 | C | T | 0.047414 | 0.002064 | 0.000398 | 2.20E-07 |
| rs58833197 | 14 | 98139896 | G | A | 0.123332 | 0.001295 | 0.000274 | 2.20E-06 |
| rs1508959 | 17 | 51675304 | A | T | 0.237119 | 0.000953 | 0.000199 | 1.70E-06 |
| rs77714526 | 17 | 8489919 | A | G | 0.033917 | 0.002095 | 0.000466 | 6.90E-06 |
| rs568710 | 20 | 5781209 | G | A | 0.88974 | -0.00123 | 0.000269 | 4.80E-06 |
| rs11913612 | 22 | 27237856 | A | G | 0.134178 | 0.001152 | 0.000248 | 3.30E-06 |
| rs79887800 | 22 | 39288854 | A | G | 0.025025 | 0.00264 | 0.000553 | 1.80E-06 |

Table 51 Instrumental variables of Colorectal cancer

| SNP | Chr | Pos | EA | OA | EAF | Beta | SE | P |
| --- | --- | --- | --- | --- | --- | --- | --- | --- |
| rs192478401 | 1 | 67402073 | T | A | 0.011155 | -0.00618 | 0.001391 | 9.10E-06 |
| rs12135286 | 1 | 222218761 | T | C | 0.193449 | 0.002033 | 0.000354 | 9.40E-09 |
| rs28798069 | 1 | 224438833 | G | A | 0.50561 | -0.00127 | 0.000281 | 5.70E-06 |
| rs4546885 | 1 | 183025555 | C | G | 0.410924 | -0.00147 | 0.000284 | 2.30E-07 |
| rs3731861 | 2 | 219191256 | C | T | 0.378262 | -0.00128 | 0.000289 | 8.60E-06 |
| rs72826124 | 2 | 104916571 | A | G | 0.068344 | 0.002707 | 0.000556 | 1.10E-06 |
| rs116113599 | 2 | 20722889 | A | G | 0.027842 | -0.00434 | 0.00092 | 2.30E-06 |
| rs6705128 | 2 | 199848803 | A | T | 0.326054 | 0.001414 | 0.000298 | 2.10E-06 |
| rs114717436 | 3 | 28450527 | G | A | 0.010823 | 0.007952 | 0.001395 | 1.20E-08 |
| rs75600034 | 3 | 57816106 | G | A | 0.087177 | 0.002203 | 0.000496 | 9.10E-06 |
| rs1947237 | 3 | 142879468 | C | T | 0.331349 | 0.001524 | 0.000299 | 3.50E-07 |
| rs12488768 | 3 | 53086234 | T | C | 0.575918 | 0.001353 | 0.000283 | 1.80E-06 |
| rs6805111 | 3 | 67095678 | A | G | 0.168976 | 0.001742 | 0.000373 | 3.00E-06 |
| rs76729464 | 4 | 164959236 | A | G | 0.071014 | 0.002598 | 0.000548 | 2.20E-06 |
| rs2735940 | 5 | 1296486 | G | A | 0.490304 | 0.001438 | 0.000281 | 3.00E-07 |
| rs72746180 | 5 | 40240420 | C | A | 0.298345 | 0.001705 | 0.000306 | 2.50E-08 |
| rs6939861 | 6 | 41703041 | A | G | 0.26195 | -0.00145 | 0.000322 | 6.80E-06 |
| rs6983267 | 8 | 128413305 | T | G | 0.482388 | -0.00268 | 0.00028 | 9.40E-22 |
| rs16892766 | 8 | 117630683 | C | A | 0.079541 | 0.003055 | 0.000516 | 3.20E-09 |
| rs184916336 | 8 | 2037032 | T | C | 0.057361 | 0.003139 | 0.000644 | 1.10E-06 |
| rs112063920 | 10 | 71624500 | T | C | 0.01492 | 0.005583 | 0.001234 | 6.00E-06 |
| rs142642563 | 10 | 21691162 | C | T | 0.017964 | -0.0049 | 0.001087 | 6.60E-06 |
| rs4944940 | 11 | 74415252 | A | G | 0.041808 | -0.00318 | 0.0007 | 5.60E-06 |
| rs7950728 | 11 | 126644477 | C | T | 0.299452 | -0.00145 | 0.000307 | 2.40E-06 |
| rs3087967 | 11 | 111156836 | C | T | 0.69919 | -0.00208 | 0.000306 | 1.10E-11 |
| rs12579000 | 12 | 54470921 | T | C | 0.55434 | 0.001264 | 0.000281 | 7.00E-06 |
| rs11835554 | 12 | 83677079 | C | G | 0.080296 | -0.00232 | 0.000518 | 7.80E-06 |
| rs4600332 | 13 | 111078872 | G | A | 0.634351 | 0.001541 | 0.00029 | 1.10E-07 |
| rs117014586 | 14 | 69998965 | T | C | 0.008275 | 0.007509 | 0.001615 | 3.30E-06 |
| rs12898159 | 14 | 54415291 | T | C | 0.402804 | 0.001328 | 0.000285 | 3.30E-06 |
| rs28647290 | 15 | 67405582 | A | G | 0.125971 | -0.00198 | 0.000431 | 4.40E-06 |
| rs58658771 | 15 | 33001734 | A | T | 0.180335 | 0.002809 | 0.000364 | 1.20E-14 |
| rs7199157 | 16 | 155966 | G | C | 0.495371 | -0.00129 | 0.000281 | 4.20E-06 |
| rs11548454 | 17 | 76972231 | A | G | 0.020188 | 0.004525 | 0.000999 | 5.90E-06 |
| rs75954926 | 17 | 81061048 | G | A | 0.655062 | 0.001577 | 0.000297 | 1.10E-07 |
| rs1078643 | 17 | 10707241 | A | G | 0.756823 | 0.00156 | 0.000326 | 1.70E-06 |
| rs4939827 | 18 | 46453463 | C | T | 0.478013 | -0.00236 | 0.00028 | 3.60E-17 |
| rs140544999 | 18 | 511139 | A | G | 0.032084 | 0.00378 | 0.00082 | 4.00E-06 |
| rs73039431 | 19 | 33523197 | G | A | 0.047268 | -0.00323 | 0.000662 | 1.10E-06 |
| rs11673650 | 19 | 16675947 | T | C | 0.04707 | 0.00293 | 0.000659 | 8.90E-06 |
| rs77930024 | 19 | 10155884 | T | C | 0.011996 | 0.006256 | 0.001384 | 6.20E-06 |
| rs6091117 | 20 | 48836883 | C | T | 0.610692 | -0.00128 | 0.000286 | 7.60E-06 |
| rs6085662 | 20 | 6698372 | C | G | 0.361938 | 0.001302 | 0.000292 | 7.90E-06 |
| rs1741640 | 20 | 60932414 | C | T | 0.755944 | 0.001685 | 0.000328 | 2.70E-07 |
| rs6066825 | 20 | 47340117 | G | A | 0.366086 | -0.00167 | 0.000292 | 1.10E-08 |
| rs143058554 | 22 | 45535850 | C | A | 0.015142 | 0.005746 | 0.00117 | 9.00E-07 |
| rs61604391 | 22 | 37395471 | A | T | 0.575036 | 0.001284 | 0.000283 | 5.50E-06 |

Table 52 Instrumental variables of Endometrial cancer

| SNP | Chr | Pos | EA | OA | EAF | Beta | SE | P |
| --- | --- | --- | --- | --- | --- | --- | --- | --- |
| rs113998067 | 1 | 38073356 | C | T | 0.048175 | 0.204335 | 0.037081 | 3.58E-08 |
| rs728048 | 1 | 34313808 | A | G | 0.234494 | -0.09363 | 0.018185 | 2.63E-07 |
| rs11583244 | 1 | 225952474 | T | C | 0.39026 | 0.078725 | 0.015939 | 7.85E-07 |
| rs7579014 | 2 | 60707894 | A | G | 0.646443 | 0.085224 | 0.016155 | 1.33E-07 |
| rs111507152 | 2 | 242667870 | C | G | 0.05011 | -0.20983 | 0.044547 | 2.47E-06 |
| rs148261157 | 2 | 60897579 | A | G | 0.040439 | 0.229226 | 0.041526 | 3.39E-08 |
| rs2960422 | 3 | 12334991 | A | G | 0.590223 | 0.078983 | 0.015689 | 4.80E-07 |
| rs1498445 | 3 | 168644843 | A | C | 0.749296 | -0.08226 | 0.017872 | 4.17E-06 |
| rs872267 | 3 | 127888067 | A | G | 0.262566 | -0.08441 | 0.017252 | 9.95E-07 |
| rs1428718 | 5 | 26534253 | T | C | 0.09029 | 0.117841 | 0.026351 | 7.75E-06 |
| rs7730712 | 5 | 36593781 | C | A | 0.054488 | 0.154335 | 0.034732 | 8.85E-06 |
| rs2736100 | 5 | 1286516 | A | C | 0.486561 | -0.069 | 0.01514 | 5.17E-06 |
| rs9296422 | 6 | 43905816 | C | G | 0.246567 | 0.085844 | 0.017555 | 1.01E-06 |
| rs9273561 | 6 | 32628998 | C | T | 0.224872 | -0.10323 | 0.021141 | 1.05E-06 |
| rs2747716 | 6 | 126008372 | G | A | 0.415954 | -0.0983 | 0.015595 | 2.91E-10 |
| rs76165228 | 6 | 150519175 | G | A | 0.851823 | -0.10323 | 0.022726 | 5.56E-06 |
| rs1740828 | 6 | 21649085 | A | G | 0.485476 | -0.13702 | 0.016845 | 4.15E-16 |
| rs72993664 | 6 | 152414489 | C | T | 0.242145 | 0.083134 | 0.018279 | 5.41E-06 |
| rs34670419 | 7 | 99130834 | T | G | 0.036333 | -0.22076 | 0.042999 | 2.84E-07 |
| rs117274813 | 7 | 68267626 | T | G | 0.017357 | 0.322256 | 0.071493 | 6.56E-06 |
| rs17158736 | 7 | 111332434 | C | G | 0.070299 | 0.136012 | 0.029516 | 4.06E-06 |
| rs144402098 | 7 | 56059736 | G | A | 0.010546 | 0.332805 | 0.072504 | 4.43E-06 |
| rs78623195 | 8 | 98241467 | G | A | 0.024664 | 0.248827 | 0.053736 | 3.65E-06 |
| rs139584729 | 8 | 129623902 | G | C | 0.01962 | -0.33001 | 0.060528 | 4.98E-08 |
| rs34284977 | 8 | 6947520 | A | G | 0.072894 | 0.1816 | 0.038859 | 2.96E-06 |
| rs4733613 | 8 | 129599278 | G | C | 0.862502 | -0.15479 | 0.0222 | 3.11E-12 |
| rs1679014 | 9 | 22207037 | C | T | 0.931754 | -0.16806 | 0.028943 | 6.38E-09 |
| rs3808757 | 9 | 17613627 | A | C | 0.034349 | 0.196171 | 0.043171 | 5.52E-06 |
| rs116885931 | 10 | 107529260 | T | C | 0.027113 | 0.203256 | 0.045783 | 9.02E-06 |
| rs4312007 | 10 | 87185828 | T | G | 0.490365 | 0.071982 | 0.015206 | 2.20E-06 |
| rs76982896 | 11 | 80349262 | T | C | 0.016701 | 0.291056 | 0.062463 | 3.17E-06 |
| rs10835920 | 11 | 32489664 | T | C | 0.364733 | 0.089208 | 0.015698 | 1.33E-08 |
| rs558029 | 11 | 4265076 | C | G | 0.134434 | 0.125307 | 0.027496 | 5.18E-06 |
| rs4767257 | 12 | 115214144 | T | C | 0.31958 | 0.096745 | 0.016507 | 4.61E-09 |
| rs7967338 | 12 | 109004950 | C | T | 0.358415 | -0.07397 | 0.016214 | 5.06E-06 |
| rs9668337 | 12 | 26426338 | A | G | 0.743413 | 0.107599 | 0.017655 | 1.10E-09 |
| rs1677893 | 12 | 78338386 | T | A | 0.540649 | -0.07846 | 0.01533 | 3.08E-07 |
| rs3184504 | 12 | 111884608 | C | T | 0.519822 | 0.098339 | 0.015252 | 1.14E-10 |
| rs11069840 | 13 | 110988807 | C | G | 0.074608 | 0.143899 | 0.031557 | 5.12E-06 |
| rs150540961 | 13 | 37269048 | G | A | 0.010485 | 0.542735 | 0.106323 | 3.31E-07 |
| rs7981863 | 13 | 73812141 | T | C | 0.265982 | -0.14643 | 0.017311 | 2.70E-17 |
| rs4072776 | 14 | 66287974 | C | T | 0.452546 | -0.08497 | 0.016657 | 3.38E-07 |
| rs79677411 | 15 | 41370582 | C | T | 0.03255 | 0.197586 | 0.041969 | 2.50E-06 |
| rs141265605 | 15 | 63822709 | G | A | 0.023049 | 0.250273 | 0.056149 | 8.30E-06 |
| rs2292072 | 15 | 82380865 | G | A | 0.010175 | 0.485352 | 0.094177 | 2.56E-07 |
| rs73416793 | 15 | 62037541 | C | T | 0.117483 | 0.107274 | 0.022848 | 2.66E-06 |
| rs937213 | 15 | 40322124 | C | T | 0.426577 | 0.089169 | 0.015256 | 5.07E-09 |
| rs17601876 | 15 | 51553909 | G | A | 0.484921 | 0.115832 | 0.015267 | 3.27E-14 |
| rs28472312 | 16 | 28826049 | T | C | 0.713245 | 0.084386 | 0.01765 | 1.74E-06 |
| rs34676612 | 16 | 10446142 | C | T | 0.136583 | 0.118059 | 0.024411 | 1.32E-06 |
| rs112358197 | 17 | 27673057 | A | G | 0.029054 | 0.228354 | 0.051438 | 9.02E-06 |
| rs882380 | 17 | 46294236 | A | C | 0.608645 | 0.091914 | 0.015688 | 4.66E-09 |
| rs11651052 | 17 | 36102381 | G | A | 0.547556 | 0.141454 | 0.015396 | 4.00E-20 |
| rs41462445 | 17 | 63410815 | C | G | 0.079971 | -0.13141 | 0.029542 | 8.66E-06 |
| rs150325239 | 17 | 75498474 | T | C | 0.015344 | 0.32397 | 0.069979 | 3.66E-06 |
| rs60856912 | 17 | 65892343 | T | G | 0.176289 | 0.102857 | 0.020376 | 4.47E-07 |
| rs74680322 | 19 | 52323592 | C | T | 0.042713 | -0.19579 | 0.041559 | 2.46E-06 |
| rs117917683 | 19 | 40936684 | G | C | 0.02318 | 0.242952 | 0.052894 | 4.37E-06 |
| rs4607001 | 20 | 22448537 | T | C | 0.210414 | 0.094963 | 0.019493 | 1.11E-06 |
| rs577034498 | 21 | 22378129 | C | A | 0.053018 | 0.161173 | 0.034471 | 2.93E-06 |
| rs5995580 | 22 | 38865863 | T | C | 0.06179 | 0.161355 | 0.03458 | 3.07E-06 |
| rs9616483 | 22 | 49577020 | A | T | 0.122385 | 0.112974 | 0.024 | 2.51E-06 |

Table 53 Instrumental variables of Gastric cancer

| SNP | Chr | Pos | EA | OA | EAF | Beta | SE | P |
| --- | --- | --- | --- | --- | --- | --- | --- | --- |
| rs7542186 | 1 | 156061222 | C | G | 0.970379 | 0.303076 | 0.054292 | 2.37E-08 |
| rs760077 | 1 | 155178782 | T | A | 0.849044 | 0.330759 | 0.026248 | 2.07E-36 |
| rs67643826 | 2 | 45436314 | A | G | 0.196223 | -0.10286 | 0.02295 | 7.40E-06 |
| rs76163157 | 4 | 189052325 | C | A | 0.015239 | -0.34808 | 0.078733 | 9.82E-06 |
| rs3805495 | 5 | 40755568 | T | C | 0.57871 | -0.16541 | 0.018174 | 8.92E-20 |
| rs2523653 | 6 | 31445514 | G | A | 0.800728 | 0.125161 | 0.02347 | 9.67E-08 |
| rs3997849 | 6 | 32682402 | T | C | 0.233868 | 0.13212 | 0.023036 | 9.73E-09 |
| rs13197513 | 6 | 32990121 | C | T | 0.049438 | -0.1841 | 0.040507 | 5.49E-06 |
| rs4712522 | 6 | 20656800 | G | C | 0.421435 | -0.08296 | 0.018316 | 5.91E-06 |
| rs9405098 | 6 | 32379736 | A | G | 0.246579 | 0.099089 | 0.020721 | 1.73E-06 |
| rs9368777 | 6 | 33788637 | C | G | 0.893154 | 0.172592 | 0.029058 | 2.86E-09 |
| rs11520970 | 7 | 91332384 | T | A | 0.800734 | -0.11253 | 0.025423 | 9.58E-06 |
| rs72690905 | 8 | 143713207 | T | C | 0.19401 | -0.17785 | 0.023389 | 2.87E-14 |
| rs2978977 | 8 | 143755720 | A | C | 0.493194 | 0.252482 | 0.018304 | 2.78E-43 |
| rs7025839 | 9 | 136124190 | A | G | 0.247486 | 0.109417 | 0.021296 | 2.78E-07 |
| rs2013486 | 11 | 12149531 | T | C | 0.326536 | -0.09878 | 0.019943 | 7.30E-07 |
| rs62641498 | 20 | 29827092 | G | T | 0.679847 | -0.11397 | 0.021598 | 1.32E-07 |

Table 54 Instrumental variables of Head and neck cancer

| SNP | Chr | Pos | EA | OA | EAF | Beta | SE | P |
| --- | --- | --- | --- | --- | --- | --- | --- | --- |
| rs12071290 | 1 | 7719695 | C | T | 0.220715 | -0.0007 | 0.000153 | 4.60E-06 |
| rs11161987 | 1 | 87818963 | A | G | 0.066362 | 0.001372 | 0.000252 | 5.50E-08 |
| rs2713209 | 2 | 122003442 | C | A | 0.019399 | 0.002026 | 0.000458 | 9.90E-06 |
| rs1265751 | 2 | 80913973 | T | C | 0.98056 | -0.00212 | 0.000477 | 8.70E-06 |
| rs62262157 | 3 | 105093483 | A | T | 0.044703 | 0.001409 | 0.000308 | 4.90E-06 |
| rs1141223 | 3 | 38565576 | T | G | 0.012901 | 0.002648 | 0.000557 | 2.00E-06 |
| rs78625750 | 4 | 100429326 | G | A | 0.077632 | -0.00108 | 0.000235 | 4.40E-06 |
| rs114007855 | 4 | 7735585 | T | C | 0.01769 | 0.002146 | 0.000483 | 8.90E-06 |
| rs42562 | 5 | 128404326 | C | T | 0.797499 | -0.00072 | 0.000157 | 4.80E-06 |
| rs139907207 | 5 | 139285648 | G | A | 0.016285 | 0.002266 | 0.000504 | 6.80E-06 |
| rs9273044 | 6 | 32611971 | C | G | 0.291172 | 0.000705 | 0.000154 | 4.80E-06 |
| rs12111777 | 7 | 2643908 | A | G | 0.029384 | 0.001825 | 0.000387 | 2.50E-06 |
| rs141222805 | 9 | 7326673 | C | G | 0.03015 | 0.001714 | 0.000381 | 6.90E-06 |
| rs35724731 | 11 | 34399619 | A | G | 0.255517 | 0.000682 | 0.000144 | 2.30E-06 |
| rs9538216 | 13 | 59384666 | A | G | 0.4161 | 0.000599 | 0.000128 | 3.00E-06 |
| rs4887047 | 15 | 78682139 | G | C | 0.021752 | 0.00201 | 0.000439 | 4.70E-06 |
| rs78981273 | 16 | 64940376 | A | G | 0.035218 | 0.001613 | 0.000348 | 3.60E-06 |
| rs144171660 | 17 | 49375951 | T | C | 0.012173 | 0.002624 | 0.000592 | 9.30E-06 |
| rs11672889 | 19 | 50444851 | G | C | 0.287918 | -0.00068 | 0.000147 | 3.40E-06 |
| rs16989370 | 20 | 39476582 | T | C | 0.021959 | 0.001937 | 0.000432 | 7.50E-06 |

Table 55 Instrumental variables of Liver & bile duct cancer

| SNP | Chr | Pos | EA | OA | EAF | Beta | SE | P |
| --- | --- | --- | --- | --- | --- | --- | --- | --- |
| rs1117660 | 1 | 92029710 | T | A | 0.170724 | -0.00044 | 9.53E-05 | 4.80E-06 |
| rs4120256 | 1 | 13841834 | C | T | 0.097965 | 0.000538 | 0.000121 | 8.60E-06 |
| rs75536586 | 2 | 18429258 | A | G | 0.065753 | 0.000697 | 0.000143 | 1.20E-06 |
| rs112207093 | 2 | 34012889 | G | A | 0.044405 | 0.000964 | 0.000198 | 1.10E-06 |
| rs4621364 | 3 | 45471624 | A | T | 0.236665 | 0.000402 | 8.45E-05 | 1.90E-06 |
| rs114348736 | 3 | 68671753 | A | G | 0.240552 | -0.00037 | 8.36E-05 | 8.10E-06 |
| rs12512024 | 4 | 184259446 | G | C | 0.041518 | 0.000835 | 0.000178 | 2.90E-06 |
| rs6872492 | 5 | 150972673 | C | G | 0.319139 | -0.00037 | 8.13E-05 | 4.80E-06 |
| rs79916965 | 6 | 17325388 | A | G | 0.053952 | 0.000871 | 0.000161 | 5.80E-08 |
| rs9267536 | 6 | 31651194 | C | A | 0.039279 | 0.000836 | 0.000182 | 4.50E-06 |
| rs10447540 | 7 | 2301484 | G | A | 0.399567 | 0.000333 | 7.25E-05 | 4.30E-06 |
| rs11765105 | 7 | 31766200 | A | G | 0.265714 | -0.00037 | 8.05E-05 | 5.80E-06 |
| rs117781420 | 9 | 19355687 | A | G | 0.057723 | 0.00069 | 0.000154 | 7.70E-06 |
| rs7028029 | 9 | 114254093 | G | A | 0.672964 | -0.00035 | 7.60E-05 | 4.10E-06 |
| rs141779100 | 9 | 112821258 | T | C | 0.058194 | 0.000674 | 0.000152 | 9.80E-06 |
| rs78771235 | 10 | 3276001 | A | G | 0.050172 | 0.000724 | 0.000163 | 8.50E-06 |
| rs7899293 | 10 | 33909579 | C | T | 0.130363 | 0.000485 | 0.000106 | 4.70E-06 |
| rs11013082 | 10 | 22942416 | G | A | 0.283968 | -0.00045 | 7.90E-05 | 1.30E-08 |
| rs11021605 | 11 | 96206391 | C | T | 0.40243 | -0.00033 | 7.24E-05 | 4.90E-06 |
| rs9561280 | 13 | 93875664 | G | A | 0.204811 | 0.000402 | 8.82E-05 | 5.10E-06 |
| rs12589608 | 14 | 52216526 | T | C | 0.143811 | 0.000542 | 0.000107 | 4.30E-07 |
| rs1542633 | 16 | 89339499 | C | G | 0.055745 | 0.00077 | 0.000155 | 6.60E-07 |
| rs649510 | 16 | 48061138 | A | G | 0.103394 | 0.000523 | 0.000118 | 8.80E-06 |
| rs12974659 | 19 | 23993112 | T | C | 0.05562 | 0.000735 | 0.000156 | 2.50E-06 |
| rs11671772 | 19 | 56438000 | A | G | 0.102726 | 0.00054 | 0.000117 | 4.00E-06 |
| rs2228603 | 19 | 19329924 | T | C | 0.074765 | 0.000679 | 0.000135 | 5.10E-07 |
| rs55853243 | 22 | 43623612 | A | G | 0.061791 | 0.000666 | 0.00015 | 8.40E-06 |

Table 56 Instrumental variables of Lung cancer

| SNP | Chr | Pos | EA | OA | EAF | Beta | SE | P |
| --- | --- | --- | --- | --- | --- | --- | --- | --- |
| rs112870428 | 1 | 214460656 | A | G | 0.068588 | 0.001754 | 0.000385 | 5.30E-06 |
| rs150425738 | 1 | 89124125 | A | G | 0.021367 | 0.003403 | 0.000688 | 7.70E-07 |
| rs10084460 | 2 | 37604136 | C | T | 0.287942 | -0.00099 | 0.000215 | 4.20E-06 |
| rs2164729 | 2 | 117758517 | C | T | 0.07868 | 0.001797 | 0.000364 | 8.10E-07 |
| rs17275627 | 4 | 107292176 | G | A | 0.026901 | 0.002785 | 0.000615 | 5.90E-06 |
| rs145244322 | 4 | 80343632 | G | A | 0.007268 | 0.005501 | 0.001185 | 3.40E-06 |
| rs62355139 | 5 | 30742803 | T | C | 0.110877 | 0.00144 | 0.00032 | 6.80E-06 |
| rs36019446 | 5 | 1339890 | G | A | 0.528371 | -0.00105 | 0.000195 | 8.70E-08 |
| rs144323430 | 5 | 117766802 | A | G | 0.018194 | 0.003358 | 0.000756 | 9.00E-06 |
| rs17335486 | 5 | 113056447 | G | C | 0.076949 | -0.00168 | 0.000365 | 4.00E-06 |
| rs28754928 | 6 | 32618476 | A | G | 0.431966 | -0.00115 | 0.000216 | 1.10E-07 |
| rs4398748 | 6 | 99219522 | T | C | 0.930118 | -0.00187 | 0.000399 | 2.60E-06 |
| rs72495774 | 8 | 108598574 | A | C | 0.01233 | 0.004064 | 0.000888 | 4.70E-06 |
| rs12543486 | 8 | 13012376 | C | T | 0.166792 | -0.00144 | 0.000262 | 4.40E-08 |
| rs11191835 | 10 | 105621859 | T | C | 0.607951 | -0.00091 | 0.000199 | 5.40E-06 |
| rs71475346 | 10 | 89108954 | G | T | 0.038624 | -0.00246 | 0.000543 | 6.20E-06 |
| rs111611526 | 10 | 130183798 | A | G | 0.015026 | 0.003809 | 0.00081 | 2.60E-06 |
| rs4923252 | 11 | 25235441 | T | G | 0.439215 | 0.000898 | 0.000196 | 4.70E-06 |
| rs79776350 | 12 | 119320808 | C | G | 0.025937 | 0.002964 | 0.000627 | 2.30E-06 |
| rs73172077 | 13 | 34552287 | G | A | 0.018815 | 0.003307 | 0.000732 | 6.20E-06 |
| rs78430609 | 13 | 101393254 | T | G | 0.013979 | 0.003919 | 0.000886 | 9.70E-06 |
| rs141884334 | 13 | 69459205 | A | G | 0.012407 | 0.004016 | 0.000898 | 7.70E-06 |
| rs75550918 | 15 | 66116154 | A | G | 0.016452 | 0.003471 | 0.000764 | 5.60E-06 |
| rs2009746 | 15 | 78754102 | G | A | 0.329847 | 0.001425 | 0.000207 | 5.70E-12 |
| rs116957319 | 16 | 12852522 | G | C | 0.026083 | 0.002691 | 0.000609 | 9.80E-06 |
| rs72838240 | 17 | 60701586 | A | G | 0.06275 | 0.002103 | 0.000411 | 3.10E-07 |
| rs138471905 | 19 | 44673059 | A | G | 0.0155 | 0.004053 | 0.000815 | 6.70E-07 |
| rs451740 | 22 | 18316620 | T | C | 0.305371 | 0.000974 | 0.000211 | 4.10E-06 |

Table 57 Instrumental variables of Malignant non-melanoma skin cancer

| SNP | Chr | Pos | EA | OA | EAF | Beta | SE | P |
| --- | --- | --- | --- | --- | --- | --- | --- | --- |
| rs2781249 | 1 | 41912138 | C | T | 0.91822 | 0.005021 | 0.000973 | 2.50E-07 |
| rs501823 | 1 | 28647181 | A | T | 0.829846 | 0.003321 | 0.000714 | 3.30E-06 |
| rs2476601 | 1 | 114377568 | G | A | 0.899399 | 0.005239 | 0.000884 | 3.00E-09 |
| rs6696046 | 1 | 217253266 | C | T | 0.405285 | -0.00257 | 0.000548 | 2.70E-06 |
| rs143773357 | 1 | 150345558 | T | C | 0.067232 | 0.006569 | 0.001079 | 1.20E-09 |
| rs12739596 | 1 | 165360114 | C | A | 0.239439 | 0.003152 | 0.000625 | 4.50E-07 |
| rs12070203 | 1 | 228994388 | T | C | 0.246166 | 0.010676 | 0.000617 | 5.40E-67 |
| rs3753639 | 1 | 154986091 | C | T | 0.243886 | 0.002866 | 0.000623 | 4.30E-06 |
| rs7528427 | 1 | 17746273 | T | C | 0.36183 | 0.008598 | 0.000554 | 2.70E-54 |
| rs7516462 | 1 | 15972597 | T | C | 0.255838 | -0.00274 | 0.000614 | 8.30E-06 |
| rs4149909 | 1 | 242023898 | G | A | 0.032732 | 0.006834 | 0.001497 | 5.00E-06 |
| rs76053290 | 2 | 182352013 | T | C | 0.02665 | -0.00731 | 0.001653 | 9.90E-06 |
| rs12692990 | 2 | 173057849 | G | A | 0.170909 | 0.003538 | 0.00071 | 6.30E-07 |
| rs1345149 | 2 | 134979983 | C | A | 0.740708 | -0.00283 | 0.000612 | 3.80E-06 |
| rs149341546 | 2 | 242659652 | C | A | 0.01587 | 0.010576 | 0.00228 | 3.50E-06 |
| rs79522206 | 2 | 7704860 | A | G | 0.026445 | 0.012333 | 0.001657 | 1.00E-13 |
| rs188686860 | 2 | 201733341 | T | C | 0.018394 | 0.008962 | 0.002021 | 9.20E-06 |
| rs340797 | 2 | 16490831 | T | G | 0.106307 | -0.00846 | 0.000864 | 1.30E-22 |
| rs3087243 | 2 | 204738919 | A | G | 0.450917 | 0.0045 | 0.000535 | 4.10E-17 |
| rs6738560 | 2 | 145634153 | A | G | 0.702348 | 0.003187 | 0.000582 | 4.40E-08 |
| rs700635 | 2 | 202153225 | A | C | 0.730992 | -0.00896 | 0.000602 | 3.70E-50 |
| rs2882274 | 2 | 5688981 | C | T | 0.514045 | 0.002718 | 0.000536 | 3.90E-07 |
| rs6707137 | 2 | 88554351 | A | G | 0.062835 | -0.01149 | 0.001102 | 2.00E-25 |
| rs184954814 | 2 | 189036510 | A | G | 0.024687 | -0.00853 | 0.00172 | 7.00E-07 |
| rs1800440 | 2 | 38298139 | C | T | 0.185871 | -0.00508 | 0.000684 | 1.10E-13 |
| rs17514215 | 3 | 189582241 | G | T | 0.174304 | 0.003301 | 0.000706 | 2.90E-06 |
| rs11720523 | 3 | 71545170 | A | C | 0.430349 | 0.006251 | 0.000539 | 3.70E-31 |
| rs60939770 | 3 | 46337606 | G | A | 0.313763 | 0.002765 | 0.000575 | 1.50E-06 |
| rs147997200 | 3 | 49726178 | A | G | 0.089137 | 0.004491 | 0.000945 | 2.00E-06 |
| rs341771 | 3 | 8979607 | T | C | 0.811141 | 0.003117 | 0.000684 | 5.20E-06 |
| rs8154 | 3 | 11596302 | C | T | 0.320099 | -0.00294 | 0.000569 | 2.50E-07 |
| rs73075145 | 3 | 55786748 | G | T | 0.208497 | 0.003015 | 0.000668 | 6.30E-06 |
| rs4638909 | 3 | 98471376 | T | C | 0.242411 | -0.00312 | 0.000623 | 5.60E-07 |
| rs4682103 | 3 | 112055793 | G | A | 0.529196 | 0.002455 | 0.000534 | 4.20E-06 |
| rs74644040 | 3 | 168568626 | A | G | 0.013423 | 0.010735 | 0.00231 | 3.40E-06 |
| rs73192661 | 3 | 188128794 | T | C | 0.451636 | 0.003844 | 0.000537 | 8.20E-13 |
| rs35286154 | 4 | 112781023 | A | C | 0.118375 | -0.00507 | 0.000826 | 8.50E-10 |
| rs559416132 | 4 | 151427424 | A | C | 0.169154 | 0.003179 | 0.000711 | 7.80E-06 |
| rs4301148 | 4 | 62103617 | T | C | 0.438938 | -0.00249 | 0.000537 | 3.40E-06 |
| rs2090632 | 4 | 186946015 | C | T | 0.683545 | -0.00263 | 0.000572 | 4.50E-06 |
| rs251459 | 5 | 149199148 | A | G | 0.203453 | -0.00343 | 0.000663 | 2.30E-07 |
| rs421284 | 5 | 1325590 | C | T | 0.442387 | -0.0079 | 0.000536 | 3.90E-49 |
| rs7705526 | 5 | 1285974 | A | C | 0.326104 | 0.003775 | 0.000576 | 5.50E-11 |
| rs42904 | 5 | 67743505 | T | C | 0.544371 | -0.00294 | 0.000537 | 4.50E-08 |
| rs16891982 | 5 | 33951693 | G | C | 0.971991 | 0.018088 | 0.001582 | 2.90E-30 |
| rs28363089 | 5 | 1412971 | A | G | 0.022672 | 0.008169 | 0.001788 | 4.90E-06 |
| rs62380786 | 5 | 141044179 | T | C | 0.084398 | 0.004596 | 0.000957 | 1.60E-06 |
| rs11741260 | 5 | 44412065 | A | G | 0.154743 | 0.003518 | 0.000735 | 1.70E-06 |
| rs9405066 | 6 | 31293644 | G | A | 0.426886 | -0.004 | 0.000636 | 3.20E-10 |
| rs6920314 | 6 | 150393262 | T | C | 0.592575 | 0.00248 | 0.000544 | 5.10E-06 |
| rs204295 | 6 | 167500562 | T | C | 0.463248 | 0.00356 | 0.000537 | 3.30E-11 |
| rs12528423 | 6 | 477223 | C | G | 0.124765 | -0.00441 | 0.000819 | 7.50E-08 |
| rs61447909 | 6 | 31409283 | A | G | 0.101063 | 0.006309 | 0.00088 | 7.50E-13 |
| rs35657997 | 6 | 32542236 | G | A | 0.112648 | 0.008825 | 0.000875 | 6.20E-24 |
| rs9275608 | 6 | 32683708 | G | A | 0.110994 | -0.0064 | 0.000878 | 3.20E-13 |
| rs2294214 | 6 | 22056923 | C | A | 0.315928 | 0.00504 | 0.000572 | 1.30E-18 |
| rs7740107 | 6 | 130374461 | A | T | 0.735973 | 0.002845 | 0.000604 | 2.50E-06 |
| rs12203592 | 6 | 396321 | T | C | 0.219691 | 0.018266 | 0.000634 | 9.29E-183 |
| rs58672943 | 6 | 11817106 | G | T | 0.082073 | 0.004554 | 0.001 | 5.30E-06 |
| rs6908626 | 6 | 91005743 | T | G | 0.177952 | -0.00569 | 0.0007 | 4.60E-16 |
| rs539094004 | 6 | 82293552 | G | T | 0.011854 | 0.012071 | 0.002518 | 1.60E-06 |
| rs77843555 | 6 | 372762 | T | C | 0.027196 | -0.00874 | 0.001727 | 4.20E-07 |
| rs125124 | 7 | 130584684 | G | C | 0.303582 | -0.00619 | 0.000582 | 2.10E-26 |
| rs117744081 | 7 | 29132279 | G | A | 0.032155 | -0.01041 | 0.00151 | 5.30E-12 |
| rs73183650 | 7 | 101421000 | T | A | 0.248087 | -0.00557 | 0.000618 | 2.20E-19 |
| rs2965634 | 7 | 52905808 | T | C | 0.188369 | 0.003171 | 0.000693 | 4.70E-06 |
| rs10950456 | 7 | 1979750 | A | G | 0.556606 | -0.00252 | 0.000538 | 2.80E-06 |
| rs35761891 | 7 | 6438217 | G | A | 0.04579 | -0.00705 | 0.001336 | 1.30E-07 |
| rs834603 | 7 | 47447921 | G | A | 0.493381 | -0.00341 | 0.000533 | 1.50E-10 |
| rs147871346 | 7 | 329891 | T | C | 0.055389 | 0.006453 | 0.001394 | 3.60E-06 |
| rs10250629 | 7 | 50191353 | C | T | 0.421236 | -0.00321 | 0.000541 | 3.00E-09 |
| rs2142331 | 8 | 116636719 | T | C | 0.602418 | -0.00252 | 0.000545 | 3.70E-06 |
| rs2241261 | 8 | 22876739 | T | C | 0.52374 | 0.002856 | 0.000537 | 1.10E-07 |
| rs55942834 | 8 | 81411215 | G | A | 0.194998 | -0.00561 | 0.000673 | 8.30E-17 |
| rs62512839 | 8 | 129009401 | G | A | 0.340963 | 0.002783 | 0.000562 | 7.30E-07 |
| rs62500177 | 8 | 32280225 | T | C | 0.43923 | 0.002546 | 0.00054 | 2.50E-06 |
| rs77501143 | 8 | 145252484 | T | G | 0.048595 | -0.00677 | 0.001329 | 3.60E-07 |
| rs12546962 | 8 | 9198185 | G | A | 0.259075 | 0.002819 | 0.000611 | 4.00E-06 |
| rs35251485 | 8 | 77483430 | A | G | 0.063052 | -0.01383 | 0.001107 | 8.40E-36 |
| rs138618201 | 8 | 90977881 | A | C | 0.046146 | 0.00569 | 0.001281 | 8.90E-06 |
| rs6989943 | 8 | 101028239 | A | G | 0.15457 | -0.00916 | 0.000738 | 2.00E-35 |
| rs10991115 | 9 | 106896111 | T | C | 0.399282 | 0.003205 | 0.000544 | 3.80E-09 |
| rs35142681 | 9 | 113449489 | T | C | 0.030917 | 0.006902 | 0.001538 | 7.20E-06 |
| rs7867194 | 9 | 681862 | A | C | 0.442862 | 0.002889 | 0.000539 | 8.40E-08 |
| rs10962474 | 9 | 16529554 | T | C | 0.055492 | -0.01017 | 0.001172 | 4.20E-18 |
| rs10965219 | 9 | 22053687 | G | A | 0.480967 | -0.0065 | 0.000532 | 2.80E-34 |
| rs10962605 | 9 | 16799623 | G | A | 0.758714 | 0.00371 | 0.000622 | 2.40E-09 |
| rs539323426 | 9 | 93896492 | G | A | 0.016248 | 0.010424 | 0.002303 | 6.00E-06 |
| rs7870431 | 9 | 44802161 | A | T | 0.659196 | 0.00381 | 0.000818 | 3.20E-06 |
| rs12350739 | 9 | 16885017 | A | G | 0.607959 | 0.004759 | 0.000549 | 4.20E-18 |
| rs2773350 | 9 | 100399132 | A | G | 0.181175 | -0.00309 | 0.000691 | 7.70E-06 |
| rs2485630 | 10 | 103842365 | G | A | 0.72407 | 0.003123 | 0.000599 | 1.90E-07 |
| rs7894774 | 10 | 67593008 | T | C | 0.11511 | 0.003918 | 0.00085 | 4.00E-06 |
| rs72782793 | 10 | 8083024 | C | T | 0.183987 | -0.0033 | 0.000687 | 1.60E-06 |
| rs79134926 | 10 | 8964984 | T | G | 0.116922 | -0.01225 | 0.000829 | 1.90E-49 |
| rs2077394 | 10 | 80602647 | A | G | 0.412386 | -0.00298 | 0.000542 | 3.80E-08 |
| rs2387397 | 10 | 6390192 | C | G | 0.780587 | -0.00291 | 0.000648 | 7.00E-06 |
| rs10995251 | 10 | 64398466 | T | C | 0.36914 | 0.002932 | 0.000551 | 1.00E-07 |
| rs174449 | 11 | 61640379 | A | G | 0.636274 | 0.003077 | 0.000557 | 3.20E-08 |
| rs875311 | 11 | 65583340 | T | C | 0.4551 | -0.00341 | 0.000534 | 1.60E-10 |
| rs11039134 | 11 | 47242472 | G | A | 0.66454 | 0.002803 | 0.000567 | 7.60E-07 |
| rs2895570 | 11 | 94463093 | C | T | 0.304342 | -0.00329 | 0.000591 | 2.60E-08 |
| rs615716 | 11 | 128594851 | T | C | 0.739363 | -0.00269 | 0.000606 | 8.80E-06 |
| rs118019665 | 11 | 26398982 | G | C | 0.050345 | 0.005461 | 0.001219 | 7.50E-06 |
| rs7931483 | 11 | 76302067 | A | C | 0.477779 | 0.002516 | 0.000535 | 2.60E-06 |
| rs267449 | 11 | 33281328 | T | C | 0.809548 | 0.003037 | 0.00068 | 7.90E-06 |
| rs1126809 | 11 | 89017961 | A | G | 0.303379 | 0.007335 | 0.000579 | 1.00E-36 |
| rs12420071 | 11 | 632059 | C | T | 0.269354 | 0.00278 | 0.000612 | 5.60E-06 |
| rs663743 | 11 | 64107735 | A | G | 0.34083 | 0.004078 | 0.000563 | 4.30E-13 |
| rs206966 | 12 | 120832146 | T | C | 0.154491 | -0.00369 | 0.000764 | 1.30E-06 |
| rs11059675 | 12 | 122668326 | A | G | 0.46037 | -0.00273 | 0.000537 | 3.70E-07 |
| rs11146931 | 12 | 133139130 | C | T | 0.417642 | 0.002785 | 0.000543 | 2.90E-07 |
| rs9668178 | 12 | 26421434 | A | T | 0.240933 | 0.00293 | 0.000624 | 2.70E-06 |
| rs2720302 | 12 | 50179021 | A | G | 0.32806 | 0.002914 | 0.000577 | 4.40E-07 |
| rs11170164 | 12 | 52913668 | T | C | 0.077792 | 0.010804 | 0.000995 | 1.80E-27 |
| rs3213737 | 12 | 96379806 | A | G | 0.575011 | -0.00287 | 0.00054 | 1.10E-07 |
| rs10431257 | 12 | 24624272 | T | A | 0.361771 | -0.00245 | 0.000554 | 9.60E-06 |
| rs11614658 | 12 | 110082320 | C | T | 0.232185 | -0.00281 | 0.000634 | 9.50E-06 |
| rs9534270 | 13 | 32939314 | T | C | 0.524274 | -0.00265 | 0.000534 | 6.90E-07 |
| rs879552 | 14 | 104010198 | A | G | 0.372071 | -0.00262 | 0.000554 | 2.30E-06 |
| rs28367284 | 14 | 89933608 | T | C | 0.080184 | 0.004639 | 0.000978 | 2.10E-06 |
| rs11849449 | 14 | 51902566 | T | G | 0.144851 | 0.003865 | 0.000762 | 3.90E-07 |
| rs61978934 | 14 | 75925202 | T | C | 0.069976 | 0.006728 | 0.001045 | 1.20E-10 |
| rs12913832 | 15 | 28365618 | G | A | 0.776499 | 0.003935 | 0.000635 | 5.60E-10 |
| rs117673640 | 15 | 63625769 | C | T | 0.00642 | 0.016574 | 0.003679 | 6.60E-06 |
| rs6493383 | 15 | 50160577 | G | A | 0.303017 | -0.00351 | 0.000583 | 1.90E-09 |
| rs11853333 | 15 | 40742338 | A | G | 0.35749 | -0.0025 | 0.000562 | 8.60E-06 |
| rs11641787 | 16 | 10902090 | A | G | 0.015999 | 0.010267 | 0.002155 | 1.90E-06 |
| rs4238604 | 16 | 11420769 | G | C | 0.551508 | -0.0031 | 0.000535 | 7.40E-09 |
| rs78498439 | 16 | 89967514 | A | G | 0.084816 | 0.008385 | 0.000959 | 2.30E-18 |
| rs140762356 | 16 | 67792094 | C | T | 0.047078 | 0.005745 | 0.001273 | 6.40E-06 |
| rs111605729 | 16 | 89151601 | A | G | 0.016621 | -0.00967 | 0.002164 | 7.90E-06 |
| rs17225866 | 16 | 89874085 | A | G | 0.030646 | -0.00702 | 0.001556 | 6.40E-06 |
| rs144242147 | 16 | 68937606 | T | C | 0.157935 | -0.00363 | 0.000735 | 7.80E-07 |
| rs62052678 | 16 | 90165964 | G | A | 0.070283 | -0.00775 | 0.001067 | 3.60E-13 |
| rs79726587 | 16 | 50167787 | G | A | 0.062289 | -0.00513 | 0.001112 | 3.90E-06 |
| rs1805007 | 16 | 89986117 | T | C | 0.102274 | 0.017272 | 0.000875 | 9.10E-87 |
| rs7405790 | 17 | 79621634 | G | A | 0.60824 | 0.002576 | 0.000548 | 2.60E-06 |
| rs12600758 | 17 | 40566463 | C | T | 0.181039 | -0.00314 | 0.000698 | 6.90E-06 |
| rs2306759 | 17 | 80540585 | G | A | 0.427534 | 0.00302 | 0.00056 | 7.10E-08 |
| rs854801 | 17 | 18081615 | T | C | 0.423516 | 0.002976 | 0.000539 | 3.30E-08 |
| rs78378222 | 17 | 7571752 | G | T | 0.012131 | 0.024955 | 0.002486 | 1.00E-23 |
| rs76770092 | 18 | 7600193 | C | G | 0.040672 | 0.00616 | 0.00135 | 5.00E-06 |
| rs75124228 | 18 | 74126119 | T | C | 0.03698 | -0.00632 | 0.001428 | 9.60E-06 |
| rs10415576 | 19 | 50164390 | C | T | 0.379493 | 0.002482 | 0.000551 | 6.70E-06 |
| rs56069439 | 19 | 17393925 | A | C | 0.293908 | 0.002995 | 0.000584 | 3.00E-07 |
| rs10424978 | 19 | 4837557 | A | C | 0.600405 | 0.003456 | 0.00055 | 3.20E-10 |
| rs6090049 | 20 | 62750247 | A | G | 0.469132 | -0.00258 | 0.000537 | 1.60E-06 |
| rs36011691 | 20 | 2170199 | A | G | 0.102698 | -0.00685 | 0.000876 | 5.10E-15 |
| rs214793 | 20 | 2285705 | T | C | 0.814825 | -0.01086 | 0.000688 | 3.10E-56 |
| rs6060648 | 20 | 34456704 | G | A | 0.142581 | -0.0039 | 0.000761 | 2.90E-07 |
| rs6059655 | 20 | 32665748 | G | A | 0.897536 | -0.01504 | 0.000894 | 1.50E-63 |
| rs111650620 | 20 | 34591725 | A | G | 0.002208 | 0.091281 | 0.014691 | 5.20E-10 |
| rs11908416 | 20 | 49392745 | A | G | 0.241892 | -0.00475 | 0.000625 | 2.90E-14 |
| rs2849699 | 21 | 43089086 | G | C | 0.320289 | -0.00529 | 0.000572 | 2.20E-20 |
| rs42936 | 22 | 30962878 | A | G | 0.657123 | 0.002868 | 0.000569 | 4.60E-07 |

Table 58 Instrumental variables of Melanoma skin cancer

| SNP | Chr | Pos | EA | OA | EAF | Beta | SE | P |
| --- | --- | --- | --- | --- | --- | --- | --- | --- |
| rs11120349 | 1 | 214718513 | T | A | 0.20584 | -0.00166 | 0.000285 | 6.10E-09 |
| rs1341336 | 1 | 226596389 | G | A | 0.325354 | -0.00115 | 0.000245 | 3.10E-06 |
| rs112917875 | 1 | 204149459 | A | G | 0.0101 | 0.005364 | 0.001165 | 4.20E-06 |
| rs143732092 | 1 | 106091862 | T | C | 0.009523 | 0.005559 | 0.0012 | 3.60E-06 |
| rs142267292 | 1 | 85920999 | G | A | 0.012158 | 0.005127 | 0.001093 | 2.70E-06 |
| rs78873744 | 1 | 183105105 | A | G | 0.024466 | 0.003291 | 0.000743 | 9.60E-06 |
| rs112227183 | 2 | 2315409 | T | G | 0.026148 | 0.003319 | 0.000724 | 4.50E-06 |
| rs116290987 | 3 | 88481220 | C | T | 0.029581 | 0.003103 | 0.0007 | 9.20E-06 |
| rs112085630 | 3 | 13885533 | T | C | 0.01875 | 0.003854 | 0.00087 | 9.50E-06 |
| rs183783391 | 3 | 69950451 | T | C | 0.013683 | 0.006939 | 0.00101 | 6.30E-12 |
| rs11707914 | 3 | 156547771 | C | A | 0.593382 | 0.001066 | 0.000234 | 5.10E-06 |
| rs73112315 | 3 | 61096839 | G | T | 0.01343 | 0.004555 | 0.001018 | 7.70E-06 |
| rs60848393 | 4 | 141538862 | C | G | 0.196407 | 0.001382 | 0.000289 | 1.70E-06 |
| rs147602346 | 4 | 85343178 | G | A | 0.013432 | 0.004858 | 0.001032 | 2.50E-06 |
| rs17354643 | 4 | 84015165 | A | G | 0.054591 | 0.002577 | 0.000505 | 3.30E-07 |
| rs31490 | 5 | 1344458 | A | G | 0.435464 | 0.00183 | 0.000231 | 2.60E-15 |
| rs717763 | 5 | 53686499 | T | C | 0.134373 | 0.001561 | 0.000343 | 5.20E-06 |
| rs16891982 | 5 | 33951693 | G | C | 0.971432 | 0.005242 | 0.000675 | 8.10E-15 |
| rs6937555 | 6 | 21159188 | C | G | 0.322469 | 0.001247 | 0.000246 | 3.90E-07 |
| rs9444473 | 6 | 87807529 | A | T | 0.35243 | -0.0011 | 0.000242 | 5.70E-06 |
| rs77686082 | 7 | 155052366 | A | G | 0.019065 | 0.003805 | 0.000847 | 7.10E-06 |
| rs117132860 | 7 | 17134708 | A | G | 0.025803 | 0.003541 | 0.000723 | 9.70E-07 |
| rs560061966 | 9 | 109680321 | A | T | 0.006841 | 0.007749 | 0.001467 | 1.30E-07 |
| rs3217986 | 9 | 22005330 | G | T | 0.092673 | 0.001889 | 0.000396 | 1.90E-06 |
| rs871024 | 9 | 21803880 | A | C | 0.519167 | -0.00189 | 0.00023 | 1.80E-16 |
| rs2488002 | 10 | 105679341 | T | C | 0.843575 | -0.00195 | 0.000315 | 6.70E-10 |
| rs1873456 | 10 | 71709374 | A | G | 0.409961 | -0.00117 | 0.000234 | 5.80E-07 |
| rs144384185 | 10 | 119947937 | C | A | 0.010194 | -0.00546 | 0.001212 | 6.70E-06 |
| rs4277005 | 10 | 8511993 | A | G | 0.93106 | 0.002092 | 0.000454 | 4.10E-06 |
| rs11203046 | 10 | 91000321 | T | C | 0.065162 | 0.002264 | 0.000465 | 1.10E-06 |
| rs12278954 | 11 | 108328952 | A | C | 0.15327 | -0.00181 | 0.000319 | 1.30E-08 |
| rs1126809 | 11 | 89017961 | A | G | 0.302059 | 0.002038 | 0.00025 | 3.60E-16 |
| rs2300560 | 12 | 96378259 | T | C | 0.355778 | -0.00107 | 0.00024 | 7.70E-06 |
| rs117412802 | 13 | 111143601 | G | A | 0.013821 | 0.004366 | 0.000981 | 8.60E-06 |
| rs1800407 | 15 | 28230318 | T | C | 0.085367 | 0.001928 | 0.00041 | 2.50E-06 |
| rs1080476 | 15 | 50281051 | A | G | 0.279395 | 0.001332 | 0.000256 | 2.10E-07 |
| rs1805007 | 16 | 89986117 | T | C | 0.101025 | 0.005736 | 0.000379 | 9.90E-52 |
| rs181286049 | 16 | 89494659 | T | C | 0.008595 | 0.005743 | 0.001284 | 7.70E-06 |
| rs74415461 | 16 | 89990843 | T | C | 0.084956 | 0.003873 | 0.000413 | 7.10E-21 |
| rs66487709 | 17 | 20928846 | C | T | 0.306844 | 0.001114 | 0.000249 | 7.90E-06 |
| rs61758854 | 18 | 61002652 | A | G | 0.033035 | 0.003382 | 0.000642 | 1.40E-07 |
| rs117490768 | 19 | 33888084 | A | G | 0.026855 | 0.003197 | 0.000724 | 9.90E-06 |
| rs117077437 | 20 | 17271886 | C | T | 0.014521 | -0.00508 | 0.000986 | 2.60E-07 |
| rs6059655 | 20 | 32665748 | G | A | 0.898713 | -0.00383 | 0.000387 | 3.70E-23 |
| rs443099 | 21 | 42743327 | T | G | 0.606603 | 0.001259 | 0.000235 | 8.50E-08 |

Table 59 Instrumental variables of Oesophageal cancer

| SNP | Chr | Pos | EA | OA | EAF | Beta | SE | P |
| --- | --- | --- | --- | --- | --- | --- | --- | --- |
| rs9726252 | 1 | 43462020 | C | T | 0.849279 | -0.00064 | 0.000144 | 9.50E-06 |
| rs146586036 | 1 | 197349836 | G | A | 0.01777 | 0.001982 | 0.0004 | 7.30E-07 |
| rs76112781 | 1 | 164951765 | C | G | 0.019779 | 0.001711 | 0.000375 | 5.10E-06 |
| rs112879361 | 2 | 219190737 | A | G | 0.034249 | 0.00134 | 0.000302 | 9.30E-06 |
| rs139840394 | 2 | 69030139 | T | A | 0.019472 | 0.001691 | 0.00038 | 8.40E-06 |
| rs73951248 | 2 | 106795719 | T | C | 0.024354 | 0.001657 | 0.000335 | 7.60E-07 |
| rs13078185 | 3 | 159434975 | G | T | 0.149998 | 0.00069 | 0.000145 | 2.00E-06 |
| rs77903146 | 3 | 172418263 | C | T | 0.028422 | 0.001478 | 0.000324 | 5.00E-06 |
| rs62289210 | 4 | 3903945 | G | A | 0.258479 | 0.000594 | 0.000122 | 1.10E-06 |
| rs184145524 | 4 | 81009345 | T | C | 0.020881 | 0.002033 | 0.000418 | 1.20E-06 |
| rs116325239 | 5 | 113735530 | A | G | 0.035043 | 0.001322 | 0.00028 | 2.30E-06 |
| rs292206 | 5 | 73507765 | T | C | 0.648997 | -0.00055 | 0.000115 | 1.90E-06 |
| rs34103843 | 5 | 172872881 | G | A | 0.265862 | 0.000565 | 0.000117 | 1.40E-06 |
| rs13205661 | 6 | 104727074 | C | G | 0.10157 | 0.000778 | 0.000172 | 5.80E-06 |
| rs801537 | 7 | 18639046 | G | A | 0.824804 | 0.000611 | 0.000136 | 7.20E-06 |
| rs117372360 | 9 | 134980520 | A | G | 0.033666 | 0.001431 | 0.0003 | 1.80E-06 |
| rs1780254 | 10 | 29013251 | A | G | 0.153902 | 0.000651 | 0.000143 | 5.10E-06 |
| rs3802909 | 11 | 121772792 | A | T | 0.312028 | 0.000572 | 0.000112 | 3.00E-07 |
| rs76133423 | 12 | 10814264 | A | G | 0.038284 | 0.001233 | 0.000274 | 6.60E-06 |
| rs78011018 | 13 | 92389790 | T | C | 0.019422 | 0.002059 | 0.000427 | 1.40E-06 |
| rs1630460 | 14 | 20383755 | C | T | 0.718236 | -0.00069 | 0.000143 | 1.70E-06 |
| rs17669329 | 17 | 28869877 | C | T | 0.092344 | 0.000847 | 0.000182 | 3.10E-06 |
| rs151291107 | 17 | 72713935 | A | C | 0.020578 | 0.001958 | 0.000411 | 1.90E-06 |
| rs658836 | 18 | 9640985 | C | T | 0.234623 | 0.000548 | 0.000123 | 8.60E-06 |
| rs117971589 | 19 | 14748068 | A | G | 0.033036 | 0.001334 | 0.000294 | 5.50E-06 |
| rs139727776 | 19 | 722064 | G | A | 0.062864 | 0.001016 | 0.00022 | 3.90E-06 |
| rs2835394 | 21 | 37962171 | A | C | 0.59924 | 0.000469 | 0.000105 | 8.60E-06 |

Table 60 Instrumental variables of Ovarian cancer

| SNP | Chr | Pos | EA | OA | EAF | Beta | SE | P |
| --- | --- | --- | --- | --- | --- | --- | --- | --- |
| rs35486093 | 1 | 85729820 | G | A | 0.090246 | -0.00211 | 0.000431 | 1.00E-06 |
| rs1800895 | 1 | 206946472 | T | C | 0.013629 | 0.005855 | 0.0011 | 1.00E-07 |
| rs61770097 | 1 | 34886788 | A | G | 0.021327 | 0.003903 | 0.00088 | 9.30E-06 |
| rs78231145 | 2 | 98453267 | T | A | 0.016594 | 0.005197 | 0.001012 | 2.80E-07 |
| rs72985919 | 2 | 230411633 | A | G | 0.027926 | 0.004062 | 0.000747 | 5.50E-08 |
| rs7593089 | 2 | 212646801 | G | T | 0.010399 | 0.005993 | 0.00124 | 1.30E-06 |
| rs75882398 | 3 | 134682295 | C | T | 0.012178 | 0.005717 | 0.001153 | 7.10E-07 |
| rs79693379 | 3 | 87006367 | G | A | 0.045021 | 0.002807 | 0.000593 | 2.20E-06 |
| rs73167285 | 3 | 172071557 | G | A | 0.03746 | 0.003286 | 0.000683 | 1.50E-06 |
| rs75014964 | 4 | 23014955 | G | A | 0.02175 | 0.003868 | 0.000861 | 7.00E-06 |
| rs149609962 | 5 | 66332540 | G | A | 0.014585 | 0.005391 | 0.001063 | 3.90E-07 |
| rs72909914 | 6 | 81369040 | T | A | 0.025767 | 0.003828 | 0.000795 | 1.50E-06 |
| rs114022155 | 6 | 6937245 | T | C | 0.021252 | 0.003837 | 0.000863 | 8.80E-06 |
| rs76264086 | 6 | 152680071 | C | T | 0.015342 | 0.004767 | 0.001007 | 2.20E-06 |
| rs4443540 | 6 | 1505511 | T | C | 0.201553 | 0.001726 | 0.000316 | 4.70E-08 |
| rs149718150 | 7 | 42288203 | A | G | 0.010469 | 0.005639 | 0.001251 | 6.50E-06 |
| rs143046132 | 7 | 92840793 | G | T | 0.010919 | 0.005883 | 0.001312 | 7.30E-06 |
| rs73711692 | 8 | 131412497 | C | T | 0.054002 | -0.00249 | 0.000547 | 5.30E-06 |
| rs117247154 | 9 | 135217116 | T | C | 0.044575 | 0.002907 | 0.000597 | 1.10E-06 |
| rs1687403 | 9 | 117061023 | C | T | 0.07545 | 0.002466 | 0.00047 | 1.50E-07 |
| rs113811212 | 11 | 69406283 | T | G | 0.056643 | 0.002336 | 0.000526 | 9.10E-06 |
| rs144948468 | 11 | 59176837 | G | A | 0.022304 | 0.003906 | 0.000861 | 5.80E-06 |
| rs1358253 | 12 | 16414427 | G | A | 0.288045 | 0.001291 | 0.000275 | 2.60E-06 |
| rs117961172 | 12 | 55062468 | A | G | 0.019445 | 0.004282 | 0.000891 | 1.50E-06 |
| rs34812126 | 12 | 115133341 | A | G | 0.080201 | 0.002055 | 0.000454 | 5.90E-06 |
| rs79009585 | 15 | 89326398 | T | G | 0.019385 | 0.004571 | 0.000893 | 3.00E-07 |
| rs28678815 | 18 | 61004727 | T | C | 0.254527 | 0.001327 | 0.000284 | 2.90E-06 |
| rs114858887 | 18 | 65809262 | G | A | 0.025257 | 0.0042 | 0.000792 | 1.10E-07 |
| rs9961715 | 18 | 3824312 | C | T | 0.194049 | 0.001402 | 0.000312 | 7.10E-06 |
| rs2143612 | 20 | 56888997 | C | T | 0.037447 | 0.003072 | 0.000653 | 2.60E-06 |

Table 61 Instrumental variables of Pancreatic cancer

| SNP | Chr | Pos | EA | OA | EAF | Beta | SE | P |
| --- | --- | --- | --- | --- | --- | --- | --- | --- |
| rs60579835 | 1 | 113113194 | T | C | 0.468425 | 0.392582 | 0.068506 | 1.00E-08 |
| rs13006138 | 2 | 183541066 | T | A | 0.595075 | 0.310516 | 0.070064 | 9.34E-06 |
| rs2047388 | 3 | 21072840 | G | A | 0.510456 | 0.309794 | 0.067314 | 4.18E-06 |
| rs77961928 | 4 | 187798362 | C | T | 0.124586 | 0.563901 | 0.114028 | 7.60E-07 |
| rs195979 | 4 | 163566956 | T | G | 0.116759 | 0.508559 | 0.112099 | 5.71E-06 |
| rs17055404 | 8 | 26383935 | A | G | 0.024458 | 1.32302 | 0.27884 | 2.09E-06 |
| rs6564887 | 16 | 81583540 | C | T | 0.885517 | -0.51155 | 0.111932 | 4.87E-06 |

Table 62 Instrumental variables of Prostate cancer

| SNP | Chr | Pos | EA | OA | EAF | Beta | SE | P |
| --- | --- | --- | --- | --- | --- | --- | --- | --- |
| rs149642445 | 1 | 107767065 | G | A | 0.008511 | 0.005456 | 0.001176 | 3.47E-06 |
| rs2539982 | 2 | 63111004 | A | G | 0.53957 | 0.00109 | 0.000209 | 1.72E-07 |
| rs138059817 | 2 | 10027099 | T | C | 0.011428 | 0.005009 | 0.00101 | 7.03E-07 |
| rs80353656 | 2 | 173309402 | C | T | 0.062533 | -0.00258 | 0.00043 | 1.98E-09 |
| rs934859 | 2 | 171370372 | G | A | 0.49949 | -0.00117 | 0.000208 | 1.97E-08 |
| rs73142674 | 3 | 87200497 | C | T | 0.133346 | 0.001484 | 0.000306 | 1.21E-06 |
| rs78416326 | 3 | 170074517 | C | G | 0.200092 | -0.00147 | 0.000262 | 1.80E-08 |
| rs2060848 | 4 | 75725785 | C | G | 0.070973 | 0.00187 | 0.000405 | 3.86E-06 |
| rs139634578 | 4 | 169730429 | T | C | 0.018236 | 0.003653 | 0.000788 | 3.54E-06 |
| rs6825684 | 4 | 106084643 | A | G | 0.125303 | -0.00158 | 0.000314 | 4.78E-07 |
| rs2510768 | 4 | 95482053 | A | G | 0.547125 | -0.00102 | 0.000209 | 1.13E-06 |
| rs76252384 | 5 | 133868014 | A | G | 0.020715 | 0.003405 | 0.00074 | 4.17E-06 |
| rs10866527 | 5 | 1891800 | T | C | 0.462974 | 0.000945 | 0.000211 | 7.23E-06 |
| rs11750860 | 5 | 118221589 | A | G | 0.057414 | 0.001997 | 0.000451 | 9.30E-06 |
| rs113639366 | 5 | 34603107 | C | T | 0.253469 | 0.001122 | 0.000241 | 3.27E-06 |
| rs184995862 | 6 | 34016653 | A | G | 0.011381 | 0.004849 | 0.001048 | 3.71E-06 |
| rs62402376 | 6 | 28507446 | C | A | 0.188715 | 0.001236 | 0.000266 | 3.31E-06 |
| rs10486567 | 7 | 27976563 | A | G | 0.232413 | -0.00127 | 0.000246 | 2.32E-07 |
| rs11768309 | 7 | 97773812 | A | C | 0.535439 | -0.00108 | 0.000208 | 2.19E-07 |
| rs38908 | 7 | 116894926 | G | A | 0.473912 | 0.001087 | 0.000209 | 1.91E-07 |
| rs28637545 | 8 | 130465270 | C | A | 0.177656 | 0.001262 | 0.000272 | 3.59E-06 |
| rs6983267 | 8 | 128413305 | T | G | 0.48044 | -0.00189 | 0.000208 | 1.06E-19 |
| rs13267884 | 8 | 23531987 | G | A | 0.590417 | -0.00094 | 0.000212 | 9.76E-06 |
| rs28615829 | 8 | 128018205 | A | G | 0.296066 | -0.00107 | 0.000228 | 2.53E-06 |
| rs77541621 | 8 | 128077146 | A | G | 0.029202 | 0.005853 | 0.000642 | 7.88E-20 |
| rs4871813 | 8 | 128547942 | G | T | 0.903887 | -0.00217 | 0.000371 | 5.37E-09 |
| rs147628160 | 9 | 104312162 | A | G | 0.01909 | 0.003408 | 0.000768 | 9.11E-06 |
| rs2420906 | 10 | 122798413 | G | A | 0.592822 | -0.0011 | 0.000212 | 2.03E-07 |
| rs4367899 | 10 | 61920750 | T | C | 0.010304 | 0.005052 | 0.001052 | 1.56E-06 |
| rs10993994 | 10 | 51549496 | C | T | 0.610399 | -0.00176 | 0.000213 | 1.32E-16 |
| rs12799883 | 11 | 69010651 | G | T | 0.507251 | 0.001612 | 0.000208 | 1.06E-14 |
| rs10743182 | 11 | 2229782 | T | C | 0.841997 | -0.00217 | 0.000285 | 3.03E-14 |
| rs17120257 | 12 | 53303331 | C | T | 0.113615 | 0.001839 | 0.00033 | 2.59E-08 |
| rs73200932 | 13 | 67288143 | T | C | 0.130071 | -0.0015 | 0.00032 | 2.84E-06 |
| rs4901309 | 14 | 53380014 | G | A | 0.812778 | 0.001435 | 0.000268 | 8.38E-08 |
| rs78448421 | 14 | 40995362 | C | T | 0.025586 | 0.003205 | 0.000661 | 1.24E-06 |
| rs12433355 | 14 | 70819717 | C | T | 0.501655 | 0.000966 | 0.000209 | 3.68E-06 |
| rs188645944 | 16 | 90083754 | C | T | 0.020105 | 0.003471 | 0.000769 | 6.28E-06 |
| rs148907705 | 17 | 12579132 | T | C | 0.092211 | 0.001872 | 0.000365 | 2.97E-07 |
| rs2474694 | 17 | 618039 | A | G | 0.353796 | 0.000967 | 0.000217 | 8.69E-06 |
| rs7501939 | 17 | 36101156 | C | T | 0.601571 | 0.001642 | 0.000213 | 1.11E-14 |
| rs7217073 | 17 | 69110923 | G | A | 0.53335 | -0.00138 | 0.000208 | 3.05E-11 |
| rs7242302 | 18 | 49139354 | A | G | 0.028851 | 0.002859 | 0.000622 | 4.27E-06 |
| rs4798998 | 18 | 76706687 | A | G | 0.620593 | -0.00107 | 0.000217 | 8.99E-07 |
| rs17632542 | 19 | 51361757 | C | T | 0.073433 | -0.00278 | 0.000398 | 2.89E-12 |
| rs60547573 | 20 | 55650893 | C | T | 0.01868 | 0.003546 | 0.000777 | 5.06E-06 |
| rs113858283 | 20 | 55804981 | G | C | 0.030687 | 0.002842 | 0.000613 | 3.53E-06 |
| rs34677426 | 21 | 42259147 | A | T | 0.065816 | 0.002045 | 0.000424 | 1.41E-06 |
| rs62219627 | 21 | 42734747 | C | G | 0.057109 | 0.002036 | 0.000458 | 8.88E-06 |
| rs134116 | 22 | 28082093 | C | T | 0.231955 | 0.001095 | 0.000247 | 9.70E-06 |
| rs5759167 | 22 | 43500212 | T | G | 0.49922 | -0.00123 | 0.000208 | 3.80E-09 |

Table 63 Instrumental variables of Small intestine cancer

| SNP | Chr | Pos | EA | OA | EAF | Beta | SE | P |
| --- | --- | --- | --- | --- | --- | --- | --- | --- |
| rs114169604 | 1 | 232877756 | T | C | 0.016091 | 0.001053 | 0.000213 | 7.43E-07 |
| rs4846201 | 1 | 9752783 | A | G | 0.006047 | 0.001592 | 0.000345 | 4.01E-06 |
| rs72756730 | 1 | 239691313 | A | G | 0.014336 | 0.001067 | 0.00023 | 3.54E-06 |
| rs114185796 | 1 | 183745993 | A | G | 0.008557 | 0.001583 | 0.000296 | 8.76E-08 |
| rs77131064 | 1 | 67359217 | A | G | 0.010027 | 0.001256 | 0.000279 | 6.96E-06 |
| rs116580423 | 1 | 155306064 | G | A | 0.033129 | 0.000707 | 0.000149 | 1.97E-06 |
| rs12465899 | 2 | 168947814 | G | T | 0.00567 | 0.001678 | 0.000354 | 2.19E-06 |
| rs116322369 | 2 | 158691707 | C | T | 0.01515 | 0.001013 | 0.000214 | 2.28E-06 |
| rs75905773 | 2 | 12642908 | A | T | 0.033927 | 0.000672 | 0.000151 | 8.89E-06 |
| rs147636075 | 2 | 101734199 | G | C | 0.021551 | 0.001032 | 0.000189 | 4.94E-08 |
| rs7604581 | 2 | 158731861 | A | G | 0.011514 | 0.001166 | 0.00025 | 3.06E-06 |
| rs143507767 | 2 | 128434467 | G | A | 0.008726 | 0.001818 | 0.000295 | 7.50E-10 |
| rs116056595 | 2 | 5874623 | T | C | 0.015002 | 0.001138 | 0.000217 | 1.57E-07 |
| rs76241169 | 3 | 113694698 | A | T | 0.005823 | 0.001545 | 0.00035 | 9.91E-06 |
| rs113754712 | 3 | 131397039 | C | A | 0.026593 | 0.000776 | 0.000172 | 6.37E-06 |
| rs6801752 | 3 | 45358054 | T | C | 0.131679 | 0.00035 | 7.78E-05 | 6.83E-06 |
| rs113335353 | 3 | 78658935 | A | G | 0.039403 | 0.000764 | 0.000138 | 2.85E-08 |
| rs9759081 | 3 | 127459754 | T | A | 0.27204 | -0.00028 | 5.96E-05 | 3.61E-06 |
| rs1805632 | 3 | 180699715 | A | G | 0.022524 | 0.000883 | 0.000181 | 1.04E-06 |
| rs149848688 | 3 | 159332958 | T | A | 0.022995 | 0.00092 | 0.000186 | 7.52E-07 |
| rs73068938 | 3 | 32650372 | C | T | 0.009775 | 0.001257 | 0.000279 | 6.70E-06 |
| rs183265654 | 4 | 14478785 | C | T | 0.003751 | 0.002417 | 0.000471 | 2.83E-07 |
| rs183404768 | 4 | 146618125 | A | G | 0.016238 | 0.000945 | 0.000213 | 9.32E-06 |
| rs12644406 | 4 | 149537415 | G | A | 0.245945 | 0.000296 | 6.08E-05 | 1.16E-06 |
| rs73840579 | 4 | 111808635 | G | A | 0.006302 | 0.001748 | 0.000351 | 6.50E-07 |
| rs145074693 | 4 | 164007135 | T | C | 0.020903 | 0.000872 | 0.000188 | 3.40E-06 |
| rs183014129 | 4 | 186529234 | G | C | 0.006808 | 0.001981 | 0.000335 | 3.46E-09 |
| rs16881599 | 5 | 8897205 | A | G | 0.029867 | 0.00075 | 0.000154 | 1.05E-06 |
| rs139663861 | 5 | 115465882 | G | A | 0.007574 | 0.00157 | 0.00032 | 9.50E-07 |
| rs77913843 | 5 | 141740605 | A | G | 0.008692 | 0.001302 | 0.000288 | 6.29E-06 |
| rs112036547 | 5 | 59339224 | A | C | 0.005019 | 0.001934 | 0.000404 | 1.69E-06 |
| rs147027317 | 5 | 15160990 | T | C | 0.002828 | 0.002709 | 0.000539 | 5.15E-07 |
| rs55859855 | 5 | 73518512 | A | G | 0.017149 | 0.001126 | 0.000215 | 1.54E-07 |
| rs114561358 | 5 | 56177369 | C | T | 0.011559 | 0.001114 | 0.000252 | 9.68E-06 |
| rs17599432 | 5 | 136297436 | C | A | 0.015005 | 0.001137 | 0.000215 | 1.32E-07 |
| rs75020146 | 6 | 14390517 | T | C | 0.011726 | 0.001193 | 0.000243 | 8.87E-07 |
| rs189528308 | 6 | 87861047 | A | T | 0.007158 | 0.001526 | 0.000327 | 3.18E-06 |
| rs187402560 | 6 | 165653604 | G | T | 0.004828 | 0.001718 | 0.000385 | 8.10E-06 |
| rs9267989 | 6 | 32219320 | T | G | 0.21377 | 0.000284 | 6.38E-05 | 8.35E-06 |
| rs145192708 | 6 | 86605453 | G | T | 0.01034 | 0.001457 | 0.000265 | 3.85E-08 |
| rs115062836 | 6 | 65672841 | G | A | 0.022994 | 0.000847 | 0.000175 | 1.25E-06 |
| rs17178873 | 7 | 81970257 | G | A | 0.021232 | 0.000888 | 0.00019 | 2.77E-06 |
| rs73119674 | 7 | 46520336 | T | G | 0.013041 | 0.001089 | 0.00024 | 5.75E-06 |
| rs62476009 | 7 | 121951449 | C | T | 0.050976 | 0.000589 | 0.000119 | 7.62E-07 |
| rs28669921 | 8 | 15948983 | T | C | 0.021406 | 0.000839 | 0.000185 | 5.59E-06 |
| rs76536680 | 8 | 130644306 | C | T | 0.025621 | 0.000841 | 0.000168 | 5.46E-07 |
| rs111933723 | 8 | 34280860 | T | C | 0.007883 | 0.001455 | 0.000305 | 1.89E-06 |
| rs148383135 | 8 | 19738408 | A | G | 0.013068 | 0.001112 | 0.000236 | 2.38E-06 |
| rs117295012 | 8 | 138204772 | G | A | 0.011177 | 0.001266 | 0.000249 | 3.61E-07 |
| rs117745518 | 8 | 58618851 | A | G | 0.014282 | 0.000997 | 0.000221 | 6.34E-06 |
| rs78596460 | 8 | 106368058 | A | G | 0.007439 | 0.001392 | 0.00031 | 6.92E-06 |
| rs80156155 | 8 | 32757540 | G | C | 0.011336 | 0.001093 | 0.000247 | 9.86E-06 |
| rs12544923 | 8 | 39887665 | T | C | 0.296954 | 0.000261 | 5.74E-05 | 5.60E-06 |
| rs12337274 | 9 | 104926856 | G | C | 0.02383 | 0.000913 | 0.000179 | 3.34E-07 |
| rs142372868 | 9 | 123541486 | A | C | 0.007254 | 0.001659 | 0.000317 | 1.67E-07 |
| rs77933629 | 9 | 113137872 | T | C | 0.034804 | 0.000828 | 0.000143 | 7.46E-09 |
| rs112360973 | 9 | 89993526 | G | A | 0.018259 | 0.000874 | 0.000197 | 9.51E-06 |
| rs1318660 | 9 | 138591684 | T | G | 0.01156 | 0.001099 | 0.000245 | 7.45E-06 |
| rs57398751 | 10 | 73362389 | T | C | 0.001527 | 0.003441 | 0.000674 | 3.24E-07 |
| rs145268931 | 10 | 99395875 | A | T | 0.007009 | 0.001546 | 0.00035 | 9.97E-06 |
| rs117576372 | 10 | 33031234 | G | A | 0.023428 | 0.000823 | 0.000184 | 7.85E-06 |
| rs10796071 | 10 | 13410425 | G | A | 0.343758 | 0.000256 | 5.53E-05 | 3.80E-06 |
| rs112525324 | 10 | 112881376 | T | C | 0.00733 | 0.001407 | 0.000317 | 9.09E-06 |
| rs149803047 | 10 | 98903880 | A | G | 0.017426 | 0.000911 | 0.0002 | 5.44E-06 |
| rs61861336 | 10 | 105994929 | G | A | 0.045623 | 0.000567 | 0.000126 | 7.15E-06 |
| rs17232129 | 10 | 131921200 | C | T | 0.372196 | 0.000244 | 5.42E-05 | 7.10E-06 |
| rs74594145 | 11 | 10027536 | C | T | 0.032109 | 0.000666 | 0.000148 | 7.20E-06 |
| rs139086999 | 11 | 67466412 | T | C | 0.010093 | 0.001237 | 0.000263 | 2.65E-06 |
| rs182604304 | 11 | 60079412 | A | T | 0.028365 | 0.000872 | 0.000161 | 5.91E-08 |
| rs147045808 | 11 | 92278503 | T | C | 0.011487 | 0.001269 | 0.000268 | 2.13E-06 |
| rs138146706 | 11 | 104557061 | G | A | 0.011922 | 0.001182 | 0.000253 | 2.92E-06 |
| rs7101362 | 11 | 83769803 | A | T | 0.001585 | 0.003069 | 0.000666 | 3.99E-06 |
| rs143743548 | 11 | 1826210 | C | G | 0.004837 | 0.001745 | 0.000385 | 5.82E-06 |
| rs4964441 | 12 | 104401790 | A | C | 0.014947 | 0.001116 | 0.000217 | 2.64E-07 |
| rs77494407 | 12 | 16140880 | G | C | 0.018849 | 0.000929 | 0.000193 | 1.51E-06 |
| rs2124525 | 12 | 127450064 | T | C | 0.007651 | 0.001456 | 0.000305 | 1.87E-06 |
| rs117550779 | 13 | 36107579 | G | A | 0.023044 | 0.000854 | 0.000176 | 1.18E-06 |
| rs149079340 | 13 | 19649218 | T | C | 0.007261 | 0.001469 | 0.000318 | 3.87E-06 |
| rs118135775 | 14 | 24016873 | G | A | 0.02898 | 0.000729 | 0.000156 | 3.03E-06 |
| rs72548756 | 14 | 64714376 | T | A | 0.014361 | 0.001052 | 0.000229 | 4.30E-06 |
| rs1873285 | 15 | 29256096 | G | A | 0.10258 | 0.000383 | 8.66E-05 | 9.88E-06 |
| rs144020426 | 15 | 83749559 | T | C | 0.001782 | 0.002894 | 0.000629 | 4.17E-06 |
| rs148330060 | 16 | 88051655 | A | C | 0.007651 | 0.001503 | 0.000316 | 1.98E-06 |
| rs45588737 | 16 | 52545335 | T | C | 0.04135 | 0.000712 | 0.000133 | 9.42E-08 |
| rs117009973 | 16 | 75696092 | G | A | 0.013175 | 0.001221 | 0.000236 | 2.34E-07 |
| rs117026454 | 16 | 27365046 | T | C | 0.014281 | 0.001125 | 0.000227 | 6.93E-07 |
| rs73524323 | 16 | 31626439 | G | A | 0.01308 | 0.001038 | 0.000232 | 7.80E-06 |
| rs117088049 | 16 | 89185124 | A | G | 0.0102 | 0.001284 | 0.000261 | 8.41E-07 |
| rs8067786 | 17 | 69085644 | G | A | 0.484527 | -0.00024 | 5.24E-05 | 7.30E-06 |
| rs142969277 | 17 | 13457275 | T | C | 0.014357 | 0.001 | 0.000226 | 9.97E-06 |
| rs470627 | 18 | 63033284 | C | A | 0.159066 | 0.000378 | 7.27E-05 | 2.02E-07 |
| rs150327154 | 18 | 36809535 | C | T | 0.005305 | 0.001891 | 0.000368 | 2.74E-07 |
| rs58680865 | 18 | 76493101 | G | A | 0.009773 | 0.001198 | 0.000268 | 7.59E-06 |
| rs72877778 | 18 | 14051288 | T | C | 0.012387 | 0.001099 | 0.000237 | 3.44E-06 |
| rs185609235 | 18 | 73170821 | T | C | 0.007279 | 0.001392 | 0.000315 | 9.78E-06 |
| rs185400394 | 19 | 41107428 | T | C | 0.005455 | 0.001657 | 0.000371 | 7.93E-06 |
| rs78201345 | 19 | 29754371 | A | C | 0.022822 | 0.000858 | 0.000177 | 1.19E-06 |
| rs8116420 | 20 | 56178612 | T | A | 0.04183 | 0.000629 | 0.000139 | 6.47E-06 |
| rs6125211 | 20 | 46663069 | A | G | 0.001738 | 0.003009 | 0.000636 | 2.26E-06 |
| rs62201808 | 20 | 60110469 | C | T | 0.119943 | 0.0004 | 8.09E-05 | 7.92E-07 |
| rs75861798 | 20 | 61973438 | T | C | 0.003823 | 0.002233 | 0.000446 | 5.55E-07 |
| rs111506410 | 20 | 1121937 | C | T | 0.037072 | 0.000678 | 0.000139 | 9.99E-07 |
| rs117451086 | 20 | 46565141 | T | C | 0.006008 | 0.001726 | 0.000367 | 2.60E-06 |
| rs151187949 | 21 | 32831557 | G | T | 0.038292 | 0.00063 | 0.000142 | 8.89E-06 |
| rs1211089 | 21 | 46016189 | T | C | 0.954408 | -0.00058 | 0.000126 | 4.99E-06 |
| rs13050657 | 21 | 35570840 | C | G | 0.049994 | 0.000542 | 0.000122 | 8.67E-06 |
| rs73175074 | 22 | 46351856 | A | G | 0.009156 | 0.001321 | 0.000277 | 1.83E-06 |
| rs113082525 | 22 | 36570383 | A | T | 0.004699 | 0.001939 | 0.000433 | 7.34E-06 |
| rs140882974 | 22 | 41599509 | G | A | 0.009443 | 0.001311 | 0.000277 | 2.18E-06 |

Table 64 Instrumental variables of Squamous cell carcinoma

| SNP | Chr | Pos | EA | OA | EAF | Beta | SE | P |
| --- | --- | --- | --- | --- | --- | --- | --- | --- |
| rs12078420 | 1 | 171617827 | A | G | 0.001862 | 0.004716 | 0.00099 | 1.91E-06 |
| rs12030145 | 1 | 24478518 | A | T | 0.187131 | -0.00051 | 0.000108 | 3.11E-06 |
| rs78101357 | 1 | 87943932 | A | G | 0.010334 | 0.001995 | 0.000419 | 1.94E-06 |
| rs145321247 | 1 | 114875774 | A | C | 0.020018 | 0.001412 | 0.000316 | 8.10E-06 |
| rs116023898 | 1 | 201648818 | G | A | 0.013081 | 0.001719 | 0.000375 | 4.66E-06 |
| rs11263844 | 1 | 35410558 | C | A | 0.053792 | 0.000917 | 0.000192 | 1.70E-06 |
| rs1344663 | 2 | 137169613 | C | T | 0.151053 | 0.000557 | 0.000118 | 2.23E-06 |
| rs115039184 | 2 | 55733511 | G | T | 0.013683 | 0.001647 | 0.000363 | 5.69E-06 |
| rs116434775 | 2 | 103968988 | C | T | 0.016596 | 0.001476 | 0.000334 | 9.61E-06 |
| rs55758632 | 3 | 197126313 | G | C | 0.163513 | 0.000555 | 0.000114 | 1.23E-06 |
| rs115093902 | 3 | 134871028 | T | G | 0.012994 | 0.001672 | 0.000371 | 6.75E-06 |
| rs146329931 | 3 | 72856284 | A | T | 0.022184 | 0.001413 | 0.000302 | 2.90E-06 |
| rs147325936 | 3 | 118040491 | A | G | 0.012734 | 0.00183 | 0.000403 | 5.70E-06 |
| rs61763247 | 3 | 154832445 | A | G | 0.009567 | 0.001932 | 0.000432 | 7.74E-06 |
| rs74959196 | 3 | 14608558 | C | T | 0.01458 | 0.001601 | 0.000352 | 5.42E-06 |
| rs143510187 | 3 | 35868678 | A | G | 0.010876 | 0.001978 | 0.000426 | 3.40E-06 |
| rs78587275 | 4 | 62496970 | G | T | 0.013532 | 0.001892 | 0.000365 | 2.10E-07 |
| rs11933135 | 4 | 83668410 | A | G | 0.108688 | -0.00065 | 0.000136 | 1.93E-06 |
| rs111787276 | 4 | 138516752 | G | C | 0.04135 | 0.000962 | 0.000214 | 6.78E-06 |
| rs186716334 | 4 | 23297549 | C | T | 0.002319 | 0.00434 | 0.000914 | 2.03E-06 |
| rs151175483 | 5 | 7460418 | C | T | 0.003786 | 0.003291 | 0.000706 | 3.16E-06 |
| rs62338630 | 5 | 5406493 | C | T | 0.079425 | 0.000894 | 0.000158 | 1.58E-08 |
| rs17103741 | 5 | 145161670 | G | A | 0.00887 | 0.002141 | 0.000451 | 2.09E-06 |
| rs7773711 | 6 | 30664989 | T | A | 0.007481 | 0.002404 | 0.000489 | 8.87E-07 |
| rs118147801 | 6 | 162062131 | T | C | 0.013258 | 0.001771 | 0.000393 | 6.59E-06 |
| rs77529239 | 6 | 143733856 | C | T | 0.021164 | 0.001624 | 0.000293 | 2.85E-08 |
| rs62414043 | 6 | 94898625 | C | T | 0.017149 | 0.001596 | 0.000341 | 2.81E-06 |
| rs141071381 | 6 | 104411249 | A | G | 0.014969 | 0.001821 | 0.000359 | 4.08E-07 |
| rs80186674 | 7 | 66478167 | T | G | 0.127932 | 0.000673 | 0.000136 | 7.27E-07 |
| rs2972121 | 7 | 147875438 | G | T | 0.205469 | -0.00048 | 0.000105 | 5.21E-06 |
| rs73207416 | 7 | 111580877 | C | T | 0.076201 | 0.000736 | 0.00016 | 4.44E-06 |
| rs72724750 | 8 | 129297915 | C | G | 0.040364 | 0.001023 | 0.00023 | 8.43E-06 |
| rs144323548 | 8 | 1194948 | A | G | 0.005921 | 0.003081 | 0.000581 | 1.16E-07 |
| rs564516009 | 8 | 69202095 | A | T | 0.004047 | 0.003628 | 0.000727 | 6.07E-07 |
| rs117551353 | 9 | 28754685 | A | G | 0.046412 | 0.000947 | 0.000205 | 3.69E-06 |
| rs188483350 | 9 | 20926669 | G | C | 0.002653 | 0.004721 | 0.000859 | 3.94E-08 |
| rs148794554 | 9 | 95157104 | T | C | 0.01234 | 0.001851 | 0.000383 | 1.37E-06 |
| rs184275325 | 10 | 61916984 | G | A | 0.013124 | 0.001726 | 0.000374 | 3.98E-06 |
| rs76332748 | 10 | 133937819 | T | C | 0.096623 | 0.000689 | 0.000142 | 1.32E-06 |
| rs117061060 | 10 | 95363098 | G | A | 0.018645 | 0.001888 | 0.000322 | 4.50E-09 |
| rs1885824 | 10 | 77632534 | G | A | 0.200939 | 0.000466 | 0.000105 | 9.65E-06 |
| rs11256840 | 10 | 10847278 | C | T | 0.072219 | 0.00072 | 0.000163 | 9.82E-06 |
| rs9666616 | 11 | 110725831 | A | C | 0.247781 | -0.00043 | 9.77E-05 | 8.61E-06 |
| rs117062315 | 12 | 11846864 | T | C | 0.0121 | 0.001785 | 0.000385 | 3.59E-06 |
| rs117911607 | 13 | 36787789 | A | G | 0.076356 | 0.000716 | 0.000161 | 8.43E-06 |
| rs186637720 | 15 | 95728369 | A | G | 0.009732 | 0.00211 | 0.000444 | 2.00E-06 |
| rs75482474 | 15 | 97384396 | T | C | 0.012294 | 0.001856 | 0.000406 | 4.87E-06 |
| rs11857482 | 15 | 60350887 | T | C | 0.044597 | 0.000945 | 0.000207 | 4.99E-06 |
| rs77676522 | 16 | 27042257 | T | C | 0.014577 | 0.00171 | 0.000369 | 3.58E-06 |
| rs150216370 | 16 | 51249965 | C | A | 0.008455 | 0.002204 | 0.000488 | 6.35E-06 |
| rs145537232 | 16 | 60962804 | T | C | 0.015793 | 0.001633 | 0.000344 | 2.02E-06 |
| rs74787028 | 16 | 9675268 | T | C | 0.137732 | -0.00055 | 0.000124 | 7.48E-06 |
| rs75665989 | 16 | 87211260 | G | A | 0.04671 | 0.000952 | 0.0002 | 1.84E-06 |
| rs141635352 | 17 | 45879416 | G | A | 0.026041 | 0.001292 | 0.000276 | 2.83E-06 |
| rs142125725 | 17 | 11853699 | A | G | 0.005632 | 0.003069 | 0.000604 | 3.79E-07 |
| rs80082632 | 17 | 51130807 | A | G | 0.032562 | 0.001215 | 0.000245 | 7.06E-07 |
| rs117571779 | 17 | 4150464 | T | G | 0.016262 | 0.00154 | 0.000346 | 8.76E-06 |
| rs189046811 | 17 | 2193194 | G | A | 0.012103 | 0.001749 | 0.000393 | 8.68E-06 |
| rs141012781 | 18 | 7094553 | T | C | 0.034474 | 0.001198 | 0.000244 | 9.47E-07 |
| rs146650419 | 18 | 4783442 | T | G | 0.009659 | 0.002257 | 0.000462 | 1.03E-06 |
| rs12961221 | 18 | 75779187 | T | A | 0.340708 | -0.0004 | 8.97E-05 | 8.75E-06 |
| rs72960526 | 18 | 73777891 | C | T | 0.006742 | 0.002498 | 0.000539 | 3.56E-06 |
| rs72870324 | 18 | 5622068 | A | T | 0.013876 | 0.001955 | 0.000371 | 1.39E-07 |
| rs12610105 | 19 | 44509403 | G | A | 0.14574 | 0.00059 | 0.00012 | 8.54E-07 |
| rs113554316 | 20 | 13570123 | G | C | 0.003749 | 0.003634 | 0.000695 | 1.70E-07 |
| rs138622955 | 20 | 2637565 | A | G | 0.011791 | 0.001856 | 0.000401 | 3.71E-06 |
| rs17810651 | 20 | 60333519 | A | G | 0.081975 | 0.000692 | 0.000154 | 7.19E-06 |
| rs148000396 | 22 | 36509709 | A | G | 0.005862 | 0.002762 | 0.000588 | 2.69E-06 |
| rs143950883 | 22 | 48545321 | G | T | 0.014139 | 0.001834 | 0.000389 | 2.39E-06 |

S3. Mendelian randomization analysis of periodontitis and 20 cancers.

**Table 1** Results of Mendelian randomization analysis

**Table 2** Sensitivity analysis of the Mendelian randomization analysis results

**Figure 1-20** Results and sensitivity analyses plots of the genetic correlation between periodontitis and 20 cancers

**Table 1 Results of Mendelian randomization analysis**

| **Outcome Exposure** | **Method** | **Periodontitis** | | |
| --- | --- | --- | --- | --- |
|  |  | **SNP (n)** | **OR** | ***P* Value** |
|  | **MR Egger** | 11 | 0.936 | 0.540 |
|  | **Weighted median** | 11 | 0.967 | 0.501 |
| **Basal cell carcinoma** | **IVW** | 11 | 1.004 | 0.924 |
|  | **Simple mode** | 11 | 0.939 | 0.421 |
|  | **Weighted mode** | 11 | 0.954 | 0.502 |
|  | **MR Egger** | 15 | 1.000 | 0.901 |
|  | **Weighted median** | 15 | 1.000 | 0.912 |
| **Bladder cancer** | **IVW** | 15 | 1.000 | 0.876 |
|  | **Simple mode** | 15 | 1.000 | 0.693 |
|  | **Weighted mode** | 15 | 1.000 | 0.872 |
|  | **MR Egger** | 11 | 1.000 | 0.930 |
|  | **Weighted median** | 11 | 1.000 | 0.864 |
| **Brain cancer** | **IVW** | 11 | 1.000 | 0.754 |
|  | **Simple mode** | 11 | 1.000 | 0.676 |
|  | **Weighted mode** | 11 | 1.000 | 0.888 |
|  | **MR Egger** | 12 | 0.999 | 0.696 |
|  | **Weighted median** | 12 | 1.002 | 0.309 |
| **Breast cancer** | **IVW** | 12 | 1.002 | 0.060 |
|  | **Simple mode** | 12 | 1.001 | 0.747 |
|  | **Weighted mode** | 12 | 1.001 | 0.640 |
|  | **MR Egger** | 16 | 1.001 | 0.226 |
|  | **Weighted median** | 16 | 1.000 | 0.980 |
| **Cancer of urinary tract** | **IVW** | 16 | 1.000 | 0.976 |
|  | **Simple mode** | 16 | 1.000 | 0.978 |
|  | **Weighted mode** | 16 | 1.001 | 0.674 |
|  | **MR Egger** | 11 | 0.999 | 0.388 |
|  | **Weighted median** | 11 | 0.999 | 0.467 |
| **Cervical cancer** | **IVW** | 11 | 0.999 | 0.207 |
|  | **Simple mode** | 11 | 1.001 | 0.631 |
|  | **Weighted mode** | 11 | 1.000 | 0.690 |
|  | **MR Egger** | 15 | 1.001 | 0.587 |
|  | **Weighted median** | 15 | 1.001 | 0.468 |
| **Colorectal cancer** | **IVW** | 15 | 1.001 | 0.217 |
|  | **Simple mode** | 15 | 1.001 | 0.508 |
|  | **Weighted mode** | 15 | 1.002 | 0.359 |
|  | **MR Egger** | 15 | 1.080 | 0.422 |
|  | **Weighted median** | 15 | 1.111 | 0.097 |
| **Endometrial cancer** | **IVW** | 15 | 1.088 | 0.062 |
|  | **Simple mode** | 15 | 1.153 | 0.265 |
|  | **Weighted mode** | 15 | 1.161 | 0.249 |
|  | **MR Egger** | 9 | 1.151 | 0.420 |
|  | **Weighted median** | 9 | 1.087 | 0.288 |
| **Gastric cancer** | **IVW** | 9 | 1.074 | 0.244 |
|  | **Simple mode** | 9 | 1.125 | 0.306 |
|  | **Weighted mode** | 9 | 1.101 | 0.272 |
|  | **MR Egger** | 15 | 0.999 | 0.380 |
|  | **Weighted median** | 15 | 0.999 | 0.101 |
| **Head and neck cancer** | **IVW** | 15 | 0.999 | 0.041 |
|  | **Simple mode** | 15 | 0.999 | 0.317 |
|  | **Weighted mode** | 15 | 0.999 | 0.319 |
|  | **MR Egger** | 9 | 1.000 | 0.622 |
|  | **Weighted median** | 9 | 1.000 | 0.709 |
| **Liver & bile duct cancer** | **IVW** | 9 | 1.000 | 0.785 |
|  | **Simple mode** | 9 | 1.000 | 0.728 |
|  | **Weighted mode** | 9 | 1.000 | 0.965 |
|  | **MR Egger** | 15 | 1.001 | 0.665 |
|  | **Weighted median** | 15 | 1.000 | 0.778 |
| **Lung cancer** | **IVW** | 15 | 1.000 | 0.633 |
|  | **Simple mode** | 15 | 1.000 | 0.943 |
|  | **Weighted mode** | 15 | 1.000 | 0.926 |
|  | **MR Egger** | 15 | 0.998 | 0.651 |
|  | **Weighted median** | 15 | 0.998 | 0.317 |
| **Malignant non-melanoma skin cancer** | **IVW** | 15 | 1.001 | 0.714 |
|  | **Simple mode** | 15 | 0.998 | 0.508 |
|  | **Weighted mode** | 15 | 0.998 | 0.486 |
|  | **MR Egger** | 15 | 1.000 | 0.952 |
|  | **Weighted median** | 15 | 0.999 | 0.455 |
| **Melanoma skin cancer** | **IVW** | 15 | 1.000 | 0.648 |
|  | **Simple mode** | 15 | 0.999 | 0.456 |
|  | **Weighted mode** | 15 | 0.999 | 0.379 |
|  | **MR Egger** | 12 | 0.999 | 0.427 |
|  | **Weighted median** | 12 | 1.000 | 0.976 |
| **Oesophageal cancer** | **IVW** | 12 | 1.000 | 0.738 |
|  | **Simple mode** | 12 | 1.000 | 0.784 |
|  | **Weighted mode** | 12 | 1.000 | 0.943 |
|  | **MR Egger** | 15 | 0.998 | 0.494 |
|  | **Weighted median** | 15 | 1.000 | 0.717 |
| **Ovarian cancer** | **IVW** | 15 | 1.000 | 0.873 |
|  | **Simple mode** | 15 | 0.999 | 0.604 |
|  | **Weighted mode** | 15 | 1.000 | 0.777 |
|  | **MR Egger** | 9 | 0.322 | 0.148 |
|  | **Weighted median** | 9 | 0.974 | 0.937 |
| **Pancreatic cancer** | **IVW** | 9 | 1.021 | 0.943 |
|  | **Simple mode** | 9 | 1.648 | 0.472 |
|  | **Weighted mode** | 9 | 0.824 | 0.627 |
|  | **MR Egger** | 12 | 0.999 | 0.363 |
|  | **Weighted median** | 12 | 0.999 | 0.300 |
| **Prostate cancer** | **IVW** | 12 | 0.999 | 0.431 |
|  | **Simple mode** | 12 | 0.998 | 0.332 |
|  | **Weighted mode** | 12 | 0.998 | 0.291 |
|  | **MR Egger** | 12 | 1.000 | 0.621 |
|  | **Weighted median** | 12 | 1.000 | 0.952 |
| **Small intestine cancer** | **IVW** | 12 | 1.000 | 0.474 |
|  | **Simple mode** | 12 | 1.000 | 0.819 |
|  | **Weighted mode** | 12 | 1.000 | 0.746 |
|  | **MR Egger** | 12 | 1.000 | 0.967 |
|  | **Weighted median** | 12 | 1.000 | 0.341 |
| **Squamous cell carcinoma** | **IVW** | 12 | 1.000 | 0.074 |
|  | **Simple mode** | 12 | 1.000 | 0.609 |
|  | **Weighted mode** | 12 | 1.000 | 0.570 |

**Table2 Sensitivity analysis of the Mendelian randomization analysis results**

| **Outcome Exposure** | **Method** | | **Periodontitis** | |
| --- | --- | --- | --- | --- |
|  | **IVW (heterogeneity)** | ***p* value** | 0.175 |  |
|  |  | **Q** | 13.954 |  |
| **Basal cell carcinoma** | **MR Egger (heterogeneity)** | ***p* value** | 0.157 |  |
|  |  | **Q** | 13.126 |  |
|  | **MR Egger (pleiotropy)** | ***p* value** | 0.470 |  |
|  |  | **intercept** | 0.012 |  |
|  | **IVW (heterogeneity)** | ***p* value** | 0.754 |  |
|  |  | **Q** | 10.118 |  |
| **Bladder cancer** | **MR Egger (heterogeneity)** | ***p* value** | 0.685 |  |
|  |  | **Q** | 10.114 |  |
|  | **MR Egger (pleiotropy)** | ***p* value** | 0.954 |  |
|  |  | **intercept** | 0.000 |  |
|  | **IVW (heterogeneity)** | ***p* value** | 0.815 |  |
|  |  | **Q** | 5.998 |  |
| **Brain cancer** | **MR Egger (heterogeneity)** | ***p* value** | 0.740 |  |
|  |  | **Q** | 5.995 |  |
|  | **MR Egger (pleiotropy)** | ***p* value** | 0.960 |  |
|  |  | **intercept** | 0.000 |  |
|  | **IVW (heterogeneity)** | ***p* value** | 0.509 |  |
|  |  | **Q** | 10.234 |  |
| **Breast cancer** | **MR Egger (heterogeneity)** | ***p* value** | 0.605 |  |
|  |  | **Q** | 8.247 |  |
|  | **MR Egger (pleiotropy)** | ***p* value** | 0.189 |  |
|  |  | **intercept** | 0.001 |  |
|  | **IVW (heterogeneity)** | ***p* value** | 0.088 |  |
|  |  | **Q** | 22.834 |  |
| **Cancer of urinary tract** | **MR Egger (heterogeneity)** | ***p* value** | 0.134 |  |
|  |  | **Q** | 19.874 |  |
|  | **MR Egger (pleiotropy)** | ***p* value** | 0.171 |  |
|  |  | **intercept** | 0.000 |  |
|  | **IVW (heterogeneity)** | ***p* value** | 0.516 |  |
|  |  | **Q** | 9.173 |  |
| **Cervical cancer** | **MR Egger (heterogeneity)** | ***p* value** | 0.436 |  |
|  |  | **Q** | 9.014 |  |
|  | **MR Egger (pleiotropy)** | ***p* value** | 0.700 |  |
|  |  | **intercept** | 0.000 |  |
|  | **IVW (heterogeneity)** | ***p* value** | 0.320 |  |
|  |  | **Q** | 15.899 |  |
| **Colorectal cancer** | **MR Egger (heterogeneity)** | ***p* value** | 0.255 |  |
|  |  | **Q** | 15.899 |  |
|  | **MR Egger (pleiotropy)** | ***p* value** | 0.982 |  |
|  |  | **intercept** | 0.000 |  |
|  | **IVW (heterogeneity)** | ***p* value** | 0.624 |  |
|  |  | **Q** | 11.779 |  |
| **Endometrial cancer** | **MR Egger (heterogeneity)** | ***p* value** | 0.546 |  |
|  |  | **Q** | 11.771 |  |
|  | **MR Egger (pleiotropy)** | ***p* value** | 0.933 |  |
|  |  | **intercept** | 0.001 |  |
|  | **IVW (heterogeneity)** | ***p* value** | 0.843 |  |
|  |  | **Q** | 4.151 |  |
| **Gastric cancer** | **MR Egger (heterogeneity)** | ***p* value** | 0.786 |  |
|  |  | **Q** | 3.944 |  |
|  | **MR Egger (pleiotropy)** | ***p* value** | 0.663 |  |
|  |  | **intercept** | -0.010 |  |
|  | **IVW (heterogeneity)** | ***p* value** | 0.963 |  |
|  |  | **Q** | 6.144 |  |
| **Head and neck cancer** | **MR Egger (heterogeneity)** | ***p* value** | 0.941 |  |
|  |  | **Q** | 6.135 |  |
|  | **MR Egger (pleiotropy)** | ***p* value** | 0.929 |  |
|  |  | **intercept** | 0.000 |  |
|  | **IVW (heterogeneity)** | ***p* value** | 0.673 |  |
|  |  | **Q** | 5.773 |  |
| **Liver & bile duct cancer** | **MR Egger (heterogeneity)** | ***p* value** | 0.623 |  |
|  |  | **Q** | 5.301 |  |
|  | **MR Egger (pleiotropy)** | ***p* value** | 0.514 |  |
|  |  | **intercept** | 0.000 |  |
|  | **IVW (heterogeneity)** | ***p* value** | 0.765 |  |
|  |  | **Q** | 9.963 |  |
| **Lung cancer** | **MR Egger (heterogeneity)** | ***p* value** | 0.702 |  |
|  |  | **Q** | 9.904 |  |
|  | **MR Egger (pleiotropy)** | ***p* value** | 0.812 |  |
|  |  | **intercept** | 0.000 |  |
|  | **IVW (heterogeneity)** | ***p* value** | 0.301 |  |
|  |  | **Q** | 16.200 |  |
| **Malignant non-melanoma skin cancer** | **MR Egger (heterogeneity)** | ***p* value** | 0.273 |  |
|  |  | **Q** | 15.565 |  |
|  | **MR Egger (pleiotropy)** | ***p* value** | 0.479 |  |
|  |  | **intercept** | 0.000 |  |
|  | **IVW (heterogeneity)** | ***p* value** | 0.050 |  |
|  |  | **Q** | 23.649 |  |
| **Melanoma skin cancer** | **MR Egger (heterogeneity)** | ***p* value** | 0.036 |  |
|  |  | **Q** | 23.470 |  |
|  | **MR Egger (pleiotropy)** | ***p* value** | 0.758 |  |
|  |  | **intercept** | 0.000 |  |
|  | **IVW (heterogeneity)** | ***p* value** | 0.349 |  |
|  |  | **Q** | 12.202 |  |
| **Oesophageal cancer** | **MR Egger (heterogeneity)** | ***p* value** | 0.317 |  |
|  |  | **Q** | 11.535 |  |
|  | **MR Egger (pleiotropy)** | ***p* value** | 0.465 |  |
|  |  | **intercept** | 0.000 |  |
|  | **IVW (heterogeneity)** | ***p* value** | 0.009 |  |
|  |  | **Q** | 29.593 |  |
| **Ovarian cancer** | **MR Egger (heterogeneity)** | ***p* value** | 0.009 |  |
|  |  | **Q** | 27.882 |  |
|  | **MR Egger (pleiotropy)** | ***p* value** | 0.388 |  |
|  |  | **intercept** | 0.000 |  |
|  | **IVW (heterogeneity)** | ***p* value** | 0.108 |  |
|  |  | **Q** | 13.128 |  |
| **Pancreatic cancer** | **MR Egger (heterogeneity)** | ***p* value** | 0.252 |  |
|  |  | **Q** | 9.015 |  |
|  | **MR Egger (pleiotropy)** | ***p* value** | 0.117 |  |
|  |  | **intercept** | 0.162 |  |
|  | **IVW (heterogeneity)** | ***p* value** | 0.850 |  |
|  |  | **Q** | 6.343 |  |
| **Prostate cancer** | **MR Egger (heterogeneity)** | ***p* value** | 0.824 |  |
|  |  | **Q** | 5.897 |  |
|  | **MR Egger (pleiotropy)** | ***p* value** | 0.519 |  |
|  |  | **intercept** | 0.000 |  |
|  | **IVW (heterogeneity)** | ***p* value** | 0.582 |  |
|  |  | **Q** | 9.437 |  |
| **Small intestine cancer** | **MR Egger (heterogeneity)** | ***p* value** | 0.495 |  |
|  |  | **Q** | 9.394 |  |
|  | **MR Egger (pleiotropy)** | ***p* value** | 0.839 |  |
|  |  | **intercept** | 0.000 |  |
|  | **IVW (heterogeneity)** | ***p* value** | 0.922 |  |
|  |  | **Q** | 5.176 |  |
| **Squamous cell carcinoma** | **MR Egger (heterogeneity)** | ***p* value** | 0.926 |  |
|  |  | **Q** | 4.432 |  |
|  | **MR Egger (pleiotropy)** | ***p* value** | 0.408 |  |
|  |  | **intercept** | 0.000 |  |


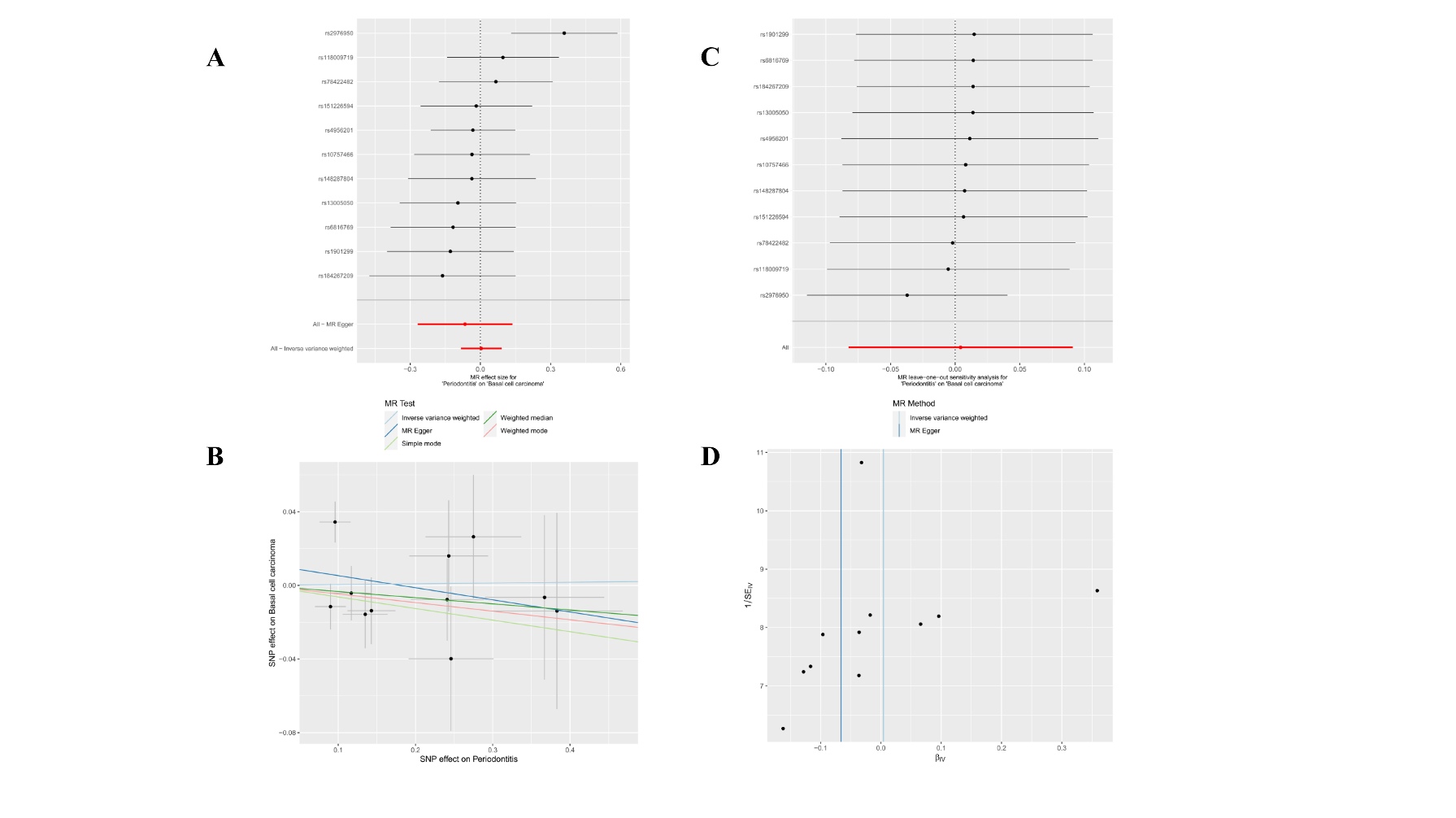


**Figure 1 Results and sensitivity analyses of the genetic correlation between periodontitis and basal cell carcinoma plotted in (A) forest plot; ( B ) scatterplot; (C) Leave-one-out sensitivity test; and (D) funnel plot.**

**
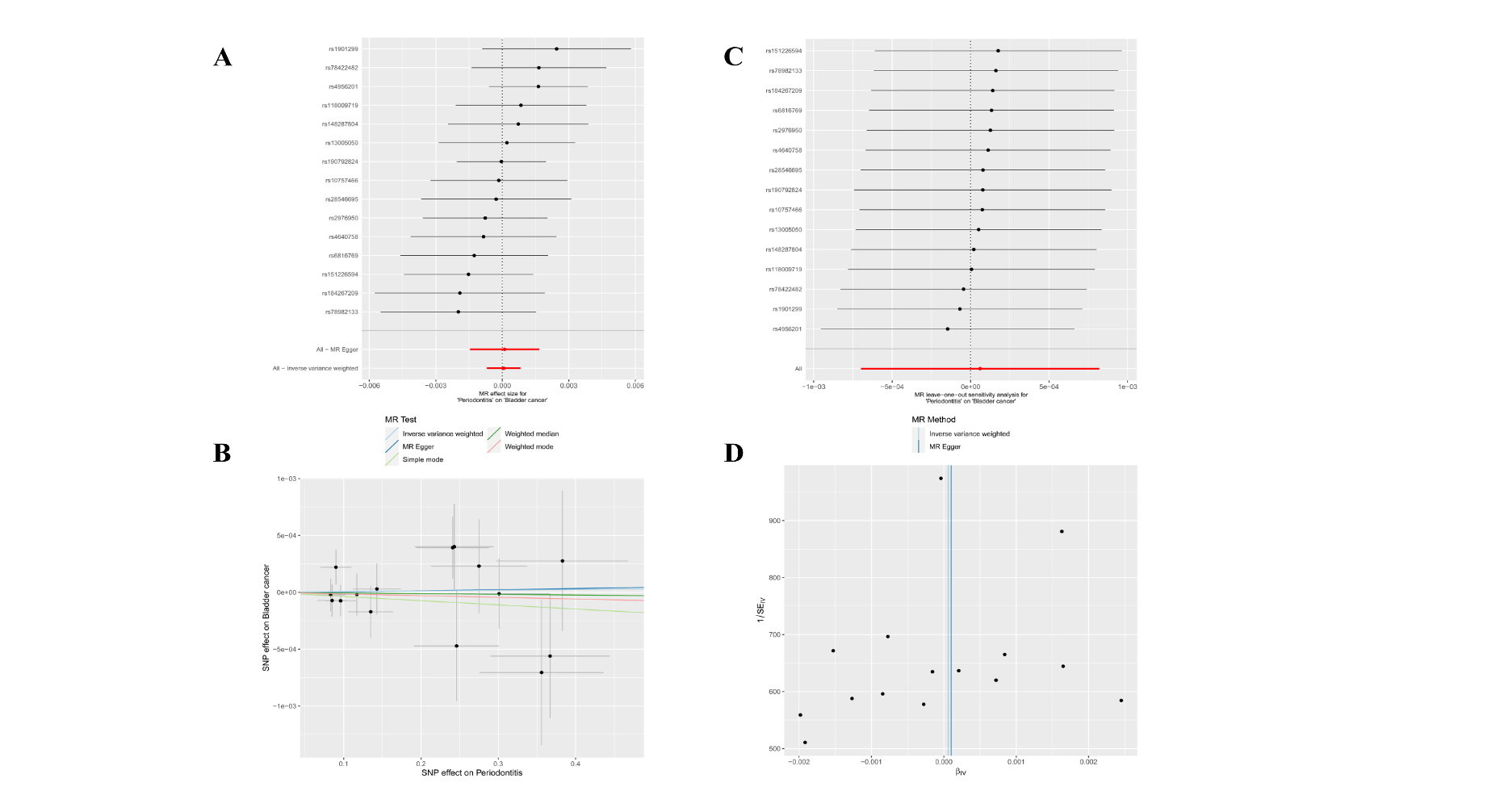
**

**Figure 2 Results and sensitivity analyses of the genetic correlation between periodontitis and bladder cancer plotted in (A) forest plot; ( B ) scatterplot; (C) Leave-one-out sensitivity test; and (D) funnel plot.**

**
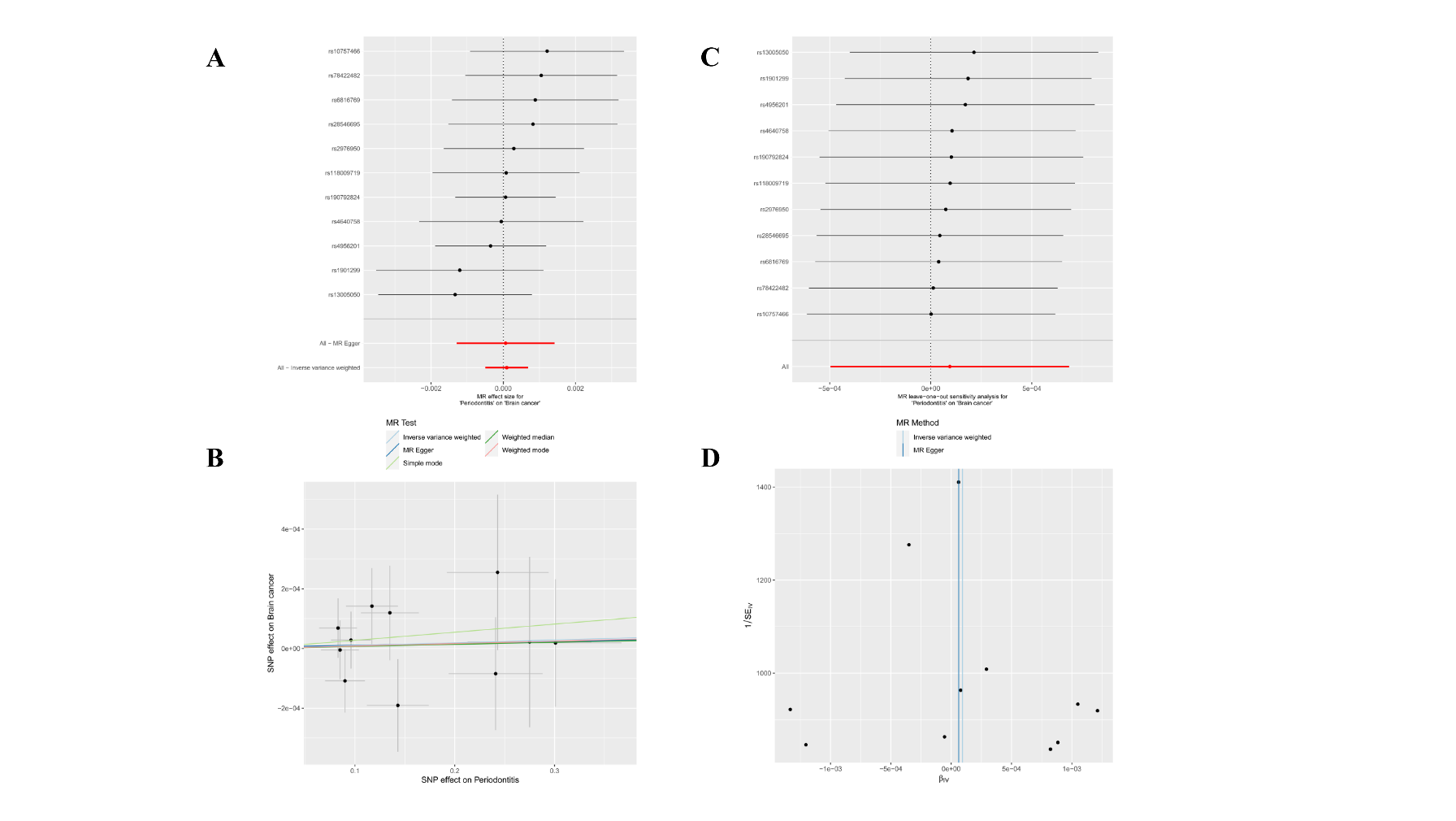
**

**Figure 3 Results and sensitivity analyses of the genetic correlation between periodontitis and brain cancer plotted in (A) forest plot; ( B ) scatterplot; (C) Leave-one-out sensitivity test; and (D) funnel plot.**

**
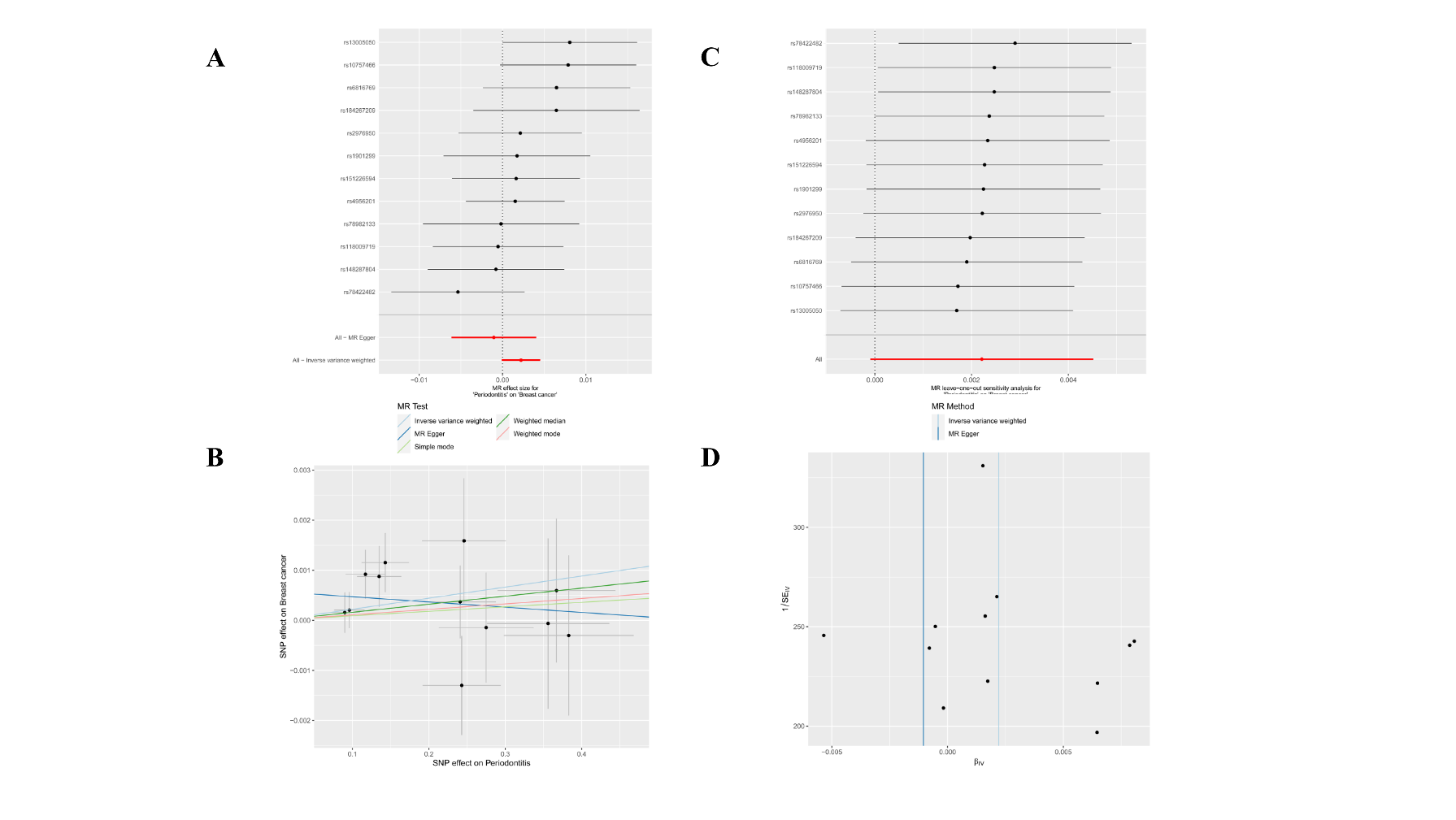
**

**Figure 4 Results and sensitivity analyses of the genetic correlation between periodontitis and breast cancer plotted in (A) forest plot; ( B ) scatterplot; (C) Leave-one-out sensitivity test; and (D) funnel plot.**

**
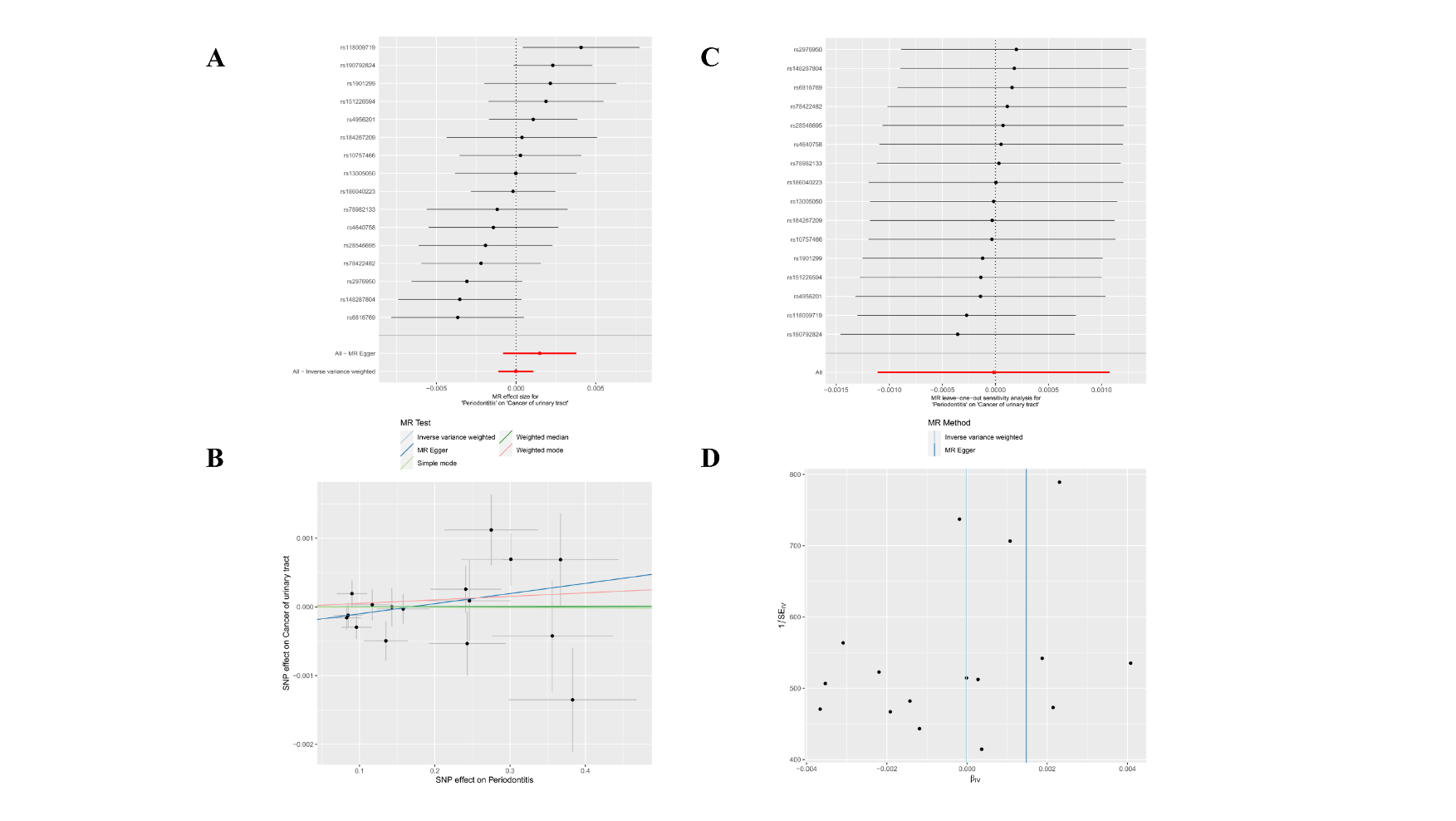
**

**Figure 5 Results and sensitivity analyses of the genetic correlation between periodontitis and cancer of urinary tract plotted in (A) forest plot; ( B ) scatterplot; (C) Leave-one-out sensitivity test; and (D) funnel plot.**

**
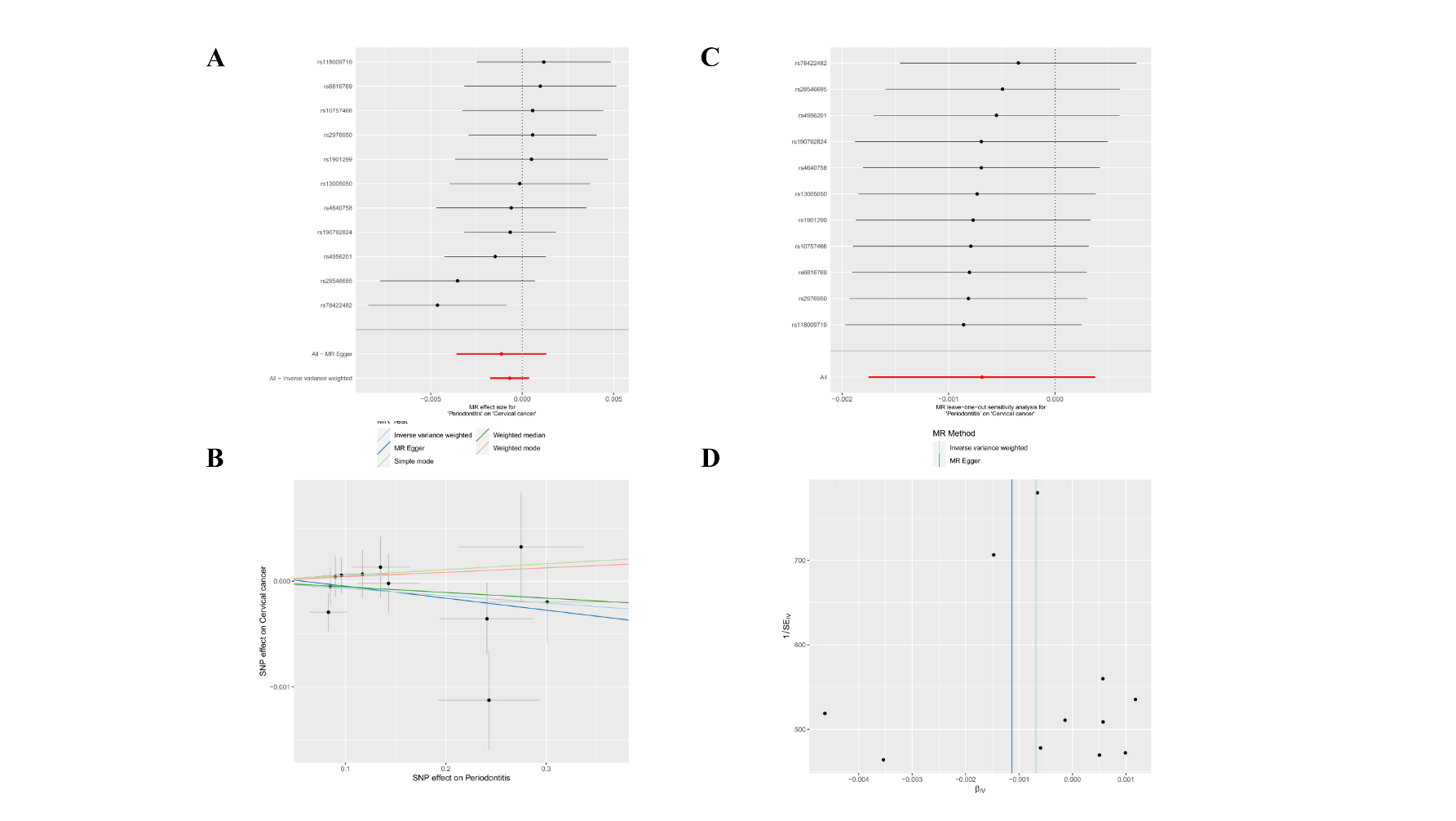
**

**Figure 6 Results and sensitivity analyses of the genetic correlation between periodontitis and cervical cancer plotted in (A) forest plot; ( B ) scatterplot; (C) Leave-one-out sensitivity test; and (D) funnel plot.**

**
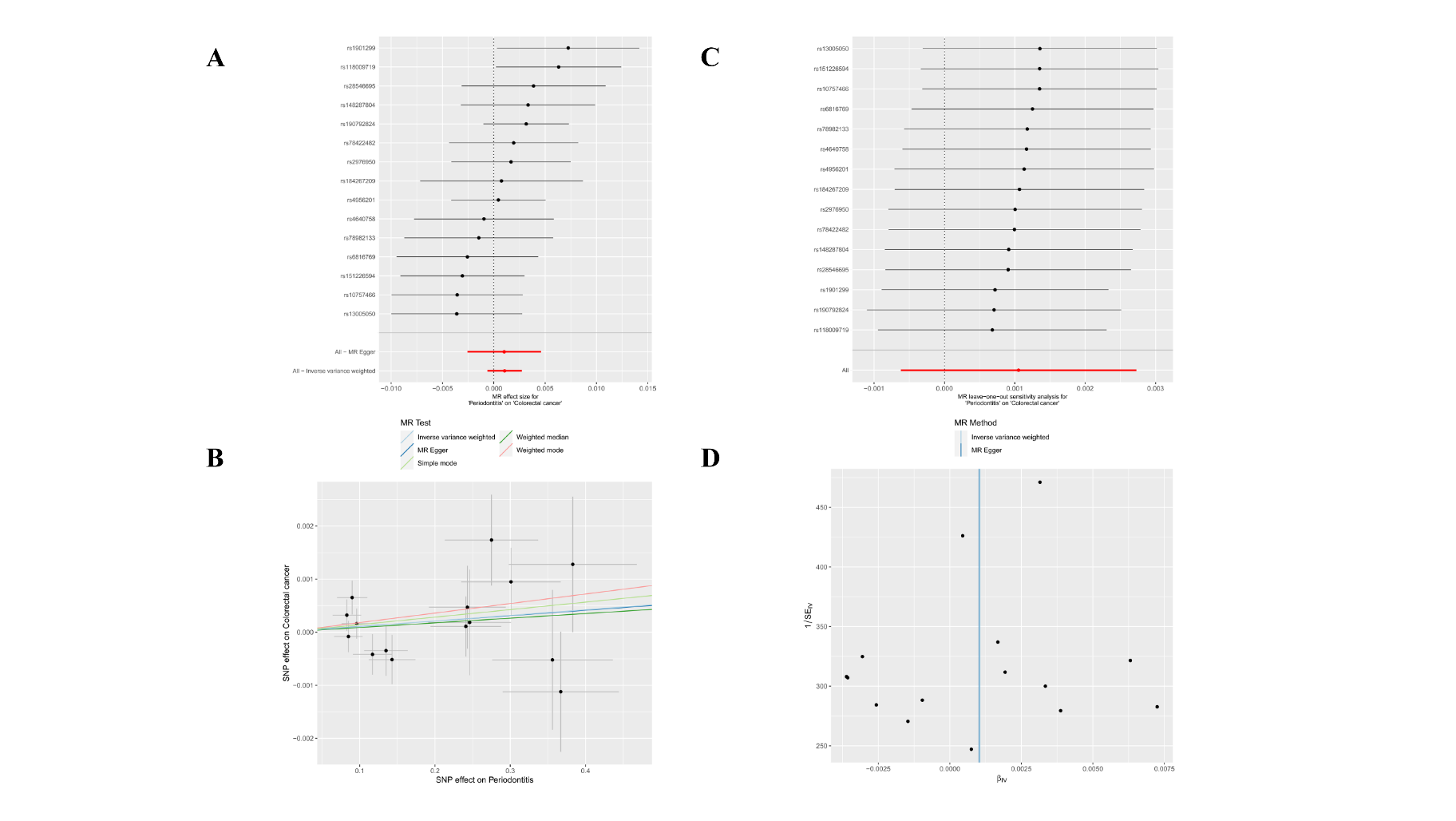
**

**Figure 7 Results and sensitivity analyses of the genetic correlation between periodontitis and colorectal cancer plotted in (A) forest plot; ( B ) scatterplot; (C) Leave-one-out sensitivity test; and (D) funnel plot.**

**
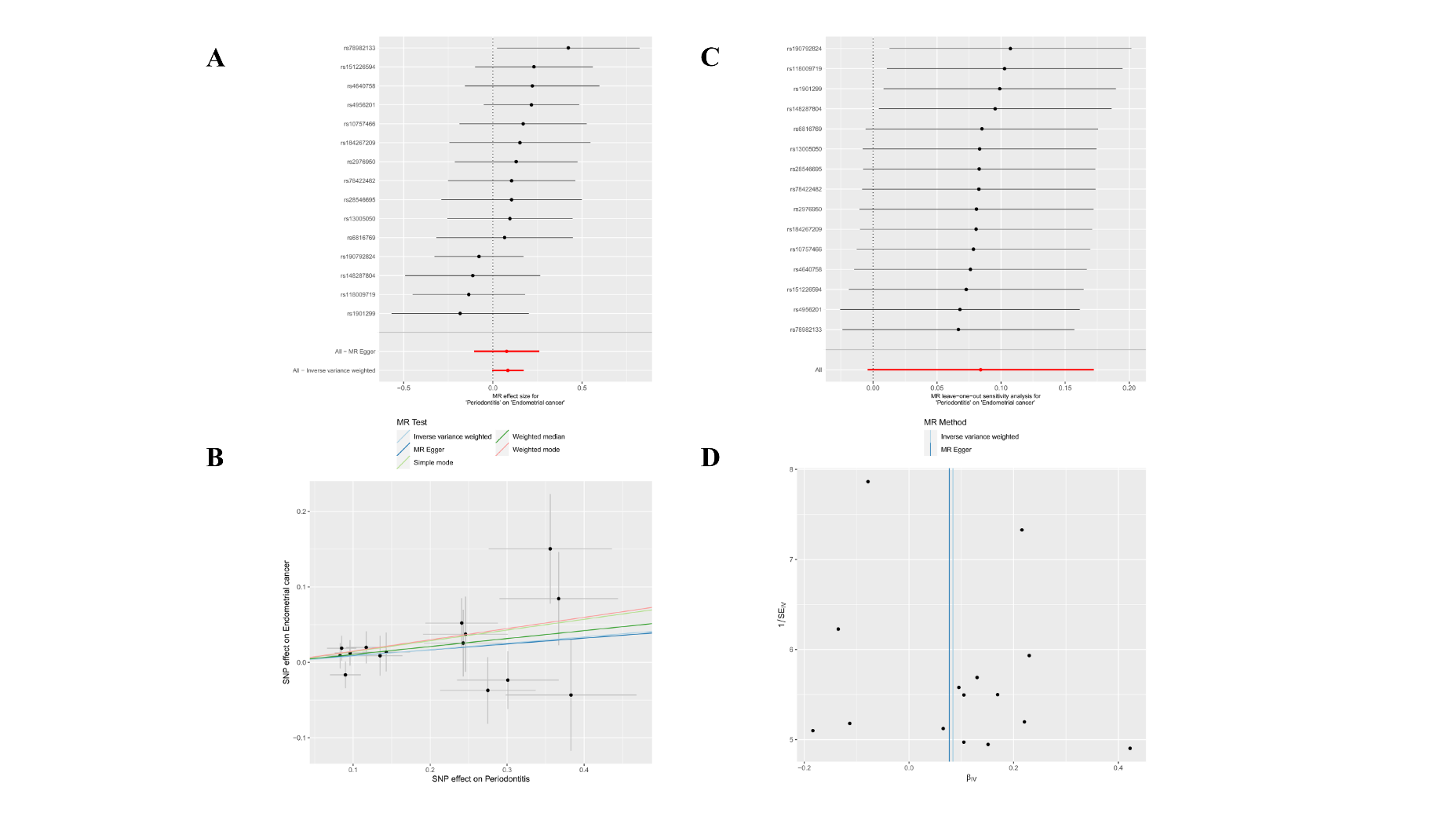
**

**Figure 8 Results and sensitivity analyses of the genetic correlation between periodontitis and endometrial cancer plotted in (A) forest plot; ( B ) scatterplot; (C) Leave-one-out sensitivity test; and (D) funnel plot.**

**
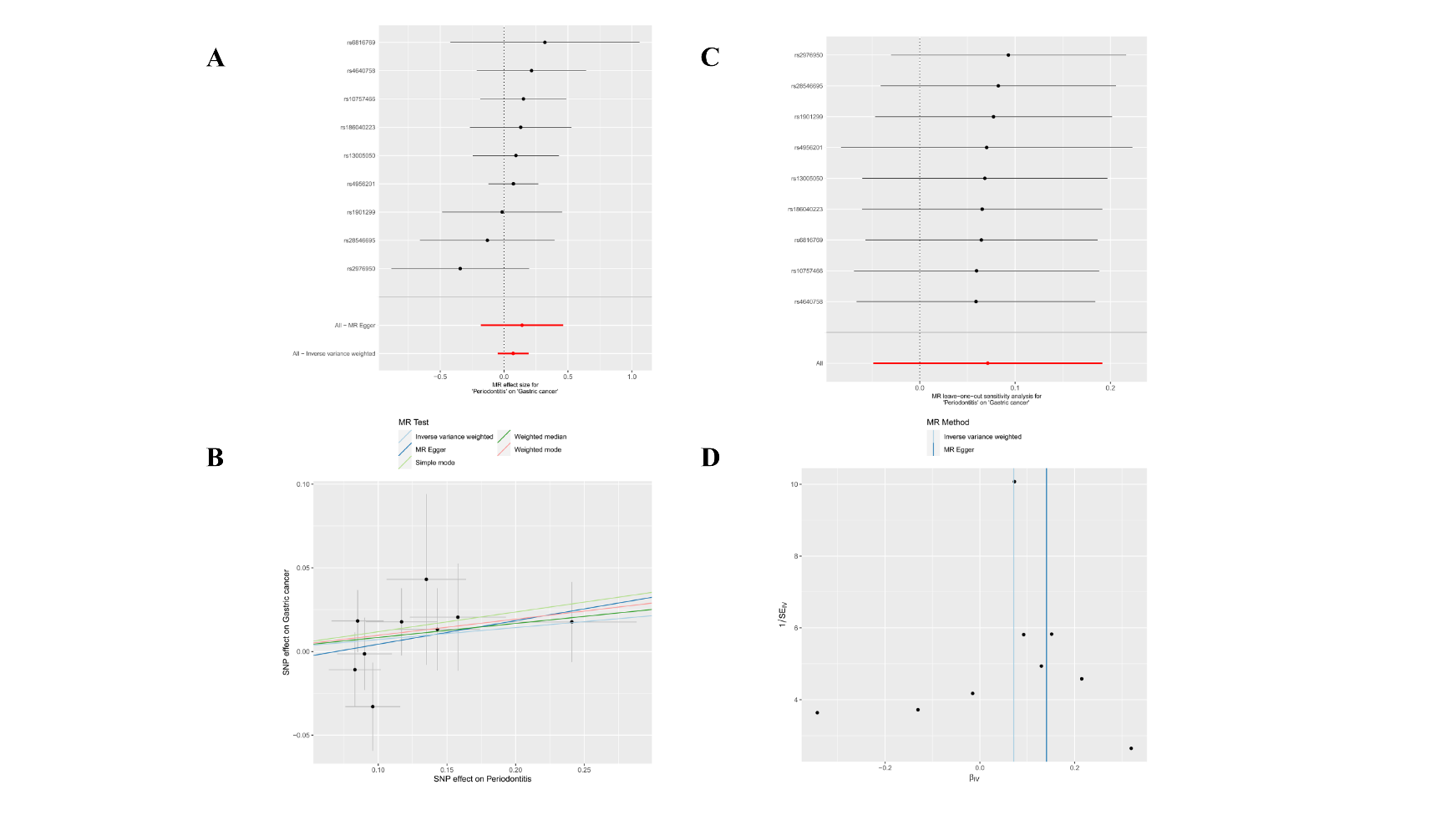
**

**Figure 9 Results and sensitivity analyses of the genetic correlation between periodontitis and gastric cancer plotted in (A) forest plot; ( B ) scatterplot; (C) Leave-one-out sensitivity test; and (D) funnel plot.**

**
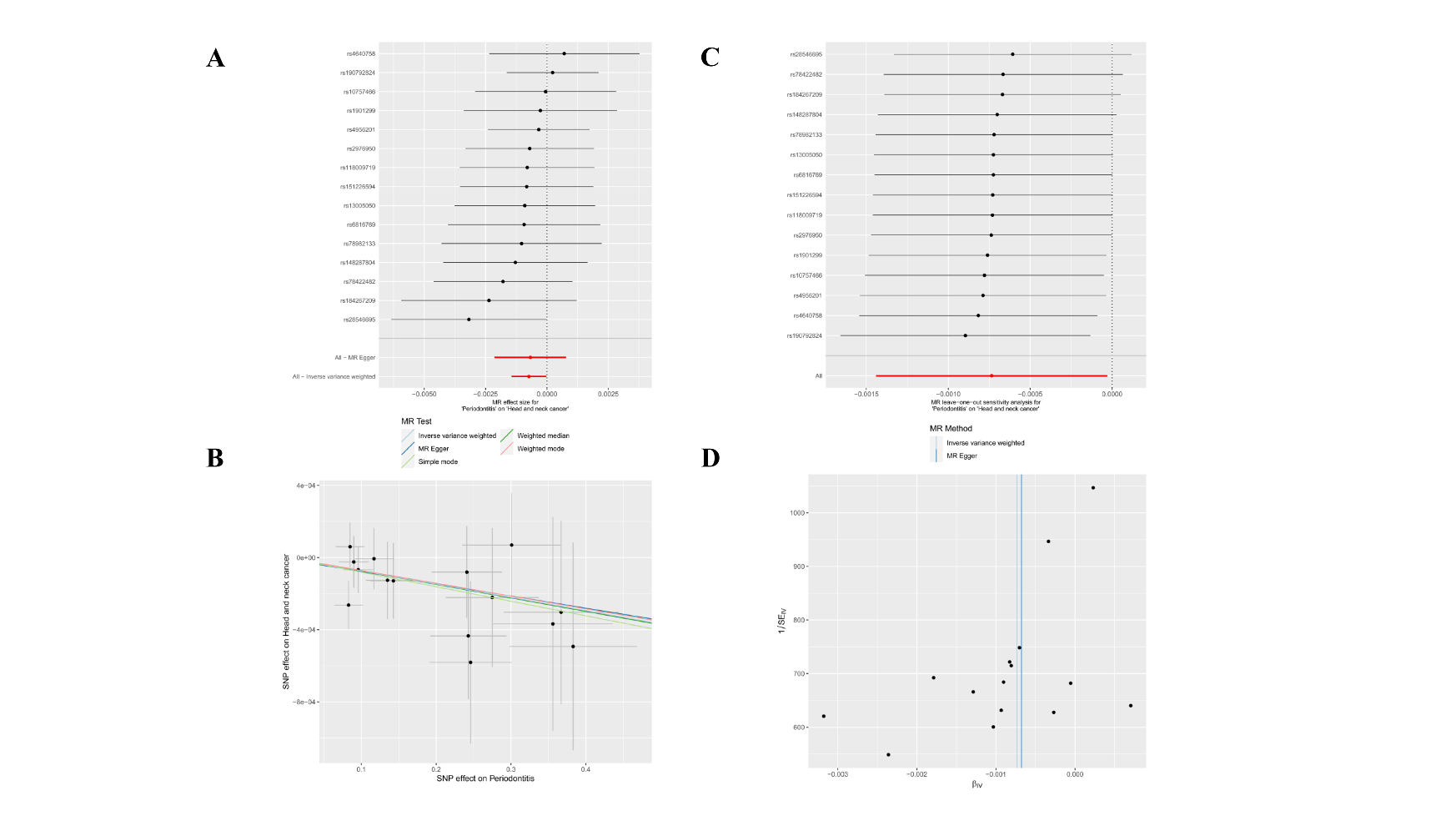
**

**Figure 10 Results and sensitivity analyses of the genetic correlation between periodontitis and head and neck cancer plotted in (A) forest plot; ( B ) scatterplot; (C) Leave-one-out sensitivity test; and (D) funnel plot.**

**
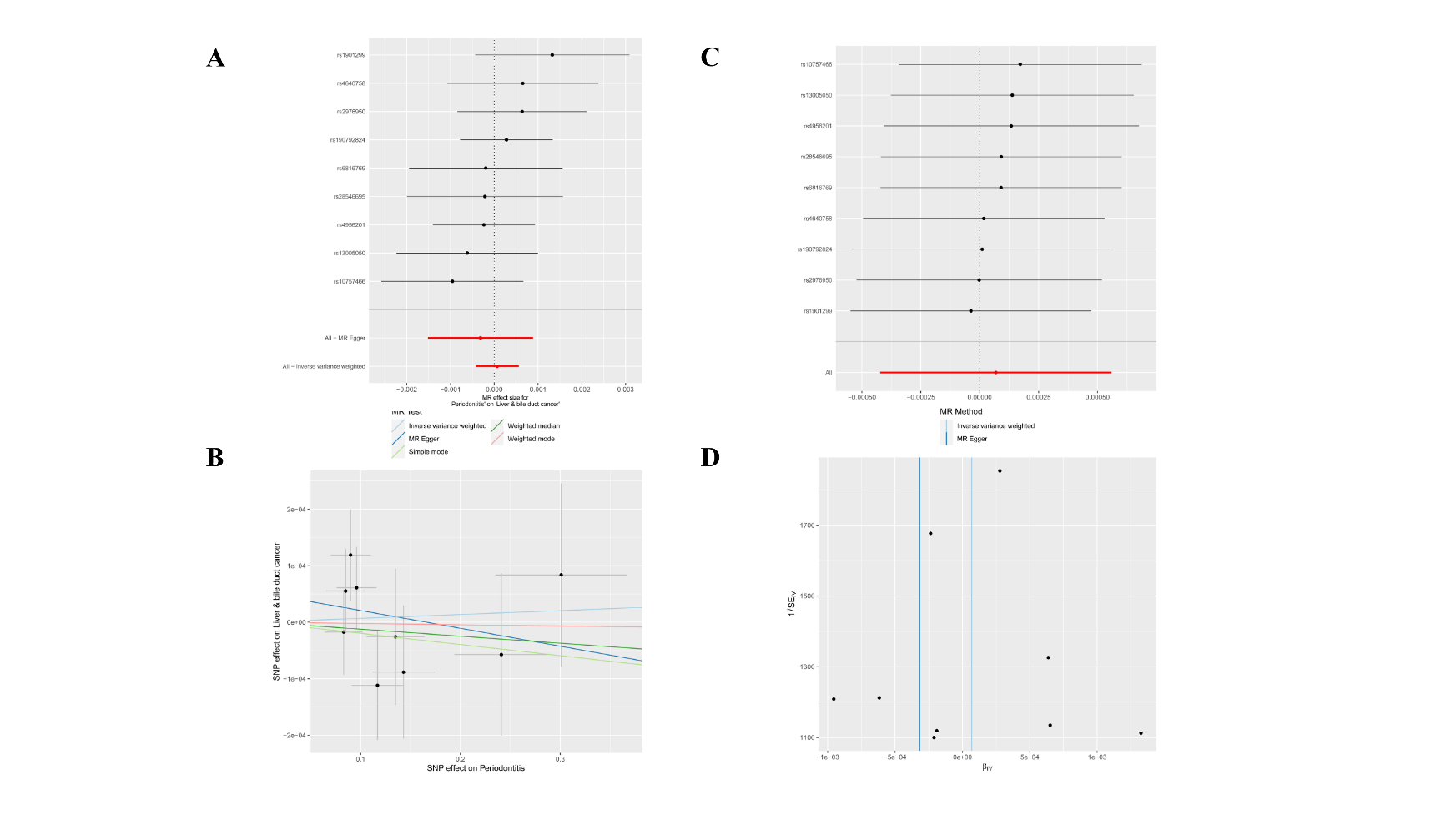
**

**Figure 11 Results and sensitivity analyses of the genetic correlation between periodontitis and liver & bile duct cancer plotted in (A) forest plot; ( B ) scatterplot; (C) Leave-one-out sensitivity test; and (D) funnel plot.**

**
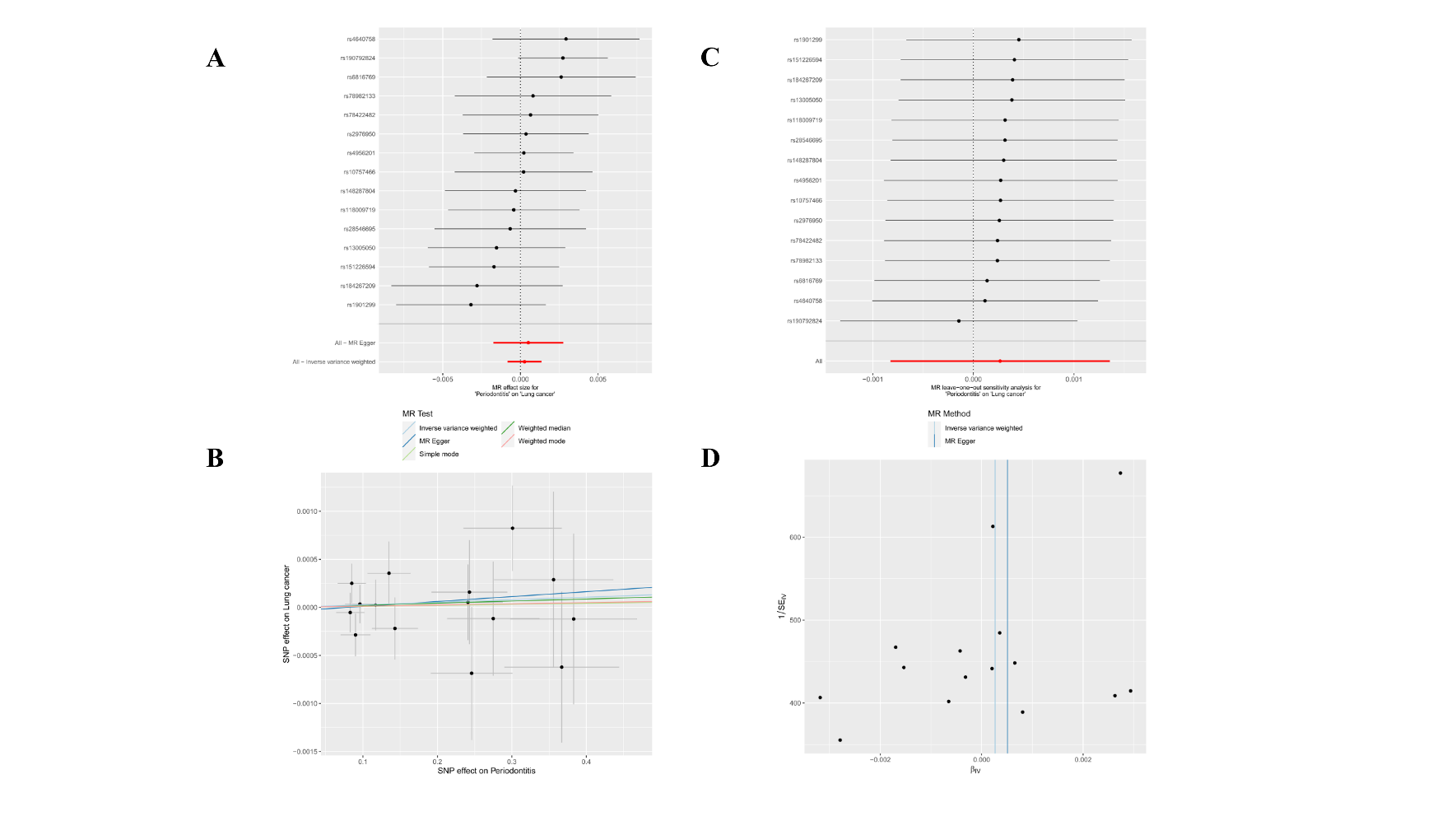
**

**Figure 12 Results and sensitivity analyses of the genetic correlation between periodontitis and lung cancer plotted in (A) forest plot; ( B ) scatterplot; (C) Leave-one-out sensitivity test; and (D) funnel plot.**

**
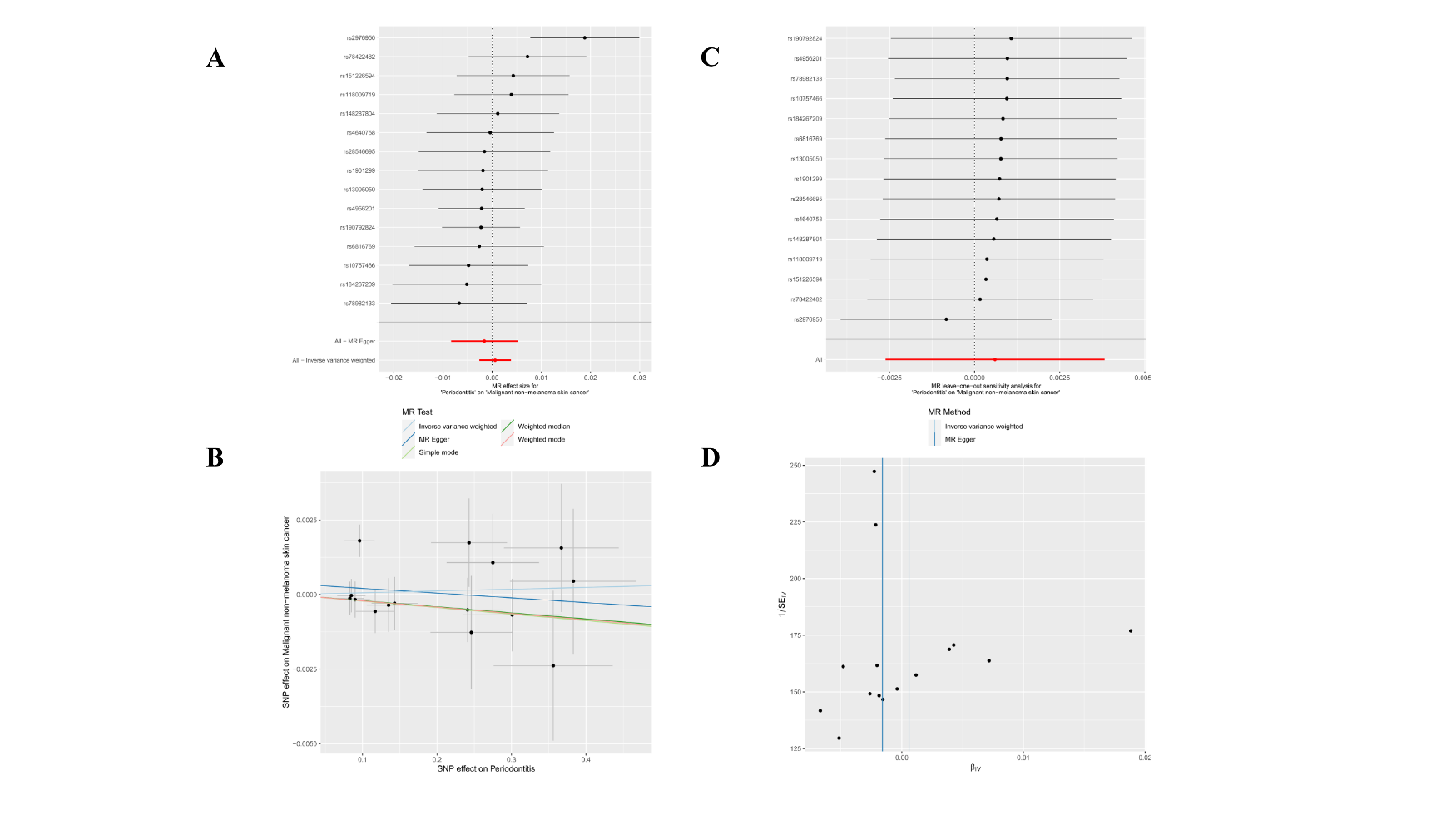
**

**Figure 13 Results and sensitivity analyses of the genetic correlation between periodontitis and malignant non-melanoma skin cancer plotted in (A) forest plot; ( B ) scatterplot; (C) Leave-one-out sensitivity test; and (D) funnel plot.**

**
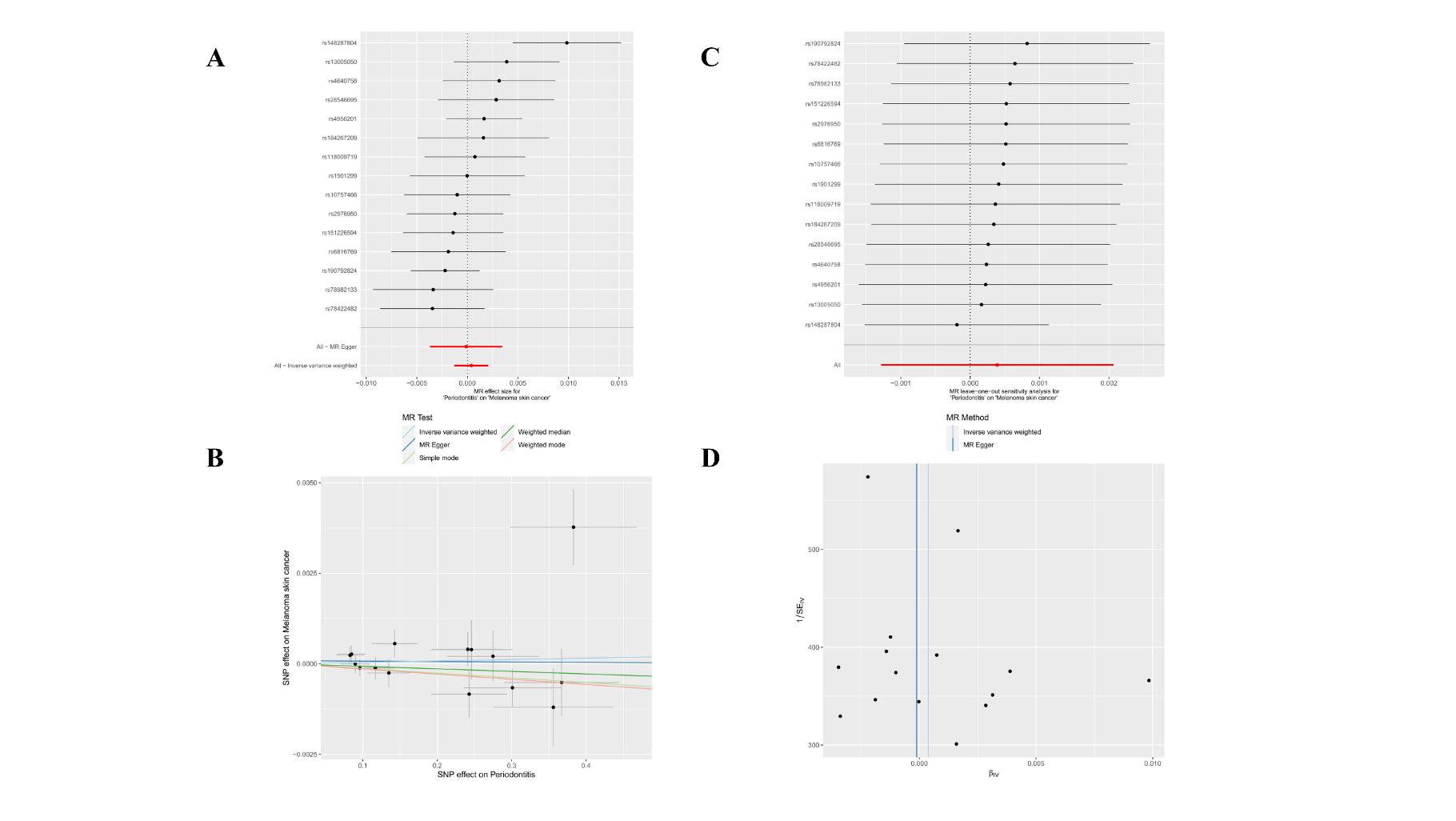
**

**Figure 14 Results and sensitivity analyses of the genetic correlation between periodontitis and melanoma skin cancer plotted in (A) forest plot; ( B ) scatterplot; (C) Leave-one-out sensitivity test; and (D) funnel plot.**

**
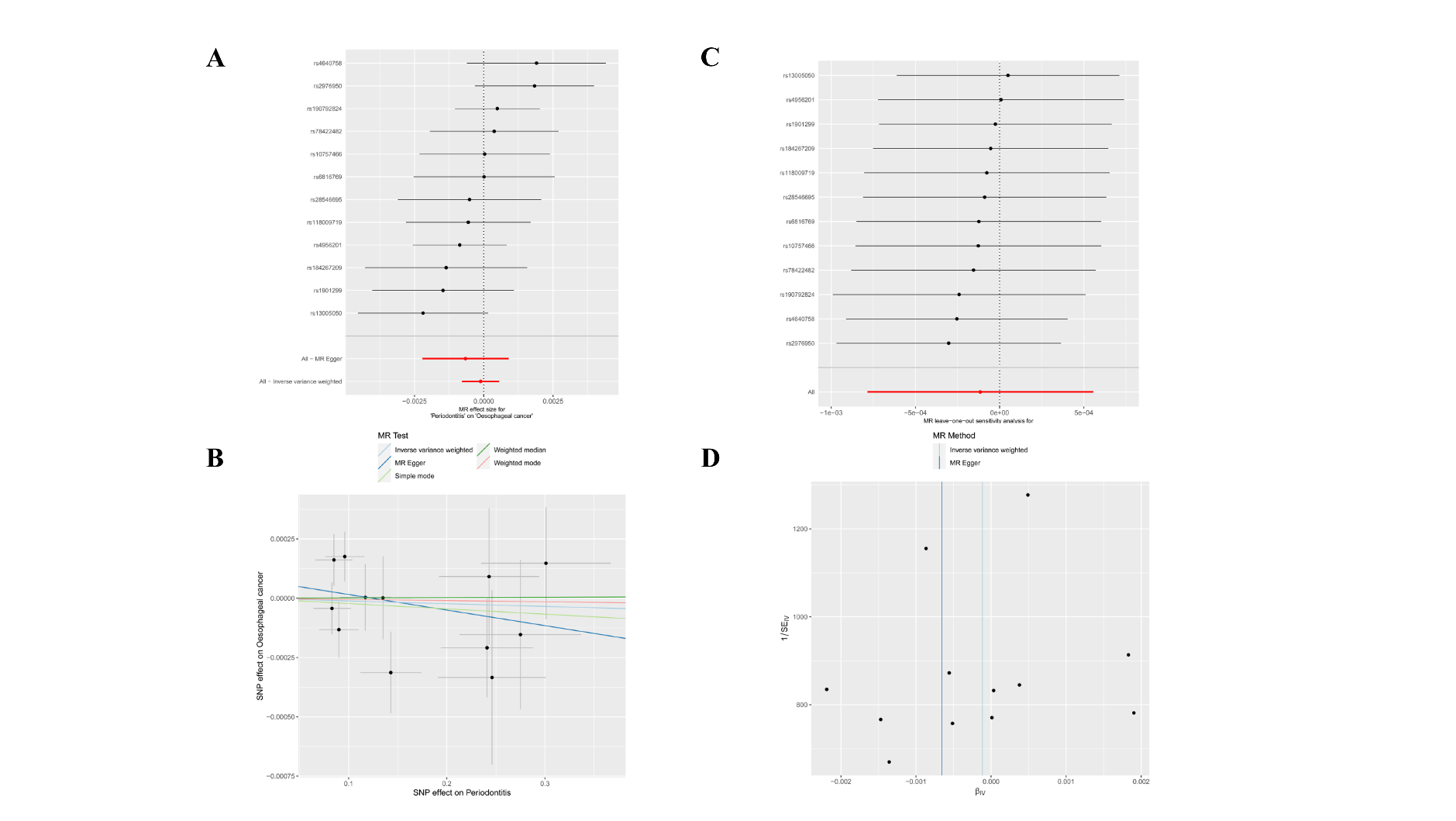
**

**Figure 15 Results and sensitivity analyses of the genetic correlation between periodontitis and oesophageal cancer plotted in (A) forest plot; ( B ) scatterplot; (C) Leave-one-out sensitivity test; and (D) funnel plot.**

**
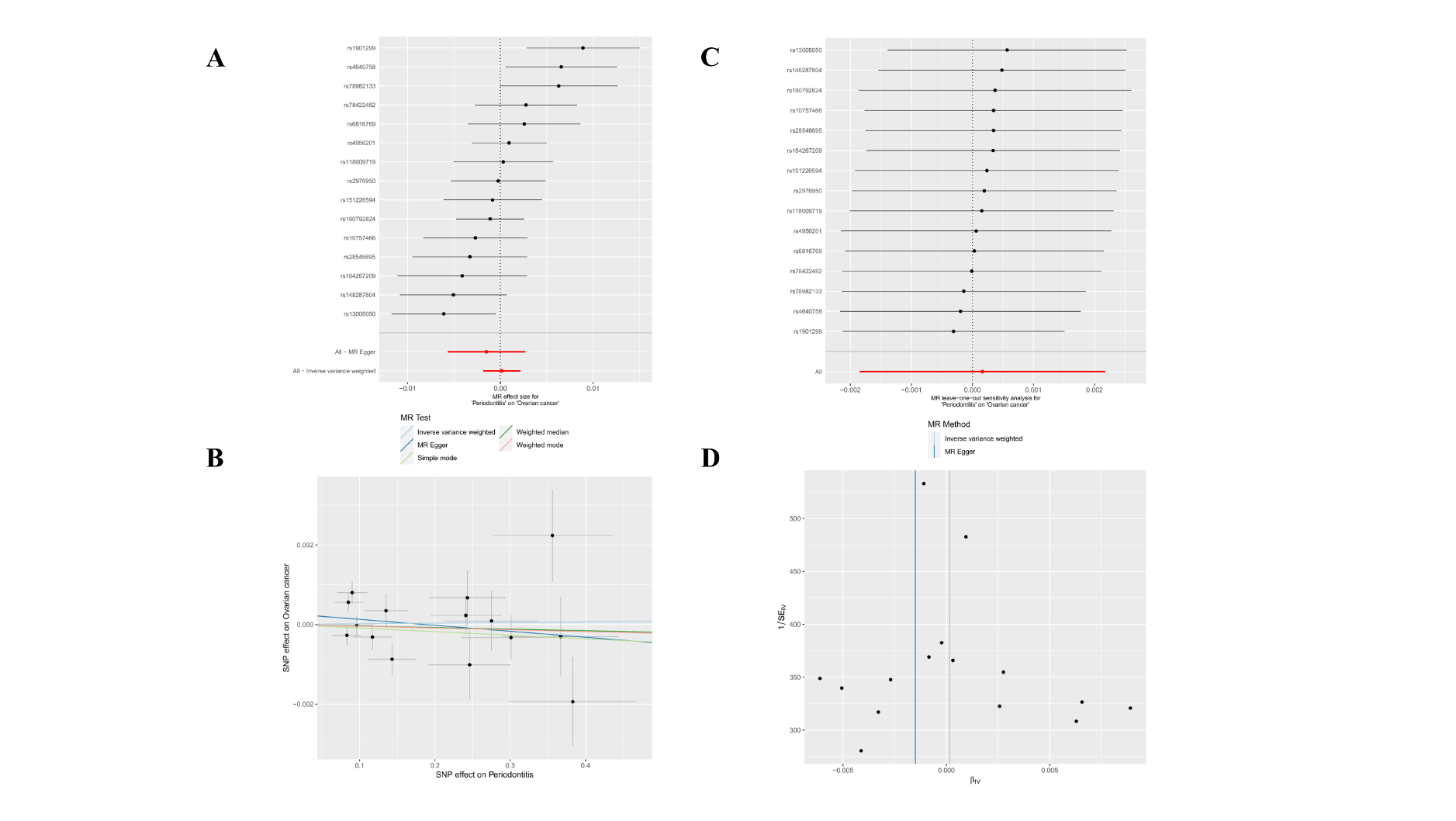
**

**Figure 16 Results and sensitivity analyses of the genetic correlation between periodontitis and ovarian cancer plotted in (A) forest plot; ( B ) scatterplot; (C) Leave-one-out sensitivity test; and (D) funnel plot.**

**
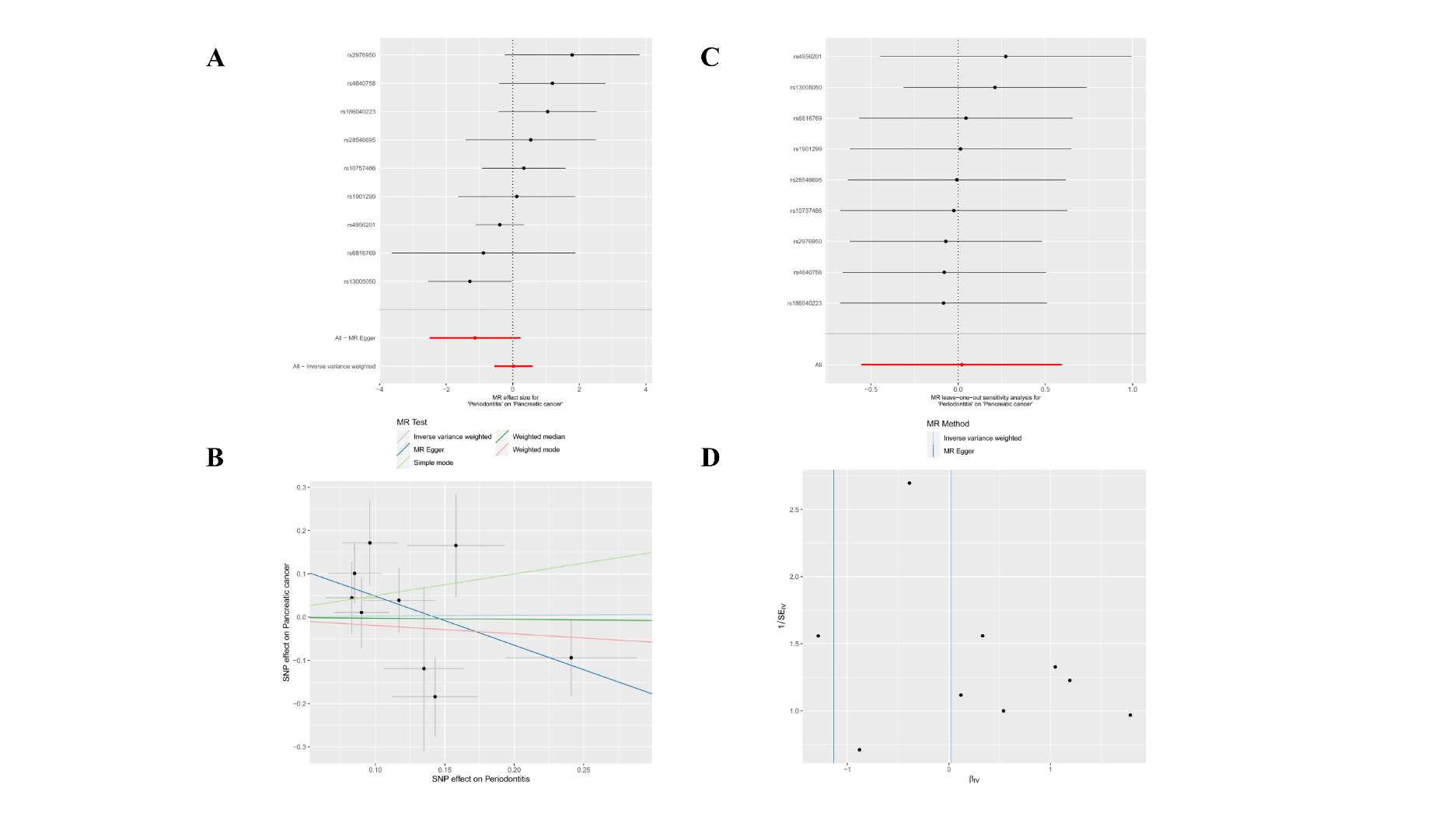
**

**Figure 17 Results and sensitivity analyses of the genetic correlation between periodontitis and pancreatic cancer plotted in (A) forest plot; ( B ) scatterplot; (C) Leave-one-out sensitivity test; and (D) funnel plot.**

**
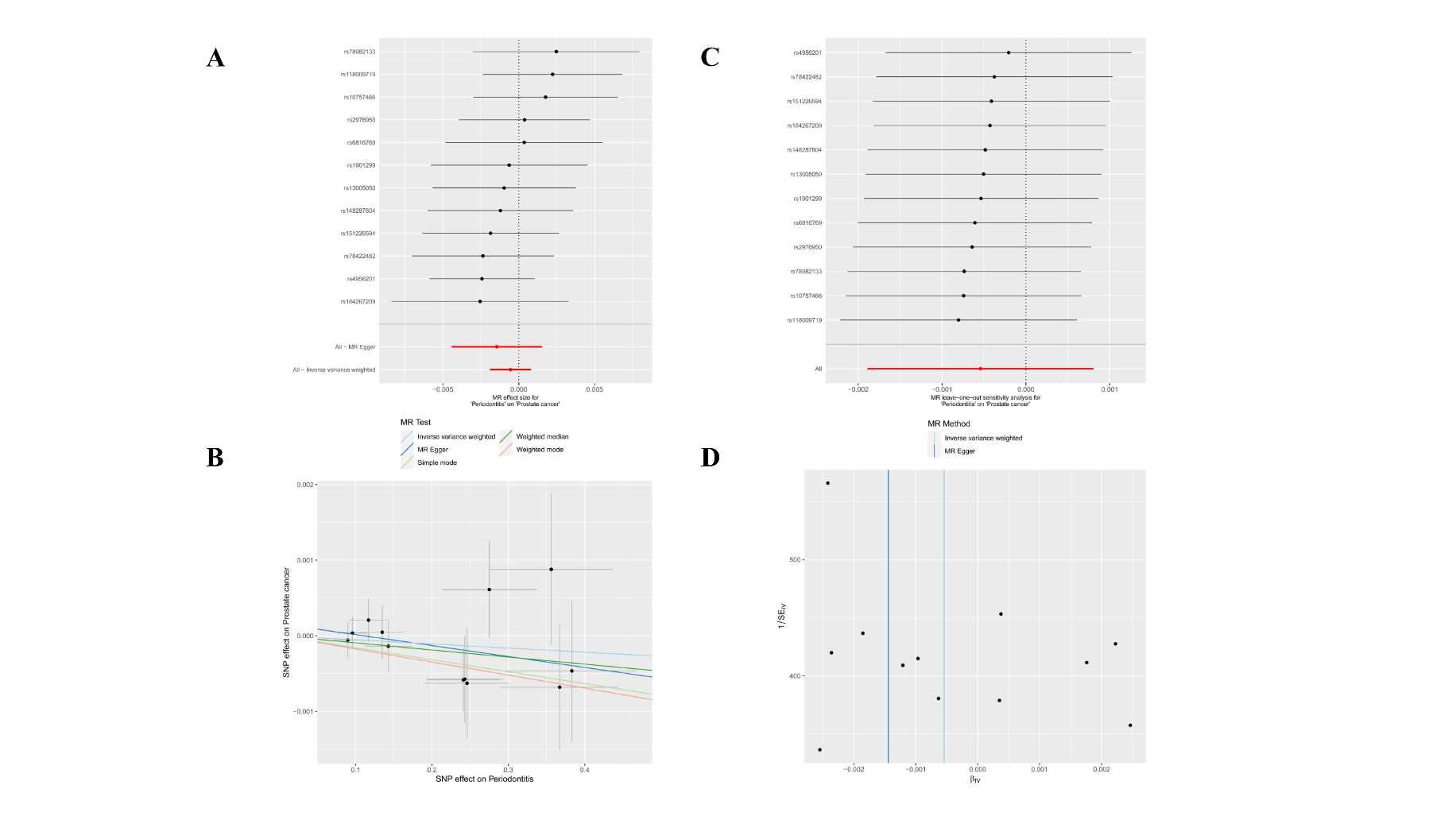
**

**Figure 18 Results and sensitivity analyses of the genetic correlation between periodontitis and prostate cancer plotted in (A) forest plot; ( B ) scatterplot; (C) Leave-one-out sensitivity test; and (D) funnel plot.**

**
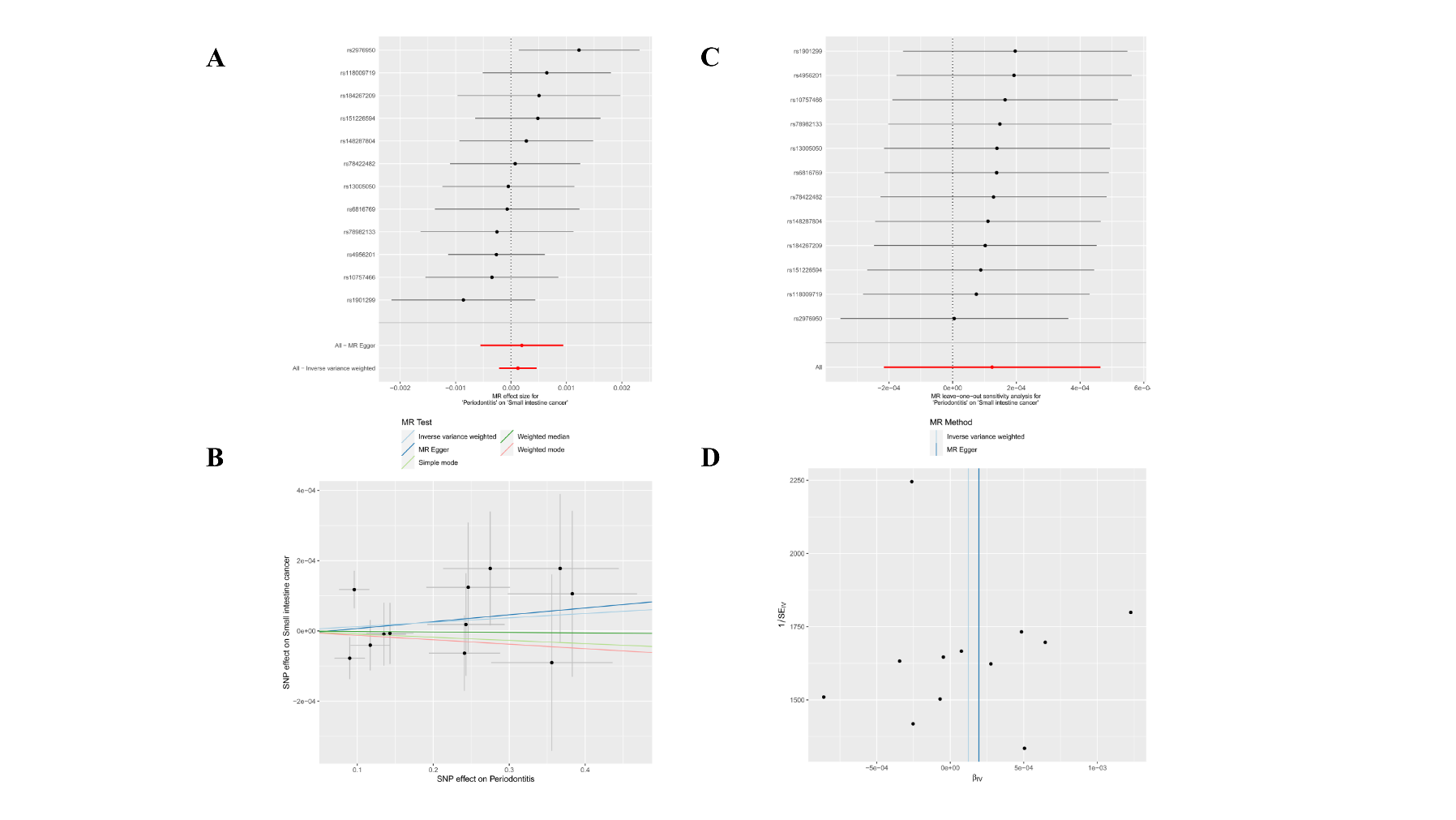
**

**Figure 19 Results and sensitivity analyses of the genetic correlation between periodontitis and small intestine cancer plotted in (A) forest plot; ( B ) scatterplot; (C) Leave-one-out sensitivity test; and (D) funnel plot.**

**
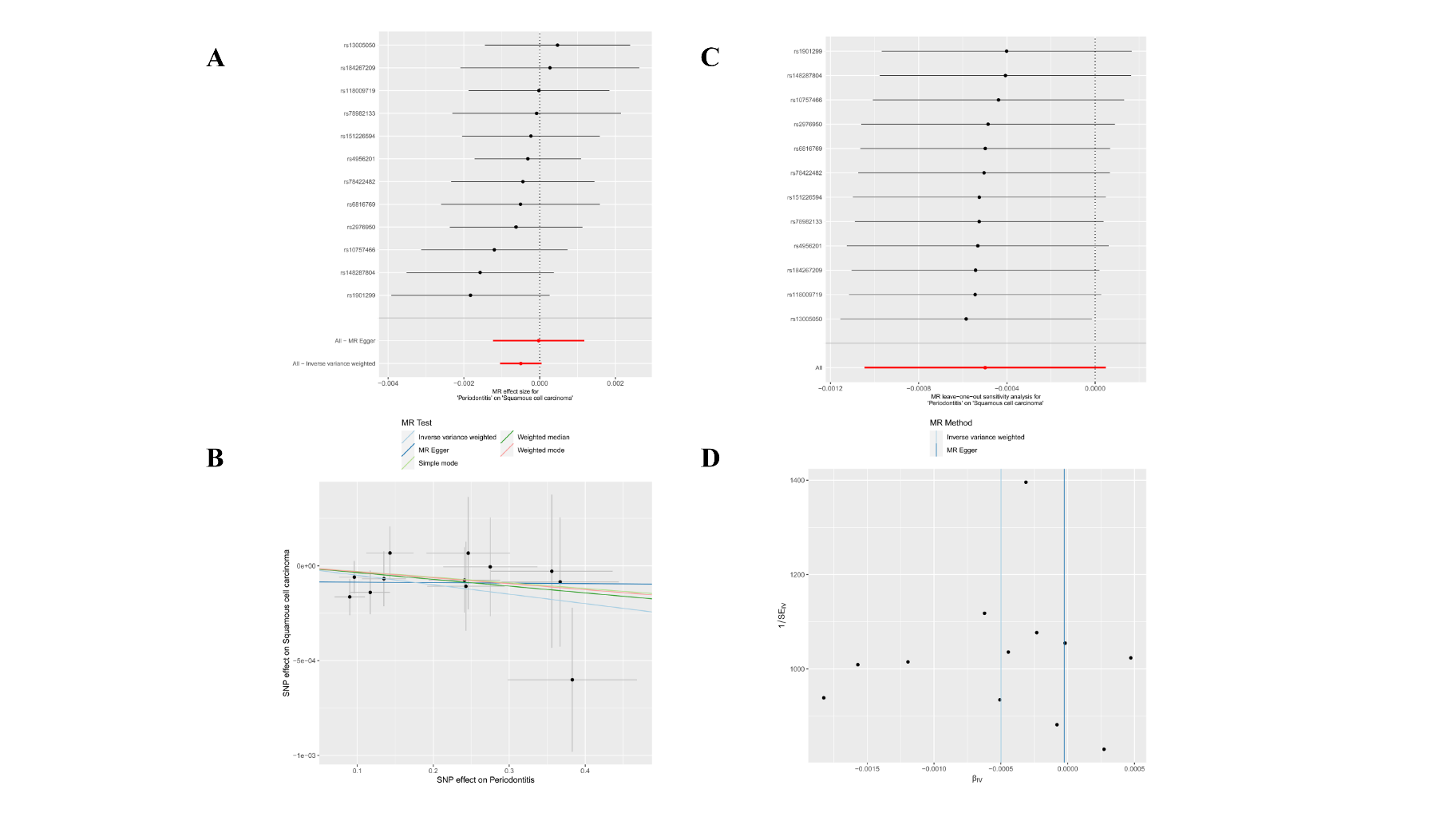
**

**Figure 20 Results and sensitivity analyses of the genetic correlation between periodontitis and squamous cell carcinoma plotted in (A) forest plot; ( B ) scatterplot; (C) Leave-one-out sensitivity test; and (D) funnel plot.**

S4. Mendelian randomization analysis of periodontitis and 4 subclasses of head and neck cancer

**Table 1** Results of Mendelian randomization analysis

**Table2** Sensitivity analysis of the Mendelian randomization analysis results

**Figure 1-4** Results and sensitivity analyses plots of the genetic correlation between periodontitis and 4 subclasses of head and neck cancer

**Table 1 Results of Mendelian randomization analysis**

| **Outcome Exposure** | **Method** | **Periodontitis** | | |
| --- | --- | --- | --- | --- |
|  |  | **SNP (n)** | **OR (95%CI)** | ***P* Value** |
|  | **MR Egger** | 9 | 1.000 | 0.396 |
|  | **Weighted median** | 9 | 1.000 | 0.280 |
| **Laryngeal cancer** | **IVW** | 9 | 1.000 | 0.310 |
|  | **Simple mode** | 9 | 1.000 | 0.954 |
|  | **Weighted mode** | 9 | 1.000 | 0.408 |
|  | **MR Egger** | 14 | 0.914 | 0.762 |
|  | **Weighted median** | 14 | 0.963 | 0.834 |
| **Malignant neoplasm of thyroid gland** | **IVW** | 14 | 0.999 | 0.992 |
|  | **Simple mode** | 14 | 1.091 | 0.787 |
|  | **Weighted mode** | 14 | 1.104 | 0.735 |
|  | **MR Egger** | 9 | 1.000 | 0.925 |
|  | **Weighted median** | 9 | 1.000 | 0.892 |
| **Oral cavity cancer** | **IVW** | 9 | 1.000 | 0.679 |
|  | **Simple mode** | 9 | 1.000 | 0.799 |
|  | **Weighted mode** | 9 | 1.000 | 0.937 |
|  | **MR Egger** | 11 | 0.999 | 0.424 |
|  | **Weighted median** | 11 | 0.999 | 0.116 |
| **Oropharyngeal cancer** | **IVW** | 11 | 0.999 | 0.022 |
|  | **Simple mode** | 11 | 0.999 | 0.334 |
|  | **Weighted mode** | 11 | 1.000 | 0.333 |

**Table2 Sensitivity analysis of the Mendelian randomization analysis results**

| **Outcome Exposure** | **Method** | | **Periodontitis** | |
| --- | --- | --- | --- | --- |
|  | **IVW (heterogeneity)** | ***p* value** | 0.592 |  |
|  |  | **Q** | 6.491 |  |
| **Laryngeal cancer** | **MR Egger (heterogeneity)** | ***p* value** | 0.516 |  |
|  |  | **Q** | 6.205 |  |
|  | **MR Egger (pleiotropy)** | ***p* value** | 0.610 |  |
|  |  | **intercept** | 0.000 |  |
|  | **IVW (heterogeneity)** | ***p* value** | 0.369 |  |
|  |  | **Q** | 14.075 |  |
| **Malignant neoplasm of thyroid gland** | **MR Egger (heterogeneity)** | ***p* value** | 0.305 |  |
|  |  | **Q** | 13.938 |  |
|  | **MR Egger (pleiotropy)** | ***p* value** | 0.737 |  |
|  |  | **intercept** | 0.016 |  |
|  | **IVW (heterogeneity)** | ***p* value** | 0.696 |  |
|  |  | **Q** | 5.561 |  |
| **Oral cavity cancer** | **MR Egger (heterogeneity)** | ***p* value** | 0.602 |  |
|  |  | **Q** | 5.475 |  |
|  | **MR Egger (pleiotropy)** | ***p* value** | 0.778 |  |
|  |  | **intercept** | 0.000 |  |
|  | **IVW (heterogeneity)** | ***p* value** | 0.983 |  |
|  |  | **Q** | 2.923 |  |
| **Oropharyngeal cancer** | **MR Egger (heterogeneity)** | ***p* value** | 0.968 |  |
|  |  | **Q** | 2.891 |  |
|  | **MR Egger (pleiotropy)** | ***p* value** | 0.862 |  |
|  |  | **intercept** | 0.000 |  |

**
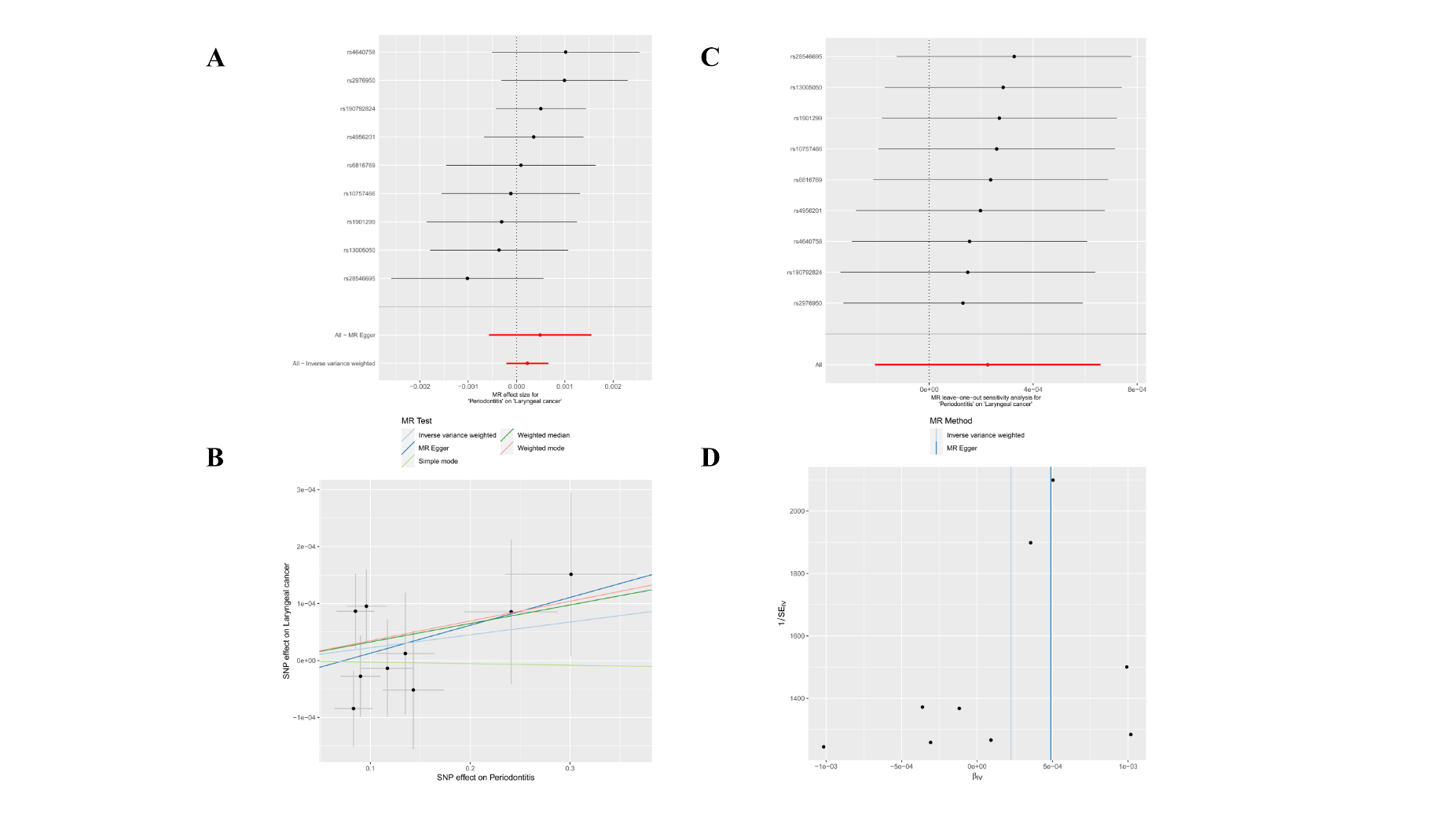
**

**Figure 1 Results and sensitivity analyses of the genetic correlation between periodontitis and laryngeal cancer plotted in (A) forest plot; ( B ) scatterplot; (C) Leave-one-out sensitivity test; and (D) funnel plot.**

**
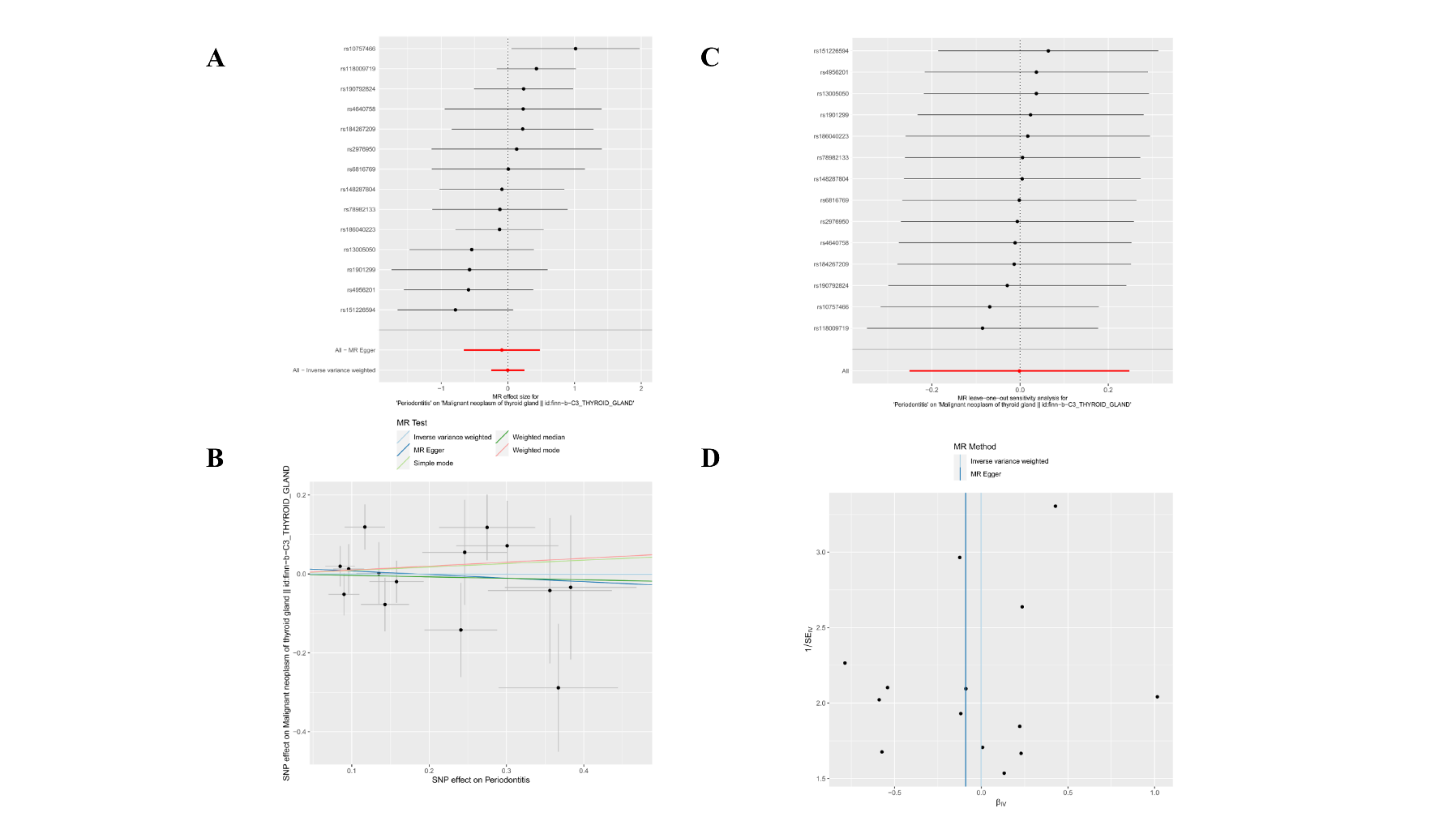
**

**Figure 2 Results and sensitivity analyses of the genetic correlation between periodontitis and malignant neoplasm of thyroid gland plotted in (A) forest plot; ( B ) scatterplot; (C) Leave-one-out sensitivity test; and (D) funnel plot.**

**
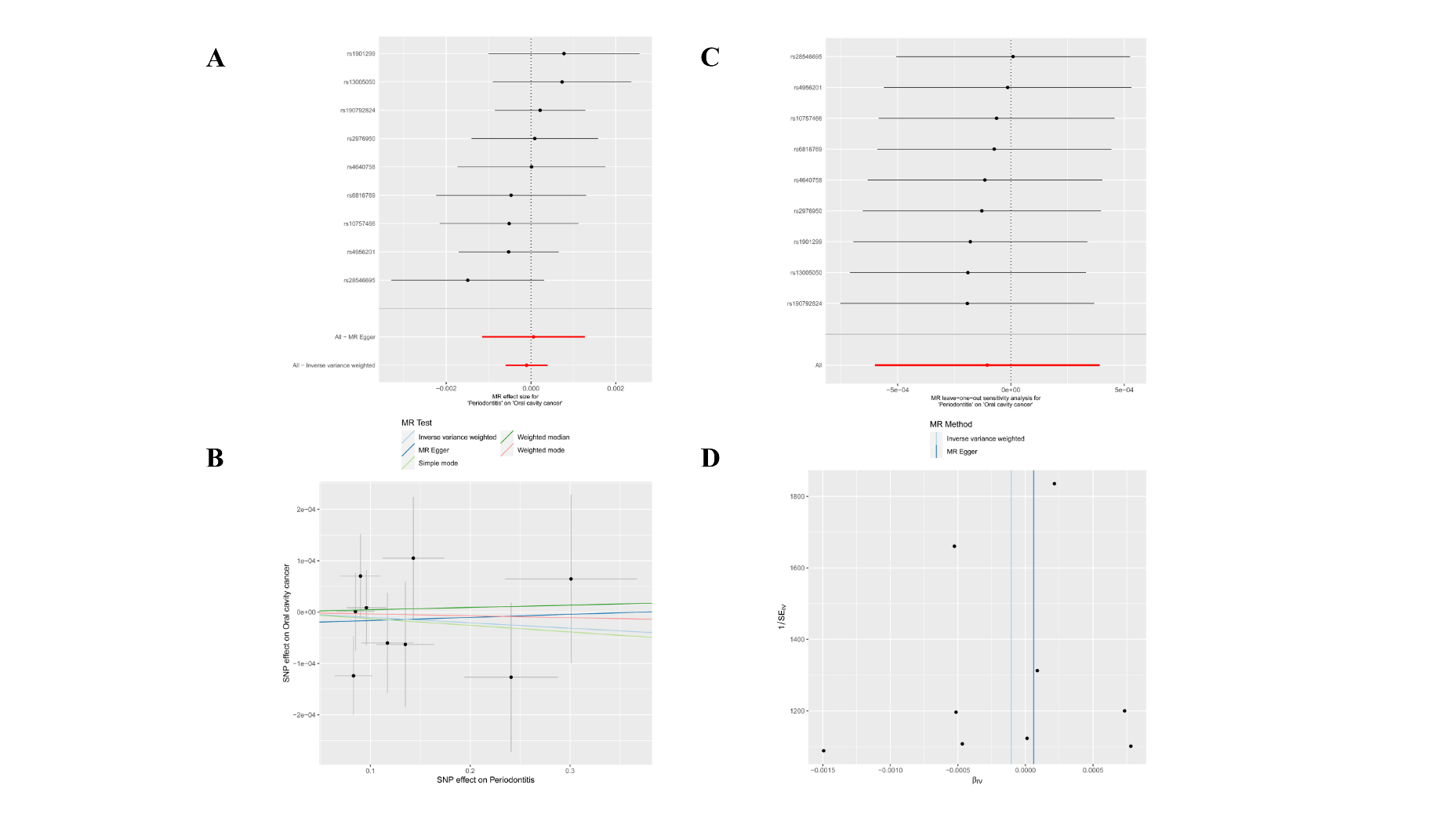
**

**Figure 3 Results and sensitivity analyses of the genetic correlation between periodontitis and oral cavity cancer plotted in (A) forest plot; ( B ) scatterplot; (C) Leave-one-out sensitivity test; and (D) funnel plot.**

**
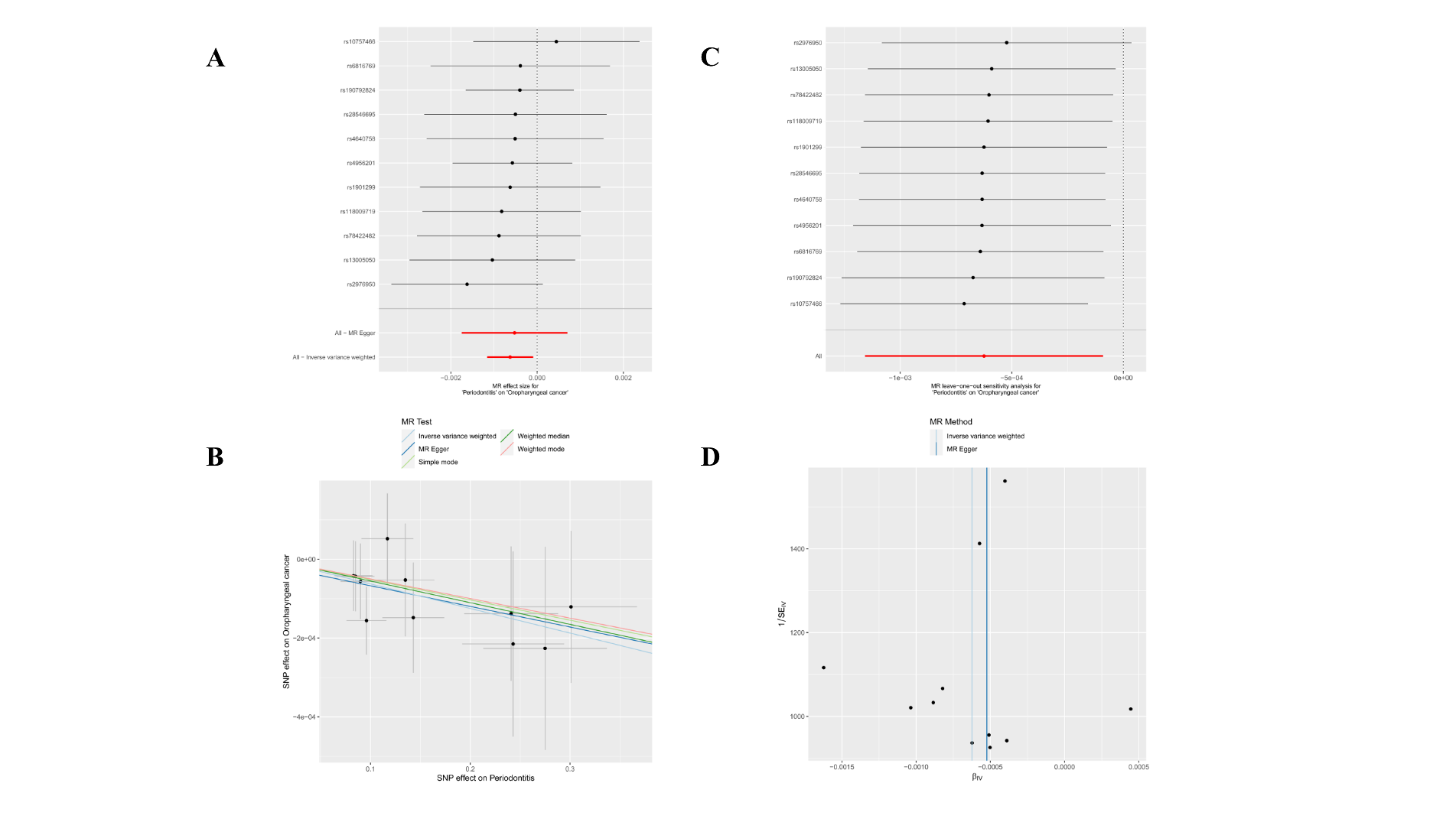
**

**Figure 4 Results and sensitivity analyses of the genetic correlation between periodontitis and oropharyngeal cancer plotted in (A) forest plot; ( B ) scatterplot; (C) Leave-one-out sensitivity test; and (D) funnel plot.**

S5. Mendelian randomization analysis of 20 oral microorganisms and oropharyngeal cancer.

**Table 1** Results of Mendelian randomization analysis

**Table2** Sensitivity analysis of the Mendelian randomization analysis results

**Figure 1-20** Results and sensitivity analyses plots of the genetic correlation between 20 oral microorganisms and oropharyngeal cancer

**Table 1 Results of Mendelian randomization analysis**

| **Exposure Outcome** | **Method** | **Oropharyngeal cancer** | | |
| --- | --- | --- | --- | --- |
|  |  | **SNP (n)** | **OR** | ***P* Value** |
|  | **MR Egger** | 12 | 1.004 | 0.415 |
|  | **Weighted median** | 12 | 1.000 | 0.882 |
| **Actinomyces oris** | **IVW** | 12 | 1.000 | 0.899 |
|  | **Simple mode** | 12 | 1.000 | 0.494 |
|  | **Weighted mode** | 12 | 1.000 | 0.626 |
|  | **MR Egger** | 7 | 0.991 | 0.417 |
|  | **Weighted median** | 7 | 1.000 | 0.803 |
| **Capnocytophaga granulosa** | **IVW** | 7 | 1.000 | 0.240 |
|  | **Simple mode** | 7 | 0.998 | 0.127 |
|  | **Weighted mode** | 7 | 1.000 | 0.729 |
| **Capnocytophaga leadbetteri** | **IVW** | 2 | 1.000 | 0.904 |
|  | **MR Egger** | 10 | 1.002 | 0.868 |
|  | **Weighted median** | 10 | 1.000 | 0.691 |
| **Capnocytophaga sputigena** | **IVW** | 10 | 1.000 | 0.452 |
|  | **Simple mode** | 10 | 1.000 | 0.891 |
|  | **Weighted mode** | 10 | 1.000 | 0.801 |
|  | **MR Egger** | 13 | 1.006 | 0.259 |
|  | **Weighted median** | 13 | 1.001 | 0.107 |
| **Corynebacterium durum** | **IVW** | 13 | 1.000 | 0.685 |
|  | **Simple mode** | 13 | 1.001 | 0.217 |
|  | **Weighted mode** | 13 | 1.001 | 0.207 |
|  | **MR Egger** | 12 | 0.999 | 0.726 |
|  | **Weighted median** | 12 | 1.000 | 0.525 |
| **Gemella haemolysans** | **IVW** | 12 | 1.000 | 0.746 |
|  | **Simple mode** | 12 | 1.000 | 0.501 |
|  | **Weighted mode** | 12 | 1.000 | 0.462 |
|  | **MR Egger** | 8 | 0.996 | 0.707 |
|  | **Weighted median** | 8 | 1.000 | 0.776 |
| **Haemophilus parainfluenzae** | **IVW** | 8 | 1.000 | 0.831 |
|  | **Simple mode** | 8 | 1.000 | 0.566 |
|  | **Weighted mode** | 8 | 1.000 | 0.760 |
|  | **MR Egger** | 9 | 1.009 | 0.187 |
|  | **Weighted median** | 9 | 1.000 | 0.856 |
| **Kingella** | **IVW** | 9 | 1.000 | 0.312 |
|  | **Simple mode** | 9 | 1.000 | 0.906 |
|  | **Weighted mode** | 9 | 1.000 | 0.947 |
|  | **MR Egger** | 8 | 1.001 | 0.884 |
|  | **Weighted median** | 8 | 1.000 | 0.536 |
| **Neisseria flava** | **IVW** | 8 | 1.000 | 0.241 |
|  | **Simple mode** | 8 | 1.000 | 0.873 |
|  | **Weighted mode** | 8 | 1.000 | 0.864 |
|  | **MR Egger** | 7 | 1.009 | 0.180 |
|  | **Weighted median** | 7 | 1.000 | 0.990 |
| **Rothia aeria** | **IVW** | 7 | 1.000 | 0.983 |
|  | **Simple mode** | 7 | 1.000 | 0.721 |
|  | **Weighted mode** | 7 | 1.000 | 0.770 |
|  | **MR Egger** | 7 | 1.005 | 0.632 |
|  | **Weighted median** | 7 | 1.000 | 0.695 |
| **Streptococcus sanguinis** | **IVW** | 7 | 1.000 | 0.712 |
|  | **Simple mode** | 7 | 1.000 | 0.729 |
|  | **Weighted mode** | 7 | 1.000 | 0.727 |
|  | **MR Egger** | 6 | 1.053 | 0.387 |
|  | **Weighted median** | 6 | 1.000 | 0.490 |
| **Veillonella parvula** | **IVW** | 6 | 1.000 | 0.291 |
|  | **Simple mode** | 6 | 1.001 | 0.517 |
|  | **Weighted mode** | 6 | 1.000 | 0.702 |
|  | **MR Egger** | 7 | 0.993 | 0.579 |
|  | **Weighted median** | 7 | 1.000 | 0.884 |
| **Alloprevotella tannerae** | **IVW** | 7 | 1.000 | 0.983 |
|  | **Simple mode** | 7 | 0.999 | 0.542 |
|  | **Weighted mode** | 7 | 1.000 | 0.584 |
|  | **MR Egger** | 4 | 1.014 | 0.451 |
|  | **Weighted median** | 4 | 1.000 | 0.466 |
| **Filifactor alocis** | **IVW** | 4 | 1.000 | 0.406 |
|  | **Simple mode** | 4 | 1.000 | 0.584 |
|  | **Weighted mode** | 4 | 0.999 | 0.490 |
|  | **MR Egger** | 6 | 1.020 | 0.316 |
|  | **Weighted median** | 6 | 1.000 | 0.378 |
| **Fusobacterium nucleatum** | **IVW** | 6 | 0.999 | 0.021 |
|  | **Simple mode** | 6 | 1.000 | 0.804 |
|  | **Weighted mode** | 6 | 1.000 | 0.782 |
|  | **MR Egger** | 8 | 0.978 | 0.249 |
|  | **Weighted median** | 8 | 1.000 | 0.608 |
| **Parvimonas** | **IVW** | 8 | 1.001 | 0.117 |
|  | **Simple mode** | 8 | 1.000 | 0.926 |
|  | **Weighted mode** | 8 | 1.000 | 0.889 |
|  | **MR Egger** | 10 | 0.998 | 0.864 |
|  | **Weighted median** | 10 | 1.000 | 0.518 |
| **Porphyromonas gingivalis** | **IVW** | 10 | 0.999 | 0.116 |
|  | **Simple mode** | 10 | 1.000 | 0.814 |
|  | **Weighted mode** | 10 | 1.000 | 0.811 |
|  | **MR Egger** | 8 | 0.992 | 0.672 |
|  | **Weighted median** | 8 | 0.999 | 0.096 |
| **Prevotella intermedia** | **IVW** | 8 | 0.999 | 0.086 |
|  | **Simple mode** | 8 | 0.999 | 0.309 |
|  | **Weighted mode** | 8 | 0.999 | 0.298 |
|  | **MR Egger** | 6 | 0.993 | 0.389 |
|  | **Weighted median** | 6 | 0.999 | 0.248 |
| **Tannerella** | **IVW** | 6 | 1.000 | 0.992 |
|  | **Simple mode** | 6 | 0.999 | 0.465 |
|  | **Weighted mode** | 6 | 0.999 | 0.356 |
|  | **MR Egger** | 11 | 0.999 | 0.903 |
|  | **Weighted median** | 11 | 0.999 | 0.063 |
| **Squamous cell carcinoma** | **IVW** | 11 | 1.000 | 0.364 |
|  | **Simple mode** | 11 | 1.000 | 0.912 |
|  | **Weighted mode** | 11 | 0.999 | 0.163 |

**Table2 Sensitivity analysis of the Mendelian randomization analysis results**

| **Exposure Outcome** | **Method** | | **Oropharyngeal cancer** |
| --- | --- | --- | --- |
|  | **IVW (heterogeneity)** | ***p* value** | 0.835 |
|  |  | **Q** | 6.534 |
| **Actinomyces oris** | **MR Egger (heterogeneity)** | ***p* value** | 0.832 |
|  |  | **Q** | 5.795 |
|  | **MR Egger (pleiotropy)** | ***p* value** | 0.410 |
|  |  | **intercept** | 0.000 |
|  | **IVW (heterogeneity)** | ***p* value** | 0.469 |
|  |  | **Q** | 5.601 |
| **Capnocytophaga granulosa** | **MR Egger (heterogeneity)** | ***p* value** | 0.428 |
|  |  | **Q** | 4.898 |
|  | **MR Egger (pleiotropy)** | ***p* value** | 0.440 |
|  |  | **intercept** | 0.001 |
| **Capnocytophaga leadbetteri** | **IVW (heterogeneity)** | ***p* value** | 0.968 |
|  |  | **Q** | 0.002 |
|  | **IVW (heterogeneity)** | ***p* value** | 0.826 |
|  |  | **Q** | 5.098 |
| **Capnocytophaga sputigena** | **MR Egger (heterogeneity)** | ***p* value** | 0.751 |
|  |  | **Q** | 5.060 |
|  | **MR Egger (pleiotropy)** | ***p* value** | 0.850 |
|  |  | **intercept** | 0.000 |
|  | **IVW (heterogeneity)** | ***p* value** | 0.498 |
|  |  | **Q** | 11.363 |
| **Corynebacterium durum** | **MR Egger (heterogeneity)** | ***p* value** | 0.531 |
|  |  | **Q** | 9.995 |
|  | **MR Egger (pleiotropy)** | ***p* value** | 0.267 |
|  |  | **intercept** | 0.001 |
|  | **IVW (heterogeneity)** | ***p* value** | 0.958 |
|  |  | **Q** | 4.354 |
| **Gemella haemolysans** | **MR Egger (heterogeneity)** | ***p* value** | 0.936 |
|  |  | **Q** | 4.242 |
|  | **MR Egger (pleiotropy)** | ***p* value** | 0.746 |
|  |  | **intercept** | 0.000 |
|  | **IVW (heterogeneity)** | ***p* value** | 0.667 |
|  |  | **Q** | 4.939 |
| **Haemophilus parainfluenzae** | **MR Egger (heterogeneity)** | ***p* value** | 0.571 |
|  |  | **Q** | 4.790 |
|  | **MR Egger (pleiotropy)** | ***p* value** | 0.712 |
|  |  | **intercept** | 0.000 |
|  | **IVW (heterogeneity)** | ***p* value** | 0.723 |
|  |  | **Q** | 5.321 |
| **Kingella** | **MR Egger (heterogeneity)** | ***p* value** | 0.854 |
|  |  | **Q** | 3.321 |
|  | **MR Egger (pleiotropy)** | ***p* value** | 0.200 |
|  |  | **intercept** | 0.001 |
|  | **IVW (heterogeneity)** | ***p* value** | 0.396 |
|  |  | **Q** | 7.325 |
| **Neisseria flava** | **MR Egger (heterogeneity)** | ***p* value** | 0.293 |
|  |  | **Q** | 7.314 |
|  | **MR Egger (pleiotropy)** | ***p* value** | 0.930 |
|  |  | **intercept** | 0.000 |
|  | **IVW (heterogeneity)** | ***p* value** | 0.705 |
|  |  | **Q** | 3.788 |
| **Rothia aeria** | **MR Egger (heterogeneity)** | ***p* value** | 0.929 |
|  |  | **Q** | 1.353 |
|  | **MR Egger (pleiotropy)** | ***p* value** | 0.179 |
|  |  | **intercept** | 0.001 |
|  | **IVW (heterogeneity)** | ***p* value** | 0.972 |
|  |  | **Q** | 1.287 |
| **Streptococcus sanguinis** | **MR Egger (heterogeneity)** | ***p* value** | 0.962 |
|  |  | **Q** | 1.012 |
|  | **MR Egger (pleiotropy)** | ***p* value** | 0.623 |
|  |  | **intercept** | 0.001 |
|  | **IVW (heterogeneity)** | ***p* value** | 0.775 |
|  |  | **Q** | 2.512 |
| **Veillonella parvula** | **MR Egger (heterogeneity)** | ***p* value** | 0.811 |
|  |  | **Q** | 1.585 |
|  | **MR Egger (pleiotropy)** | ***p* value** | 0.390 |
|  |  | **intercept** | 0.005 |
|  | **IVW (heterogeneity)** | ***p* value** | 0.800 |
|  |  | **Q** | 3.068 |
| **Alloprevotella tannerae** | **MR Egger (heterogeneity)** | ***p* value** | 0.744 |
|  |  | **Q** | 2.716 |
|  | **MR Egger (pleiotropy)** | ***p* value** | 0.579 |
|  |  | **intercept** | 0.001 |
|  | **IVW (heterogeneity)** | ***p* value** | 0.506 |
|  |  | **Q** | 2.336 |
| **Filifactor alocis** | **MR Egger (heterogeneity)** | ***p* value** | 0.492 |
|  |  | **Q** | 1.420 |
|  | **MR Egger (pleiotropy)** | ***p* value** | 0.440 |
|  |  | **intercept** | 0.001 |
|  | **IVW (heterogeneity)** | ***p* value** | 0.376 |
|  |  | **Q** | 5.336 |
| **Fusobacterium nucleatum** | **MR Egger (heterogeneity)** | ***p* value** | 0.420 |
|  |  | **Q** | 3.901 |
|  | **MR Egger (pleiotropy)** | ***p* value** | 0.297 |
|  |  | **intercept** | 0.002 |
|  | **IVW (heterogeneity)** | ***p* value** | 0.874 |
|  |  | **Q** | 3.121 |
| **Parvimonas** | **MR Egger (heterogeneity)** | ***p* value** | 0.967 |
|  |  | **Q** | 1.386 |
|  | **MR Egger (pleiotropy)** | ***p* value** | 0.236 |
|  |  | **intercept** | 0.002 |
|  | **IVW (heterogeneity)** | ***p* value** | 0.206 |
|  |  | **Q** | 12.133 |
| **Porphyromonas gingivalis** | **MR Egger (heterogeneity)** | ***p* value** | 0.147 |
|  |  | **Q** | 12.106 |
|  | **MR Egger (pleiotropy)** | ***p* value** | 0.897 |
|  |  | **intercept** | 0.000 |
|  | **IVW (heterogeneity)** | ***p* value** | 0.629 |
|  |  | **Q** | 5.252 |
| **Prevotella intermedia** | **MR Egger (heterogeneity)** | ***p* value** | 0.533 |
|  |  | **Q** | 5.085 |
|  | **MR Egger (pleiotropy)** | ***p* value** | 0.697 |
|  |  | **intercept** | 0.001 |
|  | **IVW (heterogeneity)** | ***p* value** | 0.199 |
|  |  | **Q** | 7.307 |
| **Tannerella** | **MR Egger (heterogeneity)** | ***p* value** | 0.205 |
|  |  | **Q** | 5.919 |
|  | **MR Egger (pleiotropy)** | ***p* value** | 0.388 |
|  |  | **intercept** | 0.001 |
|  | **IVW (heterogeneity)** | ***p* value** | 0.019 |
|  |  | **Q** | 21.352 |
| **Treponema denticola** | **MR Egger (heterogeneity)** | ***p* value** | 0.011 |
|  |  | **Q** | 21.346 |
|  | **MR Egger (pleiotropy)** | ***p* value** | 0.962 |
|  |  | **intercept** | 0.000 |


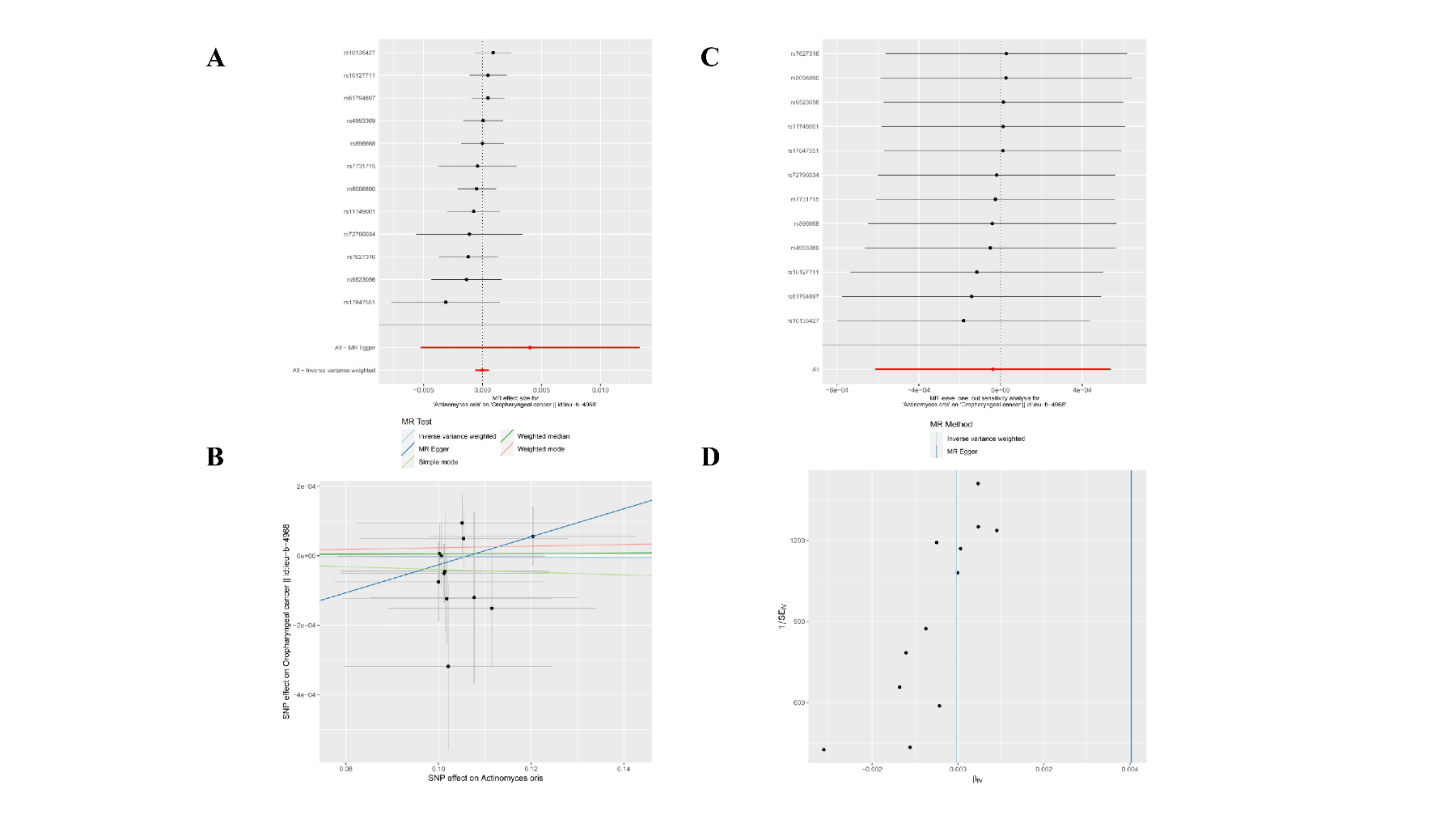


**Figure 1 Results and sensitivity analyses of the genetic correlation between** **Actinomyces oris and oropharyngeal cancer plotted in (A) forest plot; ( B ) scatterplot; (C) Leave-one-out sensitivity test; and (D) funnel plot.**


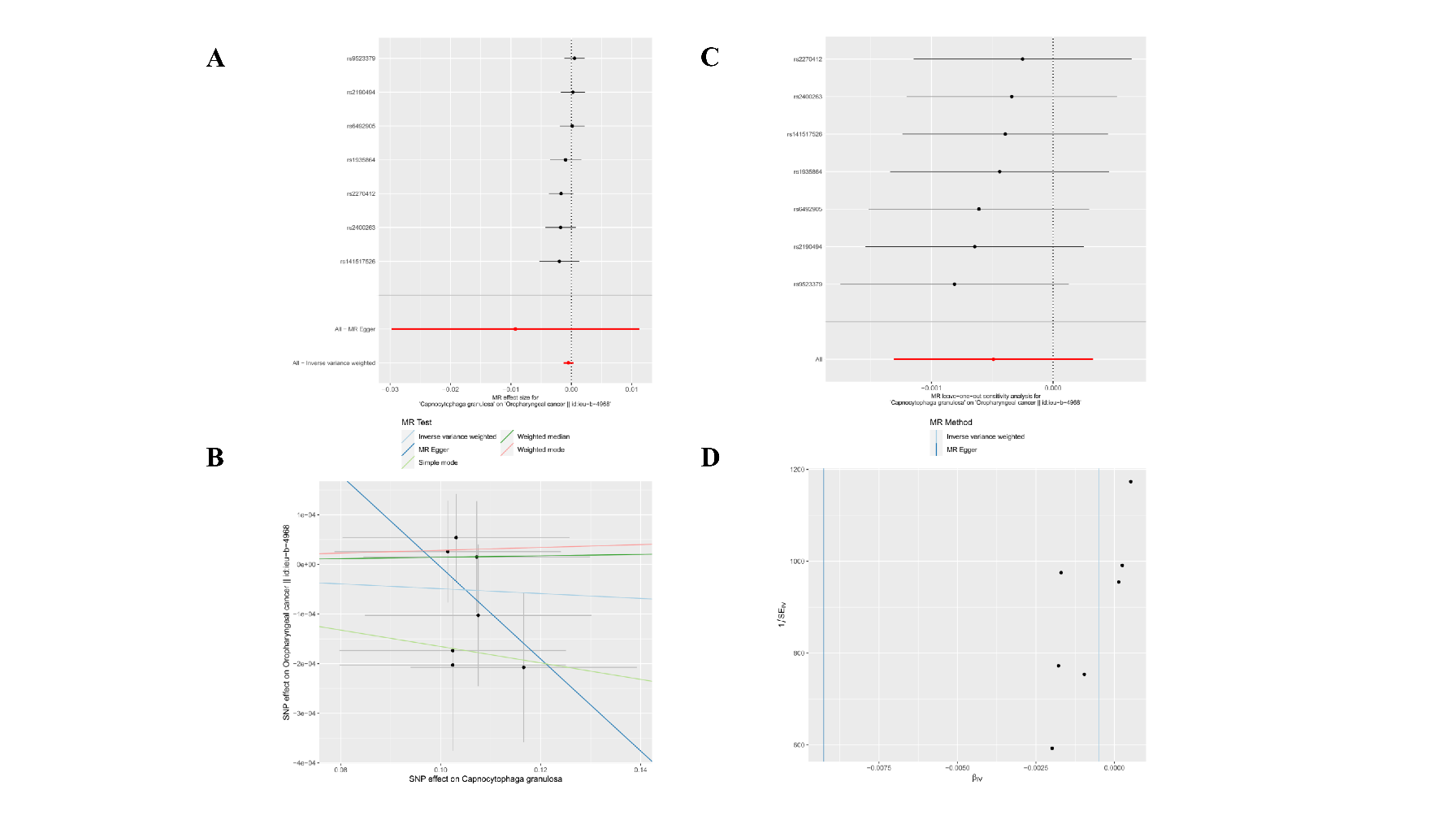


**Figure 2 Results and sensitivity analyses of the genetic correlation between** **Capnocytophaga granulosa and oropharyngeal cancer plotted in (A) forest plot; ( B ) scatterplot; (C) Leave-one-out sensitivity test; and (D) funnel plot.**


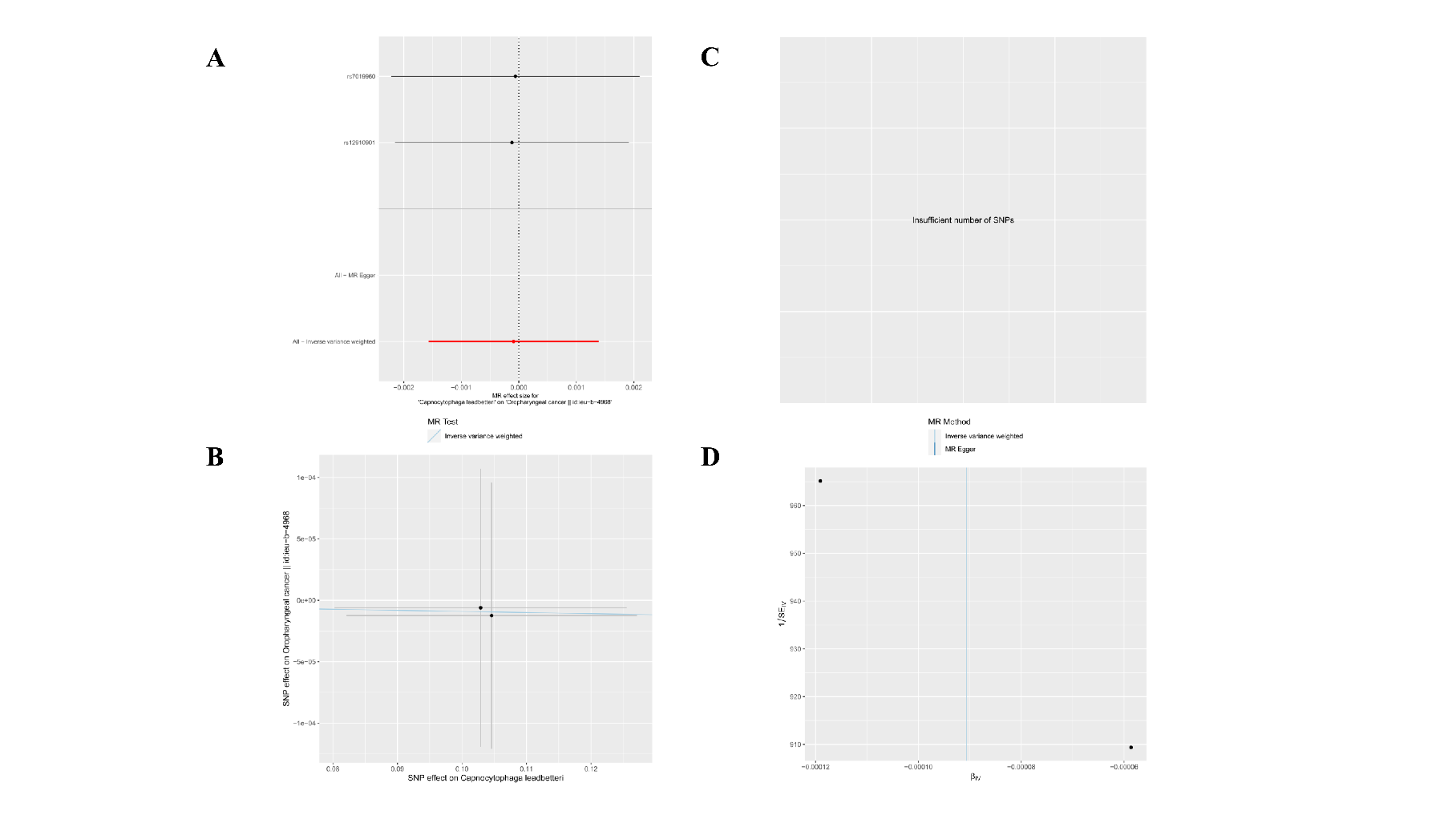


**Figure 3 Results and sensitivity analyses of the genetic correlation between** **Capnocytophaga leadbetteri and oropharyngeal cancer plotted in (A) forest plot; ( B ) scatterplot; (C) Leave-one-out sensitivity test; and (D) funnel plot.**


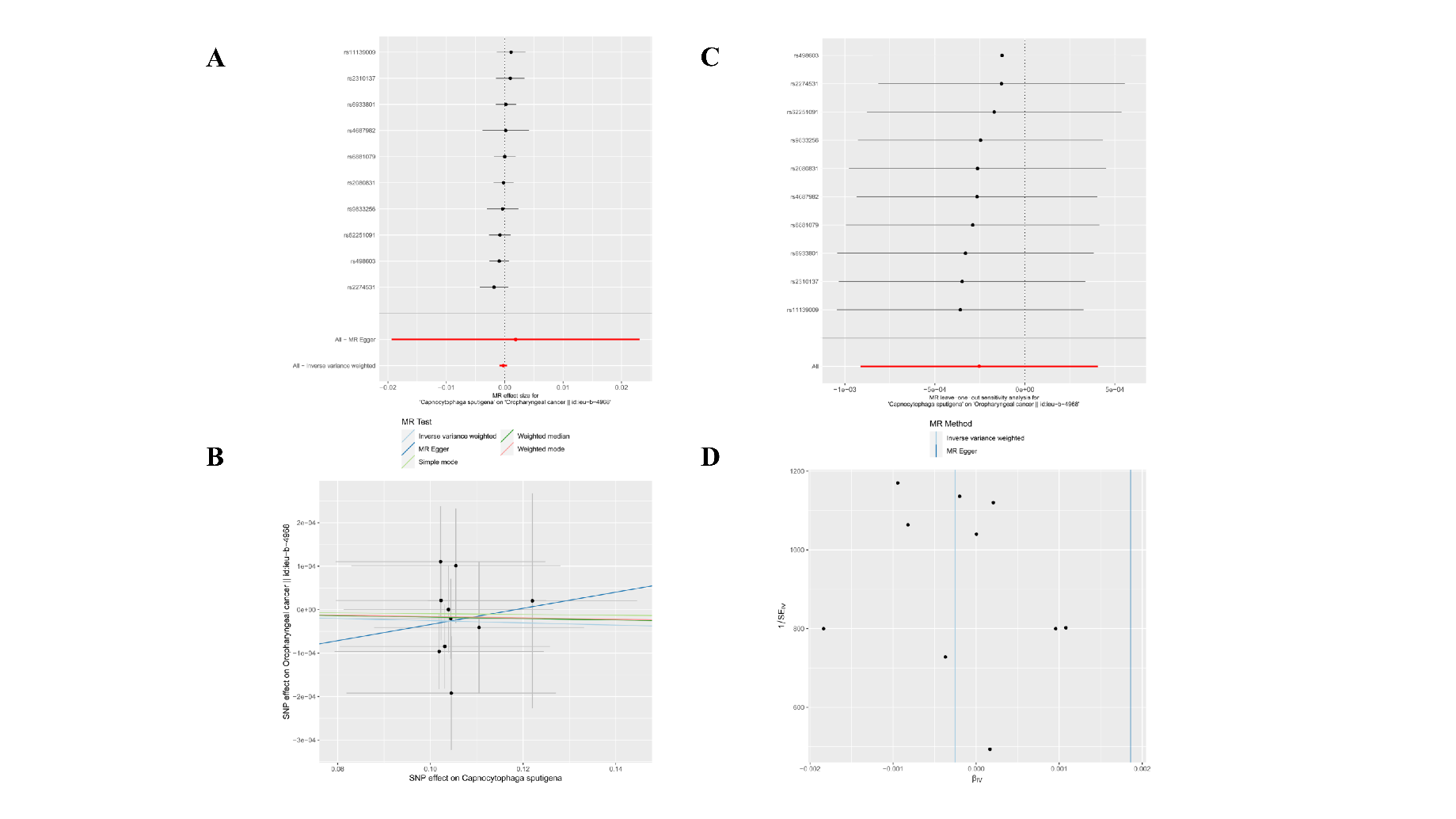


**Figure 4 Results and sensitivity analyses of the genetic correlation between** **Capnocytophaga sputigena and oropharyngeal cancer plotted in (A) forest plot; ( B ) scatterplot; (C) Leave-one-out sensitivity test; and (D) funnel plot.**


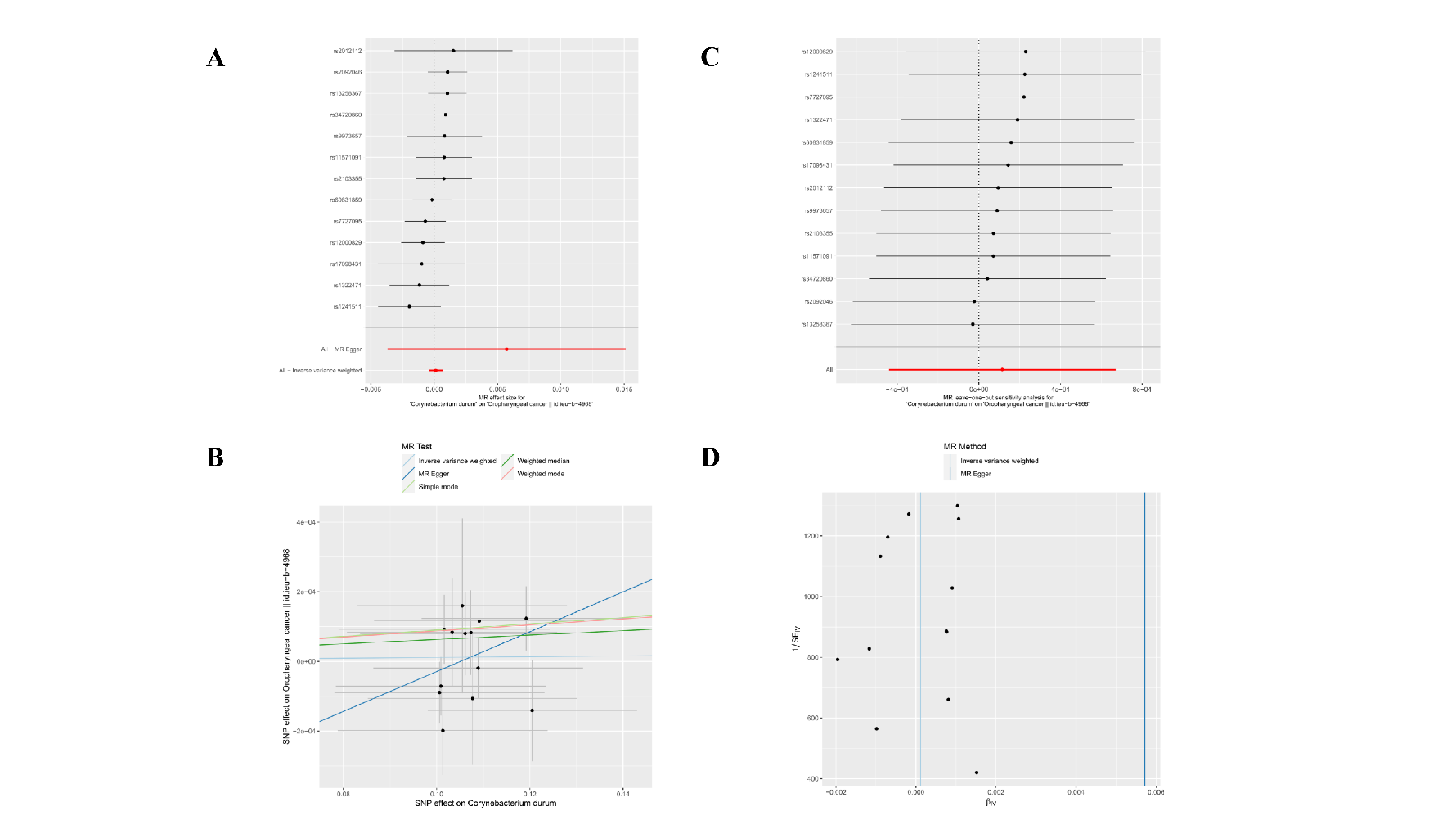


**Figure 5 Results and sensitivity analyses of the genetic correlation between** **Corynebacterium durum and oropharyngeal cancer plotted in (A) forest plot; ( B ) scatterplot; (C) Leave-one-out sensitivity test; and (D) funnel plot.**


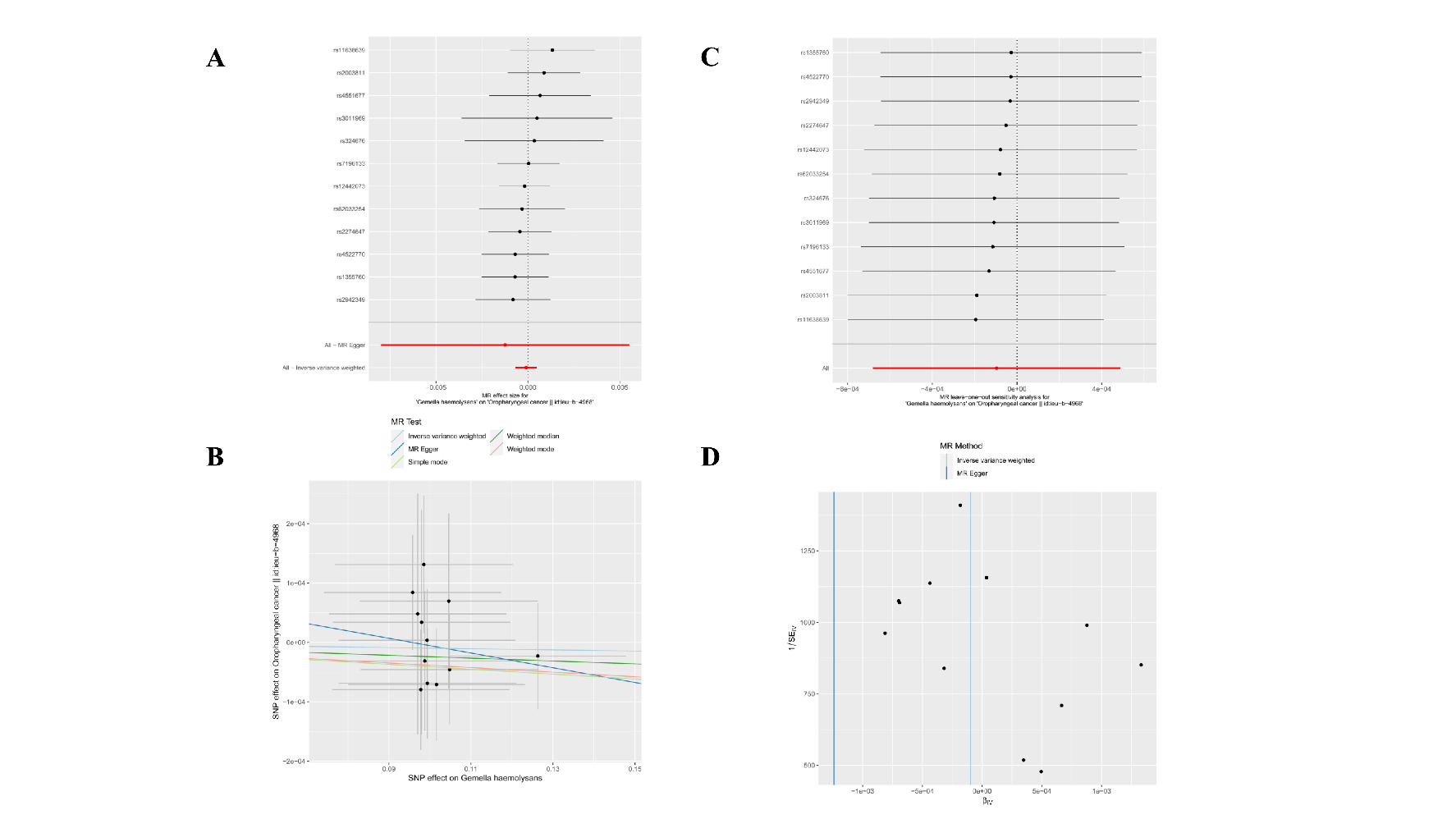


**Figure 6 Results and sensitivity analyses of the genetic correlation between** **Gemella haemolysans and oropharyngeal cancer plotted in (A) forest plot; ( B ) scatterplot; (C) Leave-one-out sensitivity test; and (D) funnel plot.**


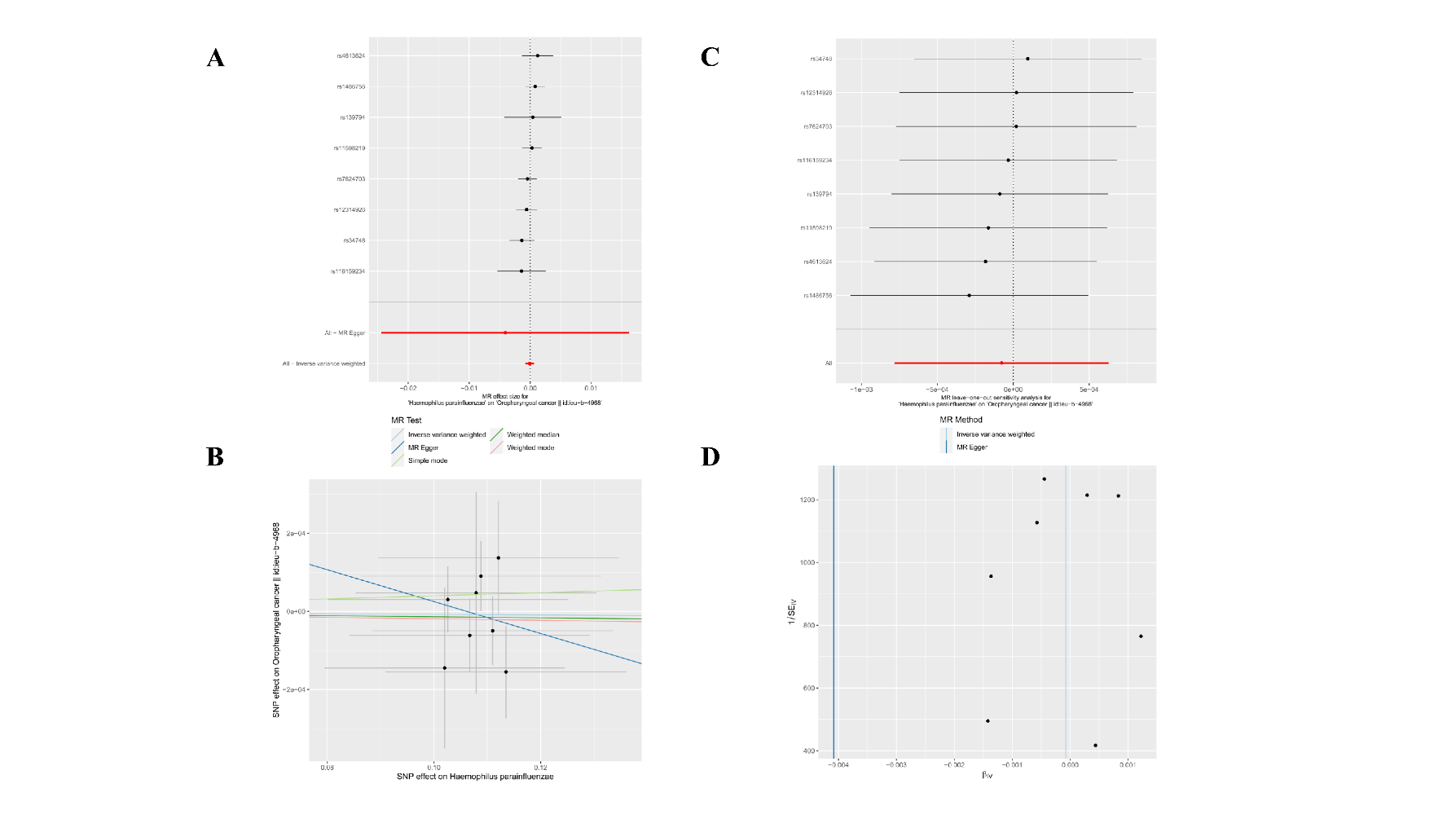


**Figure 7 Results and sensitivity analyses of the genetic correlation between** **Haemophilus parainfluenzae and oropharyngeal cancer plotted in (A) forest plot; ( B ) scatterplot; (C) Leave-one-out sensitivity test; and (D) funnel plot.**


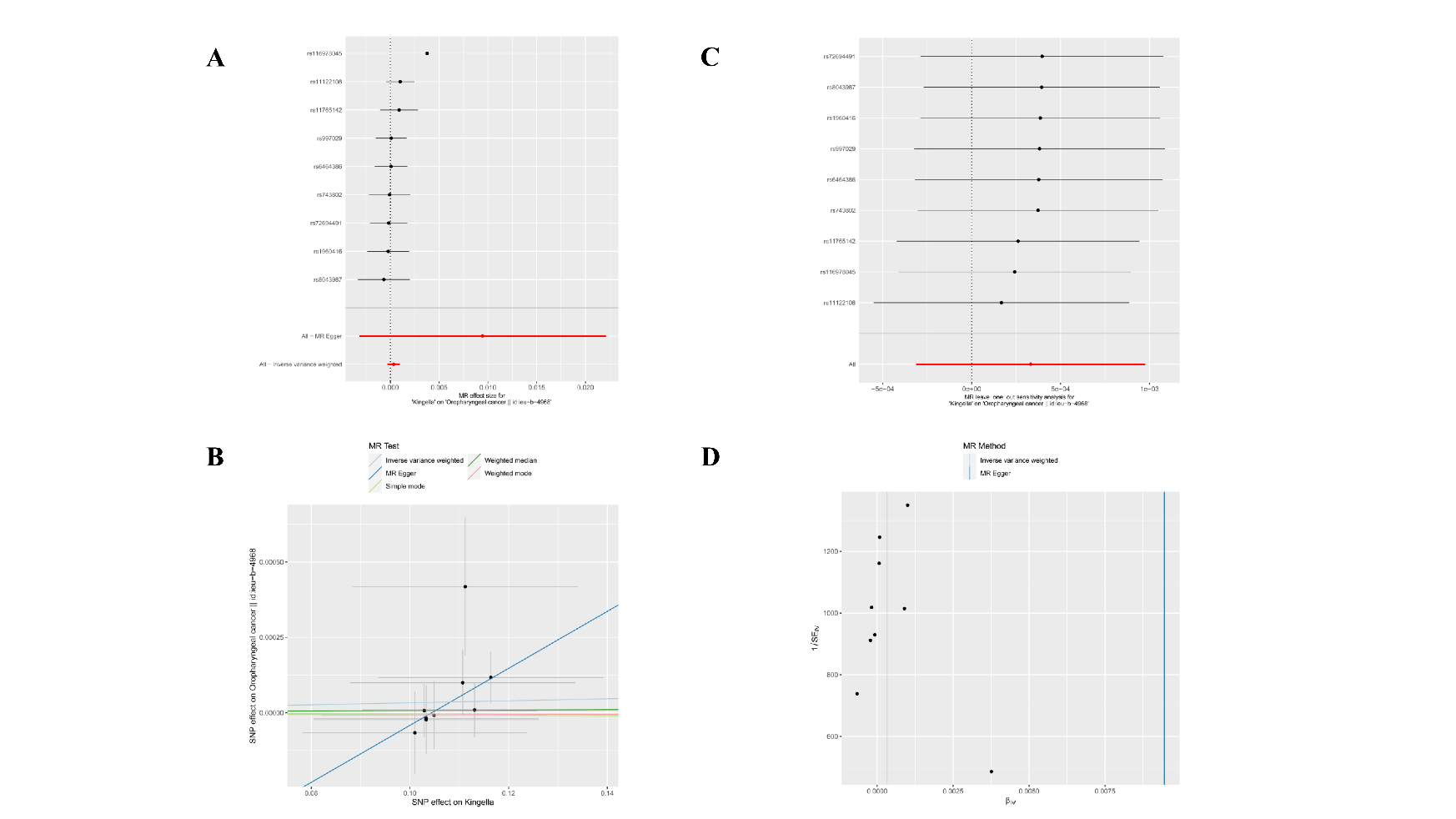


**Figure 8 Results and sensitivity analyses of the genetic correlation between** **Kingella and oropharyngeal cancer plotted in (A) forest plot; ( B ) scatterplot; (C) Leave-one-out sensitivity test; and (D) funnel plot.**


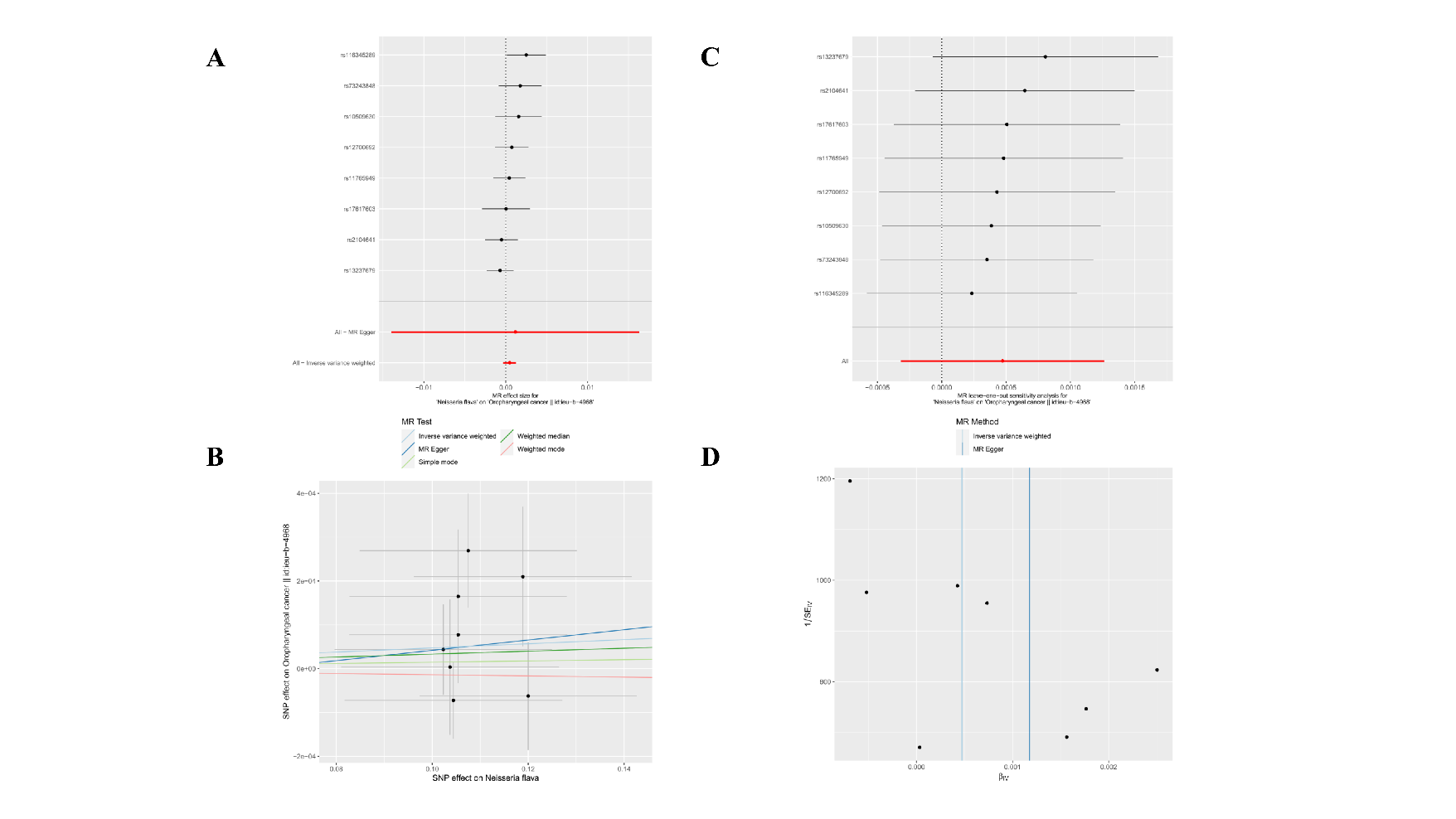


**Figure 9 Results and sensitivity analyses of the genetic correlation between** **Neisseria flava and oropharyngeal cancer plotted in (A) forest plot; ( B ) scatterplot; (C) Leave-one-out sensitivity test; and (D) funnel plot.**


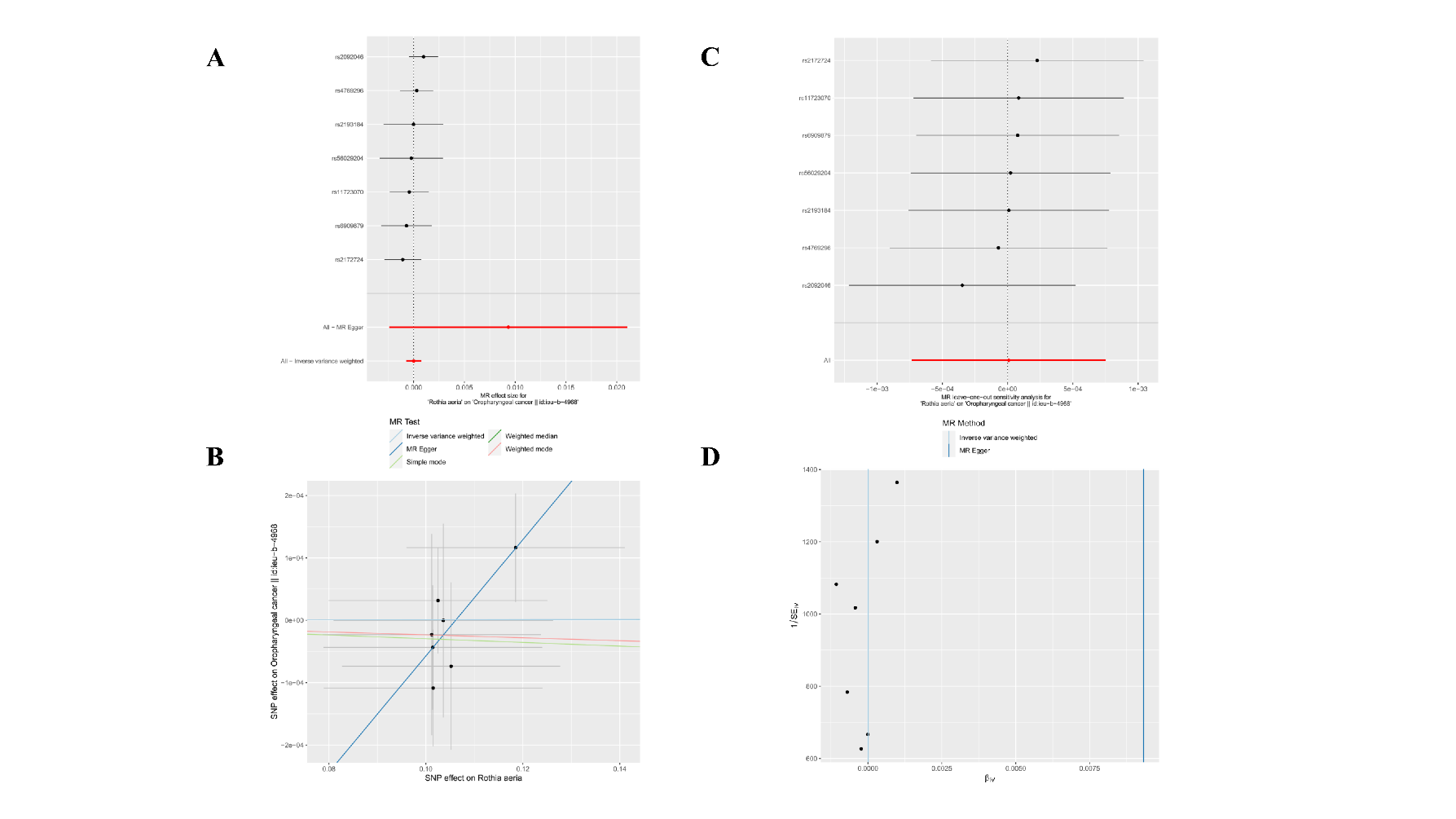


**Figure 10 Results and sensitivity analyses of the genetic correlation between** **Rothia aeria and oropharyngeal cancer plotted in (A) forest plot; ( B ) scatterplot; (C) Leave-one-out sensitivity test; and (D) funnel plot.**


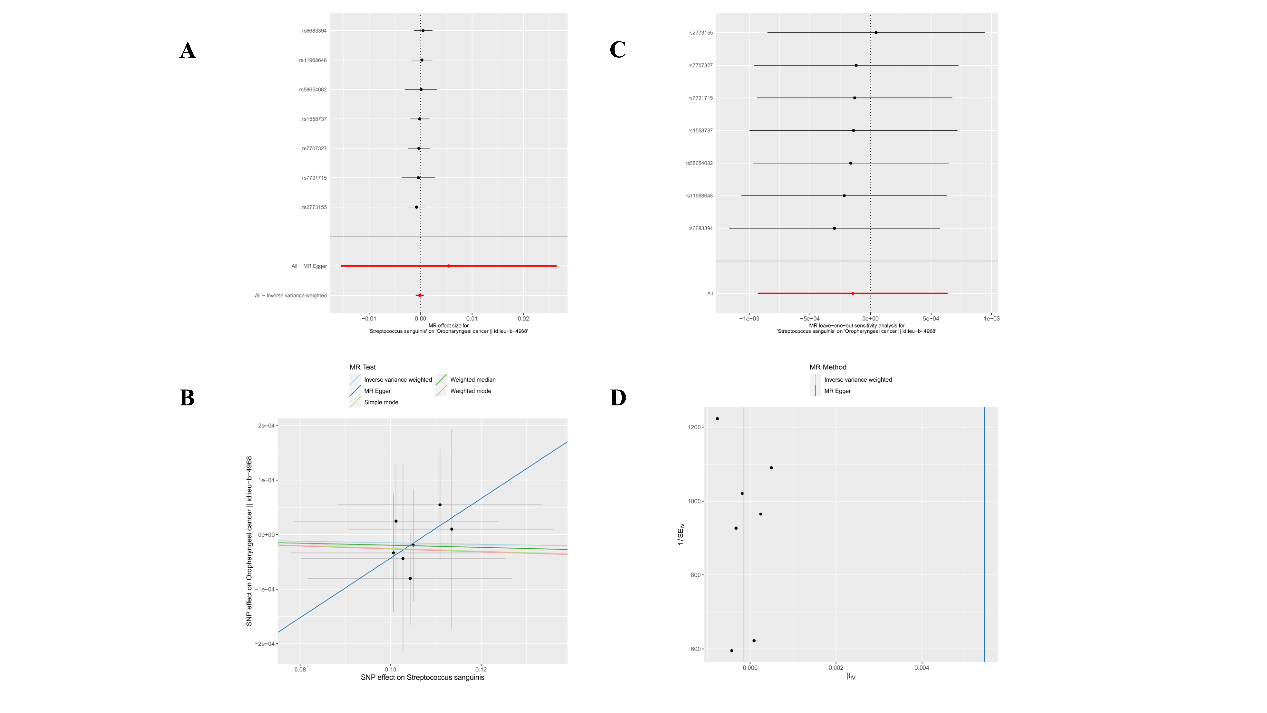


**Figure 11 Results and sensitivity analyses of the genetic correlation between** **Streptococcus sanguinis and oropharyngeal cancer plotted in (A) forest plot; ( B ) scatterplot; (C) Leave-one-out sensitivity test; and (D) funnel plot.**


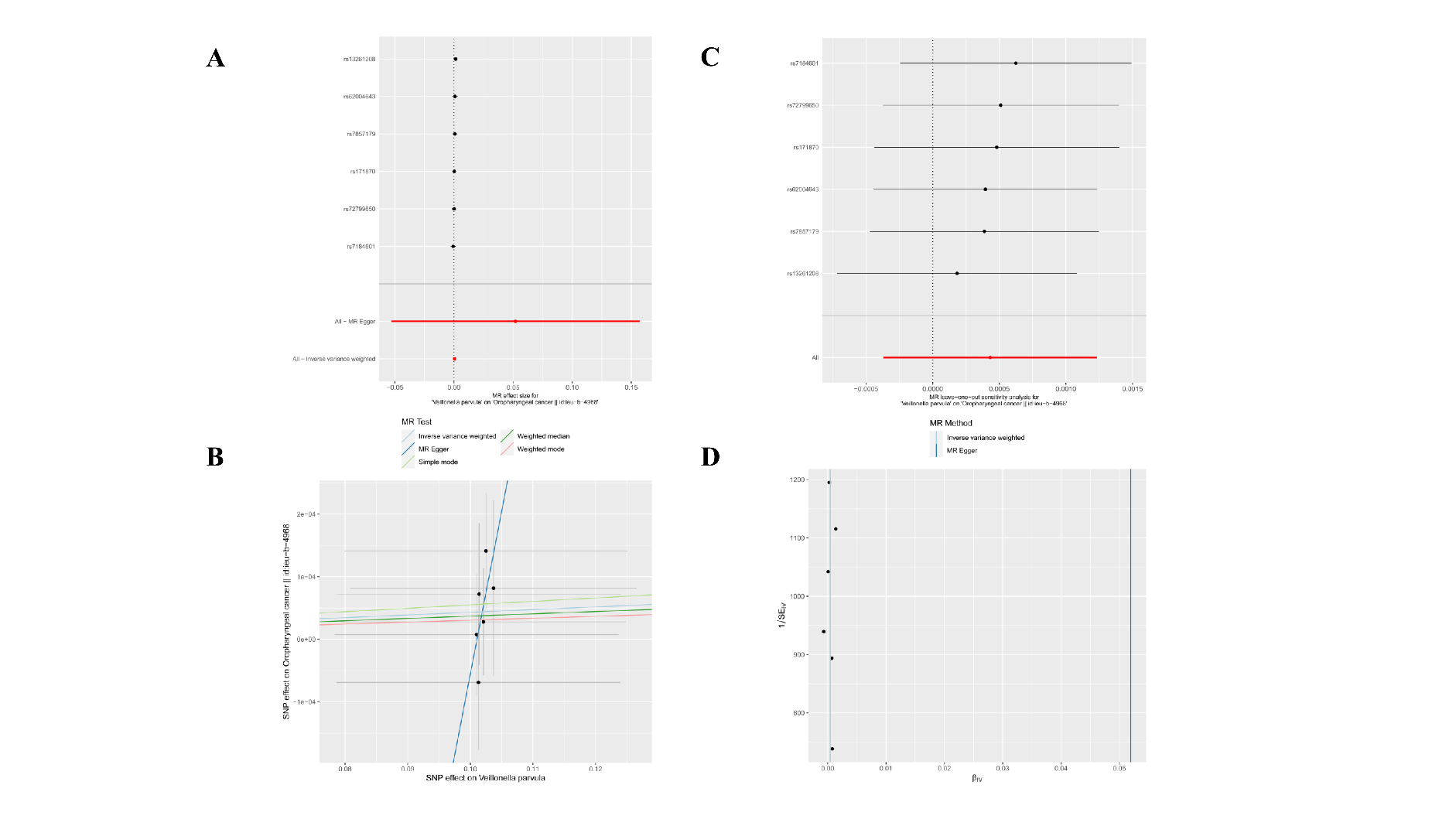


**Figure 12 Results and sensitivity analyses of the genetic correlation between** **Veillonella parvula and oropharyngeal cancer plotted in (A) forest plot; ( B ) scatterplot; (C) Leave-one-out sensitivity test; and (D) funnel plot.**


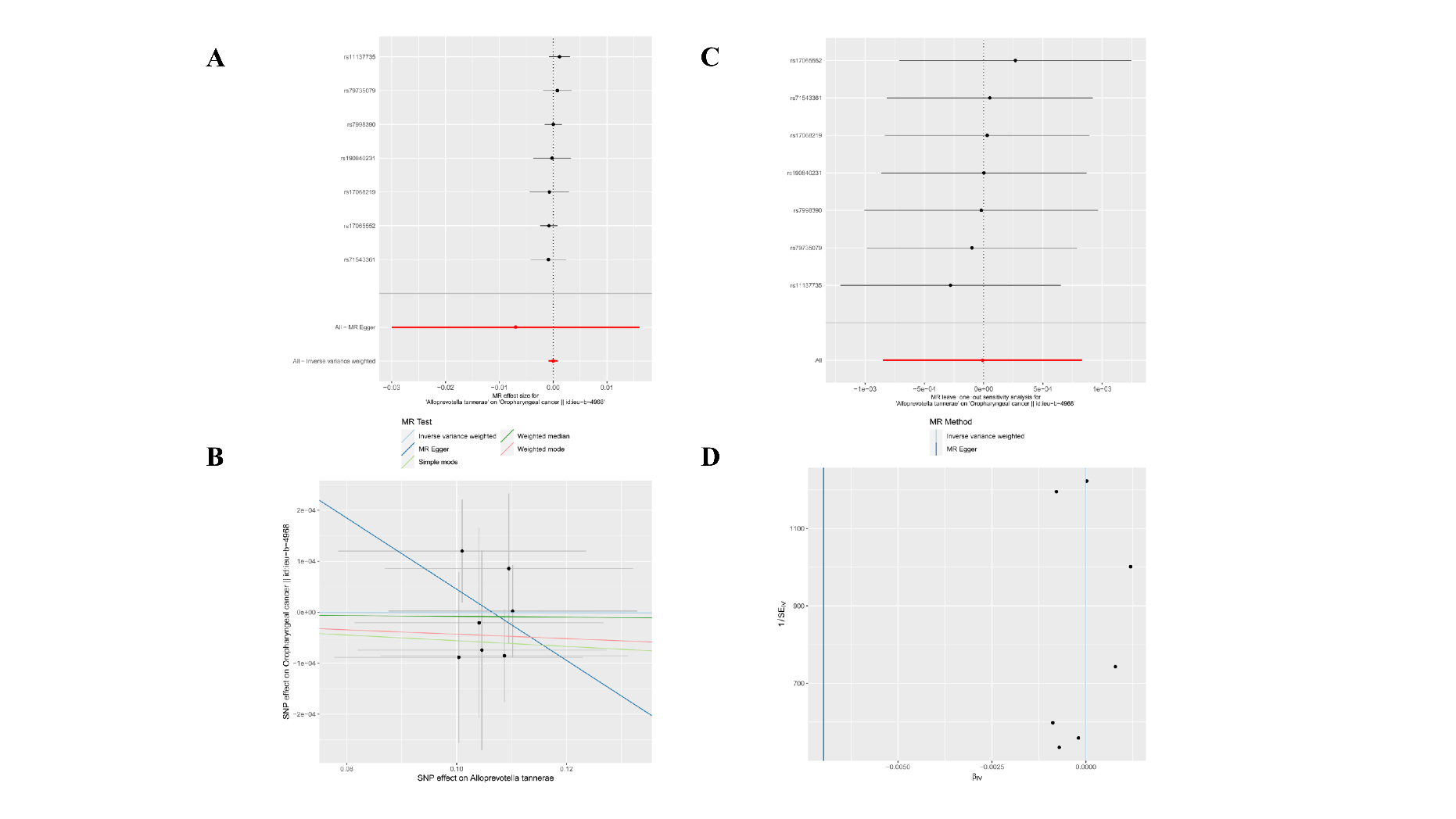


**Figure 13 Results and sensitivity analyses of the genetic correlation between** **Alloprevotella tannerae and oropharyngeal cancer plotted in (A) forest plot; ( B ) scatterplot; (C) Leave-one-out sensitivity test; and (D) funnel plot.**


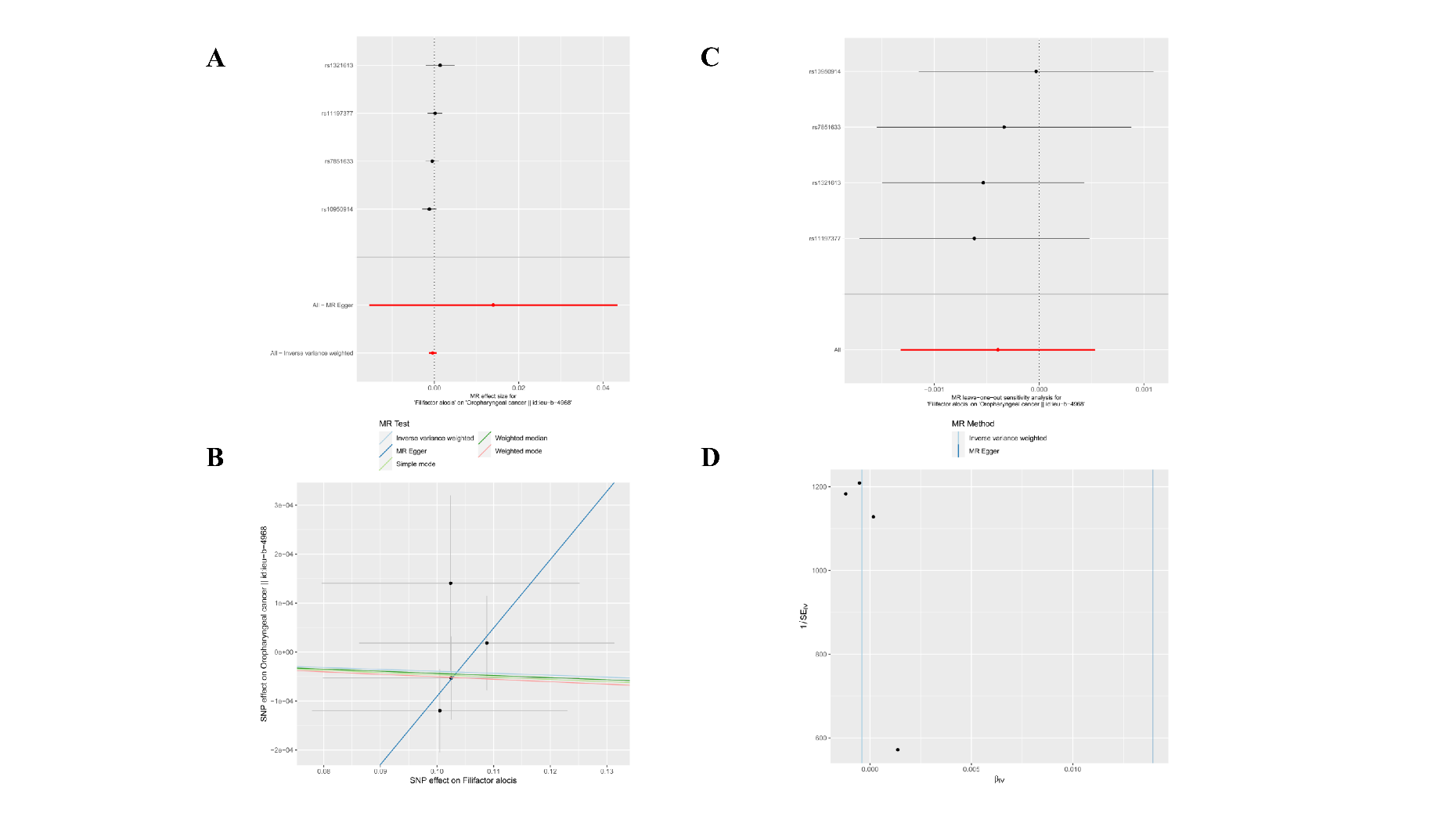


**Figure 14 Results and sensitivity analyses of the genetic correlation between** **Filifactor alocis and oropharyngeal cancer plotted in (A) forest plot; ( B ) scatterplot; (C) Leave-one-out sensitivity test; and (D) funnel plot.**


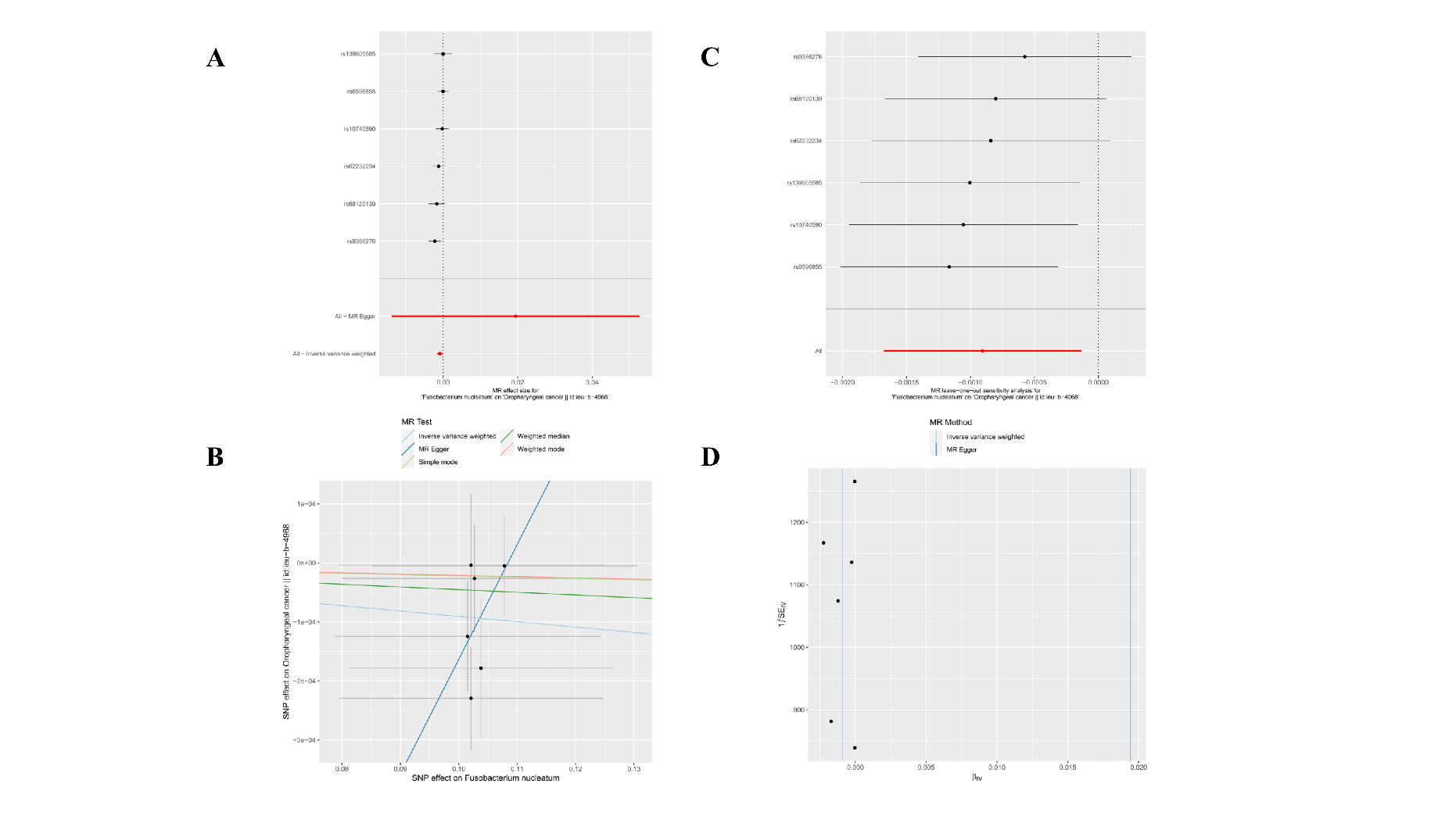


**Figure 15 Results and sensitivity analyses of the genetic correlation between** **Fusobacterium nucleatum and oropharyngeal cancer plotted in (A) forest plot; ( B ) scatterplot; (C) Leave-one-out sensitivity test; and (D) funnel plot.**


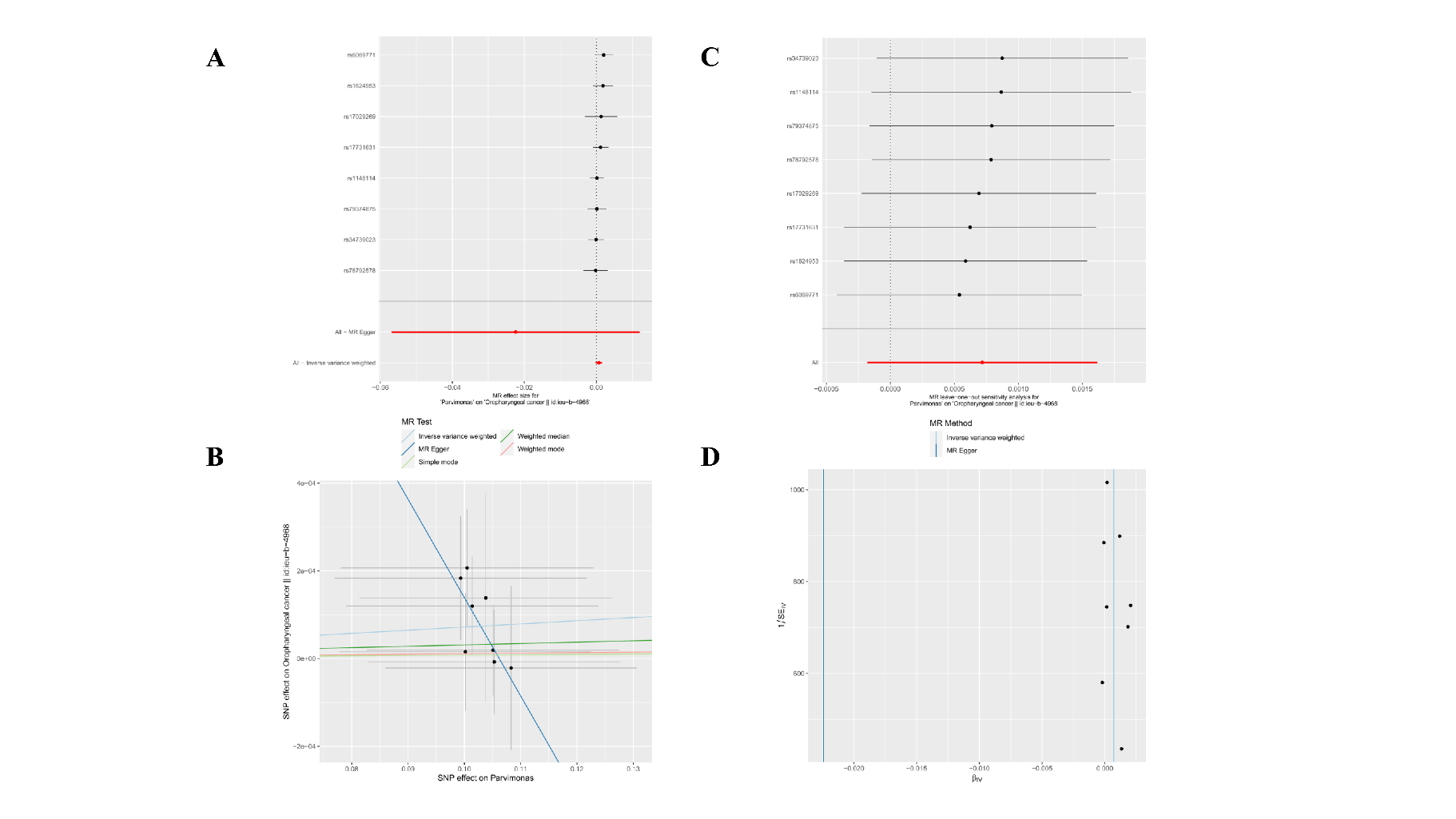


**Figure 16 Results and sensitivity analyses of the genetic correlation between** **Parvimonas and oropharyngeal cancer plotted in (A) forest plot; ( B ) scatterplot; (C) Leave-one-out sensitivity test; and (D) funnel plot.**


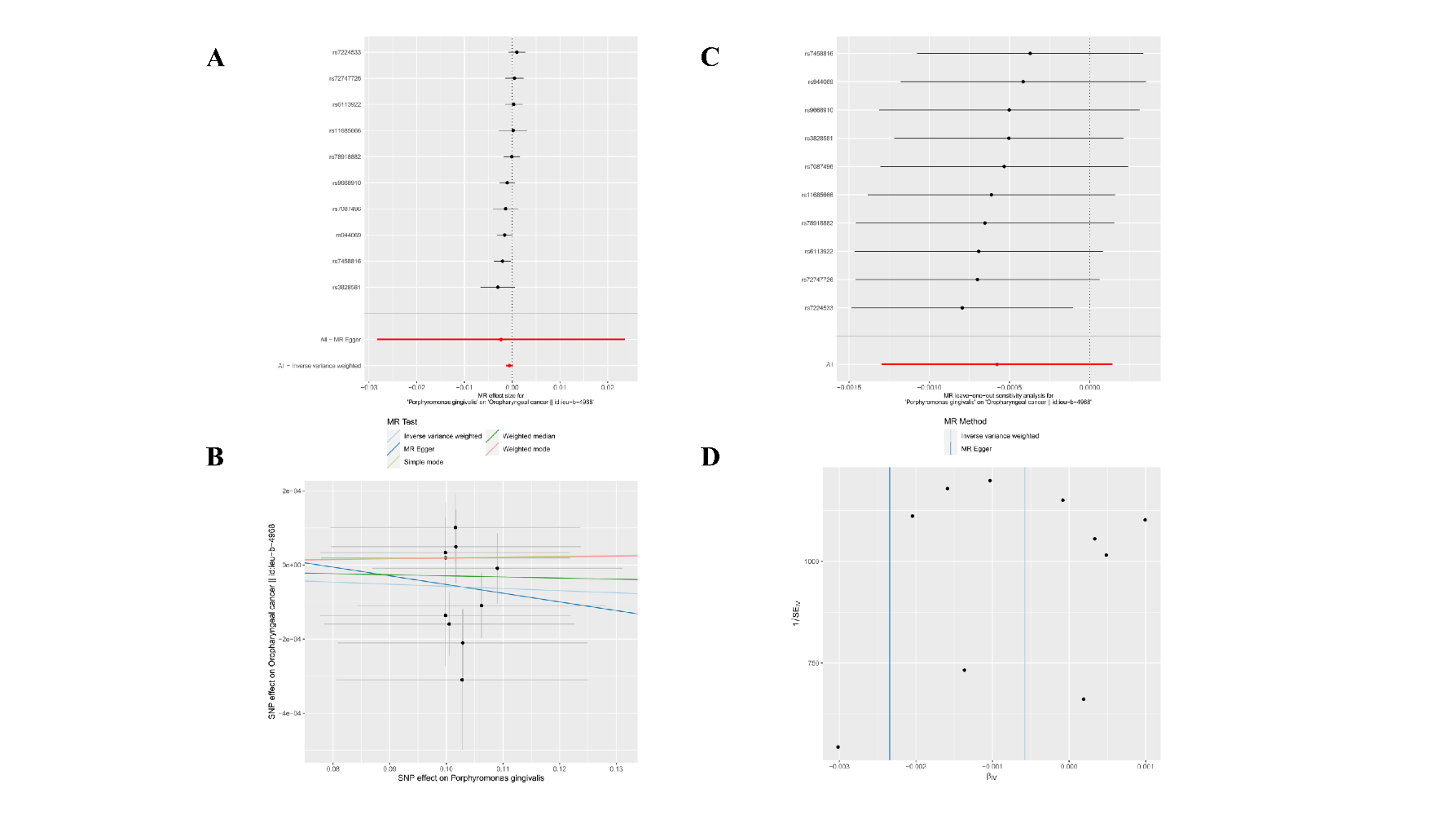


**Figure 17 Results and sensitivity analyses of the genetic correlation between** **Porphyromonas gingivalis and oropharyngeal cancer plotted in (A) forest plot; ( B ) scatterplot; (C) Leave-one-out sensitivity test; and (D) funnel plot.**


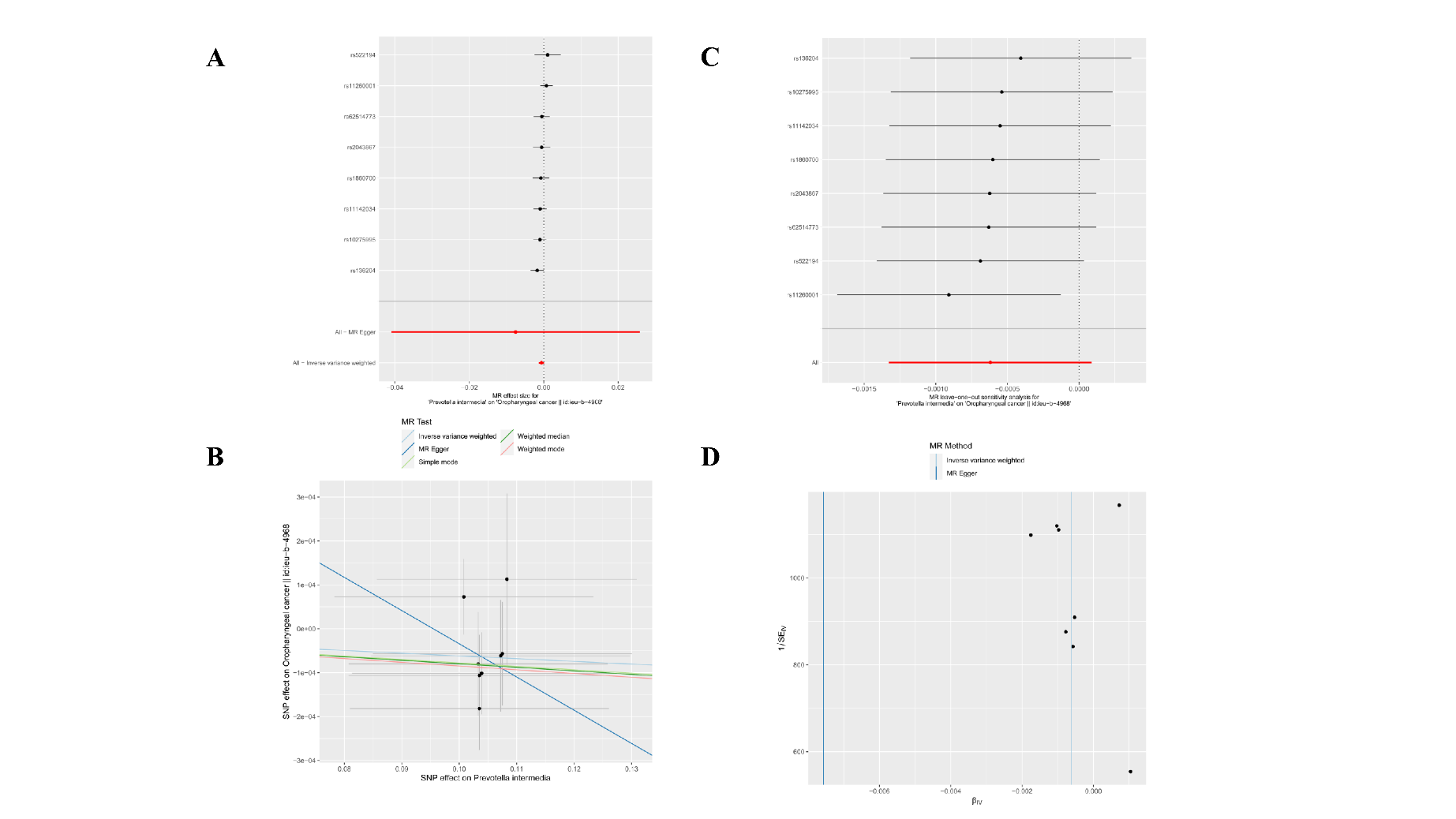


**Figure 18 Results and sensitivity analyses of the genetic correlation between** **Prevotella intermedia and oropharyngeal cancer plotted in (A) forest plot; ( B ) scatterplot; (C) Leave-one-out sensitivity test; and (D) funnel plot.**


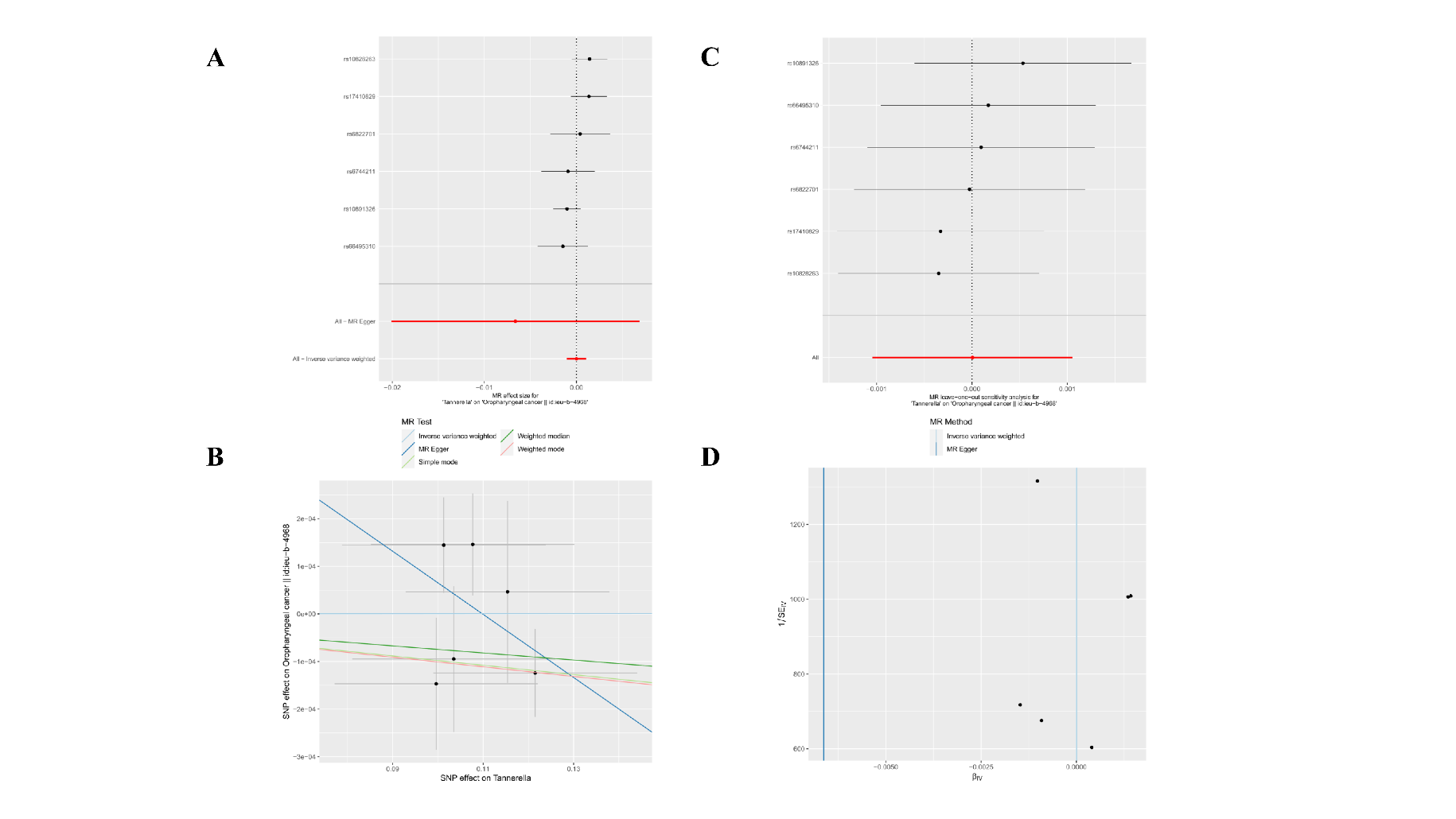


**Figure 19 Results and sensitivity analyses of the genetic correlation between** **Tannerella and oropharyngeal cancer plotted in (A) forest plot; ( B ) scatterplot; (C) Leave-one-out sensitivity test; and (D) funnel plot.**


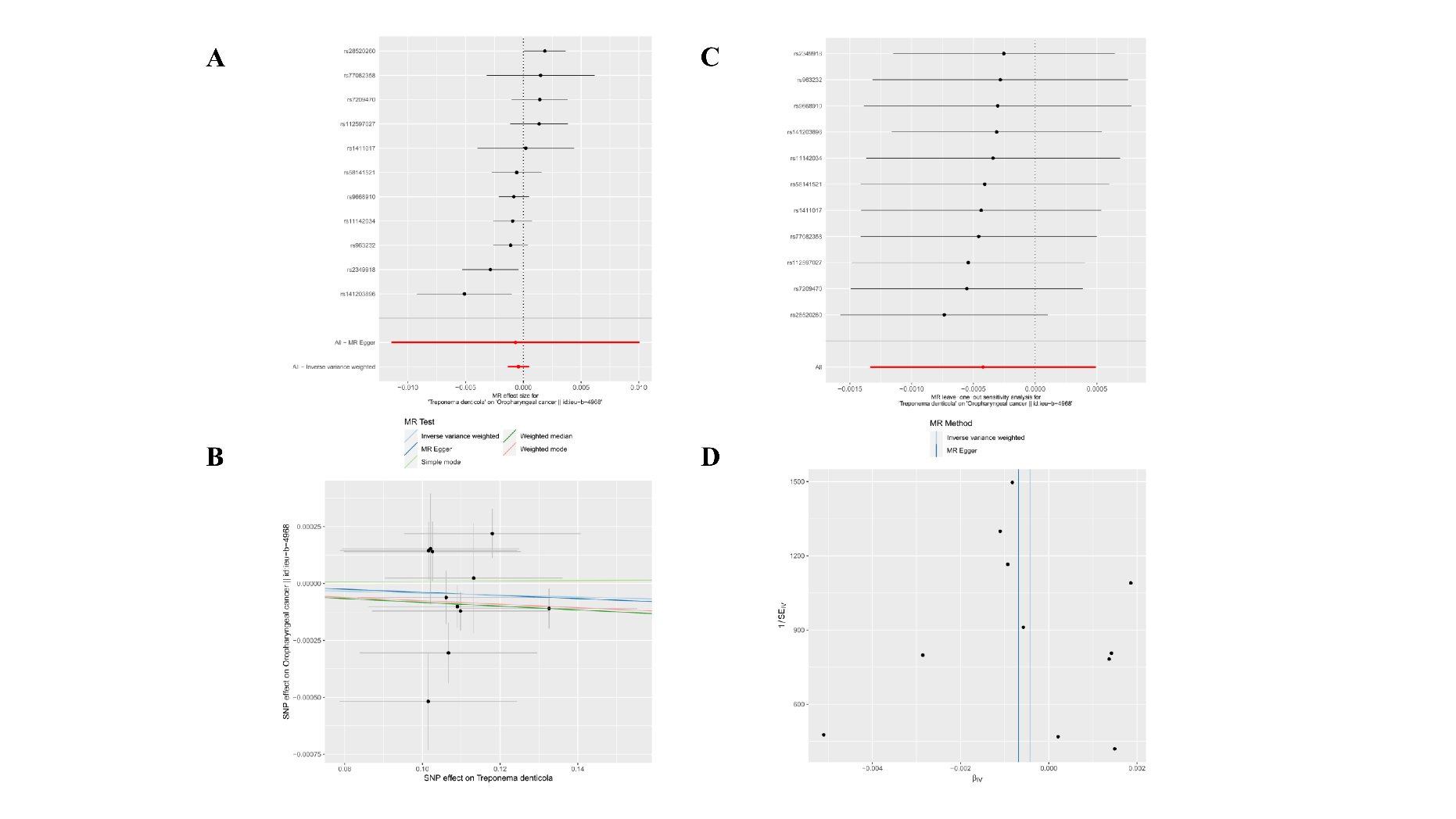


**Figure 20 Results and sensitivity analyses of the genetic correlation between** **Treponema denticola and oropharyngeal cancer plotted in (A) forest plot; ( B ) scatterplot; (C) Leave-one-out sensitivity test; and (D) funnel plot.**

S6. Mendelian randomization analysis of 9 immune cells and oropharyngeal cancer.

**Table 1** Results of Mendelian randomization analysis

**Table 2** Sensitivity analysis of the Mendelian randomization analysis results

**Figure 1-9** Results and sensitivity analyses plots of the genetic correlation between 9 immune cells and oropharyngeal cancer

**Table 1 Results of Mendelian randomization analysis**

| **Exposure Outcome** | **Method** | **Oropharyngeal cancer** | | |
| --- | --- | --- | --- | --- |
|  |  | **SNP (n)** | **OR** | ***P* Value** |
|  | **MR Egger** | 24 | 1.000 | 0.680 |
|  | **Weighted median** | 24 | 1.000 | 0.540 |
| **B cell** | **IVW** | 24 | 1.000 | 0.240 |
|  | **Simple mode** | 24 | 1.000 | 0.582 |
|  | **Weighted mode** | 24 | 1.000 | 0.793 |
|  | **MR Egger** | 12 | 1.000 | 0.331 |
|  | **Weighted median** | 12 | 1.000 | 0.995 |
| **B cell memory** | **IVW** | 12 | 1.000 | 0.457 |
|  | **Simple mode** | 12 | 1.000 | 0.732 |
|  | **Weighted mode** | 12 | 1.000 | 0.709 |
|  | **MR Egger** | 18 | 1.000 | 0.769 |
|  | **Weighted median** | 18 | 1.000 | 0.559 |
| **CD4+ T cell resting** | **IVW** | 18 | 1.000 | 0.202 |
|  | **Simple mode** | 18 | 1.000 | 0.536 |
|  | **Weighted mode** | 18 | 1.000 | 0.564 |
|  | **MR Egger** | 20 | 1.000 | 0.856 |
|  | **Weighted median** | 20 | 1.000 | 0.750 |
| **CD8+ T cell** | **IVW** | 20 | 1.000 | 0.268 |
|  | **Simple mode** | 20 | 1.000 | 0.369 |
|  | **Weighted mode** | 20 | 1.000 | 0.995 |
|  | **MR Egger** | 13 | 1.000 | 0.626 |
|  | **Weighted median** | 13 | 1.000 | 0.355 |
| **T cell** | **IVW** | 13 | 1.000 | 0.485 |
|  | **Simple mode** | 13 | 1.000 | 0.389 |
|  | **Weighted mode** | 13 | 1.000 | 0.373 |
|  | **MR Egger** | 18 | 0.999 | 0.139 |
|  | **Weighted median** | 18 | 1.000 | 0.776 |
| **B cell naive** | **IVW** | 18 | 1.000 | 0.584 |
|  | **Simple mode** | 18 | 1.000 | 0.906 |
|  | **Weighted mode** | 18 | 1.000 | 0.963 |
|  | **MR Egger** | 11 | 1.000 | 0.815 |
|  | **Weighted median** | 11 | 1.000 | 0.142 |
| **CD4+ T cell activated** | **IVW** | 11 | 1.000 | 0.435 |
|  | **Simple mode** | 11 | 1.000 | 0.278 |
|  | **Weighted mode** | 11 | 1.000 | 0.223 |
|  | **MR Egger** | 48 | 1.000 | 0.963 |
|  | **Weighted median** | 48 | 1.000 | 0.461 |
| **Neutrophil cell** | **IVW** | 48 | 1.000 | 0.370 |
|  | **Simple mode** | 48 | 1.001 | 0.598 |
|  | **Weighted mode** | 48 | 1.000 | 0.983 |
|  | **MR Egger** | 14 | 0.999 | 0.256 |
|  | **Weighted median** | 14 | 1.000 | 0.176 |
| **Plasma cell** | **IVW** | 14 | 1.000 | 0.082 |
|  | **Simple mode** | 14 | 1.000 | 0.466 |
|  | **Weighted mode** | 14 | 1.000 | 0.394 |

**Table2 Sensitivity analysis of the Mendelian randomization analysis results**

| **Exposure Outcome** | **Method** | | **Oropharyngeal cancer** |
| --- | --- | --- | --- |
|  | **IVW (heterogeneity)** | ***p* value** | 0.111 |
|  |  | **Q** | 31.497 |
| **B cell** | **MR Egger (heterogeneity)** | ***p* value** | 0.086 |
|  |  | **Q** | 31.495 |
|  | **MR Egger (pleiotropy)** | ***p* value** | 0.972 |
|  |  | **intercept** | 0.000 |
|  | **IVW (heterogeneity)** | ***p* value** | 0.116 |
|  |  | **Q** | 16.719 |
| **B cell memory** | **MR Egger (heterogeneity)** | ***p* value** | 0.210 |
|  |  | **Q** | 13.253 |
|  | **MR Egger (pleiotropy)** | ***p* value** | 0.137 |
|  |  | **intercept** | 0.000 |
|  | **IVW (heterogeneity)** | ***p* value** | 0.514 |
|  |  | **Q** | 16.136 |
| **CD4+ T cell resting** | **MR Egger (heterogeneity)** | ***p* value** | 0.446 |
|  |  | **Q** | 16.104 |
|  | **MR Egger (pleiotropy)** | ***p* value** | 0.862 |
|  |  | **intercept** | 0.000 |
|  | **IVW (heterogeneity)** | ***p* value** | 0.020 |
|  |  | **Q** | 33.746 |
| **CD8+ T cell** | **MR Egger (heterogeneity)** | ***p* value** | 0.015 |
|  |  | **Q** | 33.332 |
|  | **MR Egger (pleiotropy)** | ***p* value** | 0.642 |
|  |  | **intercept** | 0.000 |
|  | **IVW (heterogeneity)** | ***p* value** | 0.931 |
|  |  | **Q** | 5.683 |
| **T cell** | **MR Egger (heterogeneity)** | ***p* value** | 0.895 |
|  |  | **Q** | 5.658 |
|  | **MR Egger (pleiotropy)** | ***p* value** | 0.875 |
|  |  | **intercept** | 0.000 |
|  | **IVW (heterogeneity)** | ***p* value** | 0.904 |
|  |  | **Q** | 9.985 |
| **B cell naive** | **MR Egger (heterogeneity)** | ***p* value** | 0.954 |
|  |  | **Q** | 7.816 |
|  | **MR Egger (pleiotropy)** | ***p* value** | 0.160 |
|  |  | **intercept** | 0.000 |
|  | **IVW (heterogeneity)** | ***p* value** | 0.422 |
|  |  | **Q** | 10.217 |
| **CD4+ T cell activated** | **MR Egger (heterogeneity)** | ***p* value** | 0.413 |
|  |  | **Q** | 9.269 |
|  | **MR Egger (pleiotropy)** | ***p* value** | 0.362 |
|  |  | **intercept** | 0.000 |
|  | **IVW (heterogeneity)** | ***p* value** | 0.010 |
|  |  | **Q** | 72.690 |
| **Neutrophil cell** | **MR Egger (heterogeneity)** | ***p* value** | 0.008 |
|  |  | **Q** | 72.436 |
|  | **MR Egger (pleiotropy)** | ***p* value** | 0.690 |
|  |  | **intercept** | 0.000 |
|  | **IVW (heterogeneity)** | ***p* value** | 0.708 |
|  |  | **Q** | 9.824 |
| **Plasma cell** | **MR Egger (heterogeneity)** | ***p* value** | 0.651 |
|  |  | **Q** | 9.598 |
|  | **MR Egger (pleiotropy)** | ***p* value** | 0.643 |
|  |  | **intercept** | 0.000 |


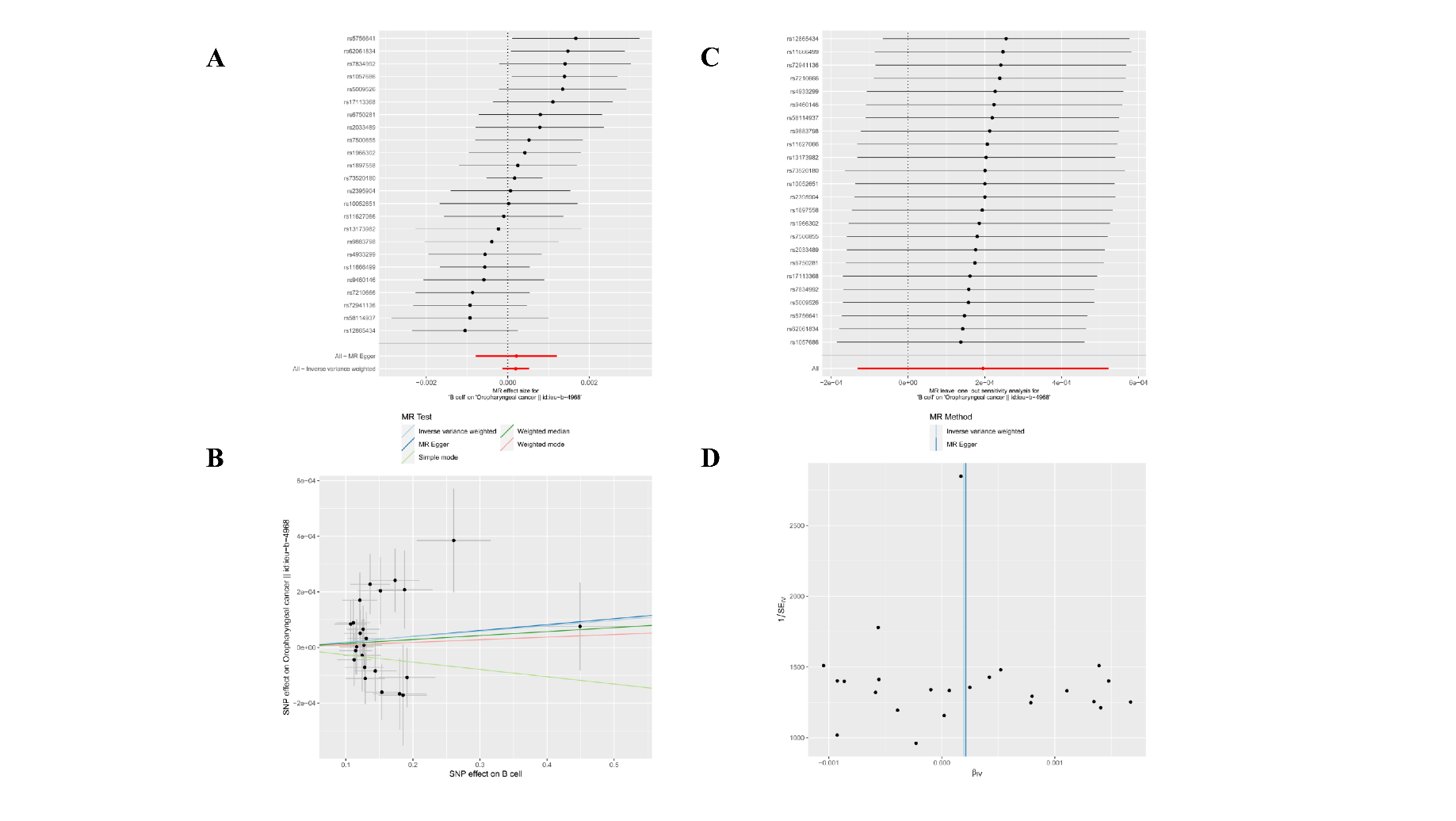


**Figure 1 Results and sensitivity analyses of the genetic correlation between** **B cell and oropharyngeal cancer plotted in (A) forest plot; ( B ) scatterplot; (C) Leave-one-out sensitivity test; and (D) funnel plot.**

**
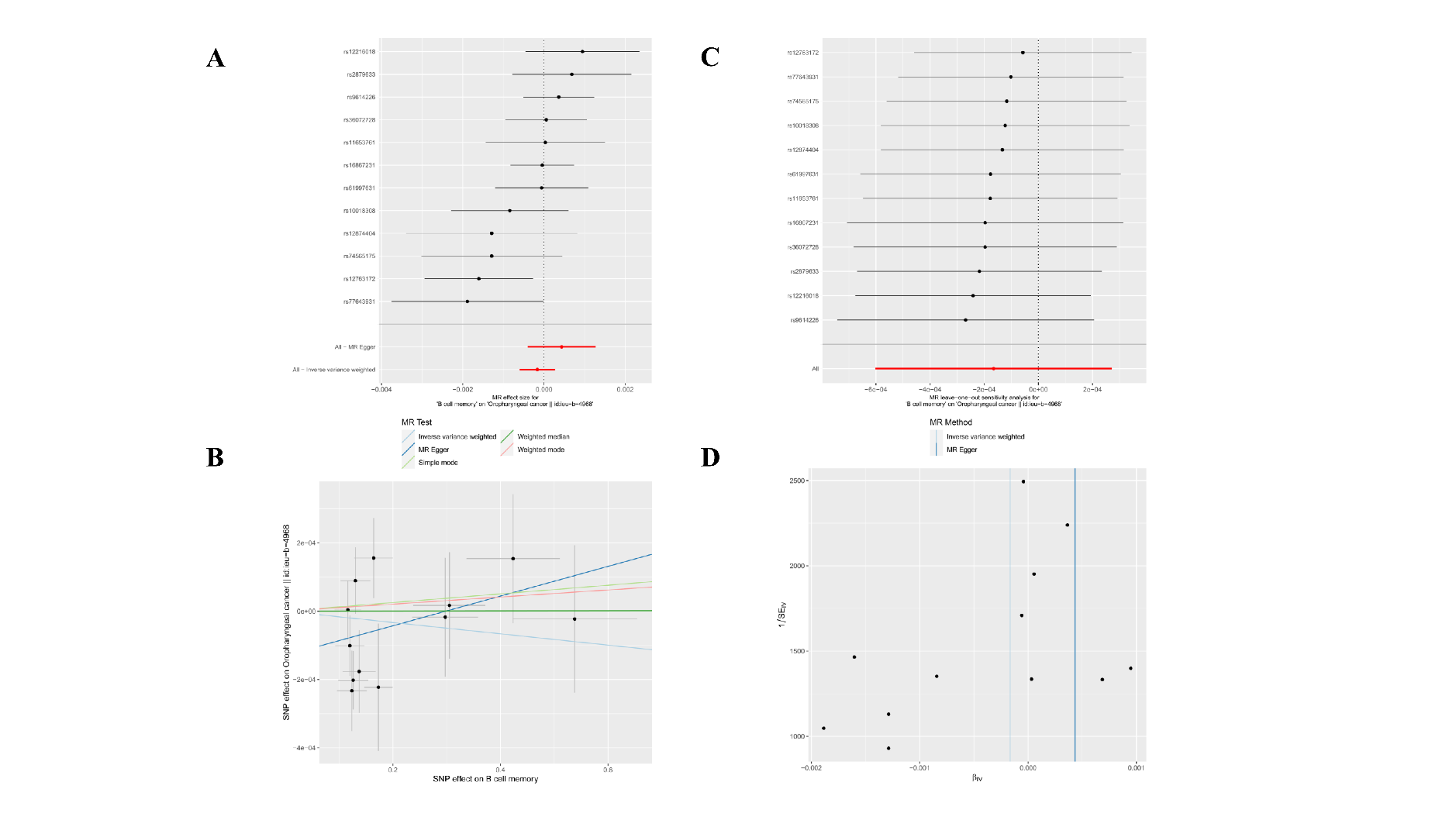
**

**Figure 2 Results and sensitivity analyses of the genetic correlation between B cell memory and oropharyngeal cancer plotted in (A) forest plot; ( B ) scatterplot; (C) Leave-one-out sensitivity test; and (D) funnel plot.**

**
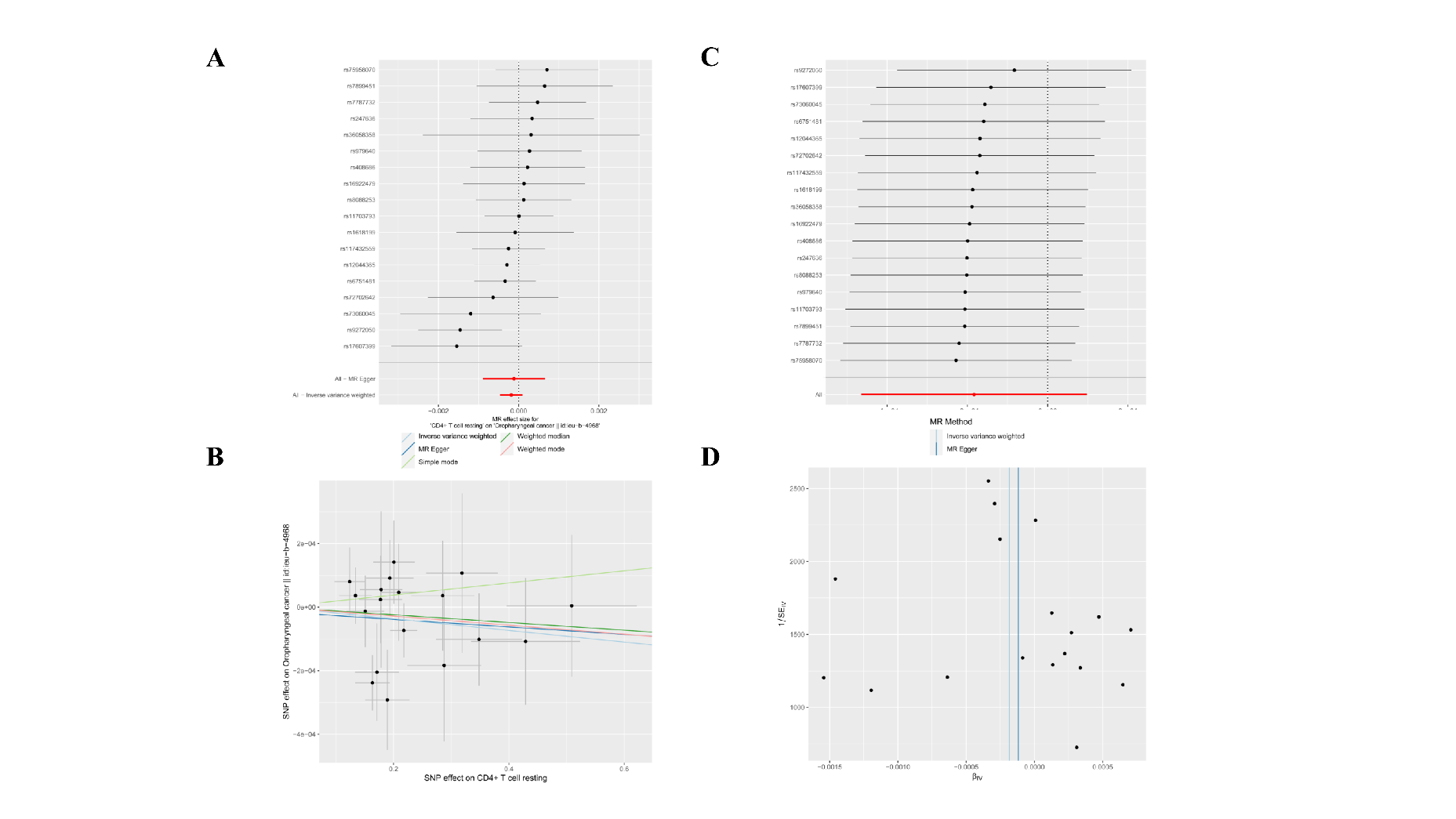
**

**Figure 3 Results and sensitivity analyses of the genetic correlation between CD4+ T cell resting and oropharyngeal cancer plotted in (A) forest plot; ( B ) scatterplot; (C) Leave-one-out sensitivity test; and (D) funnel plot.**

**
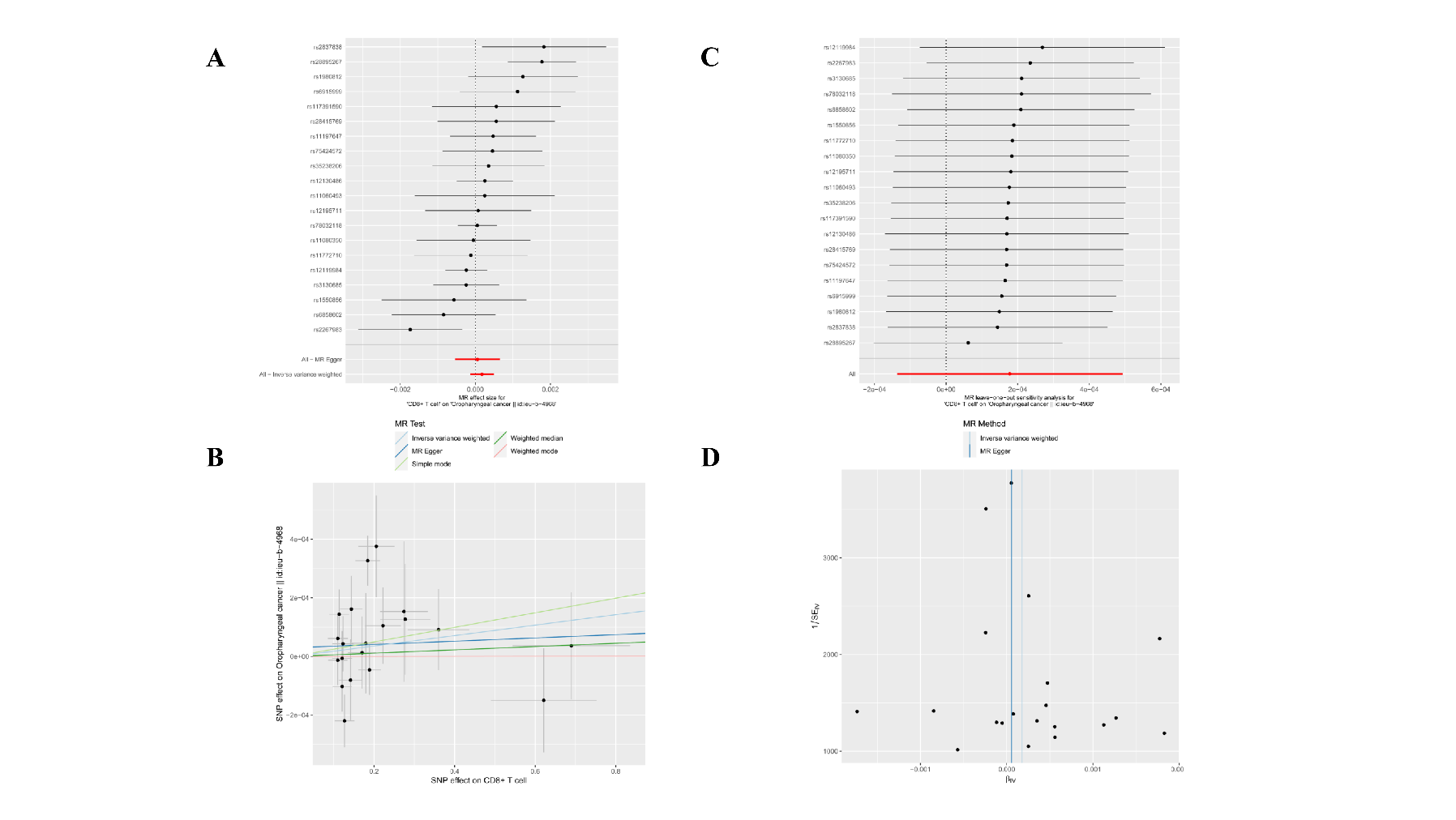
**

**Figure 4 Results and sensitivity analyses of the genetic correlation between CD8+ T cell and oropharyngeal cancer plotted in (A) forest plot; ( B ) scatterplot; (C) Leave-one-out sensitivity test; and (D) funnel plot.**

**
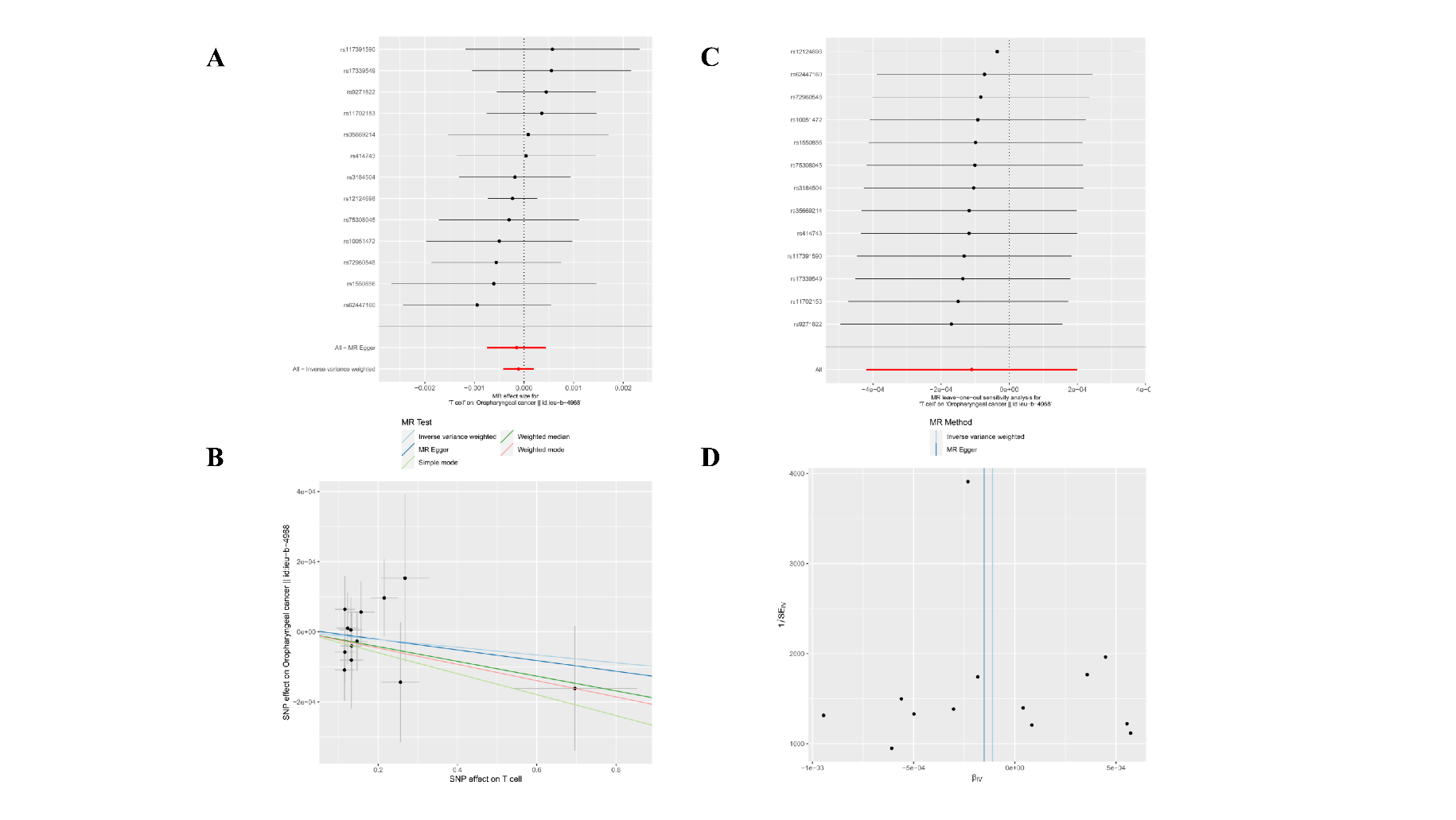
**

**Figure 5 Results and sensitivity analyses of the genetic correlation between T cell and oropharyngeal cancer plotted in (A) forest plot; ( B ) scatterplot; (C) Leave-one-out sensitivity test; and (D) funnel plot.**

**
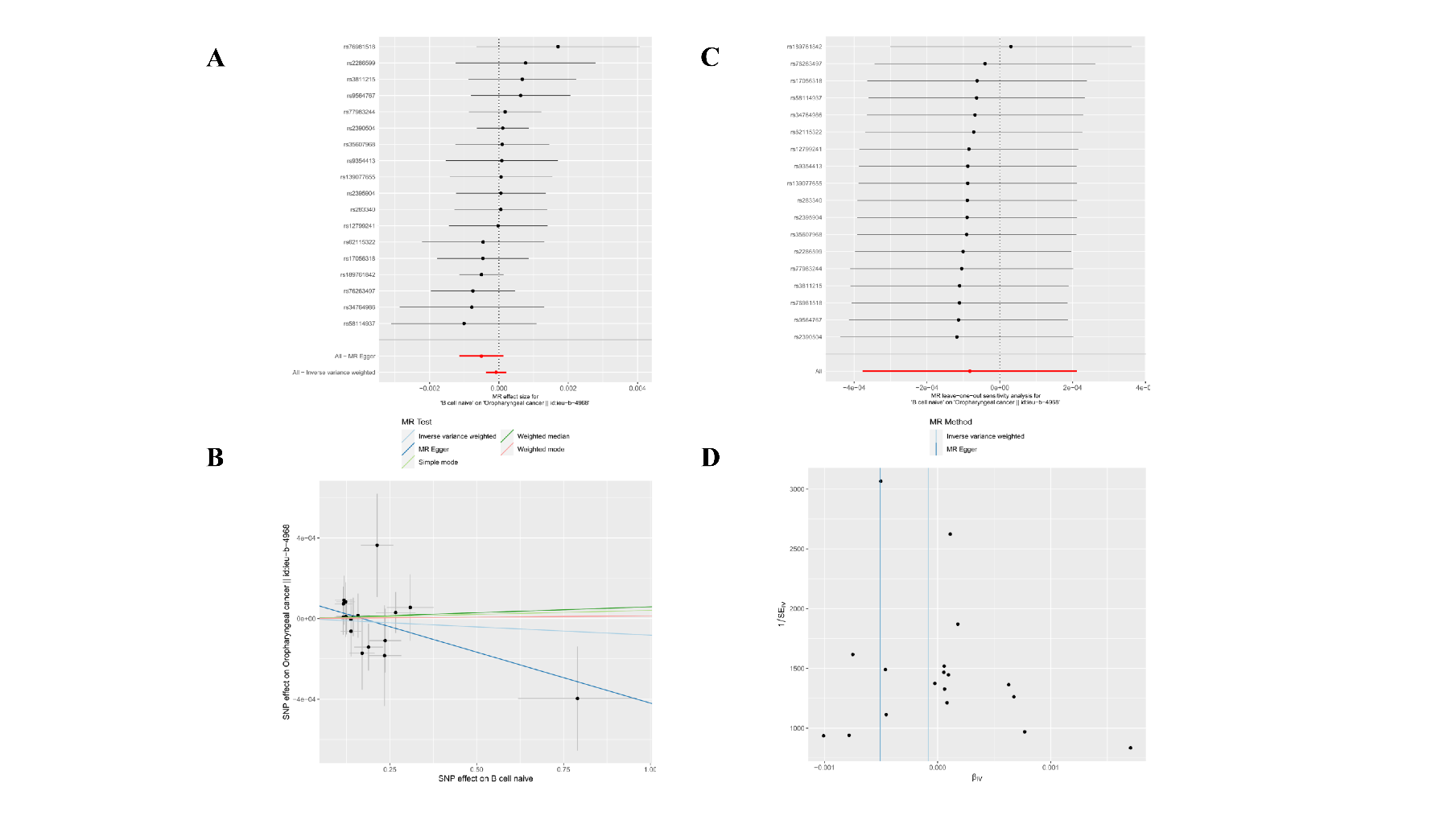
**

**Figure 6 Results and sensitivity analyses of the genetic correlation between B cell naive and oropharyngeal cancer plotted in (A) forest plot; ( B ) scatterplot; (C) Leave-one-out sensitivity test; and (D) funnel plot.**

**
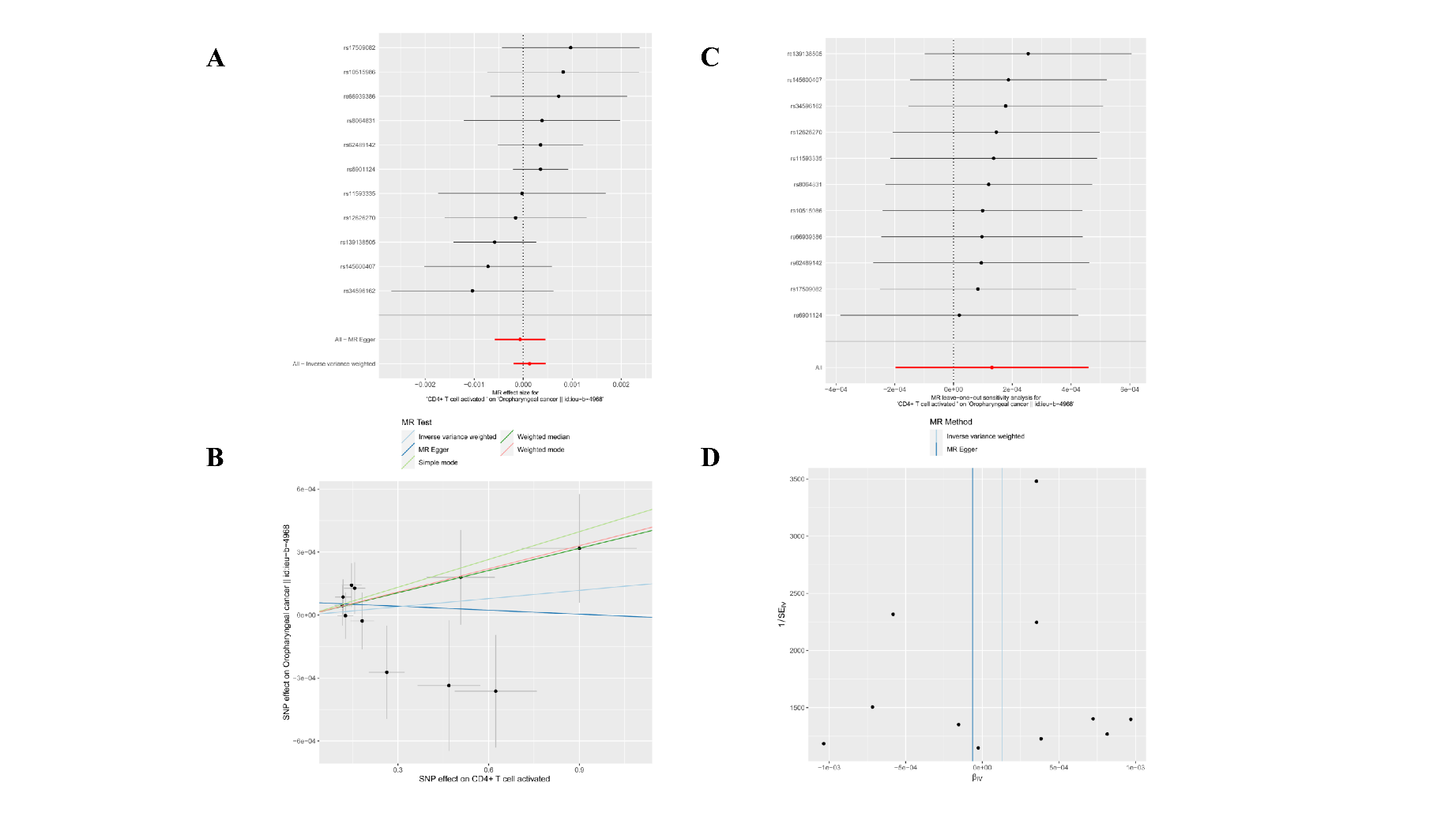
**

**Figure 7 Results and sensitivity analyses of the genetic correlation between CD4+ T cell activated and oropharyngeal cancer plotted in (A) forest plot; ( B ) scatterplot; (C) Leave-one-out sensitivity test; and (D) funnel plot.**

**
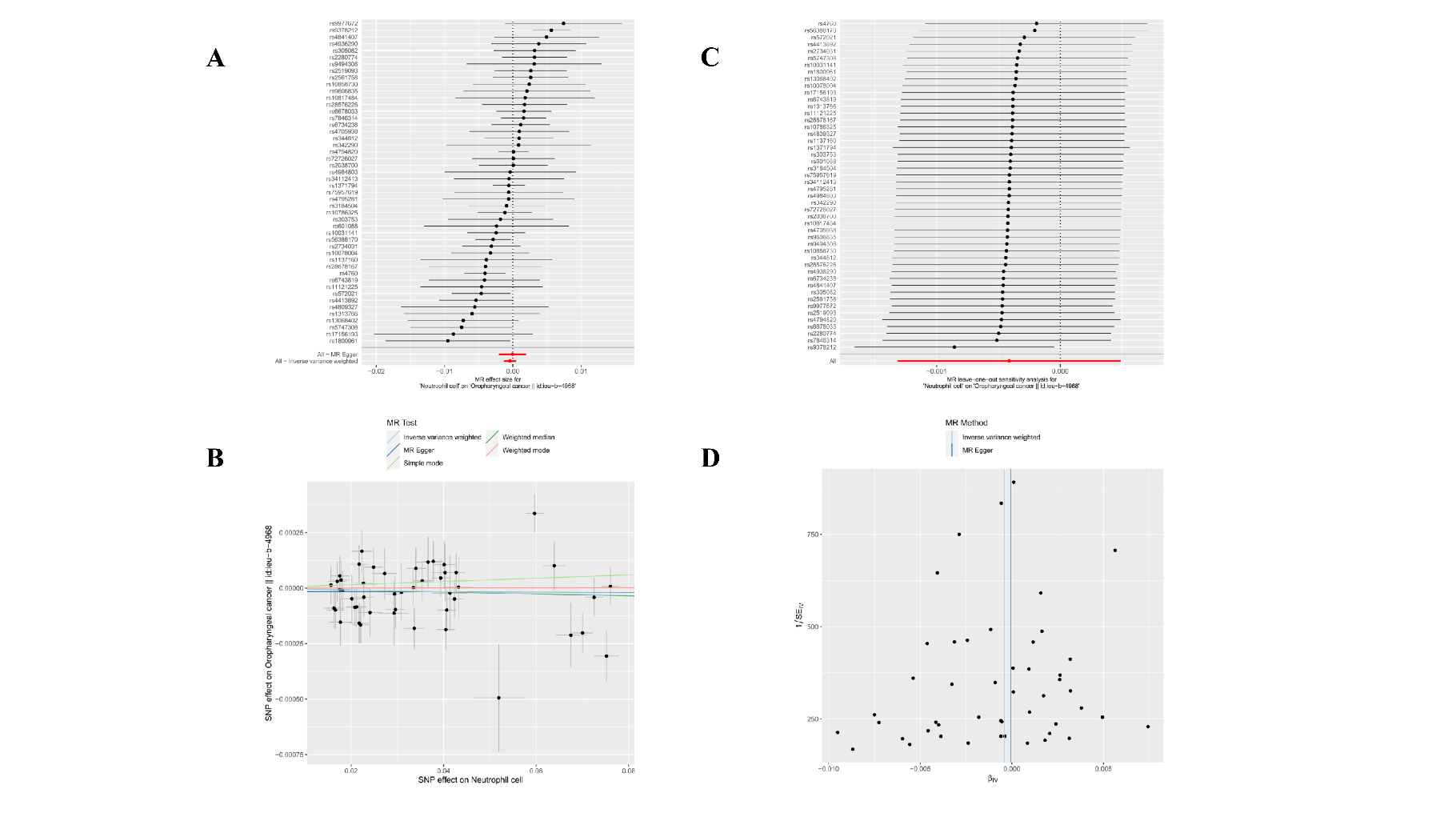
**

**Figure 8 Results and sensitivity analyses of the genetic correlation between Neutrophil cell and oropharyngeal cancer plotted in (A) forest plot; ( B ) scatterplot; (C) Leave-one-out sensitivity test; and (D) funnel plot.**

**
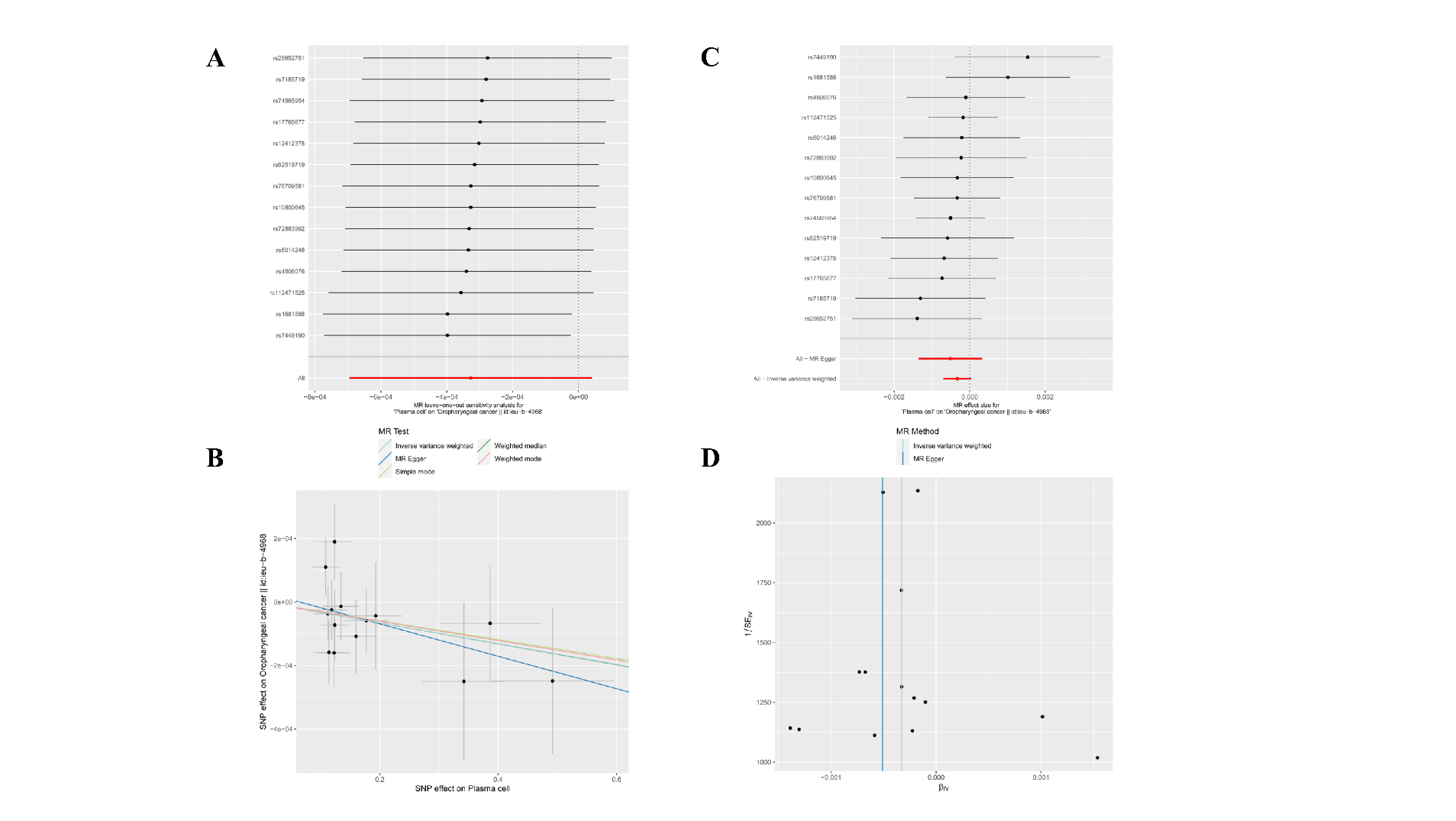
**

**Figure 9 Results and sensitivity analyses of the genetic correlation between Plasma cell and oropharyngeal cancer plotted in (A) forest plot; ( B ) scatterplot; (C) Leave-one-out sensitivity test; and (D) funnel plot.**

S7. Mendelian randomization analysis of 14 inflammatory factors and oropharyngeal cancer.

**Table 1** Results of Mendelian randomization analysis

**Table 2** Sensitivity analysis of the Mendelian randomization analysis results

**Figure 1-14** Results and sensitivity analyses plots of the genetic correlation between 14 inflammatory factors and oropharyngeal cancer

**Table 1 Results of Mendelian randomization analysis**

| **Exposure Outcome** | **Method** | **Oropharyngeal cancer** | | |
| --- | --- | --- | --- | --- |
|  |  | **SNP (n)** | **OR** | ***P* Value** |
|  | **MR Egger** | 11 | 1.000 | 0.798 |
|  | **Weighted median** | 11 | 1.000 | 0.794 |
| **IL-4** | **IVW** | 11 | 1.000 | 0.882 |
|  | **Simple mode** | 11 | 1.000 | 0.825 |
|  | **Weighted mode** | 11 | 1.000 | 0.819 |
|  | **MR Egger** | 10 | 1.000 | 0.898 |
|  | **Weighted median** | 10 | 1.000 | 0.931 |
| **IFN-**γ | **IVW** | 10 | 1.000 | 0.793 |
|  | **Simple mode** | 10 | 1.000 | 0.944 |
|  | **Weighted mode** | 10 | 1.000 | 0.983 |
|  | **MR Egger** | 9 | 0.999 | 0.129 |
|  | **Weighted median** | 9 | 1.000 | 0.473 |
| **IL-1α** | **IVW** | 9 | 1.000 | 0.738 |
|  | **Simple mode** | 9 | 1.000 | 0.326 |
|  | **Weighted mode** | 9 | 1.000 | 0.329 |
|  | **MR Egger** | 8 | 1.001 | 0.329 |
|  | **Weighted median** | 8 | 1.000 | 0.234 |
| **IL-1**β | **IVW** | 8 | 1.000 | 0.342 |
|  | **Simple mode** | 8 | 1.000 | 0.390 |
|  | **Weighted mode** | 8 | 1.000 | 0.318 |
|  | **MR Egger** | 11 | 0.999 | 0.215 |
|  | **Weighted median** | 11 | 0.999 | 0.149 |
| **IL-6** | **IVW** | 11 | 1.000 | 0.202 |
|  | **Simple mode** | 11 | 0.999 | 0.176 |
|  | **Weighted mode** | 11 | 0.999 | 0.200 |
|  | **MR Egger** | 9 | 1.000 | 0.633 |
|  | **Weighted median** | 9 | 1.000 | 0.520 |
| **IL-8** | **IVW** | 9 | 1.000 | 0.968 |
|  | **Simple mode** | 9 | 1.000 | 0.807 |
|  | **Weighted mode** | 9 | 1.000 | 0.511 |
|  | **MR Egger** | 15 | 0.998 | 0.548 |
|  | **Weighted median** | 15 | 1.000 | 0.616 |
| **IL-10** | **IVW** | 15 | 1.000 | 0.272 |
|  | **Simple mode** | 15 | 1.000 | 0.716 |
|  | **Weighted mode** | 15 | 1.000 | 0.774 |
|  | **MR Egger** | 15 | 0.999 | 0.281 |
|  | **Weighted median** | 15 | 1.000 | 0.620 |
| **IL-12** | **IVW** | 15 | 1.000 | 0.760 |
|  | **Simple mode** | 15 | 1.000 | 0.530 |
|  | **Weighted mode** | 15 | 1.000 | 0.721 |
|  | **MR Egger** | 11 | 0.999 | 0.258 |
|  | **Weighted median** | 11 | 1.000 | 0.622 |
| **IL-17** | **IVW** | 11 | 1.000 | 0.830 |
|  | **Simple mode** | 11 | 1.000 | 0.697 |
|  | **Weighted mode** | 11 | 1.000 | 0.714 |
|  | **MR Egger** | 14 | 1.000 | 0.535 |
|  | **Weighted median** | 14 | 1.000 | 0.868 |
| **IL-18** | **IVW** | 14 | 1.000 | 0.848 |
|  | **Simple mode** | 14 | 1.000 | 0.730 |
|  | **Weighted mode** | 14 | 1.000 | 0.954 |
|  | **MR Egger** | 16 | 1.001 | 0.265 |
|  | **Weighted median** | 16 | 1.001 | 0.087 |
| **MCP-1** | **IVW** | 16 | 1.000 | 0.567 |
|  | **Simple mode** | 16 | 1.001 | 0.391 |
|  | **Weighted mode** | 16 | 1.001 | 0.158 |
|  | **MR Egger** | 10 | 0.999 | 0.186 |
|  | **Weighted median** | 10 | 1.000 | 0.599 |
| **MIP1α** | **IVW** | 10 | 1.000 | 0.608 |
|  | **Simple mode** | 10 | 1.000 | 0.679 |
|  | **Weighted mode** | 10 | 1.000 | 0.550 |
|  | **MR Egger** | 10 | 0.999 | 0.318 |
|  | **Weighted median** | 10 | 1.000 | 0.364 |
| **RANTES** | **IVW** | 10 | 1.000 | 0.856 |
|  | **Simple mode** | 10 | 1.000 | 0.444 |
|  | **Weighted mode** | 10 | 1.000 | 0.490 |
|  | **MR Egger** | 4 | 1.001 | 0.424 |
|  | **Weighted median** | 4 | 1.000 | 0.754 |
| **TNF-α** | **IVW** | 4 | 1.000 | 0.717 |
|  | **Simple mode** | 4 | 1.000 | 0.548 |
|  | **Weighted mode** | 4 | 1.000 | 0.733 |

**Table2 Sensitivity analysis of the Mendelian randomization analysis results**

| **Exposure Outcome** | **Method** | | **Oropharyngeal cancer** |
| --- | --- | --- | --- |
|  | **IVW (heterogeneity)** | ***p* value** | 0.966 |
|  |  | **Q** | 3.519 |
| **IL-4** | **MR Egger (heterogeneity)** | ***p* value** | 0.949 |
|  |  | **Q** | 3.347 |
|  | **MR Egger (pleiotropy)** | ***p* value** | 0.688 |
|  |  | **intercept** | 0.000 |
|  | **IVW (heterogeneity)** | ***p* value** | 0.909 |
|  |  | **Q** | 4.040 |
| **IFN-**γ | **MR Egger (heterogeneity)** | ***p* value** | 0.853 |
|  |  | **Q** | 4.040 |
|  | **MR Egger (pleiotropy)** | ***p* value** | 0.992 |
|  |  | **intercept** | 0.000 |
|  | **IVW (heterogeneity)** | ***p* value** | 0.808 |
|  |  | **Q** | 4.510 |
| **IL-1α** | **MR Egger (heterogeneity)** | ***p* value** | 0.977 |
|  |  | **Q** | 1.636 |
|  | **MR Egger (pleiotropy)** | ***p* value** | 0.134 |
|  |  | **intercept** | 0.000 |
|  | **IVW (heterogeneity)** | ***p* value** | 0.983 |
|  |  | **Q** | 1.484 |
| **IL-1**β | **MR Egger (heterogeneity)** | ***p* value** | 0.987 |
|  |  | **Q** | 0.959 |
|  | **MR Egger (pleiotropy)** | ***p* value** | 0.496 |
|  |  | **intercept** | 0.000 |
|  | **IVW (heterogeneity)** | ***p* value** | 0.892 |
|  |  | **Q** | 4.992 |
| **IL-6** | **MR Egger (heterogeneity)** | ***p* value** | 0.891 |
|  |  | **Q** | 4.300 |
|  | **MR Egger (pleiotropy)** | ***p* value** | 0.427 |
|  |  | **intercept** | 0.000 |
|  | **IVW (heterogeneity)** | ***p* value** | 0.427 |
|  |  | **Q** | 8.065 |
| **IL-8** | **MR Egger (heterogeneity)** | ***p* value** | 0.361 |
|  |  | **Q** | 7.690 |
|  | **MR Egger (pleiotropy)** | ***p* value** | 0.578 |
|  |  | **intercept** | 0.000 |
|  | **IVW (heterogeneity)** | ***p* value** | 0.498 |
|  |  | **Q** | 13.364 |
| **IL-10** | **MR Egger (heterogeneity)** | ***p* value** | 0.462 |
|  |  | **Q** | 12.811 |
|  | **MR Egger (pleiotropy)** | ***p* value** | 0.470 |
|  |  | **intercept** | 0.000 |
|  | **IVW (heterogeneity)** | ***p* value** | 0.454 |
|  |  | **Q** | 13.946 |
| **IL-12** | **MR Egger (heterogeneity)** | ***p* value** | 0.493 |
|  |  | **Q** | 12.426 |
|  | **MR Egger (pleiotropy)** | ***p* value** | 0.239 |
|  |  | **intercept** | 0.000 |
|  | **IVW (heterogeneity)** | ***p* value** | 0.401 |
|  |  | **Q** | 10.462 |
| **IL-17** | **MR Egger (heterogeneity)** | ***p* value** | 0.438 |
|  |  | **Q** | 8.993 |
|  | **MR Egger (pleiotropy)** | ***p* value** | 0.256 |
|  |  | **intercept** | 0.000 |
|  | **IVW (heterogeneity)** | ***p* value** | 0.496 |
|  |  | **Q** | 12.389 |
| **IL-18** | **MR Egger (heterogeneity)** | ***p* value** | 0.461 |
|  |  | **Q** | 11.813 |
|  | **MR Egger (pleiotropy)** | ***p* value** | 0.463 |
|  |  | **intercept** | 0.000 |
|  | **IVW (heterogeneity)** | ***p* value** | 0.491 |
|  |  | **Q** | 14.455 |
| **MCP-1** | **MR Egger (heterogeneity)** | ***p* value** | 0.493 |
|  |  | **Q** | 13.426 |
|  | **MR Egger (pleiotropy)** | ***p* value** | 0.328 |
|  |  | **intercept** | (0.000) |
|  | **IVW (heterogeneity)** | ***p* value** | 0.741 |
|  |  | **Q** | 5.992 |
| **MIP1α** | **MR Egger (heterogeneity)** | ***p* value** | 0.843 |
|  |  | **Q** | 4.158 |
|  | **MR Egger (pleiotropy)** | ***p* value** | 0.213 |
|  |  | **intercept** | 0.000 |
|  | **IVW (heterogeneity)** | ***p* value** | 0.057 |
|  |  | **Q** | 16.501 |
| **RANTES** | **MR Egger (heterogeneity)** | ***p* value** | 0.084 |
|  |  | **Q** | 13.931 |
|  | **MR Egger (pleiotropy)** | ***p* value** | 0.259 |
|  |  | **intercept** | 0.000 |
|  | **IVW (heterogeneity)** | ***p* value** | 0.493 |
|  |  | **Q** | 2.404 |
| **TNF-α** | **MR Egger (heterogeneity)** | ***p* value** | 0.658 |
|  |  | **Q** | 0.838 |
|  | **MR Egger (pleiotropy)** | ***p* value** | 0.337 |
|  |  | **intercept** | 0.000 |


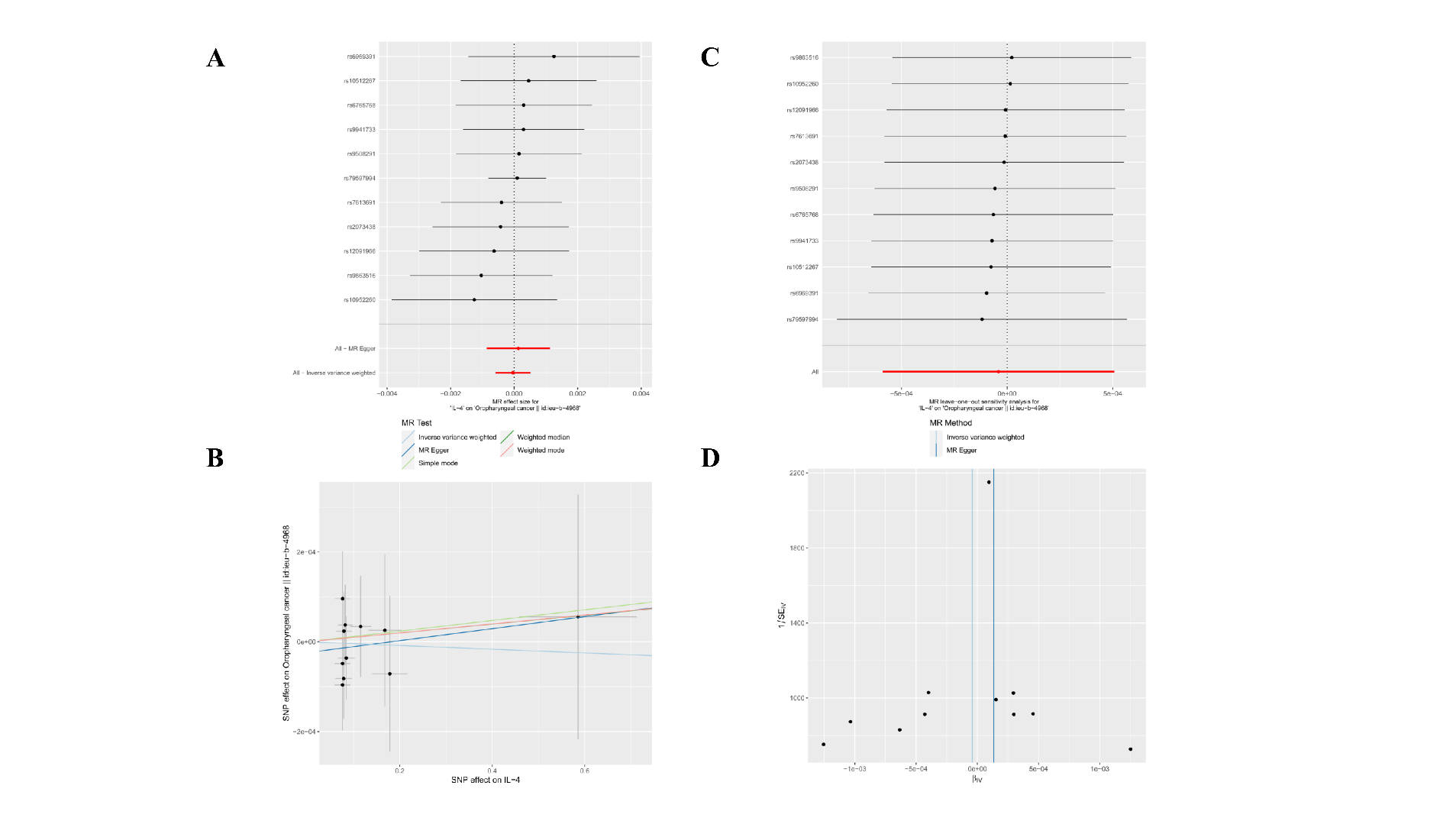


**Figure 1 Results and sensitivity analyses of the genetic correlation between** **IL-4 and oropharyngeal cancer plotted in (A) forest plot; ( B ) scatterplot; (C) Leave-one-out sensitivity test; and (D) funnel plot.**


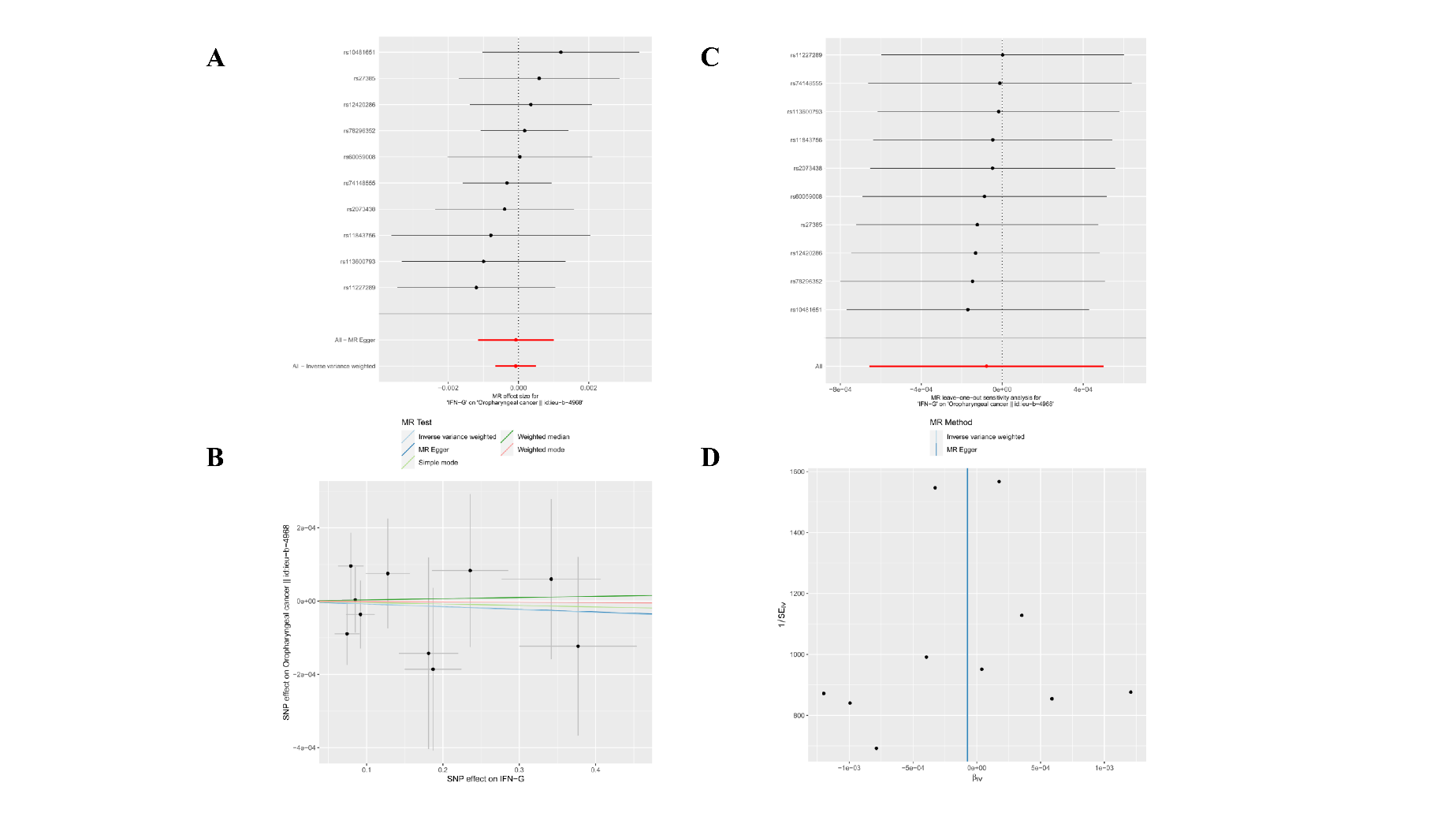


**Figure 2 Results and sensitivity analyses of the genetic correlation between** **IFN-**γ **and oropharyngeal cancer plotted in (A) forest plot; ( B ) scatterplot; (C) Leave-one-out sensitivity test; and (D) funnel plot.**


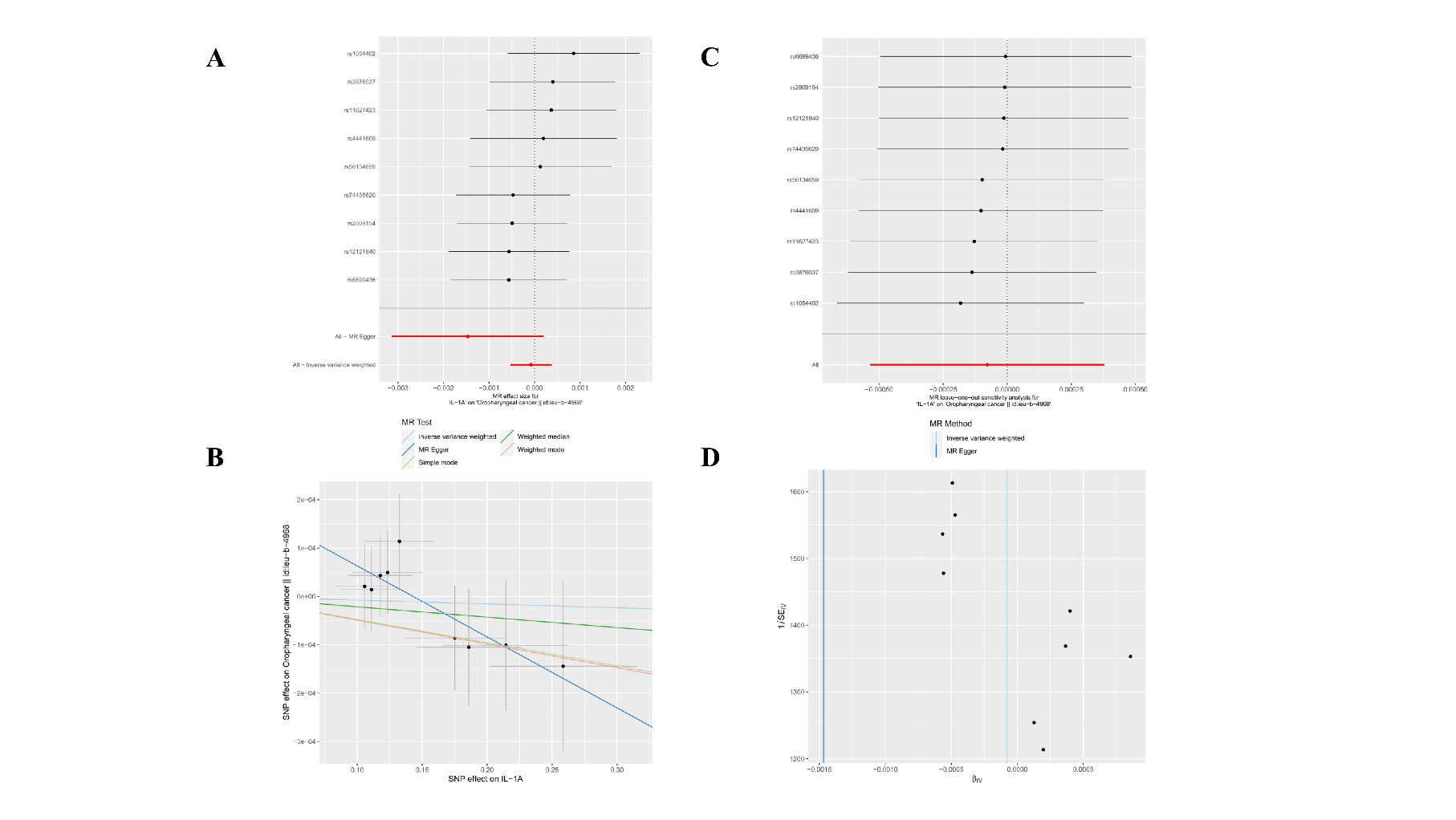


**Figure 3 Results and sensitivity analyses of the genetic correlation between** **IL-1α and oropharyngeal cancer plotted in (A) forest plot; ( B ) scatterplot; (C) Leave-one-out sensitivity test; and (D) funnel plot.**


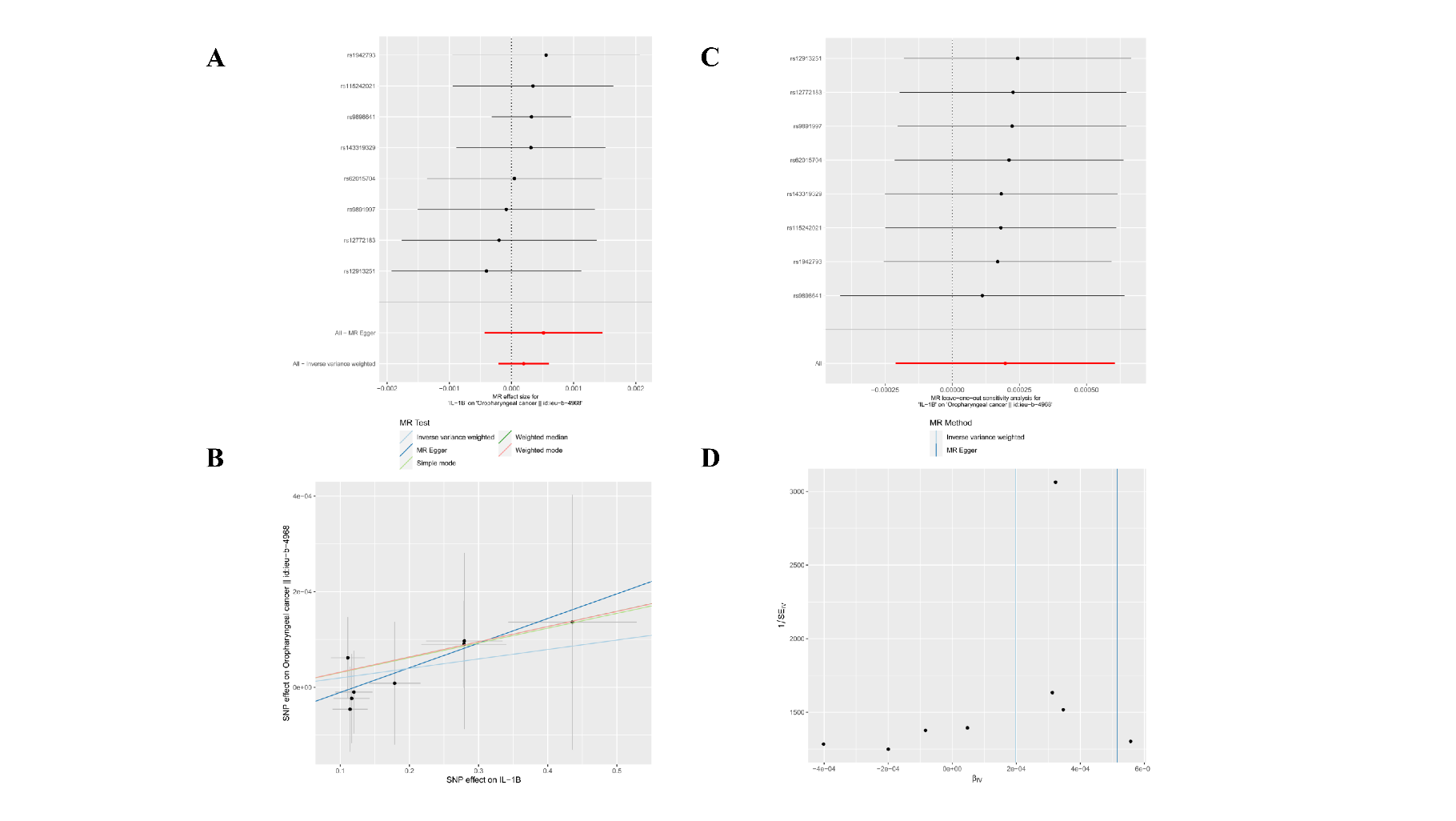


**Figure 4 Results and sensitivity analyses of the genetic correlation between** **IL-1β and oropharyngeal cancer plotted in (A) forest plot; ( B ) scatterplot; (C) Leave-one-out sensitivity test; and (D) funnel plot.**


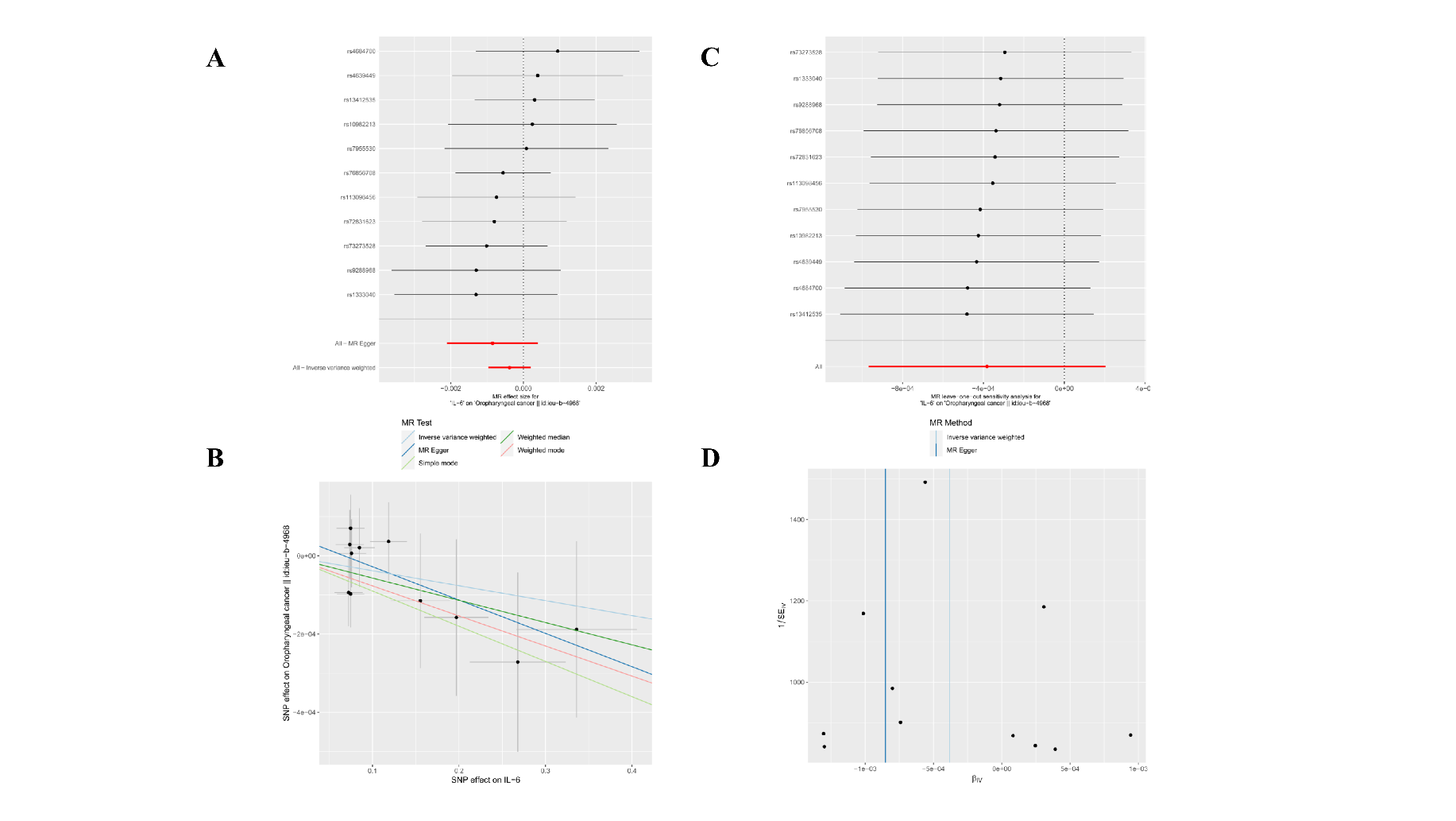


**Figure 5 Results and sensitivity analyses of the genetic correlation between** **IL-6 and oropharyngeal cancer plotted in (A) forest plot; ( B ) scatterplot; (C) Leave-one-out sensitivity test; and (D) funnel plot.**


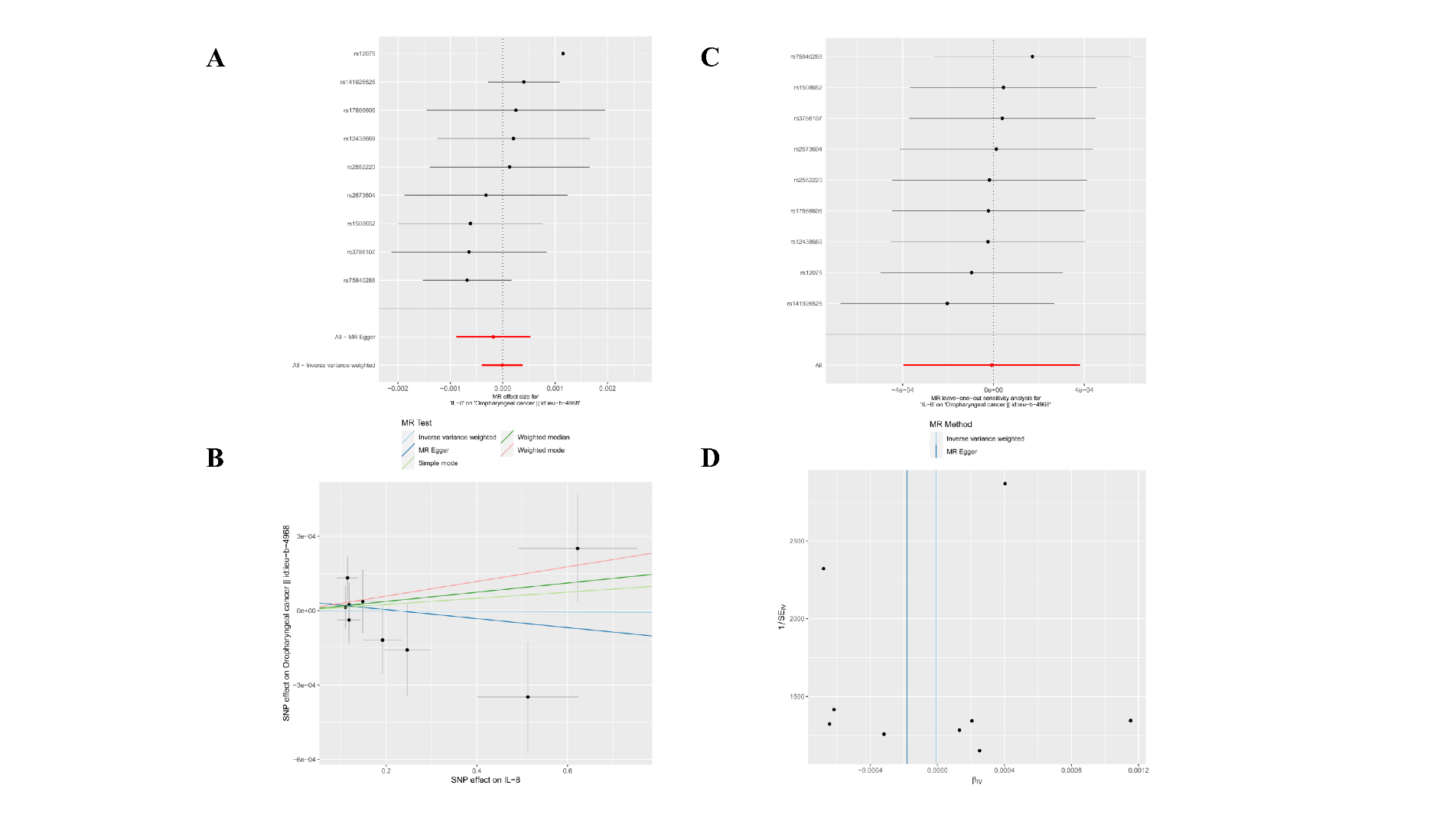


**Figure 6 Results and sensitivity analyses of the genetic correlation between** **IL-8 and oropharyngeal cancer plotted in (A) forest plot; ( B ) scatterplot; (C) Leave-one-out sensitivity test; and (D) funnel plot.**


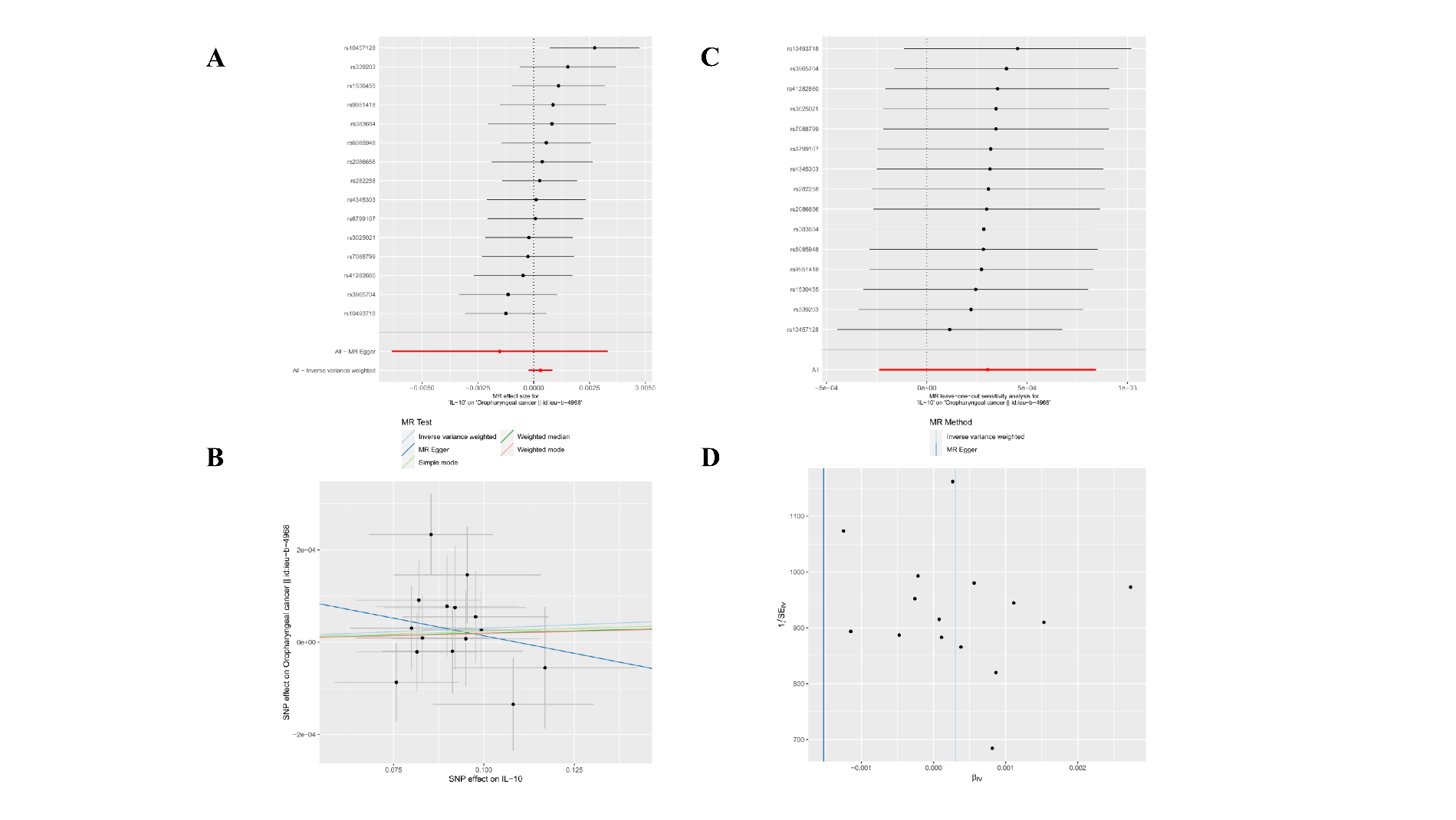


**Figure 7 Results and sensitivity analyses of the genetic correlation between** **IL-10 and oropharyngeal cancer plotted in (A) forest plot; ( B ) scatterplot; (C) Leave-one-out sensitivity test; and (D) funnel plot.**


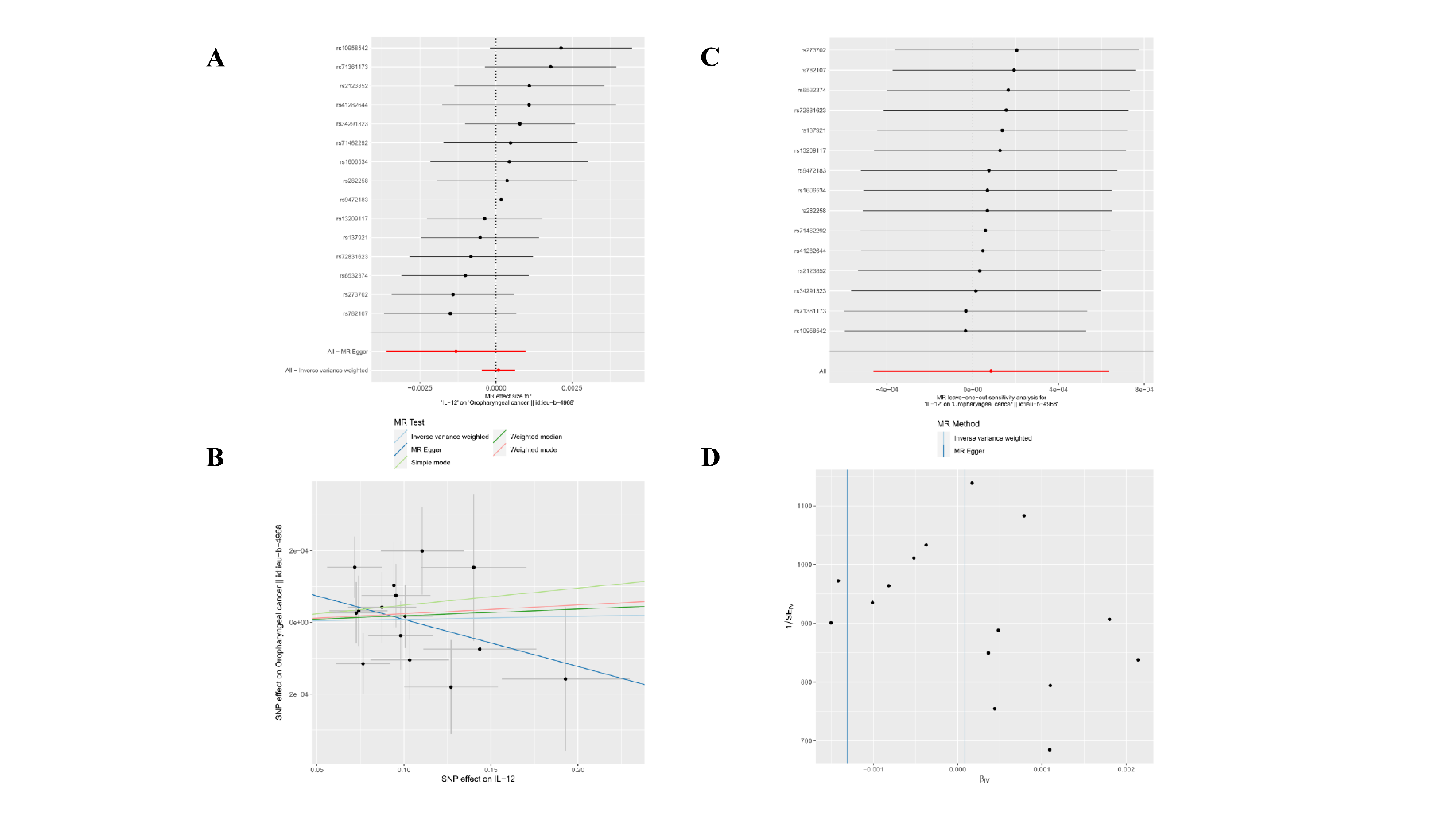


**Figure 8 Results and sensitivity analyses of the genetic correlation between** **IL-12 and oropharyngeal cancer plotted in (A) forest plot; ( B ) scatterplot; (C) Leave-one-out sensitivity test; and (D) funnel plot.**


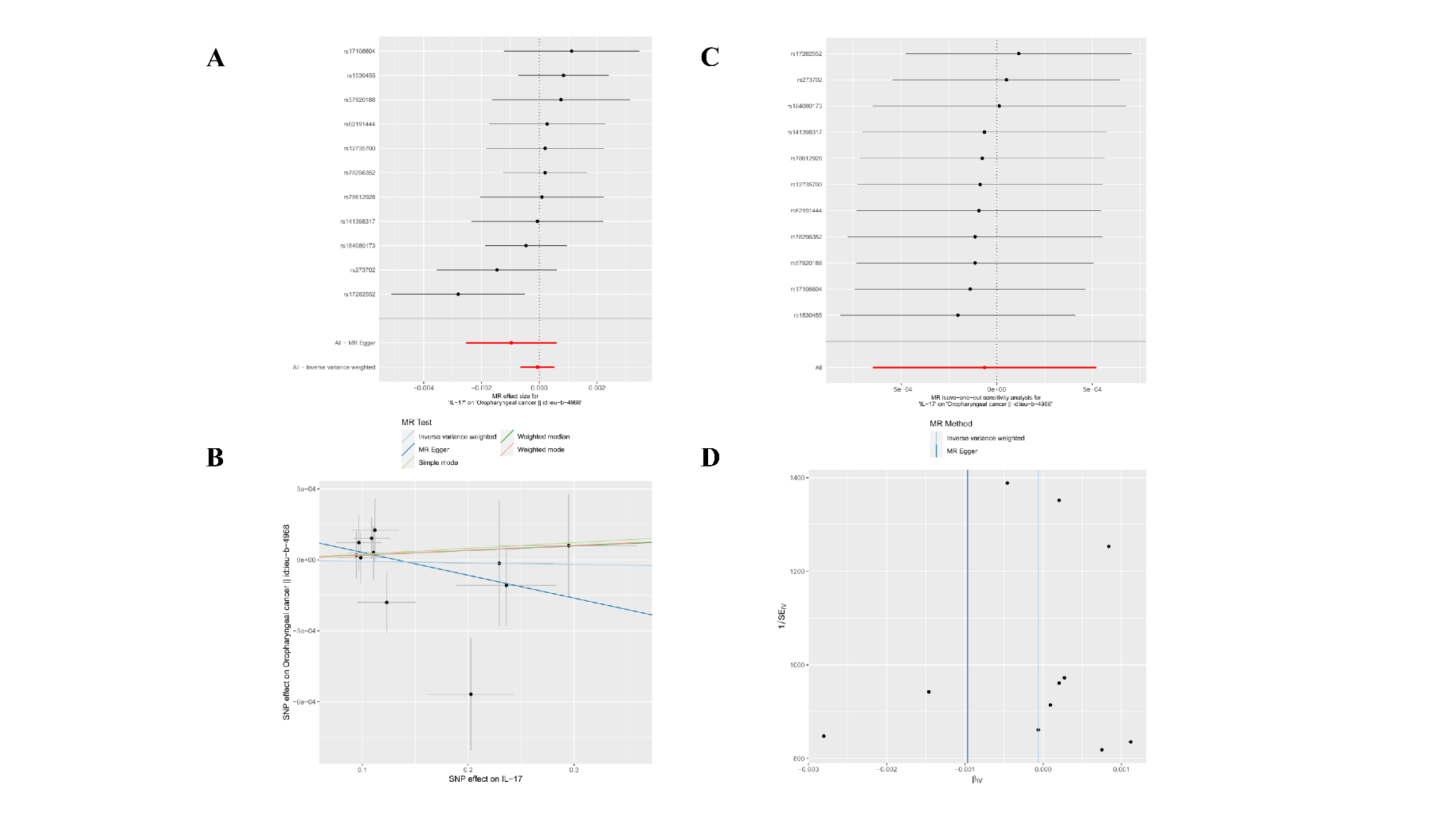


**Figure 9 Results and sensitivity analyses of the genetic correlation between** **IL-17 and oropharyngeal cancer plotted in (A) forest plot; ( B ) scatterplot; (C) Leave-one-out sensitivity test; and (D) funnel plot.**


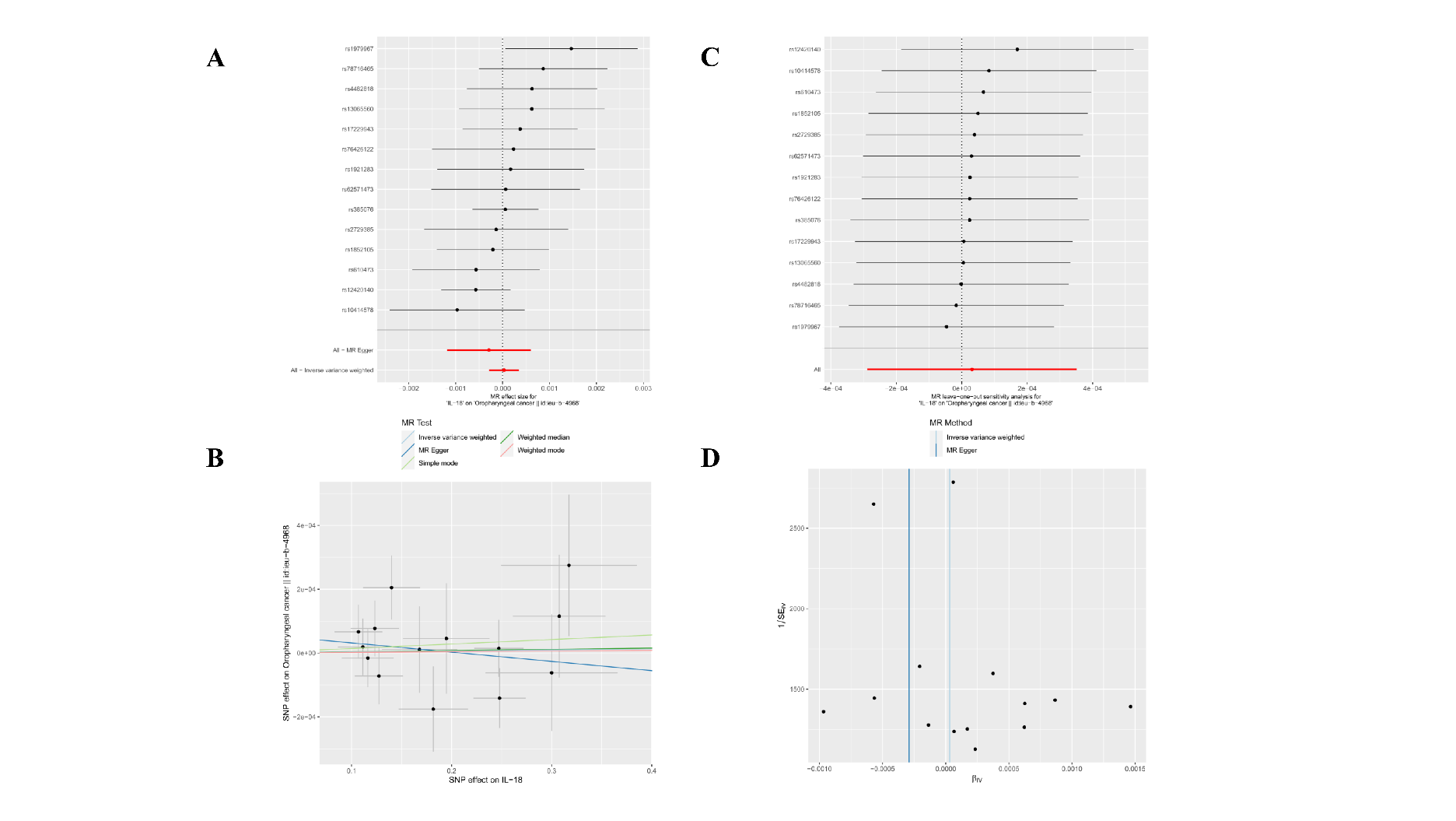


**Figure 10 Results and sensitivity analyses of the genetic correlation between IL-18** **and oropharyngeal cancer plotted in (A) forest plot; ( B ) scatterplot; (C) Leave-one-out sensitivity test; and (D) funnel plot.**


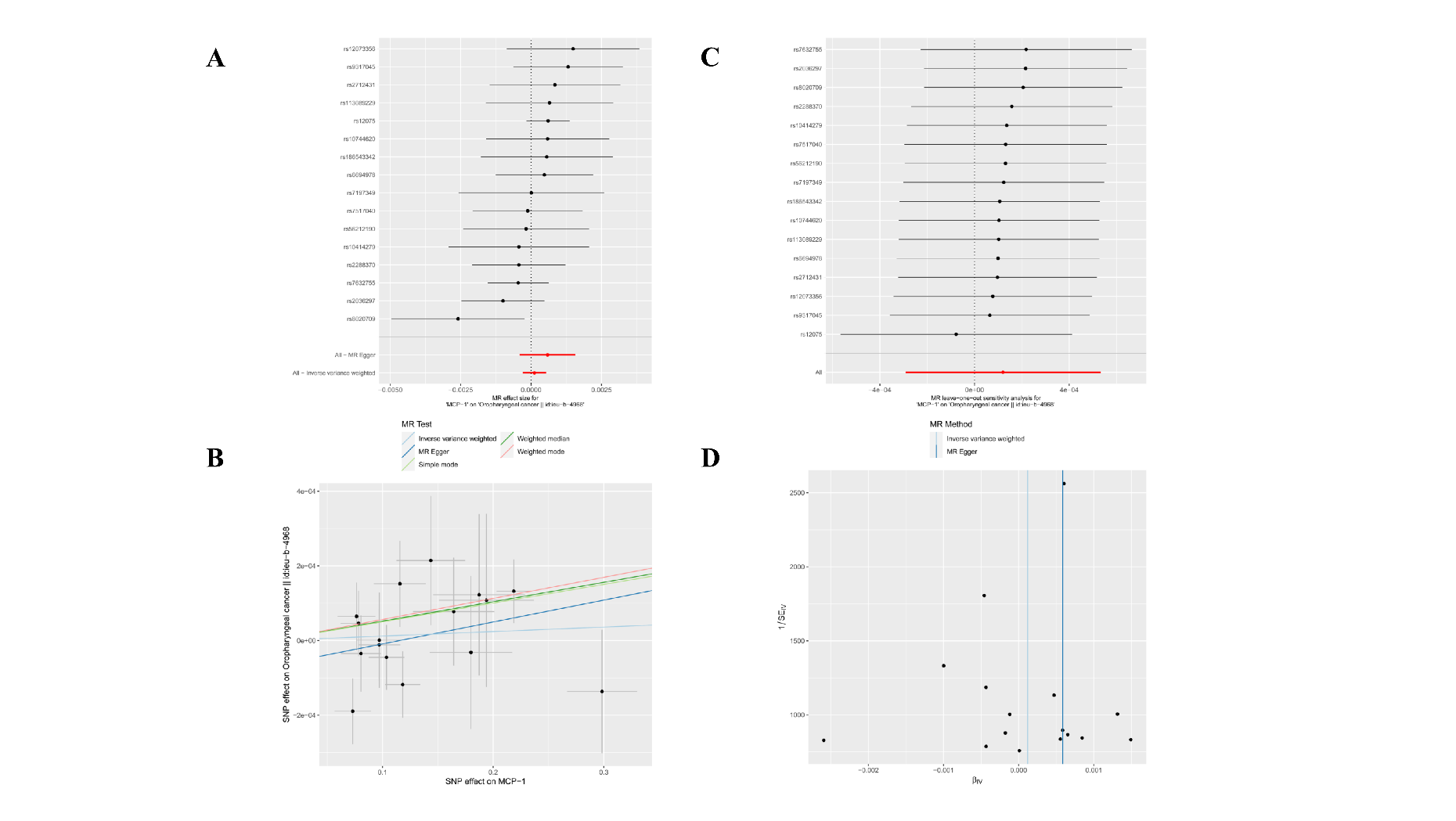


**Figure 11 Results and sensitivity analyses of the genetic correlation between** **MCP-1 and oropharyngeal cancer plotted in (A) forest plot; ( B ) scatterplot; (C) Leave-one-out sensitivity test; and (D) funnel plot.**


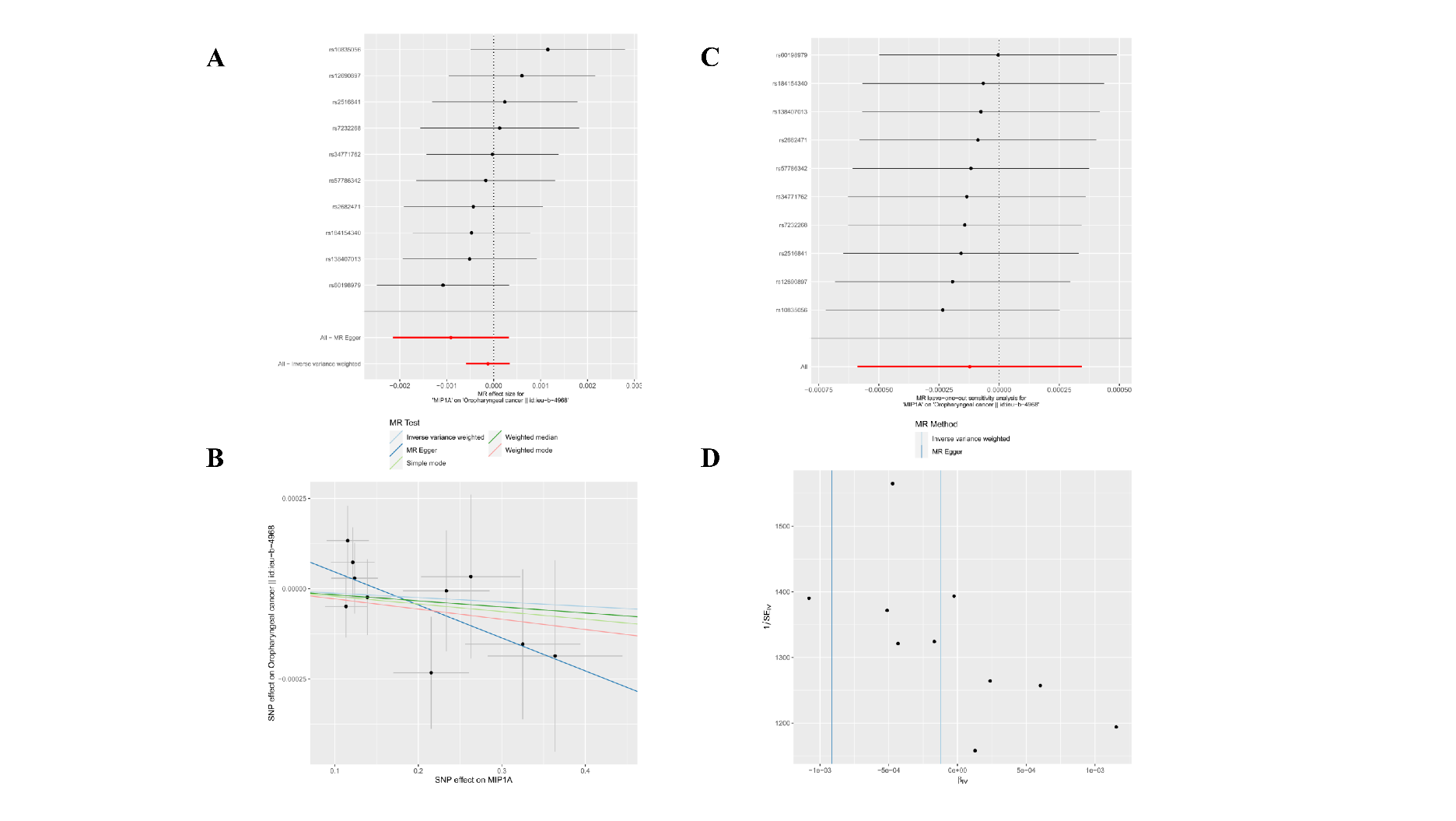


**Figure 12 Results and sensitivity analyses of the genetic correlation between** **MIP1α and oropharyngeal cancer plotted in (A) forest plot; ( B ) scatterplot; (C) Leave-one-out sensitivity test; and (D) funnel plot.**


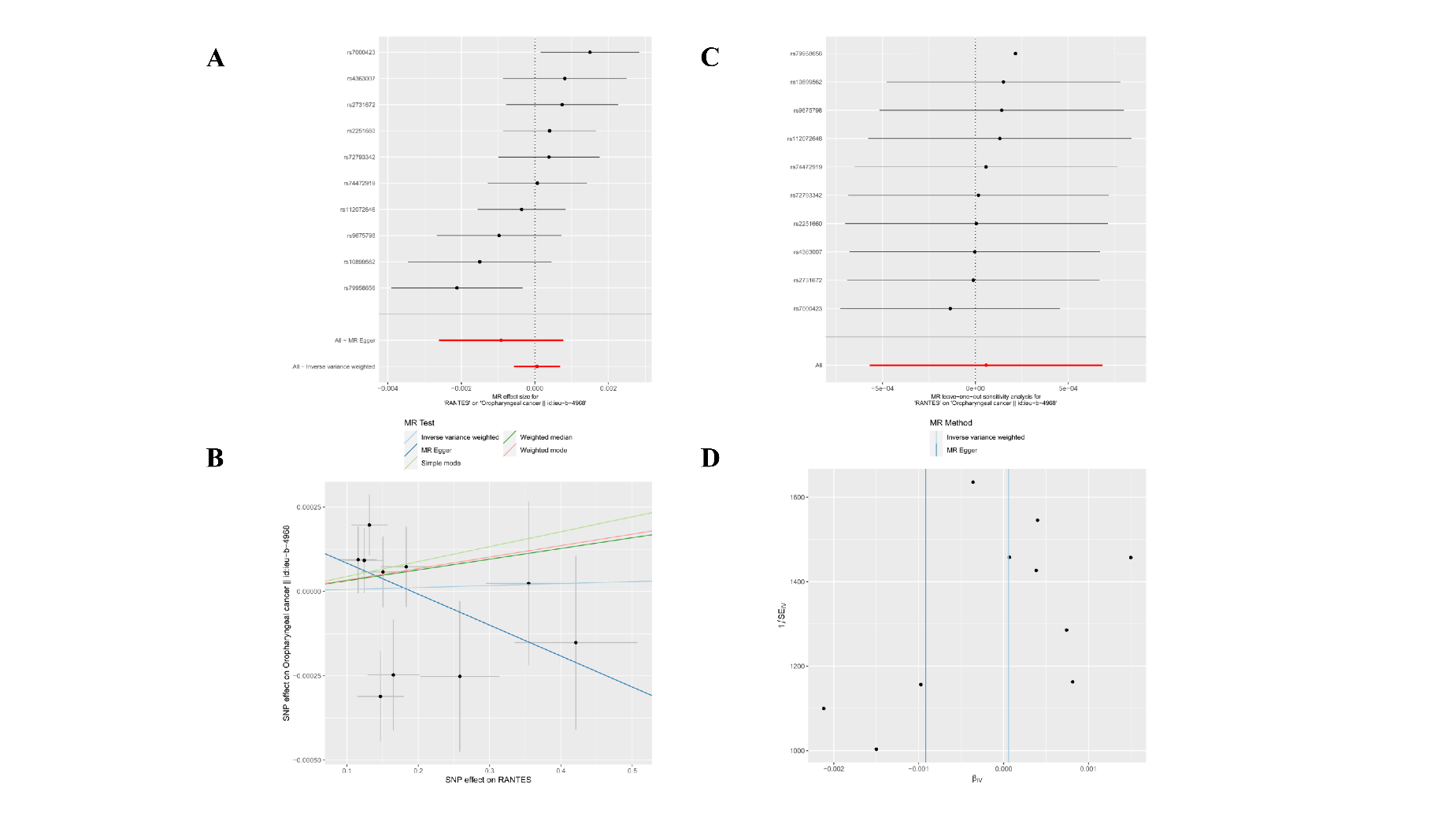


**Figure 13 Results and sensitivity analyses of the genetic correlation between** **RANTES and oropharyngeal cancer plotted in (A) forest plot; ( B ) scatterplot; (C) Leave-one-out sensitivity test; and (D) funnel plot.**


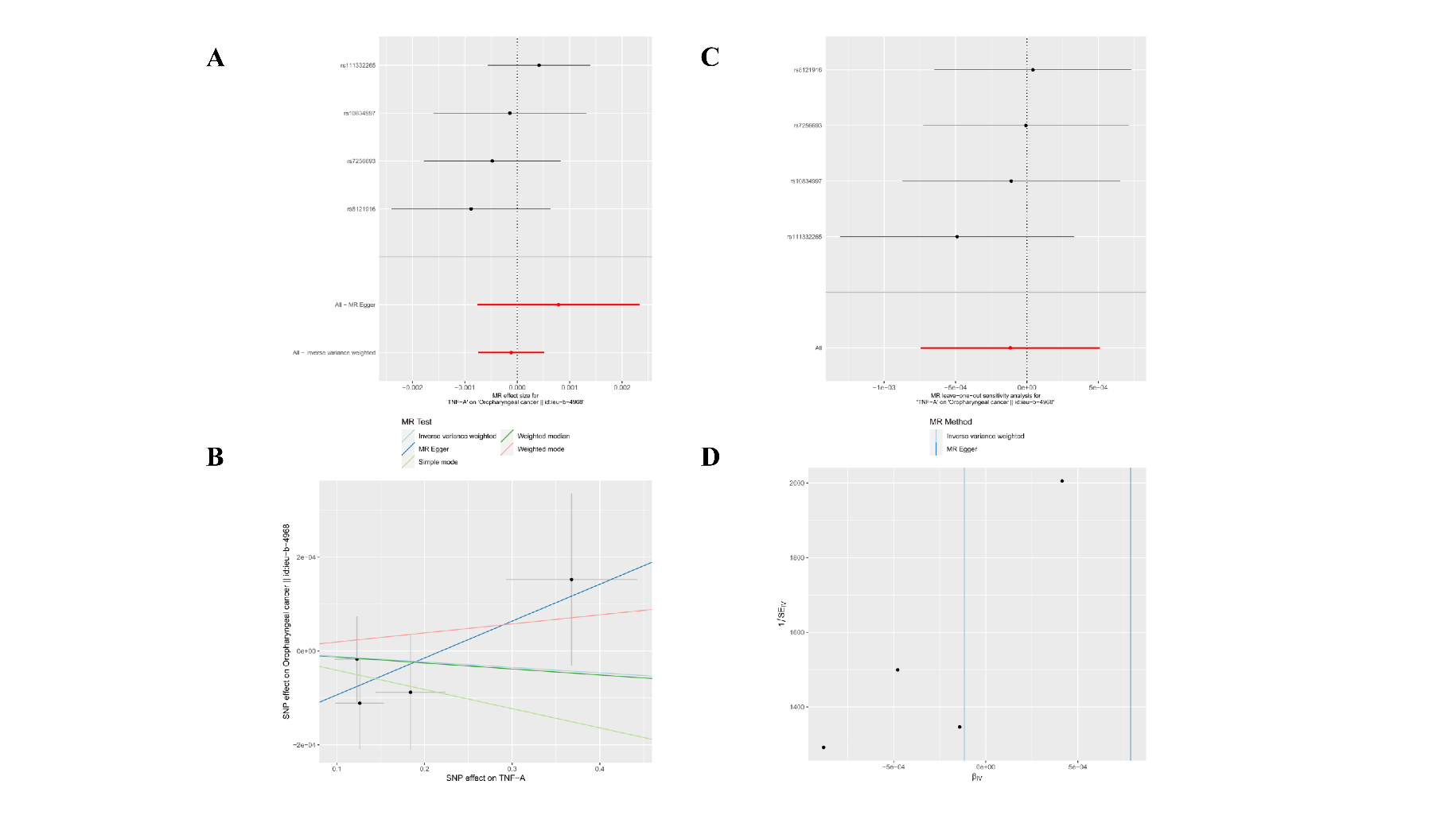


**Figure 14 Results and sensitivity analyses of the genetic correlation between** **TNF-α and oropharyngeal cancer plotted in (A) forest plot; ( B ) scatterplot; (C) Leave-one-out sensitivity test; and (D) funnel plot.**

S8. Mendelian randomization analysis of 20 cancers and periodontitis.

**Table 1** Results of Mendelian randomization analysis

**Table 2** Sensitivity analysis of the Mendelian randomization analysis results

**Figure 1-20** Results and sensitivity analyses plots of the genetic correlation between 20 cancers and periodontitis

**Table 1 Results of Mendelian randomization analysis**

| **Exposure Outcome** | **Method** | **Periodontitis** | | |
| --- | --- | --- | --- | --- |
|  |  | **SNP (n)** | **OR** | ***P* Value** |
|  | **MR Egger** | 116 | 0.982 | 0.111 |
|  | **Weighted median** | 116 | 0.987 | 0.102 |
| **Basal cell carcinoma** | **IVW** | 116 | 0.987 | 0.020 |
|  | **Simple mode** | 116 | 1.000 | 0.989 |
|  | **Weighted mode** | 116 | 0.991 | 0.419 |
|  | **MR Egger** | 27 | 5.852 | 0.502 |
|  | **Weighted median** | 27 | 14.713 | 0.135 |
| **Bladder cancer** | **IVW** | 27 | 6.299 | 0.140 |
|  | **Simple mode** | 27 | 51.329 | 0.312 |
|  | **Weighted mode** | 27 | 39.859 | 0.303 |
|  | **MR Egger** | 30 | 0.349 | 0.813 |
|  | **Weighted median** | 30 | 0.220 | 0.517 |
| **Brain cancer** | **IVW** | 30 | 1.505 | 0.809 |
|  | **Simple mode** | 30 | 0.004 | 0.238 |
|  | **Weighted mode** | 30 | 0.007 | 0.230 |
|  | **MR Egger** | 49 | 0.940 | 0.940 |
|  | **Weighted median** | 49 | 1.419 | 0.443 |
| **Breast cancer** | **IVW** | 49 | 1.445 | 0.250 |
|  | **Simple mode** | 49 | 3.934 | 0.177 |
|  | **Weighted mode** | 49 | 1.445 | 0.602 |
|  | **MR Egger** | 22 | 5.512 | 0.592 |
|  | **Weighted median** | 22 | 0.564 | 0.733 |
| **Cancer of urinary tract** | **IVW** | 22 | 0.389 | 0.465 |
|  | **Simple mode** | 22 | 0.095 | 0.508 |
|  | **Weighted mode** | 22 | 0.170 | 0.611 |
|  | **MR Egger** | 18 | 1.998 | 0.840 |
|  | **Weighted median** | 18 | 1.013 | 0.994 |
| **Cervical cancer** | **IVW** | 18 | 1.562 | 0.729 |
|  | **Simple mode** | 18 | 0.376 | 0.766 |
|  | **Weighted mode** | 18 | 0.349 | 0.723 |
|  | **MR Egger** | 37 | 1.190 | 0.909 |
|  | **Weighted median** | 37 | 1.905 | 0.376 |
| **Colorectal cancer** | **IVW** | 37 | 1.071 | 0.900 |
|  | **Simple mode** | 37 | 1.809 | 0.705 |
|  | **Weighted mode** | 37 | 2.995 | 0.391 |
|  | **MR Egger** | 47 | 0.990 | 0.621 |
|  | **Weighted median** | 47 | 0.984 | 0.111 |
| **Endometrial cancer** | **IVW** | 47 | 0.984 | 0.027 |
|  | **Simple mode** | 47 | 0.977 | 0.313 |
|  | **Weighted mode** | 47 | 0.983 | 0.388 |
|  | **MR Egger** | 10 | 1.006 | 0.873 |
|  | **Weighted median** | 10 | 0.992 | 0.594 |
| **Gastric cancer** | **IVW** | 10 | 0.988 | 0.282 |
|  | **Simple mode** | 10 | 0.997 | 0.904 |
|  | **Weighted mode** | 10 | 0.993 | 0.660 |
|  | **MR Egger** | 15 | 21.595 | 0.450 |
|  | **Weighted median** | 15 | 5.851 | 0.469 |
| **Head and neck cancer** | **IVW** | 15 | 14.420 | 0.153 |
|  | **Simple mode** | 15 | 1.311 | 0.951 |
|  | **Weighted mode** | 15 | 3.027 | 0.794 |
|  | **MR Egger** | 22 | 947102575.995 | 0.031 |
|  | **Weighted median** | 22 | 1798.884 | 0.052 |
| **Liver & bile duct** | **IVW** | 22 | 82.992 | 0.137 |
| **cancer** | **Simple mode** | 22 | 14012.049 | 0.242 |
|  | **Weighted mode** | 22 | 14012.049 | 0.244 |
|  | **MR Egger** | 24 | 2.733 | 0.678 |
|  | **Weighted median** | 24 | 13.197 | 0.068 |
| **Lung cancer** | **IVW** | 24 | 6.594 | 0.074 |
|  | **Simple mode** | 24 | 54.904 | 0.221 |
|  | **Weighted mode** | 24 | 48.483 | 0.233 |
|  | **MR Egger** | 136 | 0.820 | 0.303 |
|  | **Weighted median** | 136 | 0.782 | 0.095 |
| **Malignant non-melanoma skin cancer** | **IVW** | 136 | 0.834 | 0.073 |
|  | **Simple mode** | 136 | 0.627 | 0.132 |
|  | **Weighted mode** | 136 | 0.924 | 0.694 |
|  | **MR Egger** | 37 | 0.116 | 0.081 |
|  | **Weighted median** | 37 | 0.459 | 0.354 |
| **Melanoma skin cancer** | **IVW** | 37 | 0.664 | 0.498 |
|  | **Simple mode** | 37 | 0.079 | 0.184 |
|  | **Weighted mode** | 37 | 0.220 | 0.130 |
|  | **MR Egger** | 23 | 6.275 | 0.675 |
|  | **Weighted median** | 23 | 0.137 | 0.433 |
| **Oesophageal cancer** | **IVW** | 23 | 0.113 | 0.229 |
|  | **Simple mode** | 23 | 0.430 | 0.862 |
|  | **Weighted mode** | 23 | 0.633 | 0.924 |
|  | **MR Egger** | 27 | 0.101 | 0.224 |
|  | **Weighted median** | 27 | 0.178 | 0.098 |
| **Ovarian cancer** | **IVW** | 27 | 0.366 | 0.221 |
|  | **Simple mode** | 27 | 0.061 | 0.237 |
|  | **Weighted mode** | 27 | 0.100 | 0.251 |
|  | **MR Egger** | 5 | 1.001 | 0.947 |
|  | **Weighted median** | 5 | 1.010 | 0.191 |
| **Pancreatic cancer** | **IVW** | 5 | 1.007 | 0.254 |
|  | **Simple mode** | 5 | 1.000 | 0.972 |
|  | **Weighted mode** | 5 | 1.011 | 0.295 |
|  | **MR Egger** | 45 | 0.705 | 0.831 |
|  | **Weighted median** | 45 | 0.903 | 0.896 |
| **Prostate cancer** | **IVW** | 45 | 0.798 | 0.720 |
|  | **Simple mode** | 45 | 0.684 | 0.795 |
|  | **Weighted mode** | 45 | 1.686 | 0.664 |
|  | **MR Egger** | 61 | 31.125 | 0.436 |
|  | **Weighted median** | 61 | 1.596 | 0.881 |
| **Small intestine cancer** | **IVW** | 61 | 1.358 | 0.895 |
|  | **Simple mode** | 61 | 3.650 | 0.864 |
|  | **Weighted mode** | 61 | 4.712 | 0.853 |
|  | **MR Egger** | 47 | 0.115 | 0.567 |
|  | **Weighted median** | 47 | 0.061 | 0.203 |
| **Squamous cell carcinoma** | **IVW** | 47 | 0.658 | 0.810 |
|  | **Simple mode** | 47 | 0.019 | 0.396 |
|  | **Weighted mode** | 47 | 0.022 | 0.437 |

**Table2 Sensitivity analysis of the Mendelian randomization analysis results**

| **Exposure Outcome** | **Method** | | **Periodontitis** | |
| --- | --- | --- | --- | --- |
|  | **IVW (heterogeneity)** | ***p* value** | 0.011 |  |
|  |  | **Q** | 152.465 |  |
| **Basal cell carcinoma** | **MR Egger (heterogeneity)** | ***p* value** | 0.010 |  |
|  |  | **Q** | 152.084 |  |
|  | **MR Egger (pleiotropy)** | ***p* value** | 0.594 |  |
|  |  | **intercept** | 0.001 |  |
|  | **IVW (heterogeneity)** | ***p* value** | 0.521 |  |
|  |  | **Q** | 24.968 |  |
| **Bladder cancer** | **MR Egger (heterogeneity)** | ***p* value** | 0.464 |  |
|  |  | **Q** | 24.967 |  |
|  | **MR Egger (pleiotropy)** | ***p* value** | 0.974 |  |
|  |  | **intercept** | 0.000 |  |
|  | **IVW (heterogeneity)** | ***p* value** | 0.790 |  |
|  |  | **Q** | 22.709 |  |
| **Brain cancer** | **MR Egger (heterogeneity)** | ***p* value** | 0.754 |  |
|  |  | **Q** | 22.580 |  |
|  | **MR Egger (pleiotropy)** | ***p* value** | 0.723 |  |
|  |  | **intercept** | 0.001 |  |
|  | **IVW (heterogeneity)** | ***p* value** | 0.152 |  |
|  |  | **Q** | 58.043 |  |
| **Breast cancer** | **MR Egger (heterogeneity)** | ***p* value** | 0.137 |  |
|  |  | **Q** | 57.647 |  |
|  | **MR Egger (pleiotropy)** | ***p* value** | 0.572 |  |
|  |  | **intercept** | 0.001 |  |
|  | **IVW (heterogeneity)** | ***p* value** | 0.231 |  |
|  |  | **Q** | 25.380 |  |
| **Cancer of urinary tract** | **MR Egger (heterogeneity)** | ***p* value** | 0.228 |  |
|  |  | **Q** | 24.329 |  |
|  | **MR Egger (pleiotropy)** | ***p* value** | 0.364 |  |
|  |  | **intercept** | -0.004 |  |
|  | **IVW (heterogeneity)** | ***p* value** | 0.371 |  |
|  |  | **Q** | 18.293 |  |
| **Cervical cancer** | **MR Egger (heterogeneity)** | ***p* value** | 0.307 |  |
|  |  | **Q** | 18.285 |  |
|  | **MR Egger (pleiotropy)** | ***p* value** | 0.938 |  |
|  |  | **intercept** | 0.000 |  |
|  | **IVW (heterogeneity)** | ***p* value** | 0.085 |  |
|  |  | **Q** | 48.122 |  |
| **Colorectal cancer** | **MR Egger (heterogeneity)** | ***p* value** | 0.069 |  |
|  |  | **Q** | 48.115 |  |
|  | **MR Egger (pleiotropy)** | ***p* value** | 0.941 |  |
|  |  | **intercept** | 0.000 |  |
|  | **IVW (heterogeneity)** | ***p* value** | 0.652 |  |
|  |  | **Q** | 41.709 |  |
| **Endometrial cancer** | **MR Egger (heterogeneity)** | ***p* value** | 0.617 |  |
|  |  | **Q** | 41.588 |  |
|  | **MR Egger (pleiotropy)** | ***p* value** | 0.730 |  |
|  |  | **intercept** | -0.001 |  |
|  | **IVW (heterogeneity)** | ***p* value** | 0.684 |  |
|  |  | **Q** | 6.552 |  |
| **Gastric cancer** | **MR Egger (heterogeneity)** | ***p* value** | 0.619 |  |
|  |  | **Q** | 6.253 |  |
|  | **MR Egger (pleiotropy)** | ***p* value** | 0.600 |  |
|  |  | **intercept** | -0.003 |  |
|  | **IVW (heterogeneity)** | ***p* value** | 0.767 |  |
|  |  | **Q** | 9.936 |  |
| **Head and neck cancer** | **MR Egger (heterogeneity)** | ***p* value** | 0.700 |  |
|  |  | **Q** | 9.922 |  |
|  | **MR Egger (pleiotropy)** | ***p* value** | 0.909 |  |
|  |  | **intercept** | 0.000 |  |
|  | **IVW (heterogeneity)** | ***p* value** | 0.208 |  |
|  |  | **Q** | 25.953 |  |
| **Liver & bile duct cancer** | **MR Egger (heterogeneity)** | ***p* value** | 0.345 |  |
|  |  | **Q** | 21.925 |  |
|  | **MR Egger (pleiotropy)** | ***p* value** | 0.070 |  |
|  |  | **intercept** | -0.009 |  |
|  | **IVW (heterogeneity)** | ***p* value** | 0.143 |  |
|  |  | **Q** | 30.239 |  |
| **Lung cancer** | **MR Egger (heterogeneity)** | ***p* value** | 0.118 |  |
|  |  | **Q** | 30.006 |  |
|  | **MR Egger (pleiotropy)** | ***p* value** | 0.683 |  |
|  |  | **intercept** | 0.002 |  |
|  | **IVW (heterogeneity)** | ***p* value** | 0.036 |  |
|  |  | **Q** | 165.998 |  |
| **Malignant non-melanoma skin cancer** | **MR Egger (heterogeneity)** | ***p* value** | 0.032 |  |
|  |  | **Q** | 165.985 |  |
|  | **MR Egger (pleiotropy)** | ***p* value** | 0.918 |  |
|  |  | **intercept** | 0.000 |  |
|  | **IVW (heterogeneity)** | ***p* value** | 0.052 |  |
|  |  | **Q** | 50.766 |  |
| **Melanoma skin cancer** | **MR Egger (heterogeneity)** | ***p* value** | 0.084 |  |
|  |  | **Q** | 47.021 |  |
|  | **MR Egger (pleiotropy)** | ***p* value** | 0.104 |  |
|  |  | **intercept** | 0.004 |  |
|  | **IVW (heterogeneity)** | ***p* value** | 0.740 |  |
|  |  | **Q** | 17.422 |  |
| **Oesophageal cancer** | **MR Egger (heterogeneity)** | ***p* value** | 0.748 |  |
|  |  | **Q** | 16.371 |  |
|  | **MR Egger (pleiotropy)** | ***p* value** | 0.317 |  |
|  |  | **intercept** | -0.004 |  |
|  | **IVW (heterogeneity)** | ***p* value** | 0.074 |  |
|  |  | **Q** | 37.023 |  |
| **Ovarian cancer** | **MR Egger (heterogeneity)** | ***p* value** | 0.070 |  |
|  |  | **Q** | 36.134 |  |
|  | **MR Egger (pleiotropy)** | ***p* value** | 0.440 |  |
|  |  | **intercept** | 0.004 |  |
|  | **IVW (heterogeneity)** | ***p* value** | 0.736 |  |
|  |  | **Q** | 1.999 |  |
| **Pancreatic cancer** | **MR Egger (heterogeneity)** | ***p* value** | 0.597 |  |
|  |  | **Q** | 1.881 |  |
|  | **MR Egger (pleiotropy)** | ***p* value** | 0.754 |  |
|  |  | **intercept** | 0.003 |  |
|  | **IVW (heterogeneity)** | ***p* value** | 0.074 |  |
|  |  | **Q** | 58.228 |  |
| **Prostate cancer** | **MR Egger (heterogeneity)** | ***p* value** | 0.061 |  |
|  |  | **Q** | 58.219 |  |
|  | **MR Egger (pleiotropy)** | ***p* value** | 0.935 |  |
|  |  | **intercept** | 0.000 |  |
|  | **IVW (heterogeneity)** | ***p* value** | 0.147 |  |
|  |  | **Q** | 71.519 |  |
| **Small intestine cancer** | **MR Egger (heterogeneity)** | ***p* value** | 0.142 |  |
|  |  | **Q** | 70.672 |  |
|  | **MR Egger (pleiotropy)** | ***p* value** | 0.404 |  |
|  |  | **intercept** | -0.002 |  |
|  | **IVW (heterogeneity)** | ***p* value** | 0.092 |  |
|  |  | **Q** | 59.148 |  |
| **Squamous cell carcinoma** | **MR Egger (heterogeneity)** | ***p* value** | 0.081 |  |
|  |  | **Q** | 58.787 |  |
|  | **MR Egger (pleiotropy)** | ***p* value** | 0.602 |  |
|  |  | **intercept** | 0.002 |  |


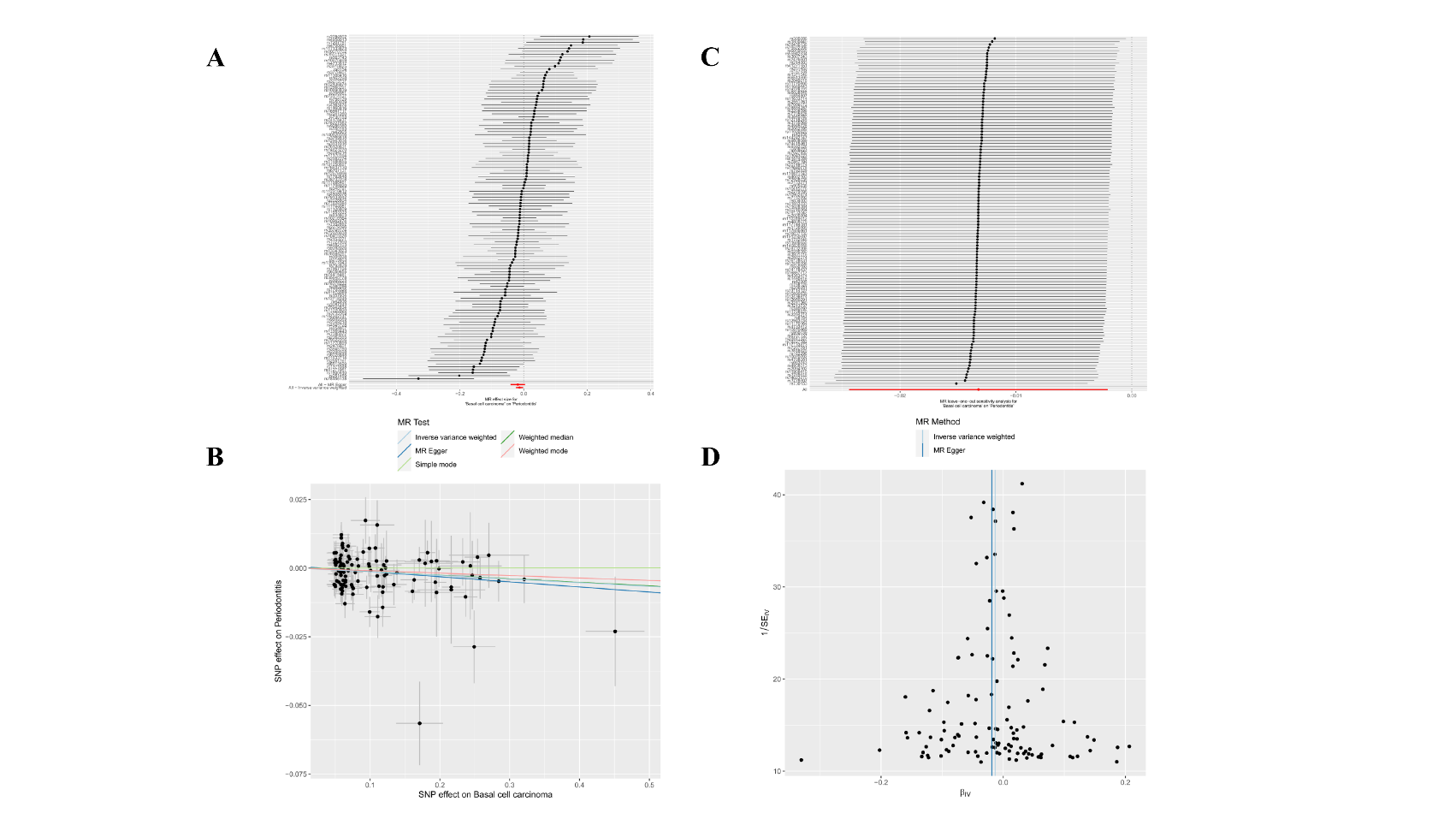


**Figure 1 Results and sensitivity analyses of the genetic correlation between basal cell carcinoma and periodontitis plotted in (A) forest plot; ( B ) scatterplot; (C) Leave-one-out sensitivity test; and (D) funnel plot.**

**
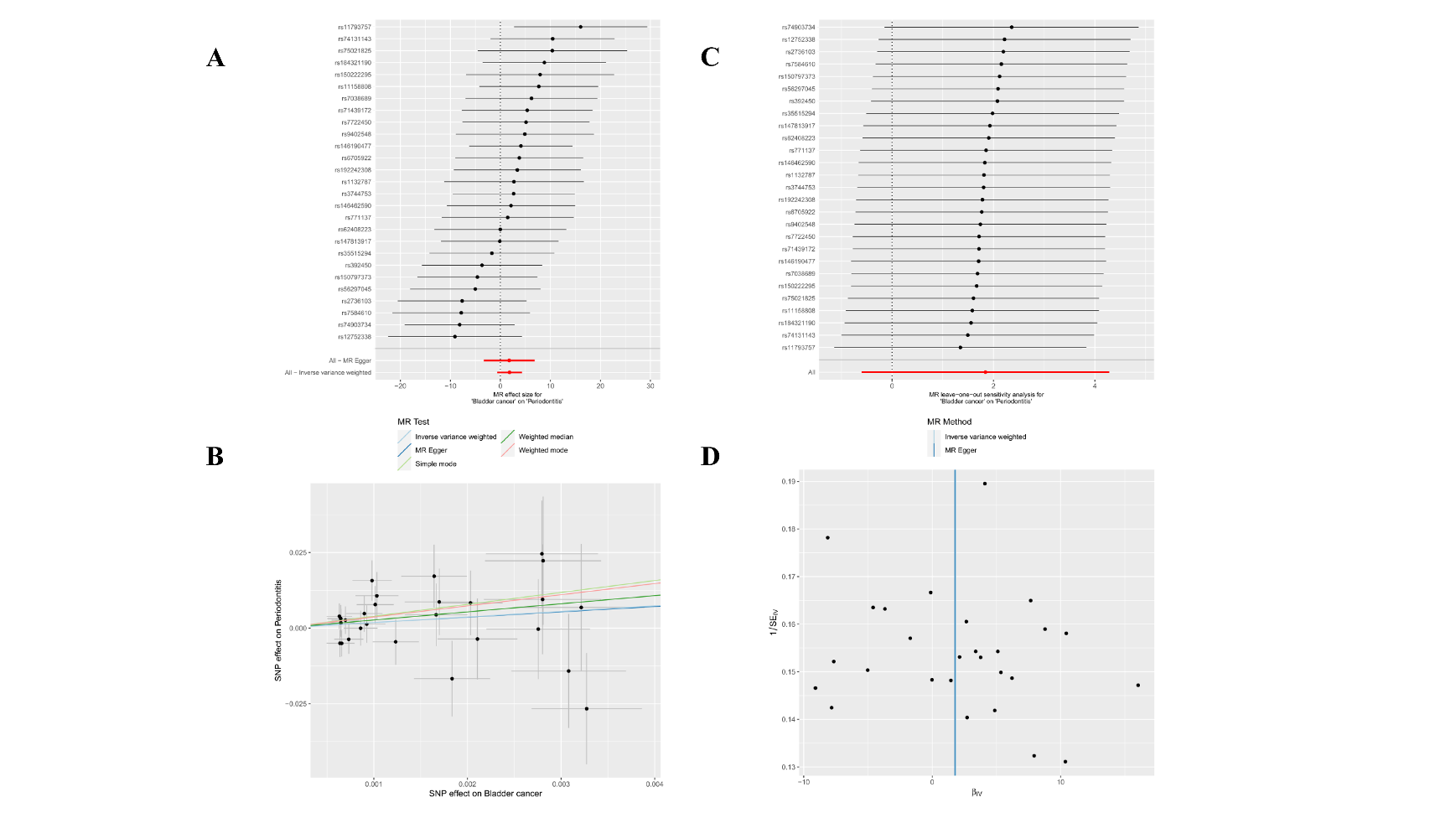
**

**Figure 2 Results and sensitivity analyses of the genetic correlation between bladder cancer and periodontitis plotted in (A) forest plot; ( B ) scatterplot; (C) Leave-one-out sensitivity test; and (D) funnel plot.**

**
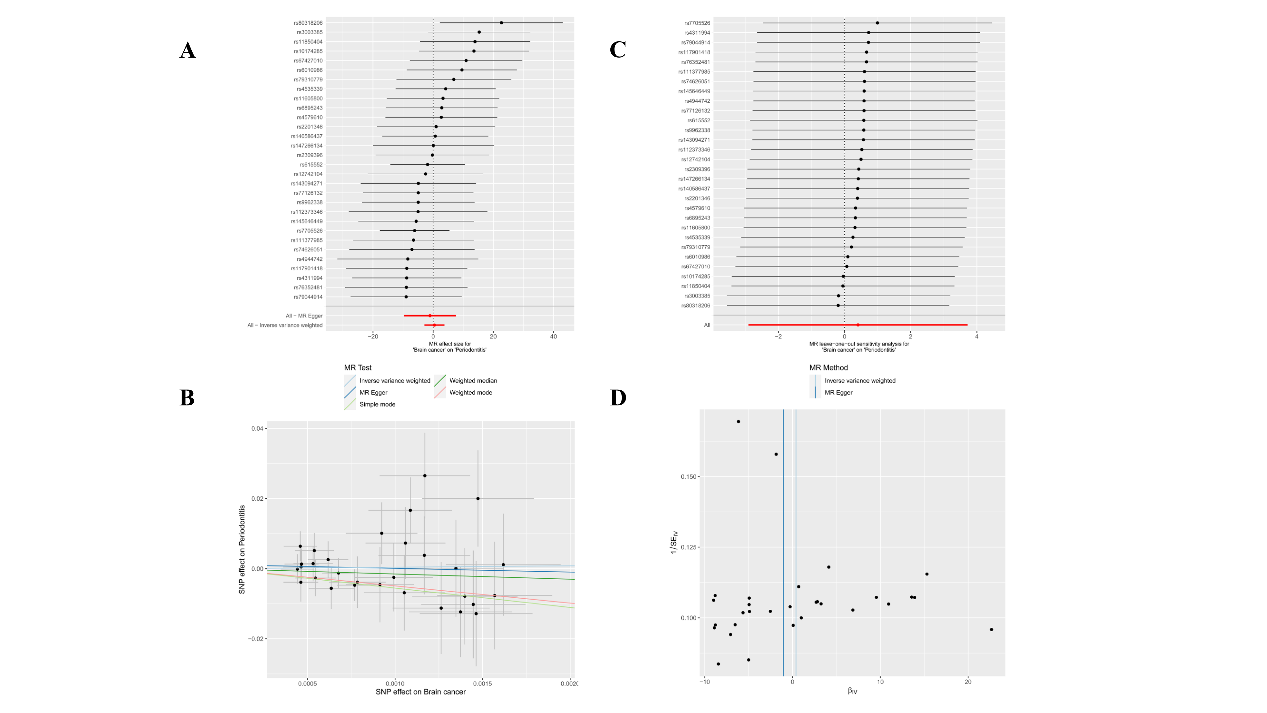
**

**Figure 3 Results and sensitivity analyses of the genetic correlation between brain cancer and periodontitis plotted in (A) forest plot; ( B ) scatterplot; (C) Leave-one-out sensitivity test; and (D) funnel plot.**

**
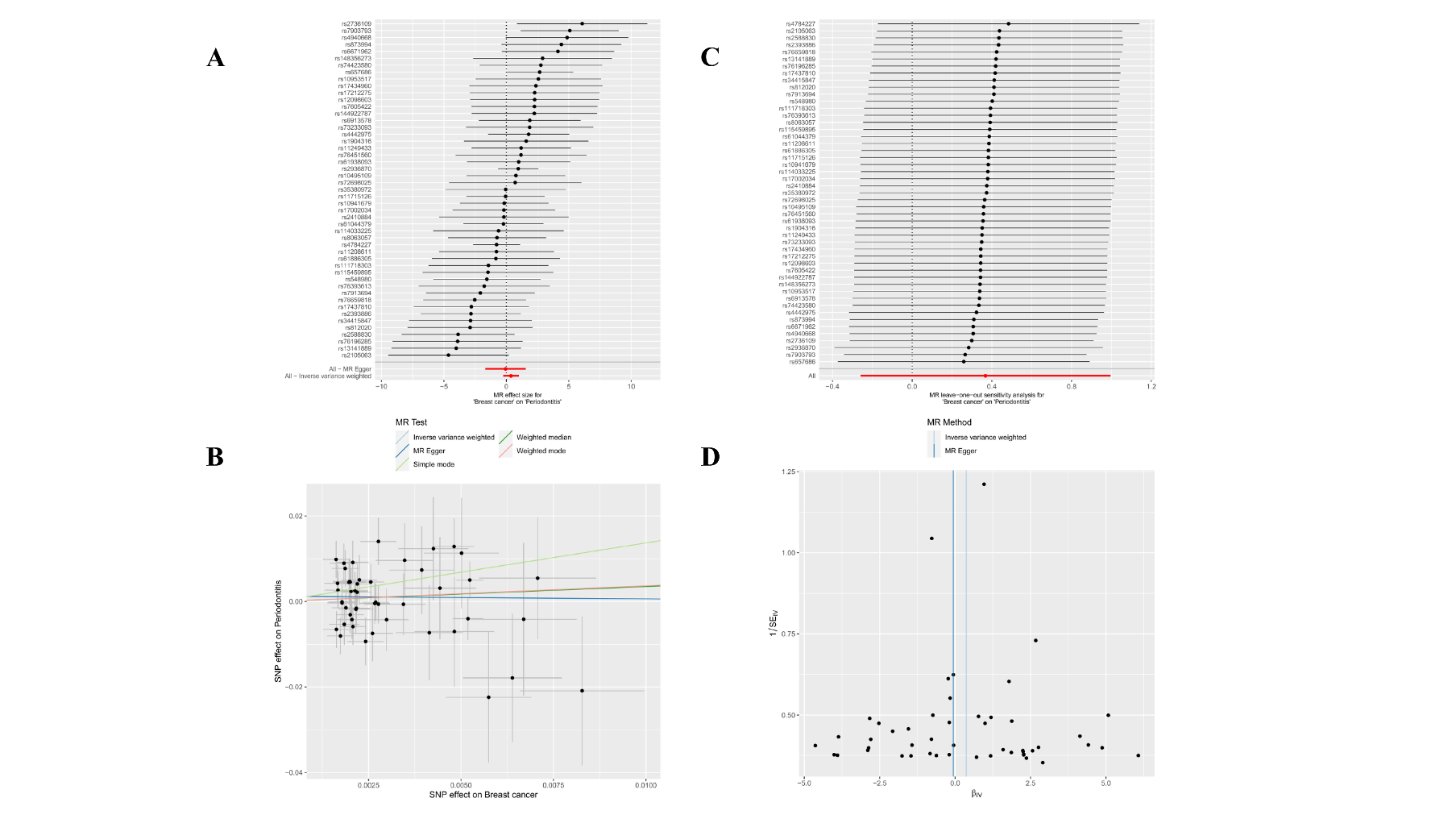
**

**Figure 4 Results and sensitivity analyses of the genetic correlation between breast cancer and periodontitis plotted in (A) forest plot; ( B ) scatterplot; (C) Leave-one-out sensitivity test; and (D) funnel plot.**

**
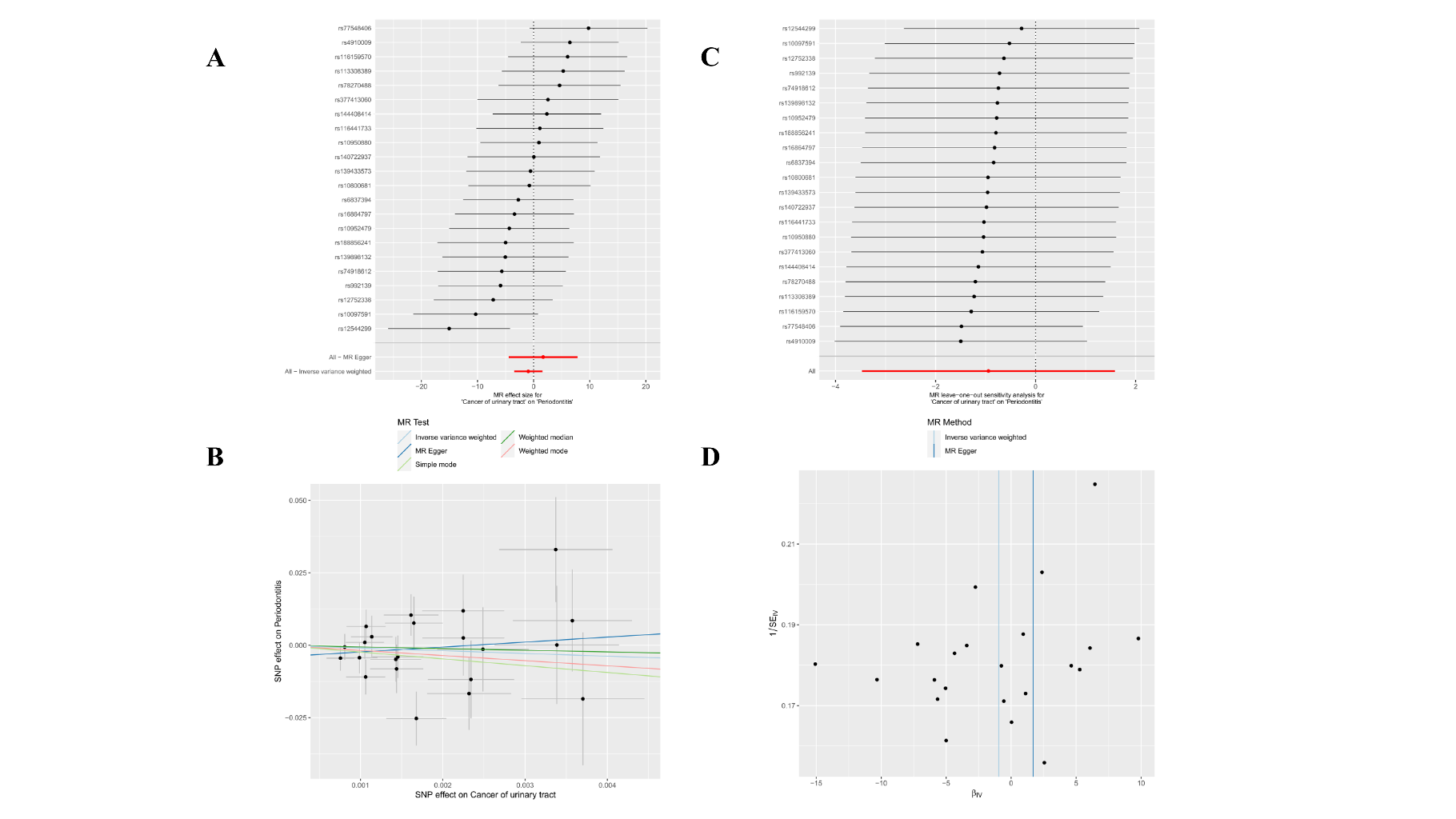
**

**Figure 5 Results and sensitivity analyses of the genetic correlation between cancer of urinary tract and periodontitis plotted in (A) forest plot; ( B ) scatterplot; (C) Leave-one-out sensitivity test; and (D) funnel plot.**

**
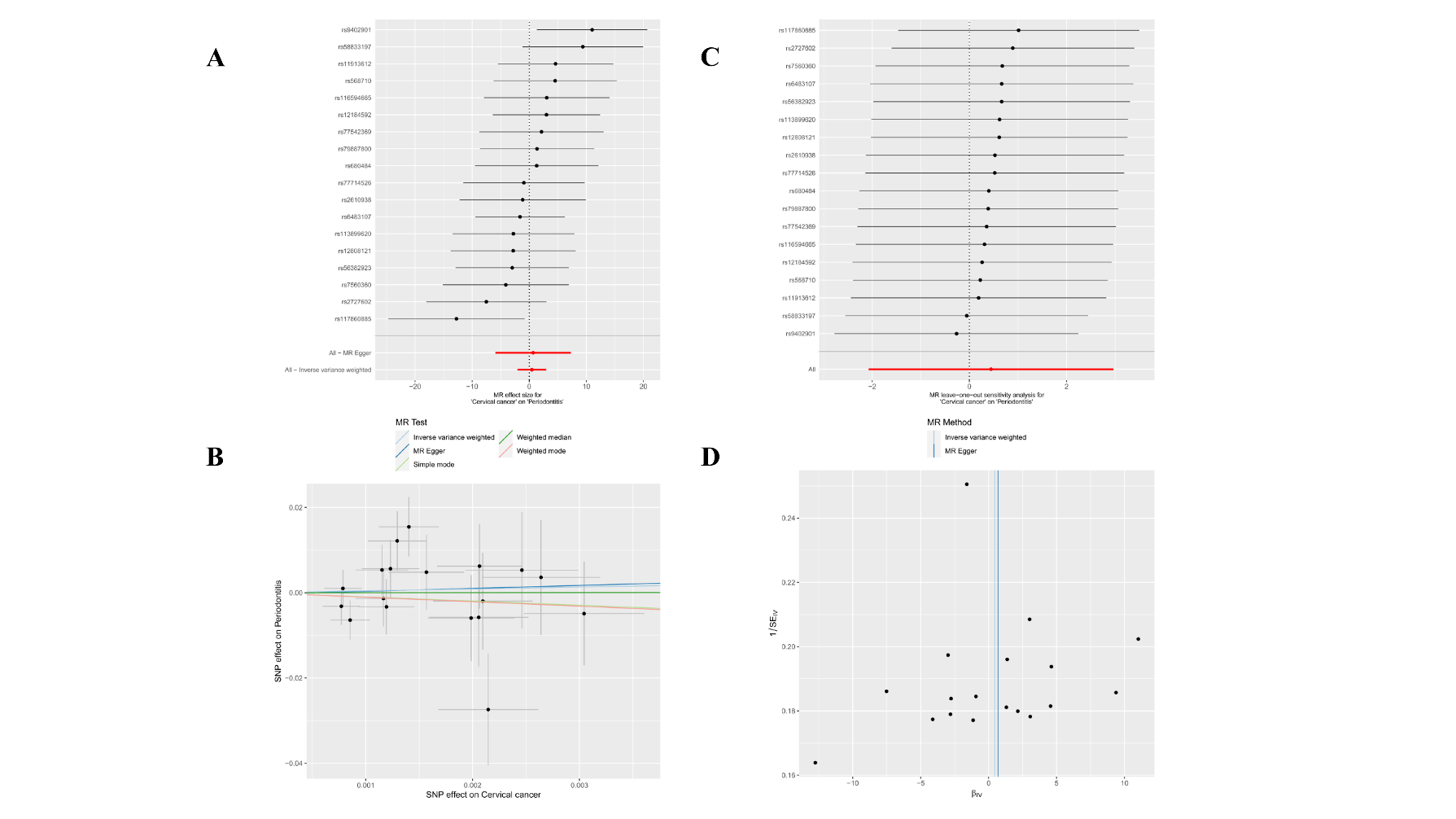
**

**Figure 6 Results and sensitivity analyses of the genetic correlation between cervical cancer and periodontitis plotted in (A) forest plot; ( B ) scatterplot; (C) Leave-one-out sensitivity test; and (D) funnel plot.**

**
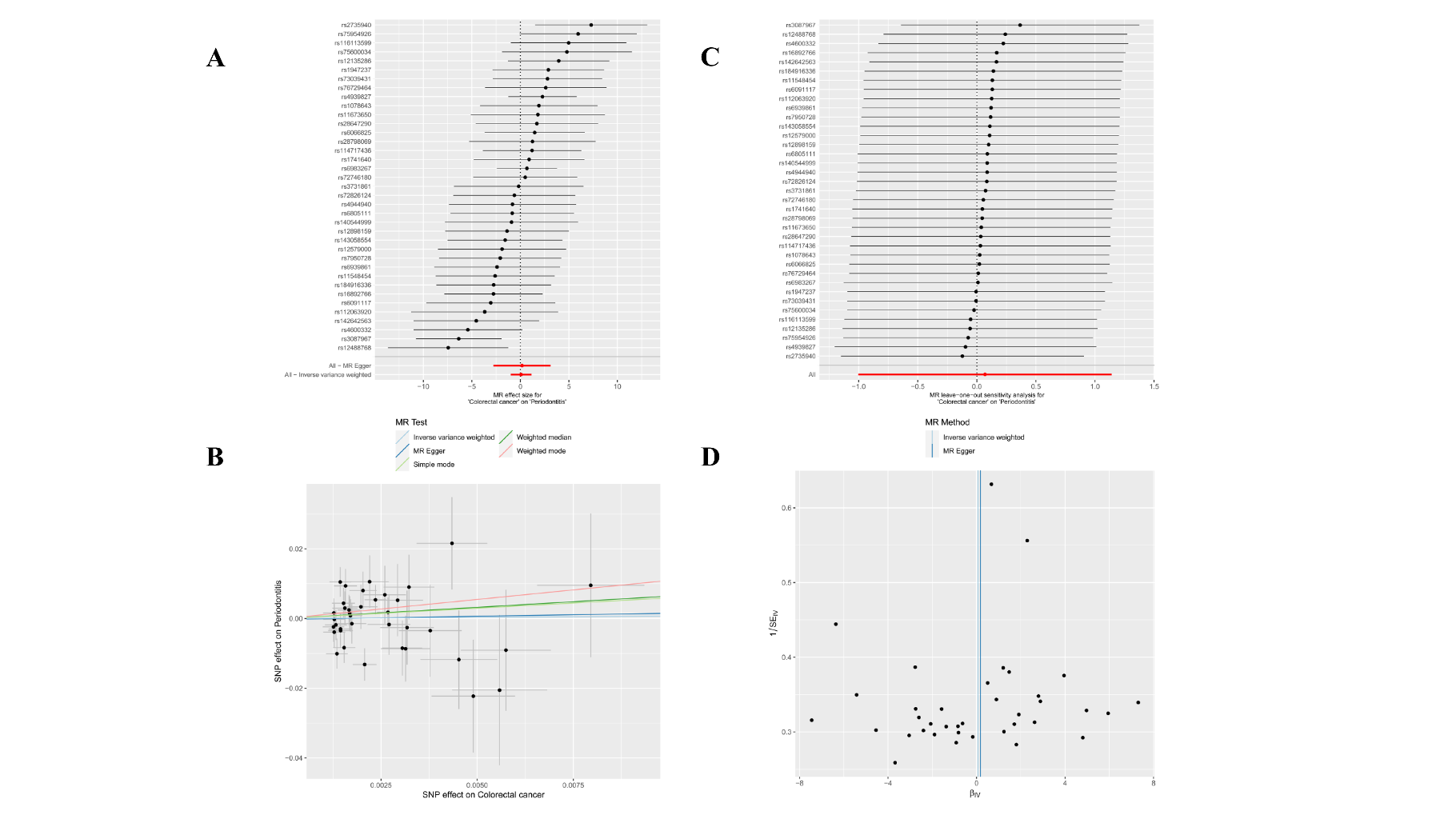
**

**Figure 7 Results and sensitivity analyses of the genetic correlation between colorectal cancer and periodontitis plotted in (A) forest plot; ( B ) scatterplot; (C) Leave-one-out sensitivity test; and (D) funnel plot.**

**
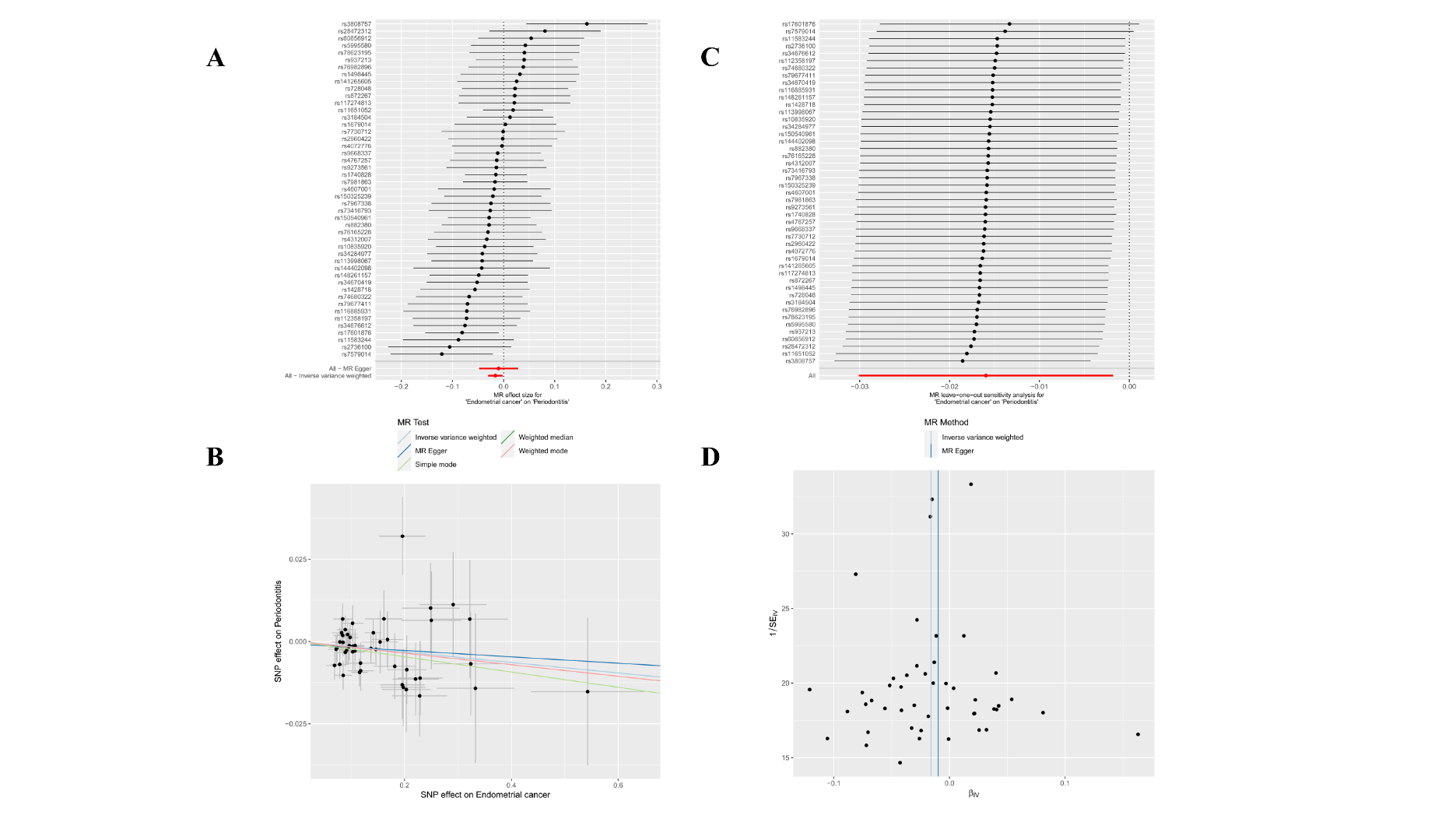
**

**Figure 8 Results and sensitivity analyses of the genetic correlation between endometrial cancer and periodontitis plotted in (A) forest plot; ( B ) scatterplot; (C) Leave-one-out sensitivity test; and (D) funnel plot.**

**
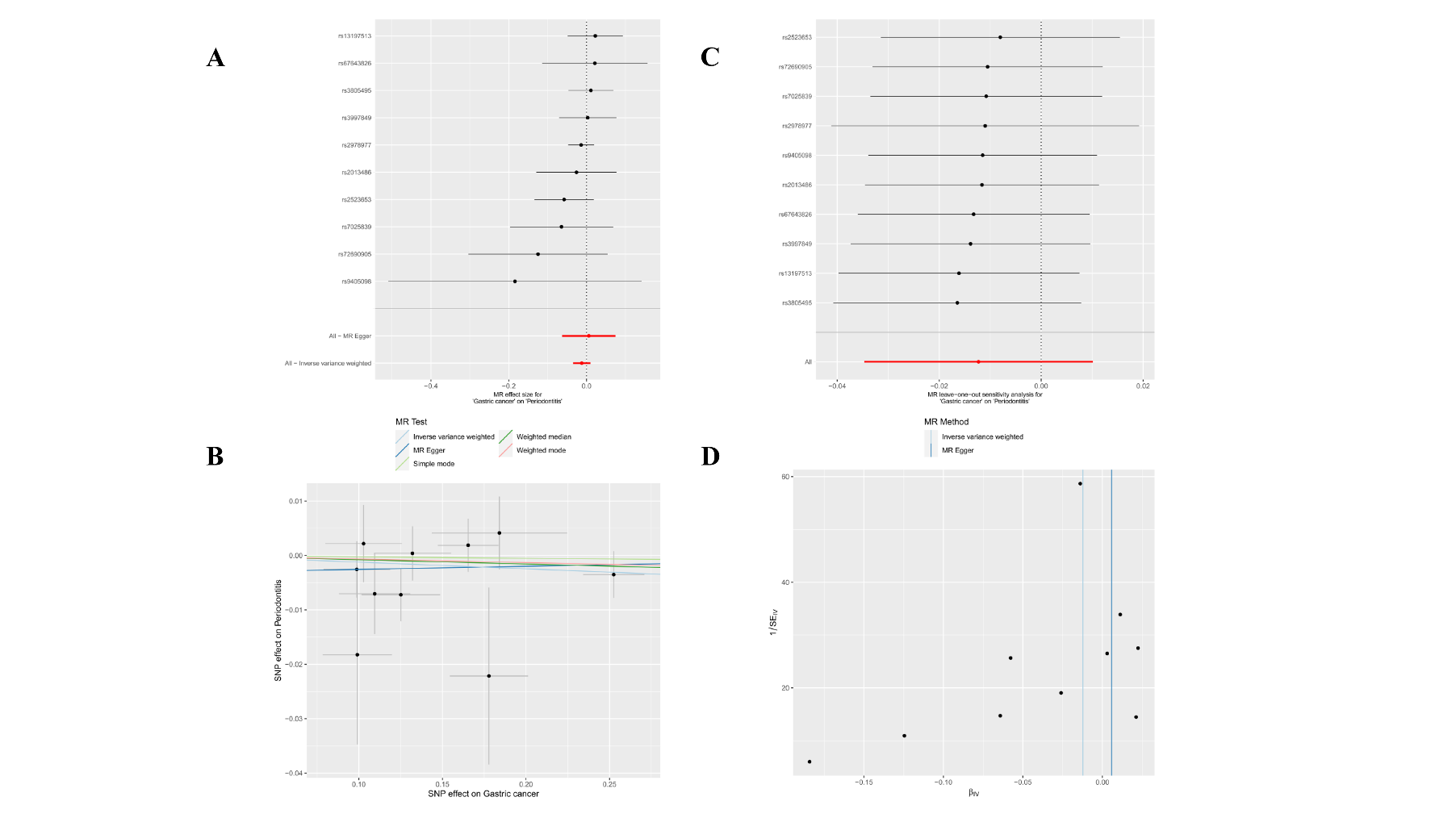
**

**Figure 9 Results and sensitivity analyses of the genetic correlation between gastric cancer and periodontitis plotted in (A) forest plot; ( B ) scatterplot; (C) Leave-one-out sensitivity test; and (D) funnel plot.**

**
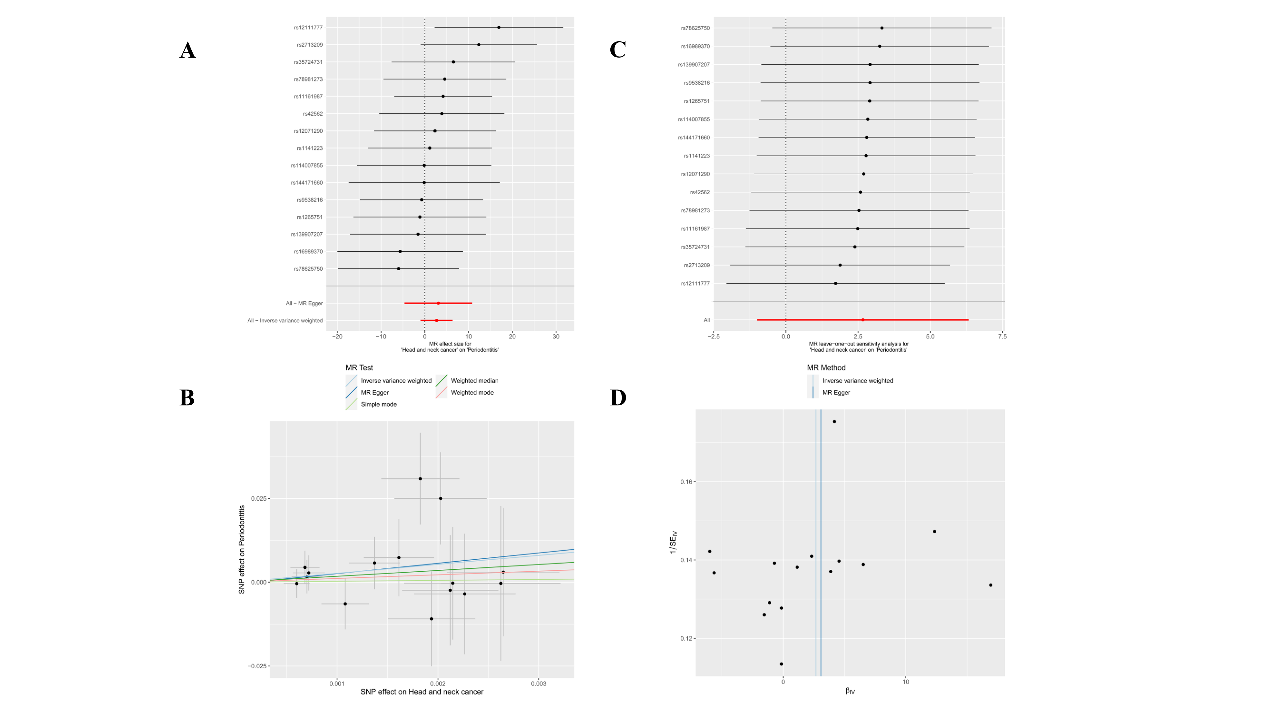
**

**Figure 10 Results and sensitivity analyses of the genetic correlation between head and neck cancer and periodontitis plotted in (A) forest plot; ( B ) scatterplot; (C) Leave-one-out sensitivity test; and (D) funnel plot.**

**
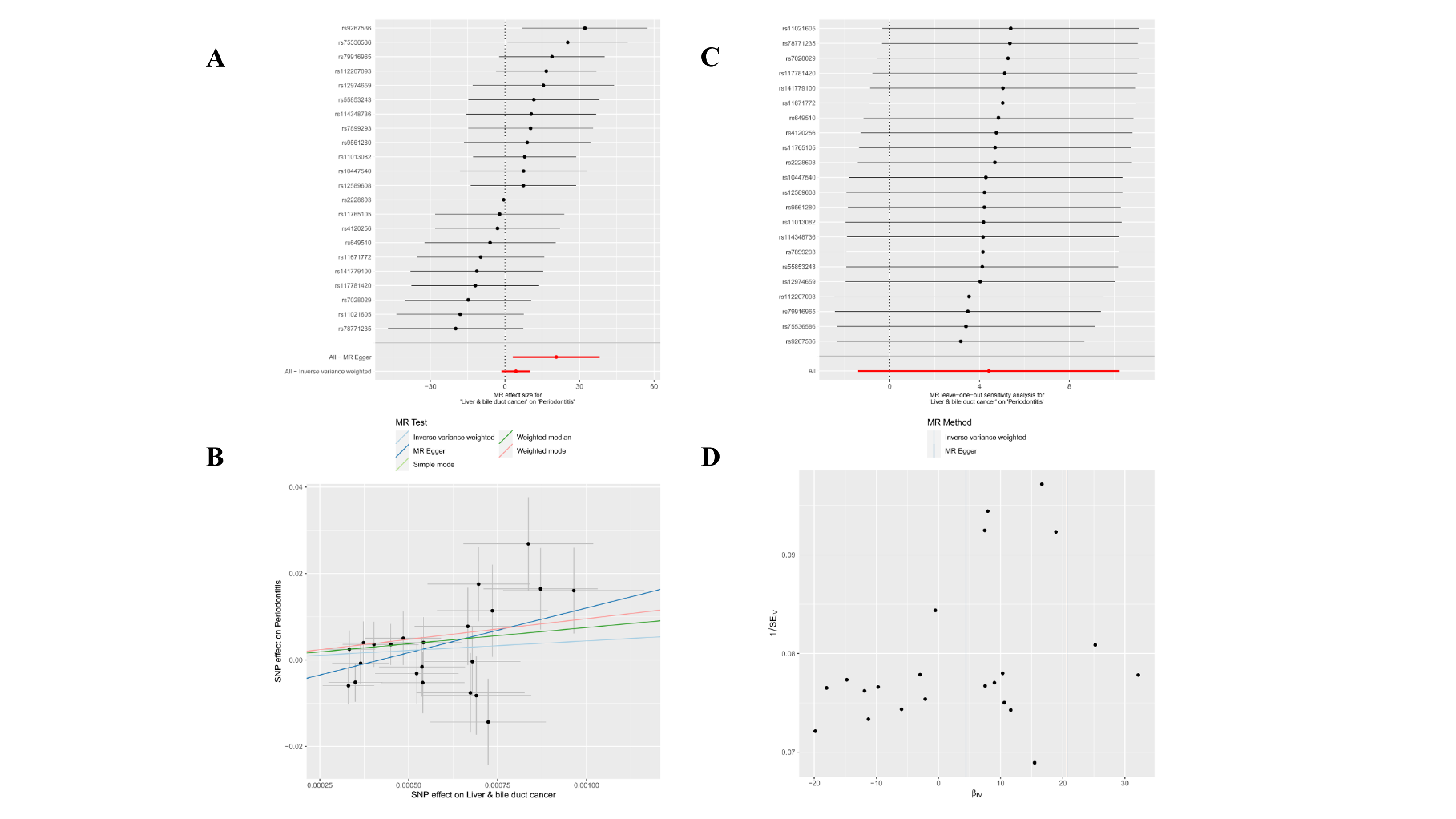
**

**Figure 11 Results and sensitivity analyses of the genetic correlation between liver & bile duct cancer and periodontitis plotted in (A) forest plot; ( B ) scatterplot; (C) Leave-one-out sensitivity test; and (D) funnel plot.**

**
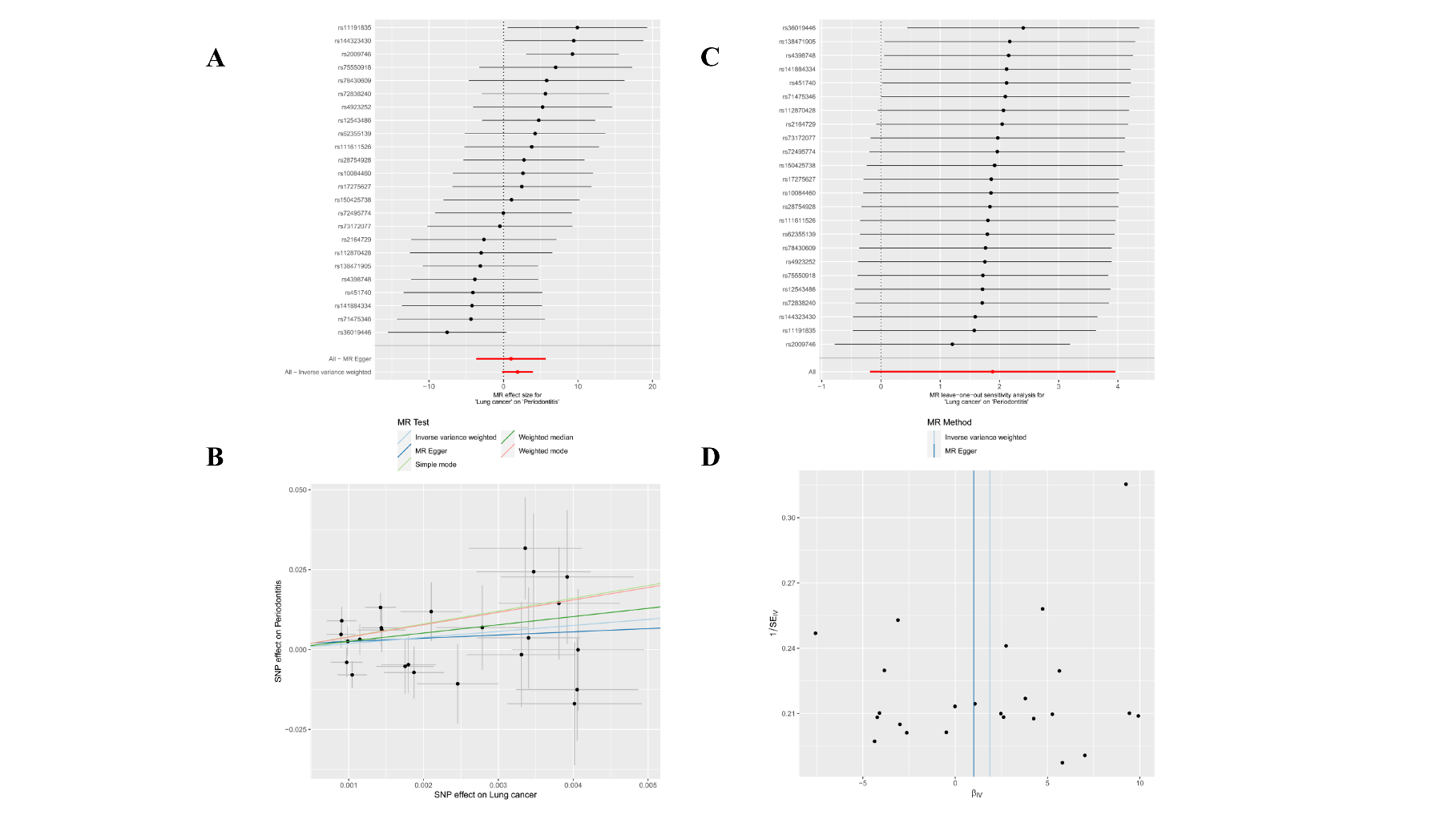
**

**Figure 12 Results and sensitivity analyses of the genetic correlation between lung cancer and periodontitis plotted in (A) forest plot; ( B ) scatterplot; (C) Leave-one-out sensitivity test; and (D) funnel plot.**

**
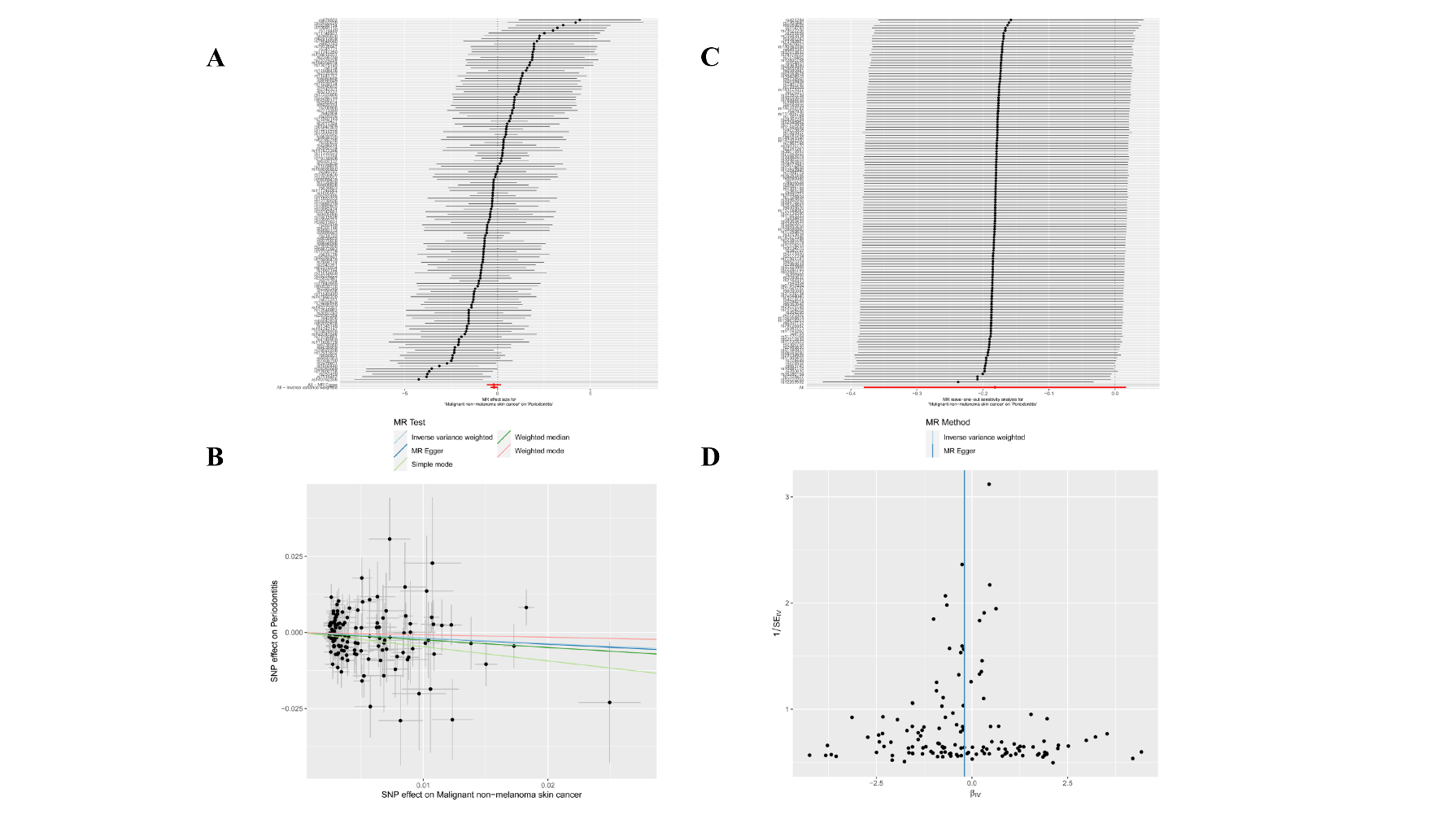
**

**Figure 13 Results and sensitivity analyses of the genetic correlation between malignant non-melanoma skin cancer and periodontitis plotted in (A) forest plot; ( B ) scatterplot; (C) Leave-one-out sensitivity test; and (D) funnel plot.**

**
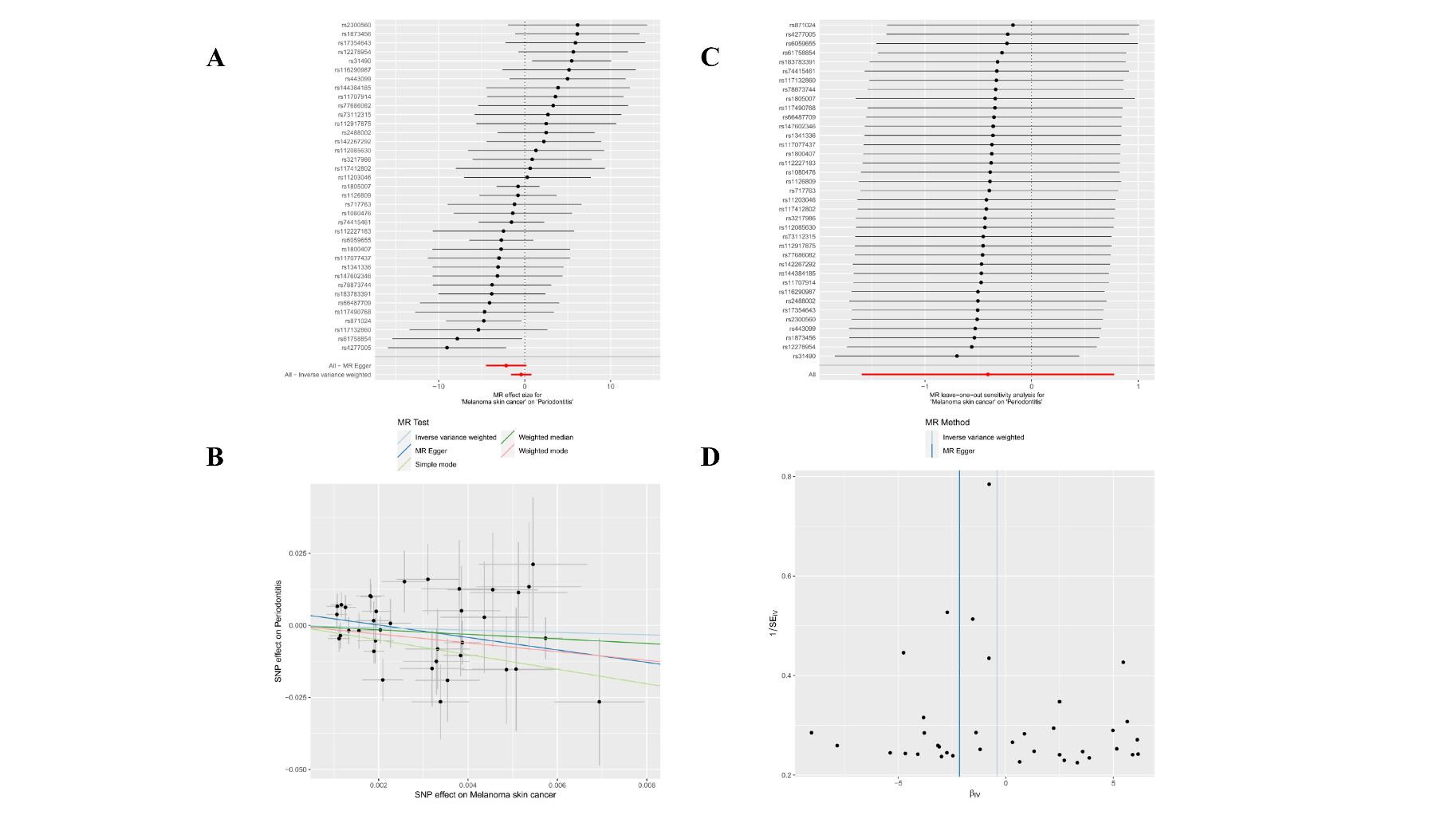
**

**Figure 14 Results and sensitivity analyses of the genetic correlation between melanoma skin cancer and periodontitis plotted in (A) forest plot; ( B ) scatterplot; (C) Leave-one-out sensitivity test; and (D) funnel plot.**

**
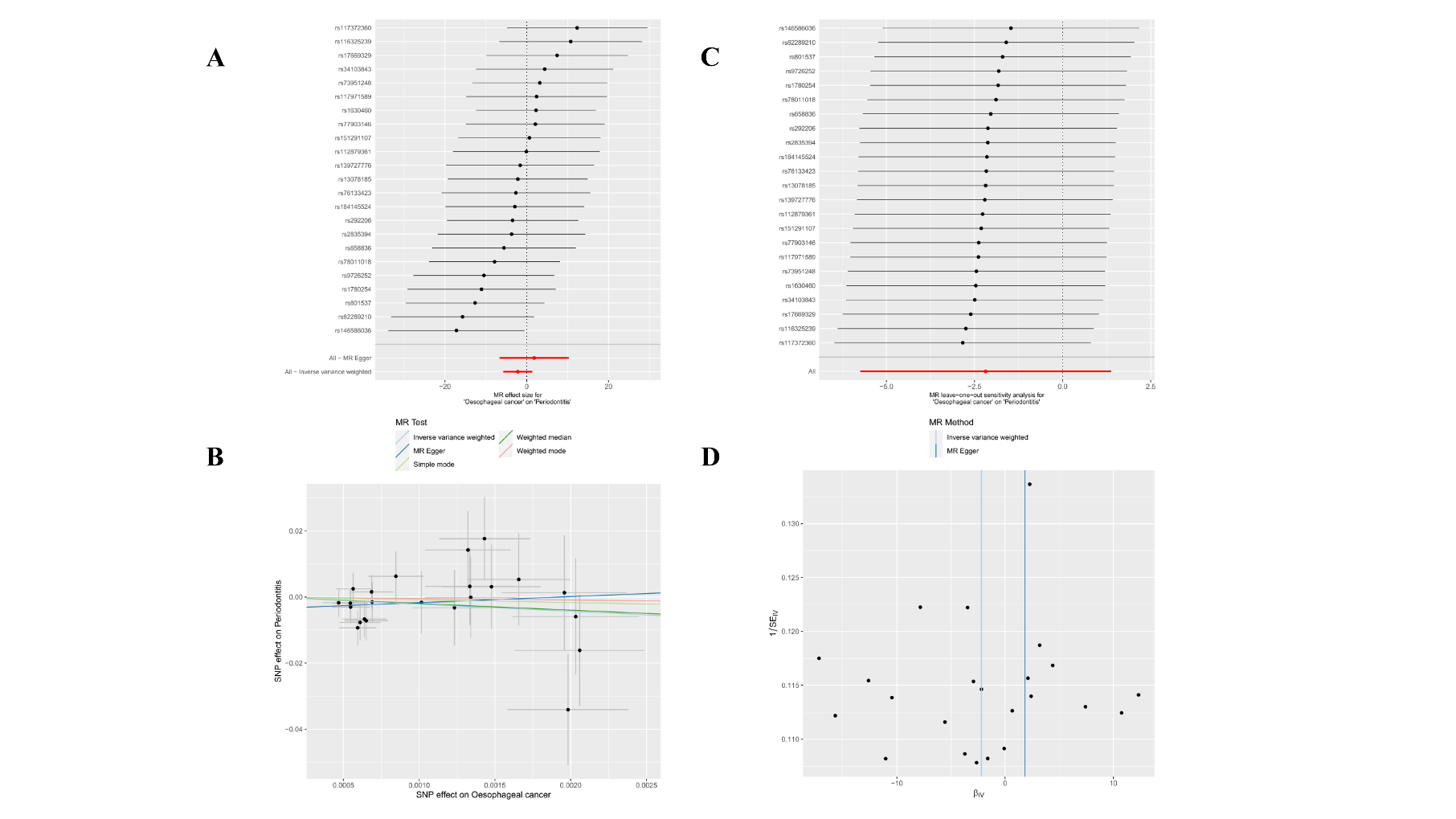
**

**Figure 15 Results and sensitivity analyses of the genetic correlation between oesophageal cancer and periodontitis plotted in (A) forest plot; ( B ) scatterplot; (C) Leave-one-out sensitivity test; and (D) funnel plot.**

**
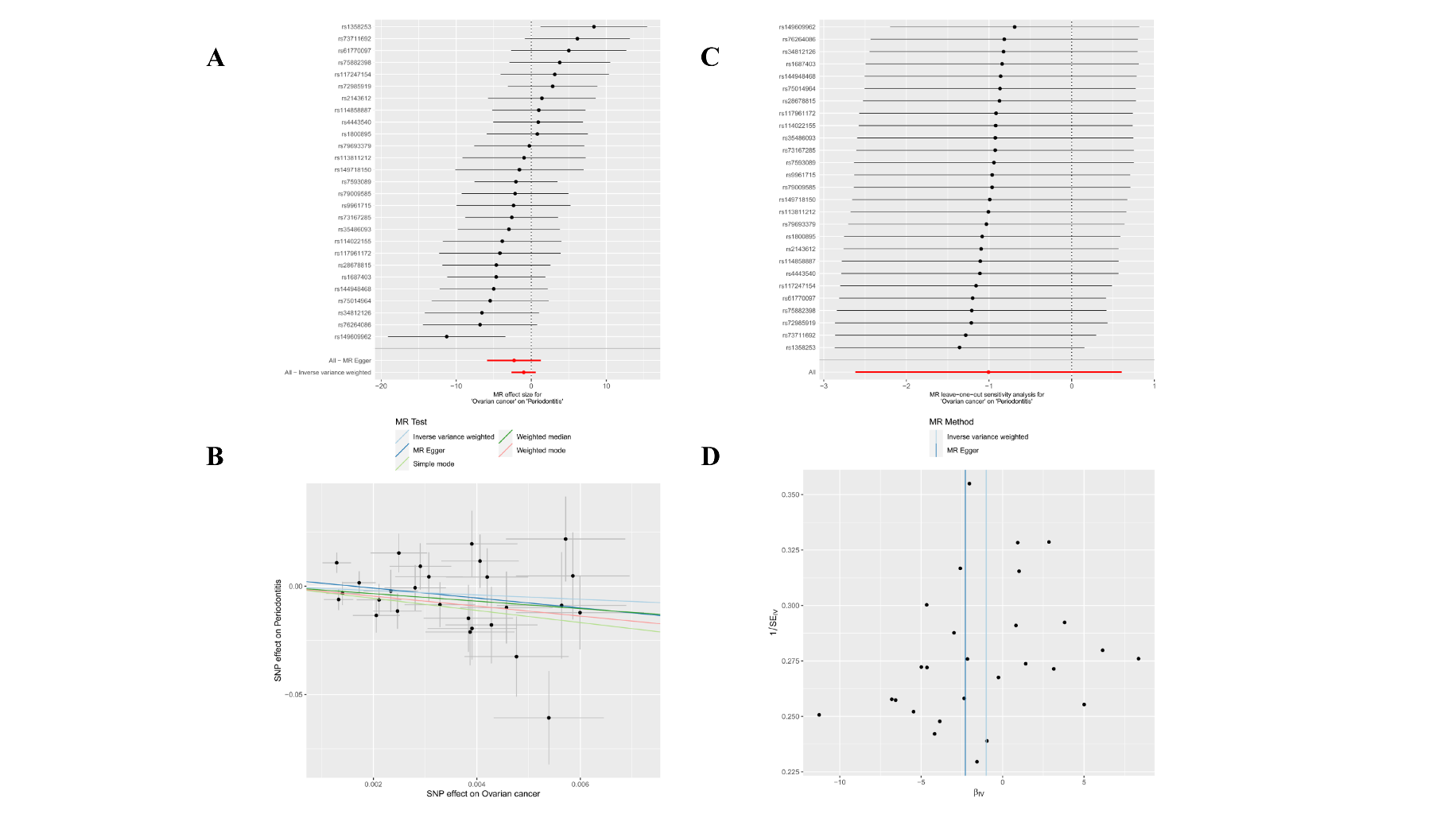
**

**Figure 16 Results and sensitivity analyses of the genetic correlation between ovarian cancer and periodontitis plotted in (A) forest plot; ( B ) scatterplot; (C) Leave-one-out sensitivity test; and (D) funnel plot.**

**
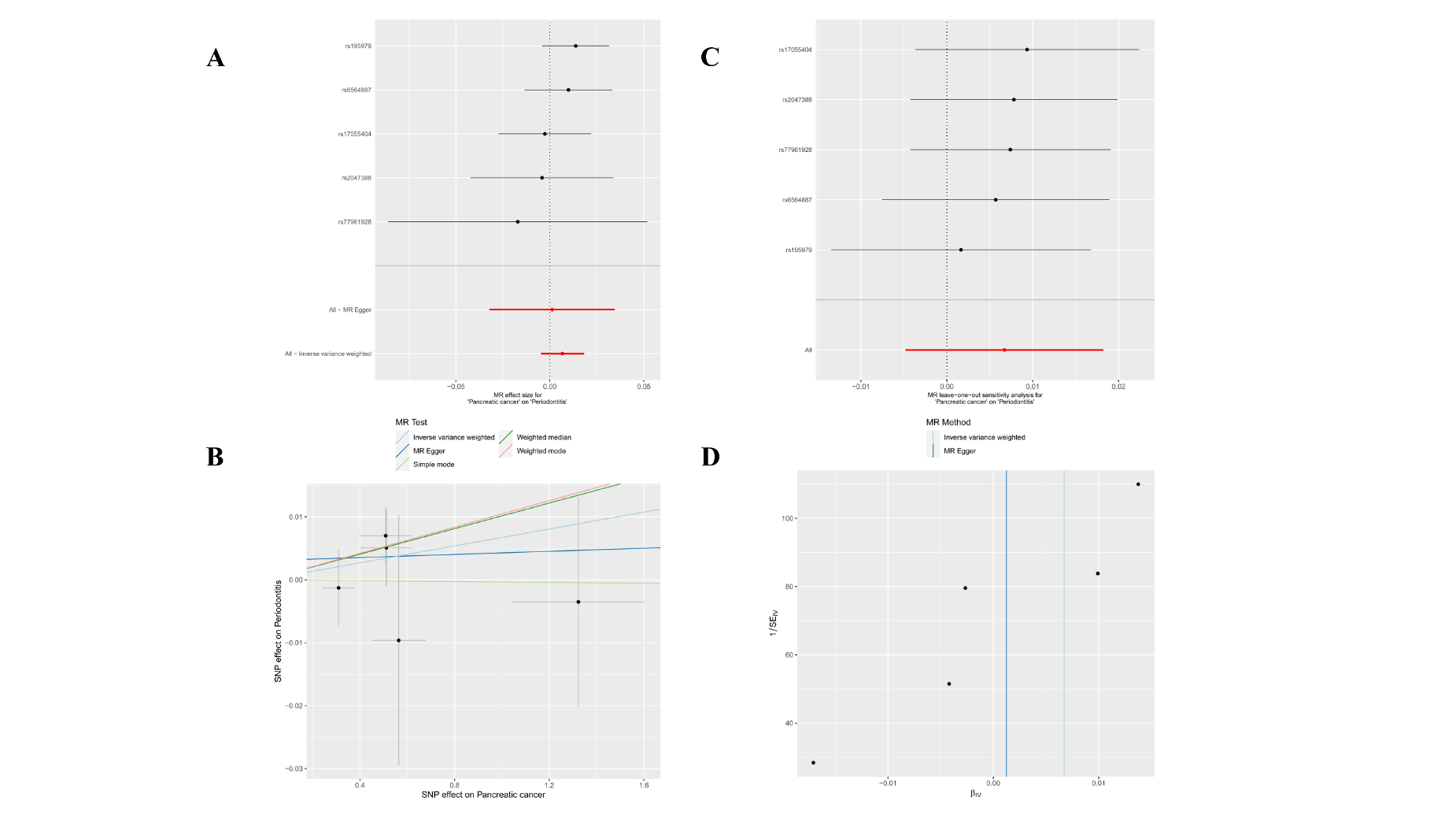
**

**Figure 17 Results and sensitivity analyses of the genetic correlation between pancreatic cancer and periodontitis plotted in (A) forest plot; ( B ) scatterplot; (C) Leave-one-out sensitivity test; and (D) funnel plot.**

**
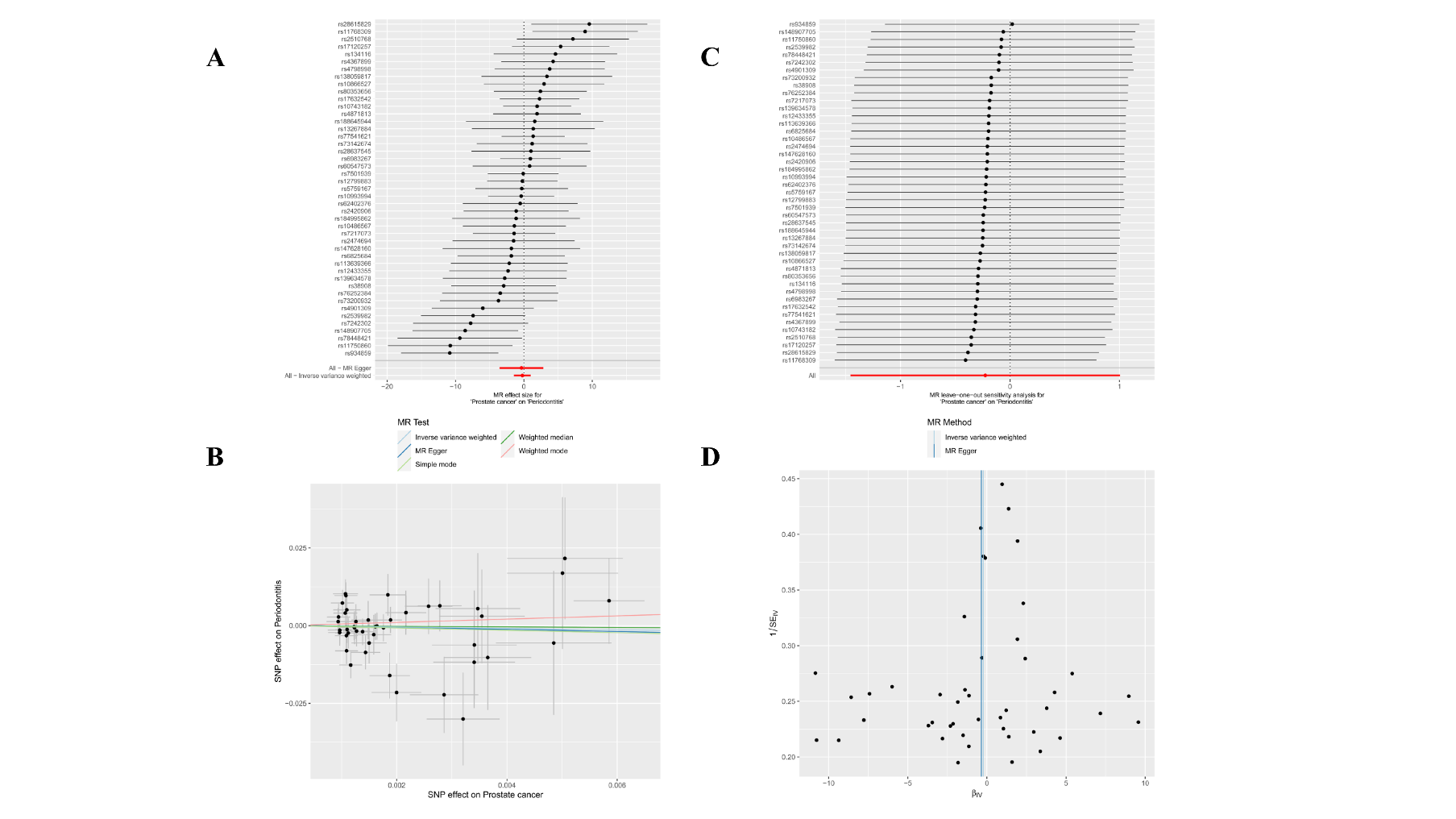
**

**Figure 18 Results and sensitivity analyses of the genetic correlation between prostate cancer and periodontitis plotted in (A) forest plot; ( B ) scatterplot; (C) Leave-one-out sensitivity test; and (D) funnel plot.**

**
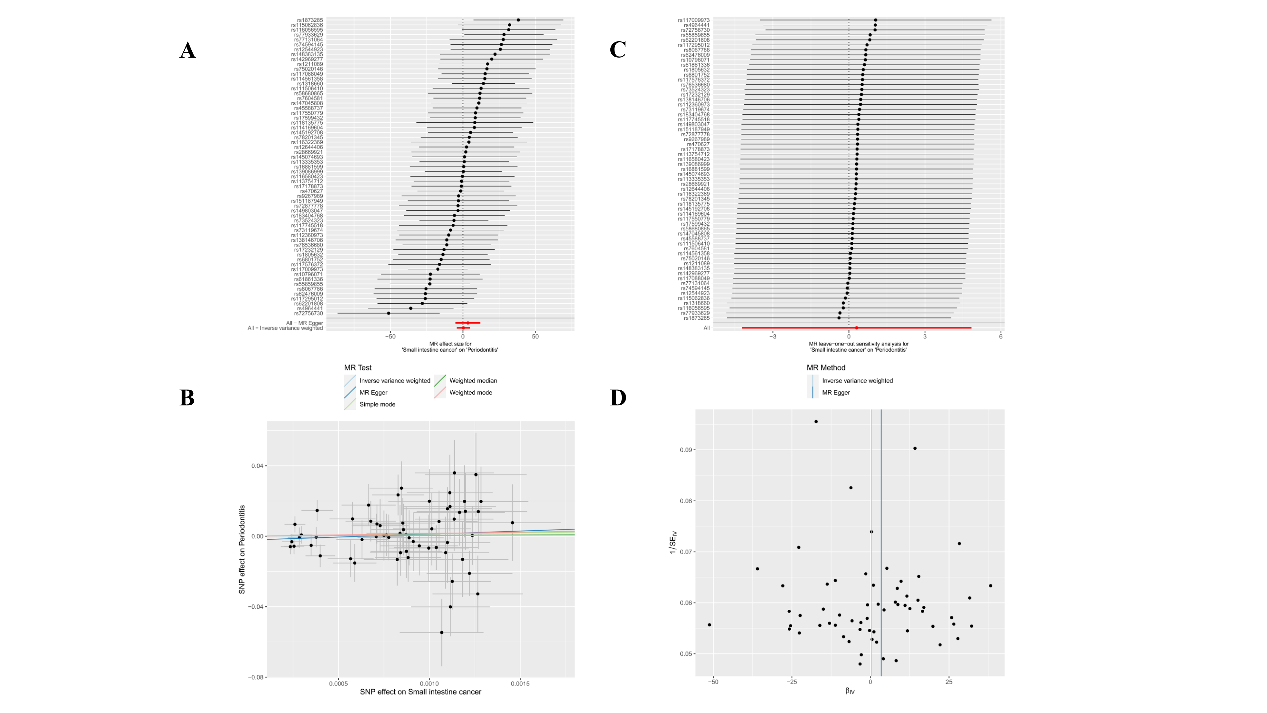
**

**Figure 19 Results and sensitivity analyses of the genetic correlation between small intestine cancer and periodontitis plotted in (A) forest plot; ( B ) scatterplot; (C) Leave-one-out sensitivity test; and (D) funnel plot.**

**
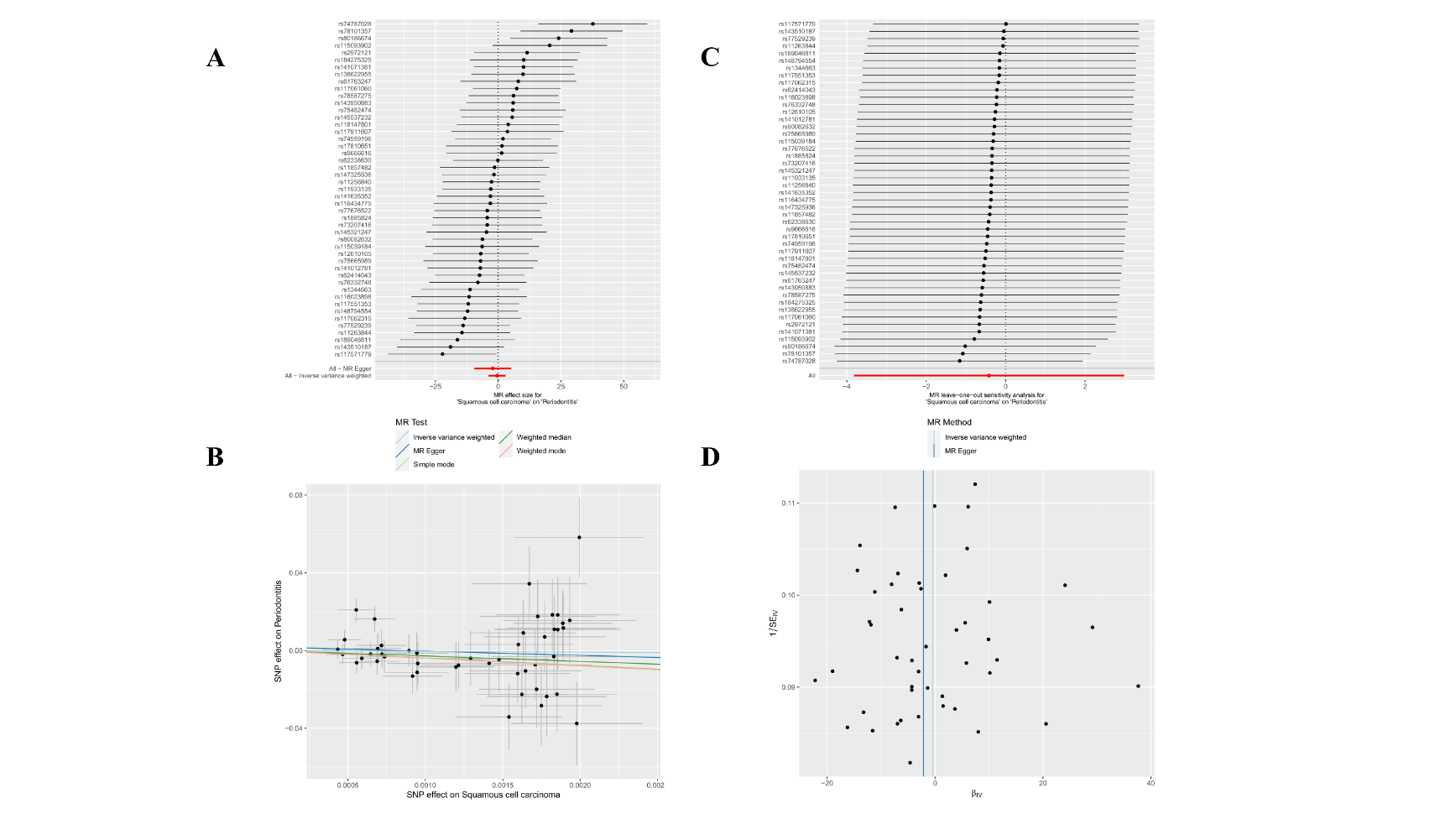
**

**Figure 20 Results and sensitivity analyses of the genetic correlation between squamous cell carcinoma and periodontitis plotted in (A) forest plot; ( B ) scatterplot; (C) Leave-one-out sensitivity test; and (D) funnel plot.**
